# DOT1L Mediated Gene Repression in Extensively Self-Renewing Erythroblasts

**DOI:** 10.3389/fgene.2022.828086

**Published:** 2022-03-23

**Authors:** Shaon Borosha, Anamika Ratri, Subhra Ghosh, Carrie A. Malcom, V. Praveen Chakravarthi, Jay L. Vivian, Timothy A. Fields, M. A. Karim Rumi, Patrick E. Fields

**Affiliations:** Department of Pathology and Laboratory Medicine, University of Kansas Medical Center, Kansas City, KS, United States

**Keywords:** DOT1L methyltransferase, Dot1l methyltransferase mutant mouse, erythroid-myeloid progenitors, erythroid-myeloid differentiation, mutant mouse models

## Abstract

DOT1L is essential for embryonic hematopoiesis but the precise mechanisms of its action remain unclear. The only recognized function of DOT1L is histone H3 lysine 79 (H3K79) methylation, which has been implicated in both transcriptional activation and repression. We observed that deletion of the mouse *Dot1L* gene (*Dot1L-*KO) or selective mutation of its methyltransferase domain (*Dot1L-*MM) can differentially affect early embryonic erythropoiesis. However, both mutations result in embryonic lethality by mid-gestation and growth of hematopoietic progenitor cells (HPCs) is similarly affected in extensively self-renewing erythroblast (ESRE) cultures established from yolk sac cells. To understand DOT1L-mediated gene regulation and to clarify the role of H3K79 methylation, we analyzed whole transcriptomes of wildtype and *Dot1L*-mutant ESRE cells. We observed that more than 80% of the differentially expressed genes (DEGs) were upregulated in the mutant ESRE cells either lacking the DOT1L protein or the DOT1L methyltransferase activity. However, approximately 45% of the DEGs were unique to either mutant group, indicating that DOT1L possesses both methyltransferase-dependent and -independent gene regulatory functions. Analyses of Gene Ontology and signaling pathways for the DEGs were consistent, with DEGs that were found to be common or unique to either mutant group. Genes related to proliferation of HPCs were primarily impacted in *Dot1L-*KO cells, while genes related to HPC development were affected in the *Dot1L-*MM cells. A subset of genes related to differentiation of HPCs were affected in both mutant groups of ESREs. Our findings suggest that DOT1L primarily acts to repress gene expression in HPCs, and this function can be independent of its methyltransferase activity.

## Introduction

DOT1L histone methyltransferase (DOT1L) is an essential regulator of vital tissue and organ development during embryonic life, including hematopoiesis (Feng et al., 2010). We observed that loss of DOT1L in mice (*Dot1L*-KO) results in lethal anemia during mid-gestation (Feng et al., 2010). DOT1L is the only known methyltransferase in eukaryotic cells to methylate lysine 79 of histone H3 (H3K79) (Feng et al., 2002). We generated a mouse line carrying a point mutation (Asn241Ala) in the mouse *Dot1l* gene (*Dot1l*-MM) that renders its catalytic domain inactive (Min et al., 2003; Malcom et al., 2022). *Dot1l*-MM mice expressed an intact DOT1L protein that lacked only the H3K79 methyltransferase activity (Malcom et al., 2022). The methyltransferase mutant, *Dot1l*-MM mice, were also embryonic lethal. The embryos died around mid-gestation (Malcom et al., 2022). The mice also displayed defects in embryonic hematopoiesis, including a decreased ability to form definitive myeloid, and oligopotent (mixed) blood progenitors in *ex vivo* cultures (Malcom et al., 2022). However, unlike the *Dot1L* knockout (*Dot1l-*KO) (Feng et al., 2010), *Dot1L-*MM embryos were not anemic at E10.5 and hematopoietic progenitor cells (HPC) from *Dot1l-*MM yolk sacs were able to produce erythroid colonies in numbers similar to the wildtype (Malcom et al., 2022).

Histone methylation is important for permissive or repressive chromatin conformation and can have a profound effect on regulation of gene expression (Jambhekar et al., 2019). DOT1L is responsible for the mono-, di- and tri-methyl marks on lysine 79 of histone H3 (H3K79) (Feng et al., 2002). These histone modifications as well as the DOT1L protein have been strongly associated with actively transcribed chromatin regions (Steger et al., 2008). Thus, it has been suggested that DOT1L is involved in epigenetic regulation of transcriptional activation of genes in a tissue-specific manner.

In this study, we examined the expression of DOT1L-regulated genes on embryonic day 10.5 (E10.5) yolk sac (YS) derived hematopoietic progenitor cells (HPC). We observed that more than 82% of the differentially expressed genes (DEGs) in *Dot1l*-KO or *Dot1l*-MM HPCs cultured *ex vivo,* were upregulated, suggesting that DOT1L primarily acts to repress transcription in yolk sac HPCs.

## Methods

### Dot1L Mutant Mouse Models

The *Dot1L*-KO mice were generated and maintained as described previously (Feng et al., 2010). To produce the *Dot1L*-MM mouse, we generated mutant mESC (Malcom et al., 2022). Briefly, mESCs (E14TG2a) were targeted with CRISPR/Cas9 to introduce a point mutation (Asp241Ala) within exon 9 of *Dot1L.* This mutation lies in the methyltransferase domain of *Dot1L* and has been demonstrated to eliminate DOT1L catalytic activity without altering the overall protein structure (Min et al., 2003). Allele-specific PCR of genomic DNA was used identify the mutated mESC clones. Biallelic mutant mES clones were evaluated for H3K79 di-methylation (H3K79me2) by Western blot analysis (Malcom et al., 2022). This analysis indicated that DOT1L methyltransferase activity was absent in these cells. These results were confirmed in the *Dot1L-*MM embryos (Malcom et al., 2022). *Dot1L*-KO and *Dot1L-*MM heterozygous mice were maintained by continuous backcrossing to 129 stocks. Genotyping was performed on tail clips by using RED extract-N-Amp Tissue PCR Kit (Sigma-Aldrich) as previously described (Rumi et al., 2014; Rumi et al., 2017). Genotyping primer sequences are shown in Supplementary Tables S1, S2. All animal experiments were performed in accordance with the protocols approved by the University of Kansas Medical Center Animal Care and Use Committee.

### Extensively Self-Renewing Erythroblasts (ESRE)

*Dot1L*-KO or *Dot1L*-MM heterozygous mutant males and females were set up for timed mating to collect the conceptuses on E10.5. Pregnant females were sacrificed, and uteri were dissected to separate embryos and yolk sacs. Embryos were treated with RED extract-N-Amp Tissue PCR reagents (Millipore Sigma, Saint-Louis, MO) to purify genomic DNA and perform the genotyping PCR.

Digested E10.5 yolk sacs were washed in IMDM, resuspended in 1 ml ESRE culture media, and plated into gelatin-coated, 12 well plates for ESRE culture, following a previously published protocol (England et al., 2011). ESRE culture media consisted of StemPro34 media containing nutrient supplement (Gibco/BRL), 2 U/ml human recombinant EPO (University of Kansas Hospital Pharmacy), 100 ng/ml SCF (PeproTech), 10^–6^ M dexamethasone (Sigma), 40 ng/ml insulin-like growth factor-1 (PeproTech) and penicillin-streptomycin (Invitrogen). After 1 day of culture, the nonadherent cells were aspirated, washed, resuspended in fresh ESRE media, and transferred to a new gelatin-coated well. After an additional 2 days in culture, total RNA was extracted from wildtype, *Dot1L*-KO or *Dot1L*-MM HPCs using TRI Reagent (Sigma-Aldrich) following the manufacturer’s instructions.

### Assessment of Cell Proliferation, Cell Cycle Analysis and Apoptosis Assays

Single-cell suspensions from E10.5 yolk sacs were cultured in MethoCult™ GF M3434 (StemCell Technologies, Vancouver, BC, Canada) for 4 days. The mix of cytokines in this methylcellulose medium promotes definitive erythroid, myeloid, and mixed progenitor differentiation. Cells were collected on day 4 and stained with Annexin V to assess apoptosis. Some cells were fixed by adding cold 70% ethanol slowly to single cell suspensions, and then stained with propidium iodide (Belloc et al., 1994). Flow cytometry was performed using a FACSCalibur (BD Biosciences, San Jose, CA) (Krishan, 1975). Analyses of the cytometric data were carried out using CellQuest Pro software (BD Biosciences) (Sen et al., 2007; Chiruvella et al., 2008; Sutherland et al., 2009).

### Sample Collection, Library Preparation and RNA-Sequencing

RNA quality was assessed by a Bioanalyzer at the KUMC Genomics Core, and samples with RIN values over 9 were selected for RNA-sequencing library preparation. RNA samples were extracted from multiple, expanded yolk sac cells obtained from embryos of the same genotype. These samples were pooled to prepare each RNA-seq library. 500 ng of total RNA was used to prepare an RNA-seq library using the True-Seq mRNA kit (Illumina, San Diego, CA) as described previously (Khristi et al., 2018a; Chakravarthi et al., 2019; Chakravarthi et al., 2020a). The quality of RNA-seq libraries was evaluated by Agilent Analysis at the KUMC Genomics Core and the sequencing was performed on an Illumina NovaSeq 6000 sequencer (KUMC Genomics Core).

### RNA-Seq Data Analysis

RNA-sequencing data were demultiplexed, trimmed, aligned, and analyzed using CLC Genomics Workbench 12.2 (Qiagen Bioinformatics, Germantown, MD) as described previously (Khristi et al., 2018a; Chakravarthi et al., 2019; Chakravarthi et al., 2020a). Through trimming, low-quality reads were removed, and good-quality reads were aligned with the *Mus musculus* genome (mm10) using default guidelines: 1) maximum number of allowable mismatches = 2, 2) minimum length and similarity fraction = 0.8, and 3) minimum number of hits per read = 10. Gene expression values were measured in transcripts per million (TPM). DEGs were identified that had an absolute fold change of TPM ≥2 and a false discovery rate (FDR) *p*-value of ≤0.05.

### Gene Ontology and Disease Pathway Analysis for the RNA-Sequencing Data

DEGs were subjected to Gene Ontology (GO) analysis (http://www.pantherdb.org) and categorized for biological, cellular and molecular function. DEGs in *Dot1L-*mutant HPCs were further analyzed by Ingenuity Pathway Analysis (IPA; Qiagen Bioinformatics, Germantown, MD) to build gene networks related to blood development. Functional analyses were performed towards understanding the biological pathways and functions altered in either of the *Dot1L* mutant progenitor cells.

### Validation of RNA-Sequencing Data

DEGs were validated by RT-qPCR. RT-qPCR validation included cDNA samples prepared with wildtype, *Dot1L*-MM and *Dot1L*-KO ESRE cell-derived total RNA. cDNAs were reverse transcribed from 1 μg of total RNA by using Applied Biosystems High-Capacity cDNA Reverse Transcription Kits (Thermo Fisher Scientific). Real-time RT-qPCR amplification of cDNAs was carried out in a 20 μL reaction mixture containing Applied Biosystems Power SYBR Green PCR Master Mix (Thermo Fisher Scientific). The genes were selected from the IPA analyses and MGI data that impacted the proliferation and differentiation of HPCs. All PCR primers were designed using Primer 3 (Untergasser et al., 2007) and the primer sequences are shown in Supplementary Table S3. Amplification and fluorescence detection of qRT-PCR were carried out on Applied Biosystems QuantStudio 7 Flex Real Time PCR System (Thermo Fisher Scientific). The results of RT-qPCR were normalized to *Rn18s* expression and calculated by the comparative ΔΔCT method (Khristi et al., 2018b; Khristi et al., 2019; Chakravarthi et al., 2020b).

### Statistical Analysis

Each RNA-seq library or cDNA was prepared from pooled RNA samples extracted from at least 3 different ESRE cultures of the same genotype. Each group for RNA sequencing consisted of three independent libraries and the differentially expressed genes (DEGs) were identified by CLC Genomics workbench as described previously (Khristi et al., 2018a; Chakravarthi et al., 2019; Chakravarthi et al., 2020a). In CLC Genomics Workbench, DEGs are determined by an inbuilt “Differential Expression for RNA-seq tool” that performs a multi-factorial statistic on the set of expression data across groups (like ANOVA) for comparison (Khristi et al., 2018a). RT-qPCR validation included at least six cDNA samples prepared from wildtype, *Dot1L*-KO and *Dot1L*-MM ESRE cell total RNA. The experimental results are expressed as mean ± standard error (SE). The RT-qPCR results were analyzed by one-way ANOVA, and the significance of mean differences were determined by Duncan’s *post hoc* test, with *p* ≤ 0.05. All the statistical calculations were done using SPSS 22 (IBM, Armonk, NY).

## Results

### Dot1L-KO and Dot1L-MM Embryos Exhibit Distinct Hematopoietic Phenotypes

**FIGURE 1:**
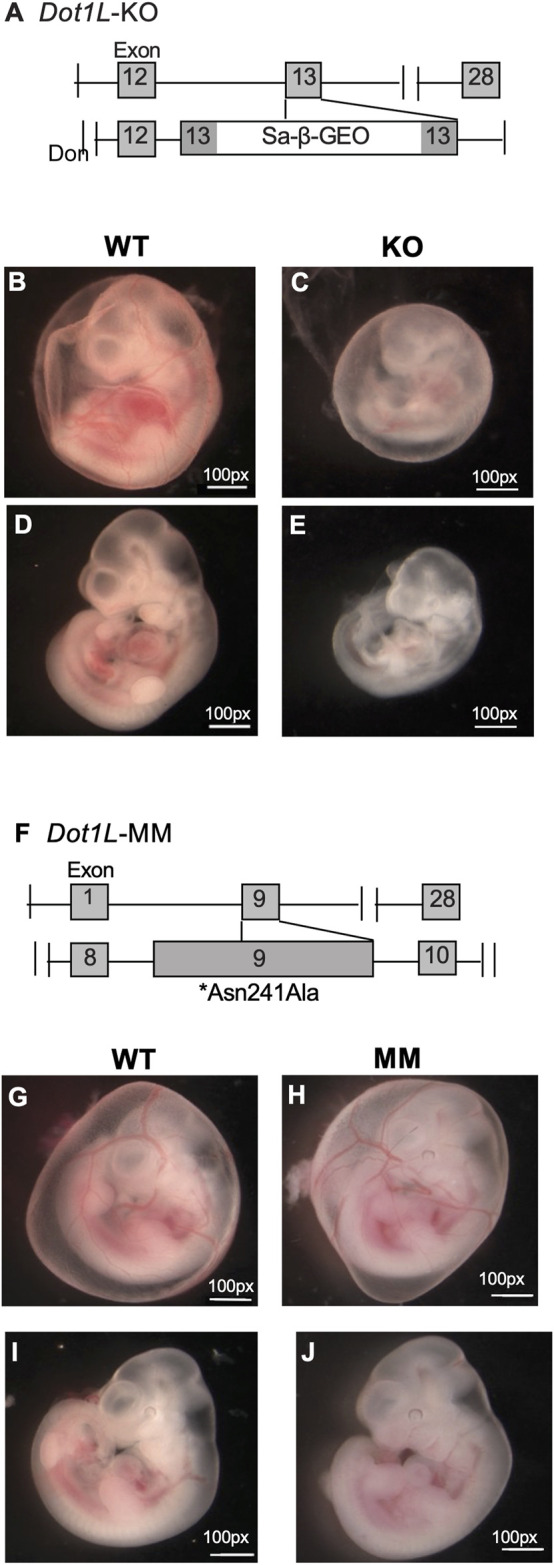
Differential phenotypes of *Dot1L*-KO and *Dot1L*-MM embryos. Schematic representation of the gene trap construct used to generate the mutant *Dot1L*-KO alleles **(A)**. Representative images of E10.5 wildtype (WT) **(B,D)**, *Dot1L*-KO **(C,E)**, show that growth of *Dot1L*-KO embryos is slower than that of WT **(B–E)**. In addition, at E10.5, the KO embryos demonstrated significant anemia, as there was noticeably less blood in both the yolk sac vasculature **(B,C)**, as well as the body of the embryo **(D,E)**. Schematic diagram of mouse *Dot1L* gene targeted by CRISPR/Cas9 to introduce a point mutation (Asn241Ala) that eliminates its methyltransferase activity (*Dot1L*-MM) **(F)**. Representative images of E10.5 WT **(G,I)** and *Dot1L*-MM **(H,J)** embryos show that the growth of *Dot1L*-MM embryos is comparable to that of wildtype embryos. While *Dot1L*-MM embryos displayed embryonic lethality, they had little to no anemia **(G–J)**, unlike the KO **(B–E)**.

**FIGURE 2:**
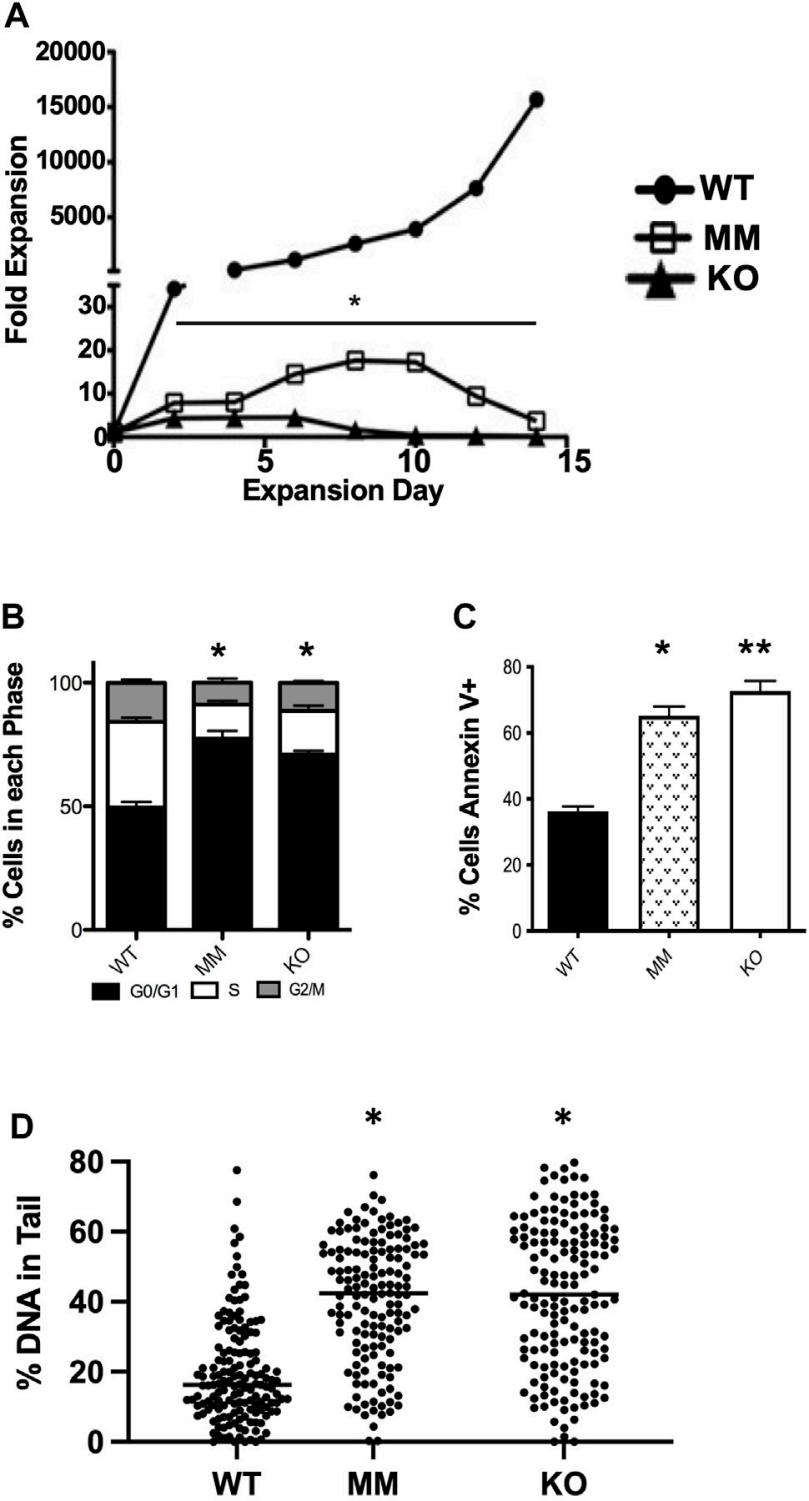
*Dot1L*-KO and *Dot1L*-MM ESREs display defective proliferation and survival. Cells isolated from E10.5 wildtype (WT), *Dot1L*-KO, and *Dot1L*-MM YSs were cultured in expansion media for ESREs. **(A)** Cell numbers were counted *via* trypan blue exclusion every 2 days for 14 days, and fold expansion was calculated relative to day 3 after isolation. On day 6–7 of counting, cells were labeled with propidium iodide or Annexin V and analyzed *via* flow cytometry for cell cycle analysis **(B)** and apoptosis **(C)**. Alkaline Comet assays were performed on day 6 ESREs to assess DNA damage. Both *Dot1L*-KO and *Dot1L*-MM ESREs displayed evidence of increased DNA damage compared to WT, as measured by increased comet “tail length”. Solid lines indicate median percent DNA in comet tails **(D)**. **(A,D)** are images of representative experiments, *n* ≥ 4. **(B,C)** are compiled data from 4 separate experiments. In these figures, bars represent mean ± SE. *indicates *p* ≤ 0.05. **indicates *p* ≤ 0.01.

We observed that *Dot1L*-KO embryos develop more slowly than WT embryos and suffer from lethal anemia (Feng et al., 2010) (Figures 1A–E). *Dot1L*-KO embryos die between embryonic day 11.5 (E11.5) and E13.5^1^. *Ex vivo* culture of HPCs from E10.5 *Dot1L*-KO YS showed that erythroid differentiation was severely affected compared to the myeloid lineage (Feng et al., 2010). We generated another mouse model that carries a point mutation (Asn241Ala) in endogenous *Dot1L*, rendering the catalytic domain inactive (Malcom et al., 2022) (Figures 1F–J). Although the *Dot1L*-methyl mutant (*Dot1L-*MM) embryos also died at mid-gestation, we observed remarkable differences in the hematopoietic phenotype between the *Dot1L*-KO and *Dot1L*-MM mice (Malcom et al., 2022) (Figures 1B–E,G–J); in particular, erythropoiesis was minimally affected in *Dot1L*-MM yolk sacs and embryos, suggesting that hematopoietic activity of DOT1L may not be limited to its MT domain. However, *ex vivo* culture of yolk sac cells demonstrated that formation of both myeloid and mixed colonies was dramatically reduced in either *Dot1L*-KO or *Dot1L*- MM^3^. Culture of *Dot1L*-KO HPCs showed decreased cell proliferation (Figure 2A) accumulation of cells in the G0/G1 stage of the cell cycle (Figure 2B), and a greater percentage of Dot1L-KO and Dot1L-MM HPCs in ESRE cultures that were Annexin V-positive (Feng et al., 2010) (Figure 2C). In addition, Alkaline Comet assays showed greater DNA damage in *Dot1L-*MM cells compared to wildtype cells. DNA damage in *Dot1L-*KO ESRE cells was also elevated in comparison to that observed in wildtype cells. (Figure 2D).

### DEGs in Dot1L-KO or Dot1L-MM ESRE Cells

**FIGURE 3:**
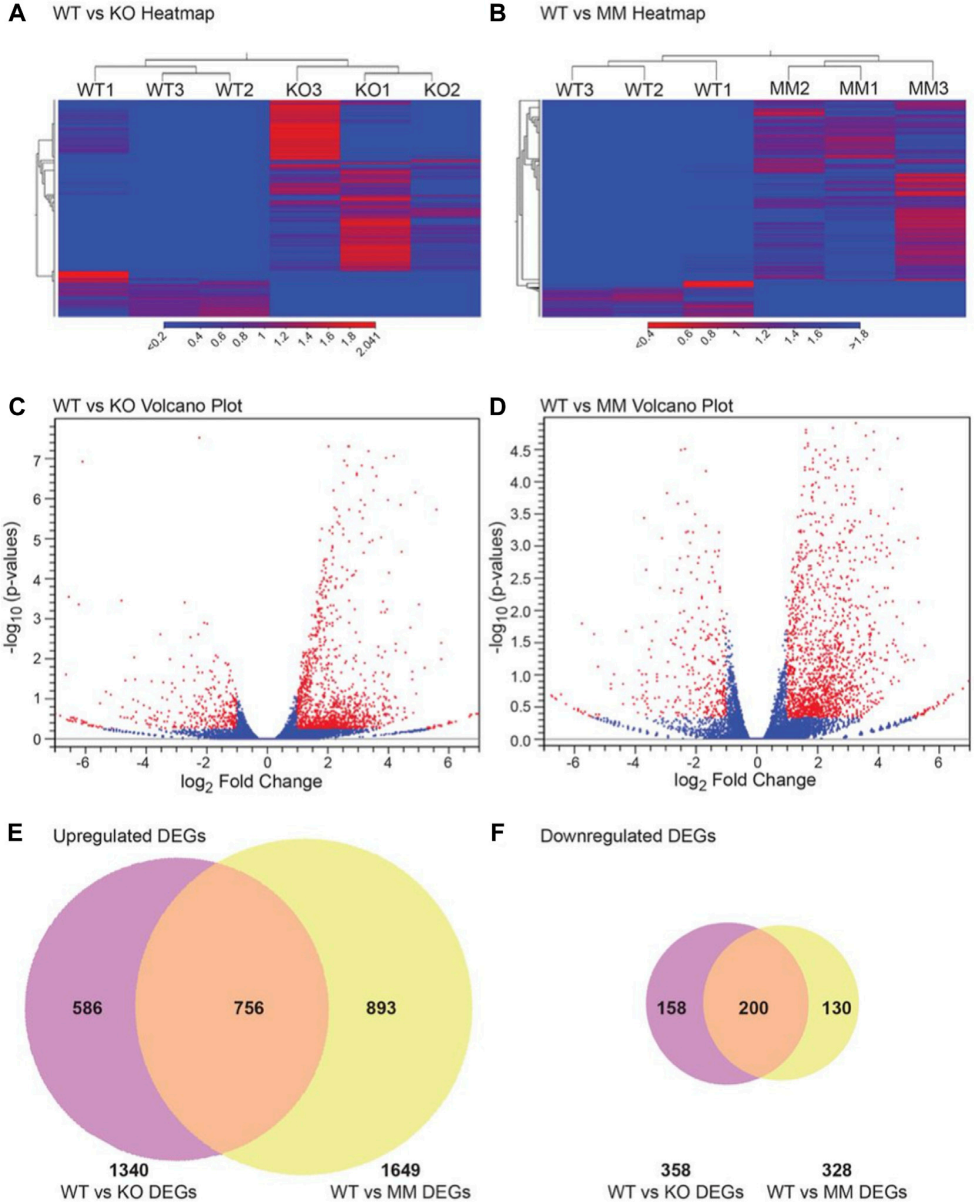
DEGs in *Dot1L*-KO and *Dot1L*-MM ESREs. Analyses of HPC transcriptomes were performed in wildtype (WT), *Dot1L*-KO, and *Dot1L*-MM ESREs on the 3rd day of *ex vivo* culture. RNA-seq libraries were prepared using 500 ng of total RNA extracted from pooled ESRE cells and sequencing was performed on an Illumina NovaSeq 6000 platform. RNA-seq data were analyzed using the CLC Genomics Workbench. All expressed genes were distributed according to TPM values. Hierarchical clustering was performed on differentially expressed genes (absolute fold change ≥2, *p value* ≤ 0.05) in WT versus *Dot1L*-KO groups **(A)**, and WT versus *Dot1L*-MM groups **(B).** The DEGs in the *Dot1L*-KO, and *Dot1L*-MM ESRE cells are presented in Volcano plots [**(C,D)** respectively], with red dots showing the differentially expressed genes (*n* = 3/genotype). Finally, Venn diagrams showing that majority of the DEGs (∼82%) in either *Dot1L*-KO or *Dot1L*- MM are upregulated **(E,F)**. Venn diagrams also showed that about 35–40% of the DEGs were unique to either *Dot1L*-mutant group **(E,F)**.

Transcriptome data sets were generated by sequencing mRNA purified from ESRE cultures using E10.5 wildtype, *Dot1L*-KO or *Dot1L*-MM YS cells. The raw data have been deposited to NCBI SRA under PRJNA666736. Analyzed data including the DEGs are shown in Figures 3A–F. Of the total 25,749 reference genes in the mm10 genome, 16,806 genes were detected in wildtype, 17,602 in *Dot1L*-KO and 17,761 in *Dot1L*-MM ESRE cells. Analyses of the detected genes for level of gene expression revealed that ∼40% had a very low abundance (<1 TPM), ∼20% had low abundance (1-5 TPM), ∼12% had moderate abundance (>5–10 TPM), ∼25% had high abundance (>10–100 TPM), and only ∼3% of the genes had a very high abundance (>100 TPM). Among these genes, 1790 were differentially expressed (absolute fold change ≥2, *p*-value ≤ 0.05) in *Dot1L*-KO, with 358 downregulated and 1408 upregulated. Similarly, 1977 genes were differentially expressed (absolute fold change ≥2, *p*-value ≤ 0.05) in *Dot1L*-MM cells, with 328∼17% downregulated and 1649∼83% upregulated. Remarkably, 812 (∼45%) of the DEGs (635 upregulated and 177 downregulated) were unique to *Dot1L*-KO and 1023 (∼52%) of the DEGs (893 upregulated and 130 downregulated) were unique to *Dot1L*-MM ESRE cells (Supplementary Table S4–S11). When gene expression was compared between the mutant groups, we identified a total of 359 differentially expressed genes (233 genes upregulated and 126 downregulated) (Supplementary Table S12, S13). The DEGs were also evident in the hierarchical clustering (Figures 3A,B) and Volcano plots (Figures 3C,D), which demonstrate that most of the DEGs were upregulated in *Dot1L*-KO or *Dot1L*-MM HPCs (Figures 3E,F).

### Gene Ontology (GO) Analysis of the DEGs

**FIGURE 4:**
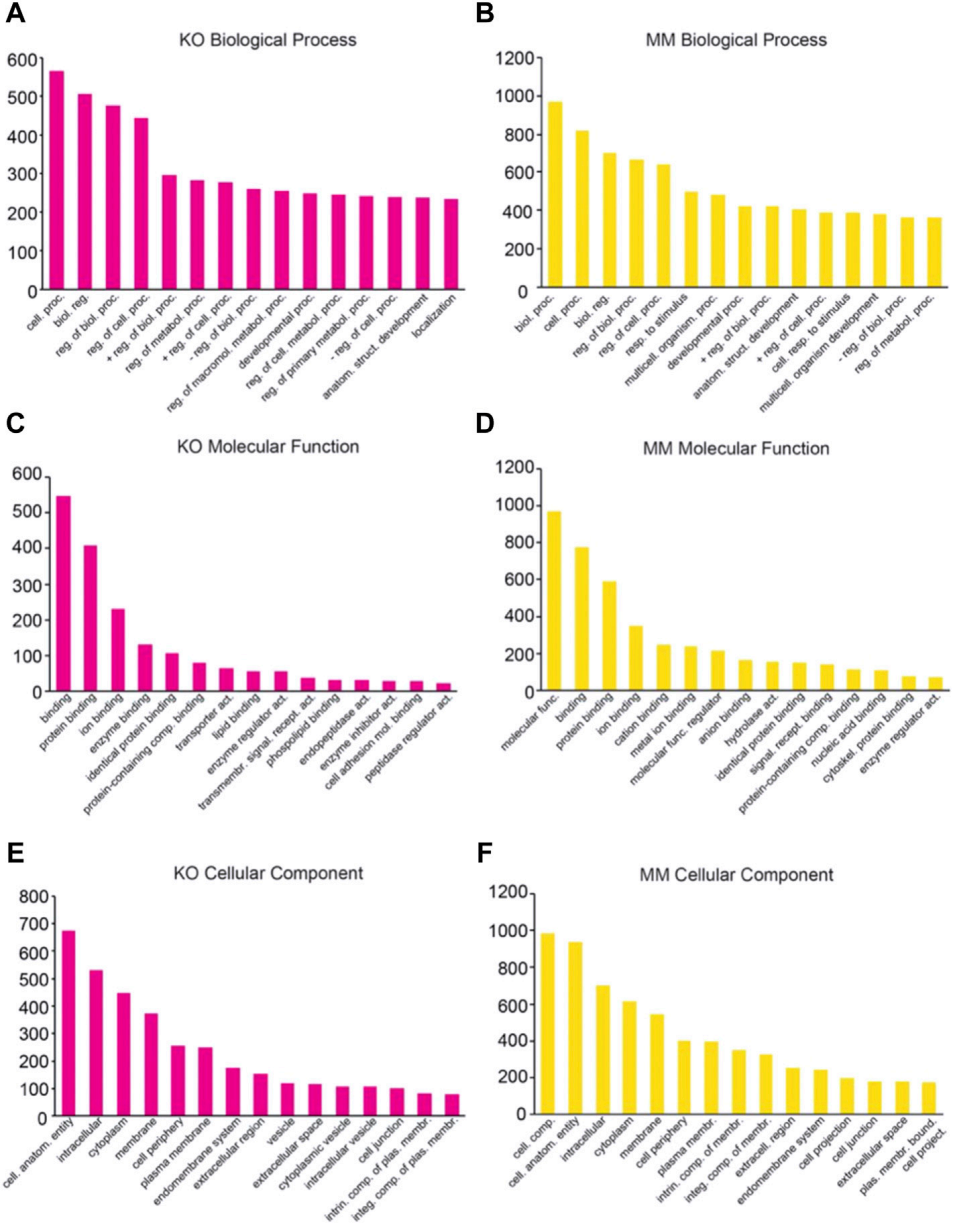
Classification of differentially expressed genes based on Gene Ontology. The differentially expressed genes in *Dot1L*-KO and *Dot1L*-MM ESREs were subjected to Panther Classification Analysis (http://pantherdb.org). Differentially expressed genes were classified. based on Biological Process **(A,B)**, Molecular Function **(C,D)** and Cellular Components **(E,F)**.

GO analysis classified the DEGs into three categories: Biological Process (Figures 4A,B), Molecular Function (Figures 4C,D) and Cellular Component (Figures 4E,F). GO analysis revealed that the majority of genes in the Biological Process group were involved in biological processes, cellular processes, or cell signaling (Figures 4A,B). The genes in Molecular Function were involved in binding, protein-protein interactions, catalytic activity, and molecular and transcriptional regulation (Figures 4C,D). The genes in Cellular Component were predominantly involved in cell parts, membranes, organelles, and protein-protein complexes (Figures 4E,F).

### Ingenuity Pathway Analysis of the DEGs

**FIGURE 5:**
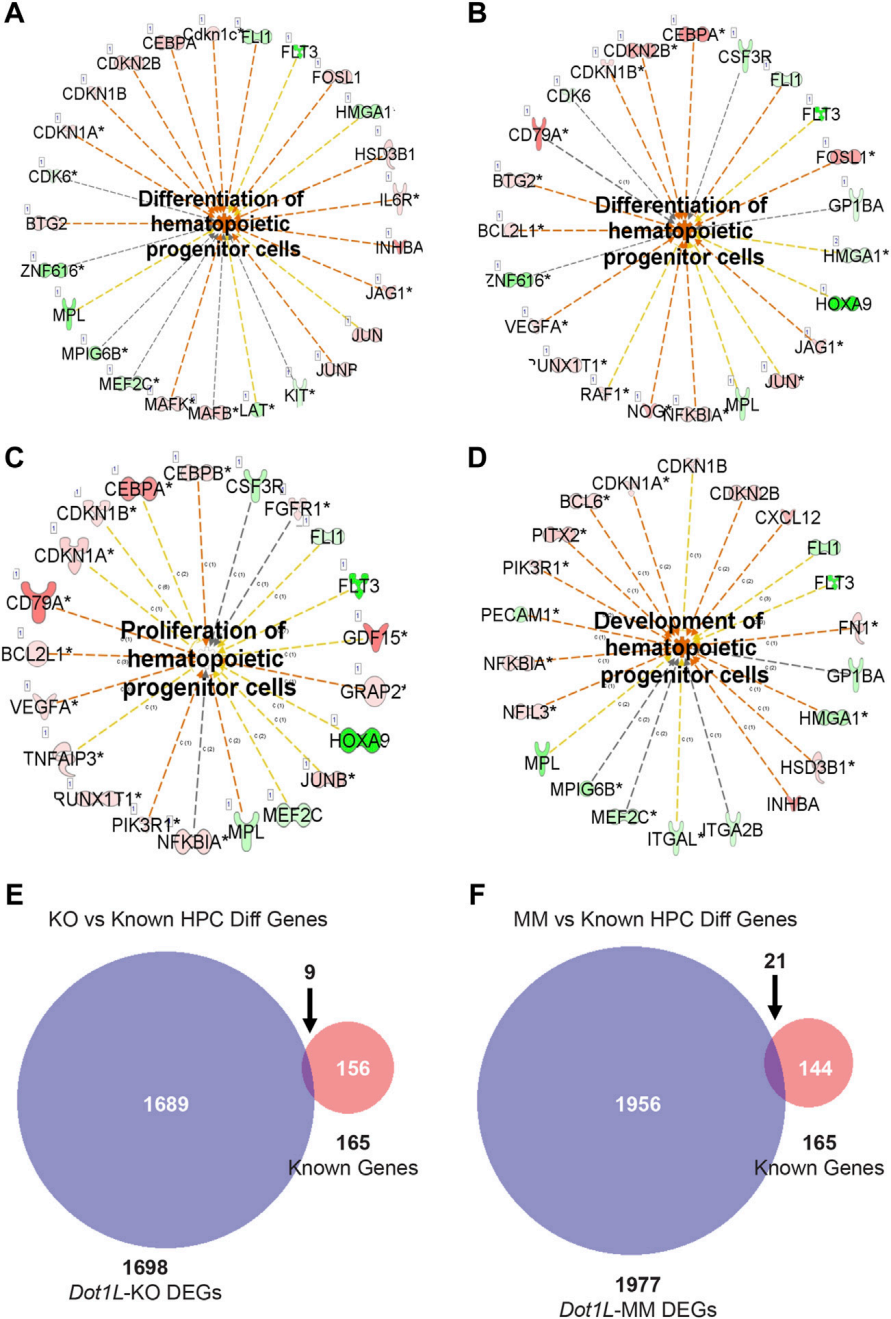
DEGs involved in proliferation and differentiation of HPCs. DEGs from RNA-seq data representing wildtype (WT) versus *Dot1L*-KO **(A,C)**, and WT versus *Dot1L*-MM **(B,D)** were subjected to IPA analysis and detected many genes crucial for proliferation and differentiation of HPCs. In addition, we curated a group of genes (*n* = 165) from the MGI database (www.informatics.jax.or) that are involved in differentiation of HPCs and compared those with the DEGs identified in our RNA-seq data. Most of the DEGs in *Dot1L*-KO or *Dot1L*-MM ESRE cells were found to be novel **(E,F)**. ‘c’ next to the relationship lines **(A-D)** stands for relationship types of ‘Causation/Leads to’ and the numbers next to ‘c’ stand for the number of reference findings supporting that link of relationship. The number before the gene name **(A-D)** inside the box represents number of splice variants. Asterisk (*) marks next to a gene name **(A-D)** indicate that multiple identifiers in the dataset file map to a single gene.

IPA of the DEGs in *Dot1L*-KO or *Dot1L*-MM HPCs in ESRE cultures revealed altered expression of genes related to regulation of hematopoiesis. Among the hematopoietic pathways involved, we were particularly interested in proliferation and differentiation of hematopoietic progenitor cells (Figures 5A–D). We observed that many of the genes related to differentiation of HPCs were affected in both *Dot1L*-KO or *Dot1L*-MM ESRE cells (Figures 5A,B). However, the genes related to proliferation of HPCs were primarily impacted in *Dot1L*-KO ESRE cells (Figure 5C), while the genes related to development of HPCs were affected in *Dot1L*-MM cells (Figure 5D). We have also included lists of the genes shown in Figure 5A–D (Supplementary Tables S14–S17). Interestingly, among the 165 known genes involved in differentiation of HPCs, only 9 were affected in *Dot1L*-KO cells, whereas 21 were affected in *Dot1L*-MM ESRE cells (Figures 5E,F).

### RT-qPCR Analyses Validated DEGs Involved in HPC Proliferation and Differentiation

**FIGURE 6:**
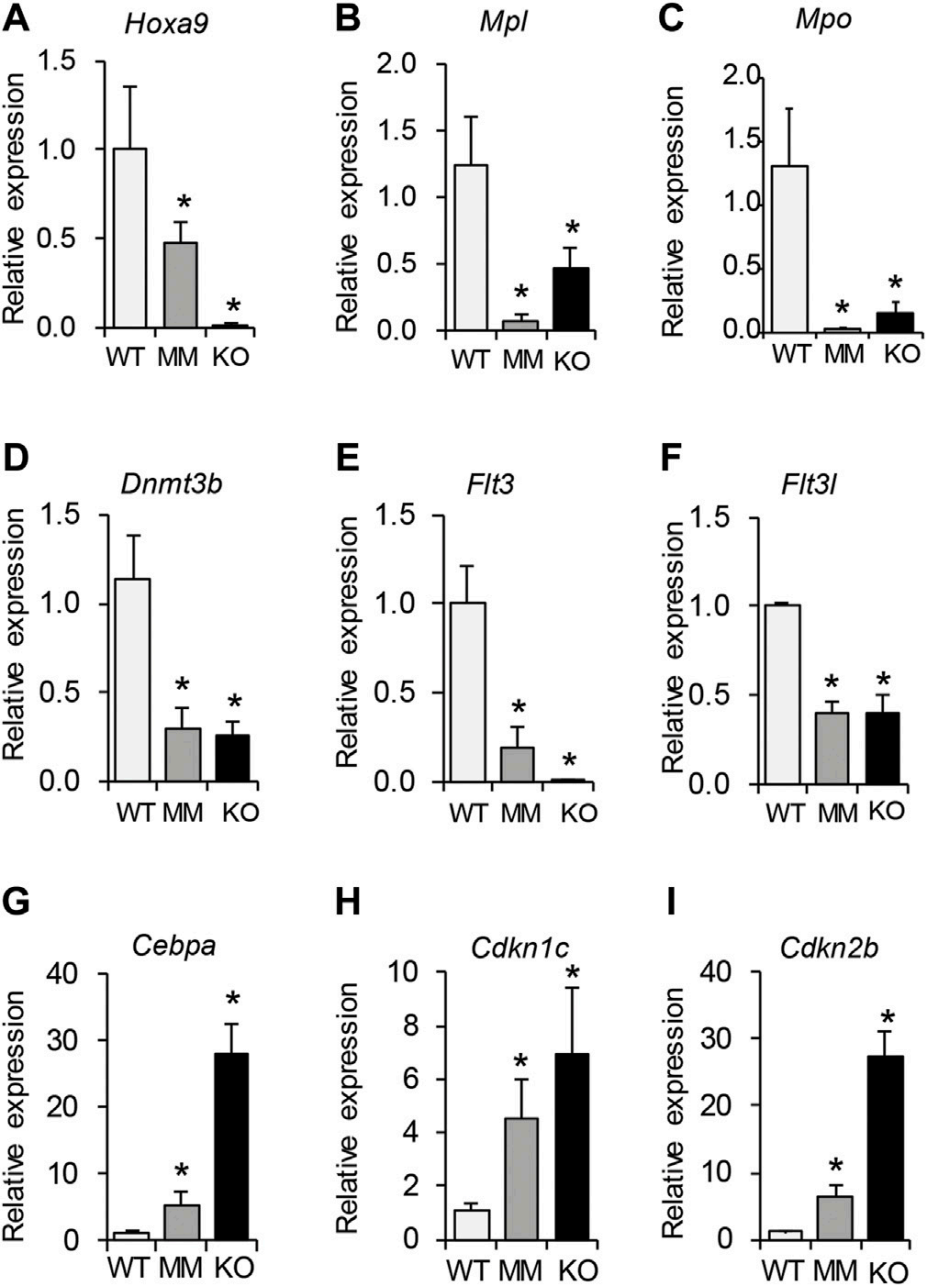
RT-qPCR validation of selected DEGs involved in proliferation and differentiation of HPCs. Differentially expressed genes that were identified to be involved in proliferation and differentiation of HPCs were further validated by RT-qPCR analyses. We observed that genes involved in proliferation of HPCs were significantly downregulated in both *Dot1L*-KO and *Dot1L*-MM ESRE cells **(A–F)**. In contrast, those involved in induction of HPC differentiation were markedly upregulated **(G–I)**. A marked increase in CDK inhibitors **(H,I)** correlates with the increased numbers of cells arrested in ^G^0^/G^1 phase of the cell cycle, as well as the increased percentage of the Dot1L-mutant ESRE cells undergoing apoptosis. Relative expression was determined using 3 individual samples and is expressed as mean ± SE. *indicates p < 0.05.

Differentially expressed genes that were identified as being involved in proliferation and differentiation of HPCs were validated by RT-qPCR analysis. We observed that while genes involved in proliferation of HPCs were significantly downregulated in both *Dot1L*-KO and *Dot1L*- MM ESRE cells (Figures 6A–F), those involved in induction of differentiation were markedly upregulated (Figures 6G–I). These included downregulation of *Hoxa9*, *Mpl*, *Mpo*, *Dnmt3b*, *Flt3*, and *Flt3l* as well as upregulation of the CDK inhibitors. Marked increase in CDK inhibitors (Figures 6H,I) can explain the accumulation of cells in the G_0_/G_1_ phase of the cell cycle and the increased proportion of the Dot1L-mutant ESRE cells undergoing apoptosis (Figure 2).

## Discussion

DOT1L is expressed at high levels in mouse HPCs (Supplementary Figure S1), suggesting a potential role for this chromatin organizer and transcriptional regulator in early blood development. This study analyzed the DEGs in both *Dot1l-*KO and *Dot1L-*MM mouse HPCs derived from E10.5 yolk sacs to examine early blood development. RNA-seq datasets were used to identify DOT1L-regulated genes in mouse HPCs and understand their potential role in early blood development.

We previously reported that hematopoietic transcription factor (TF) *Gata2* was significantly reduced in *Dot1L-*KO HPCs, whereas *Pu.1*, an erythropoiesis inhibiting TF was upregulated (Feng et al., 2010). We also observed that KIT-positive HPCs from *Dot1L*-KO yolk sacs expressed low levels of *Trpc6* (Feng et al., 20202020). Our RNA-seq data recapitulated the previous observations regarding expression of *Gata2, Pu.1 and Trpc6* (PRJNA666736). Gene ontology and IPA analyses indicated that DOT1L regulates genes responsible for cell signaling and protein-protein interactions. Our previous studies have demonstrated that either loss of DOT1L expression (Feng et al., 2010) or loss of its methyltransferase activity leads to G0/G1 cell cycle arrest and increased apoptosis. IPA analyses showed that the DEGs are linked to proliferation and differentiation of HPCs, which were further validated by RT-qPCR analyses. We observed that while genes involved in proliferation of HPCs were significantly downregulated, those involved in induction of differentiation were markedly upregulated (Figure 6).

It is possible that the observed phenotype of failed primitive erythroid development in *Dot1L*-KO, but not *Dot1L*-MM mice is consistent with the observation that genes related to proliferation are preferentially affected in the *Dot1L*-KO cells. During embryonic hematopoiesis in the yolk sac, the vast majority of proliferation is involved in early erythrocyte formation, necessary to supply the developing embryo with sufficient oxygen for proper growth and development. If proliferation of these developing cells is diminished by these alterations in gene expression, then this might result in the phenotypic differences we observed. We postulate that the changes in proliferation-related genes result in a preferential reduction in proliferation in *Dot1*-KO HPCs, *in vivo*, compared to *Dot1L*-MM HPCs.

A marked increase in CDK inhibitors positively correlates with the accumulation of cells in the G_0_/G_1_ phase of the cell cycle and an increased proportion of the *Dot1L*-mutant ESRE cells undergoing apoptosis. The gene expression profile also showed a positive correlation with mechanisms involved in increased DNA damage.

DOTL1L is responsible for methylation of H3K79 (Feng et al., 2002). Histone methylation is integral to permissive or repressive chromatin conformation, and regulation of gene expression (Miller and Grant, 2013). Expression of *Dot1/Dot1L* is conserved across species (Feng et al., 2002; van Leeuwen et al., 2002; Shanower et al., 2005; Janzen et al., 2006; Steger et al., 2008) and enrichment of H3K79 methyl marks is associated with actively transcribed chromatin regions (Feng et al., 2002; van Leeuwen et al., 2002; Shanower et al., 2005; Janzen et al., 2006; Steger et al., 2008). However, DOT1L has also been associated with repression of gene transcription (Wong et al., 2015).

In this study we found that more than 80% of DEGs are upregulated in the absence of either the entire DOT1L protein or the H3K79 methyltransferase activity, consistent with DOT1L activity being involved primarily in transcriptional *repression* of gene expression in HPC. Solely based on gene expression data, it is not possible to conclude that DOT1L acts as a transcriptional repressor. The effect can be indirect; instead of DOT1L acting directly to repress the expression of the genes, it is possible that one or more of the DOT1L-upregulated genes are responsible for inducing repression of the observed downregulated genes in ESRE cells. Another possibility is that DOT1L could be required for the expression of master transcriptional activators, which will also generate the same effects on gene expression. These and other possible scenarios will be examined in future studies.

Although 60% of DEGs were common to both *Dot1L*-mutant groups, the remaining 40% of DEGs were unique to either group (Figure 3). These data indicate that while a majority of HPC gene expression requires DOT1L methyltransferase activity, expression of a large number of DOT1L-regulated genes do not require the intrinsic methyltransferase activity of the protein. These data are consistent with a recent study in mouse embryonic stem cells (mESC) which demonstrated a potential H3K79 methylation-independent role of DOT1L in transcriptional elongation and cell fate determination (Cao et al., 2020).

Given the potential clinical relevance of targeted DOT1L activity in various cancer therapies, identification of the precise molecular mechanisms of the methyltransferase independent DOT1L function in the regulation of gene expression is a priority. Our data point to a previously undescribed role for DOT1L in regulating gene expression in a defined, murine developmental system. Identifying the components of this mode of gene regulation may lead to the discovery of novel DOT1L functions of this very interesting epigenetic regulator and may provide opportunity to identify new therapeutic targets.

## Data Availability

The datasets presented in this study can be found in online repositories. The names of the repository/repositories and accession number(s) can be found below: https://www.ncbi.nlm.nih.gov/, PRJNA666736.

## Supplementary Material

Supplementary Figure S1 KMT and KDM profiling analyses in *Dot1L*-KO and *Dot1L*-MM ESRE cells. The expression levels of a total of 27 KMTs and KDMs were analyzed using our RNA-seq data. The expression levels of *Dot1L* were the 4^th^ highest of these genes in wildtype (WT) ESRE cells **(A)**. In *Dot1L*-KO cells expression was very low **(B)** and high in *Dot1L*-MM cells **(C)**, comparable to that of WT HPCs.
