# Supplementary figures and images for: DOT1L Mediated Gene Repression in Extensively Self-Renewing Erythroblasts
Source: Front Genet. 2022 Mar 23;13:828086. doi: 10.3389/fgene.2022.828086 (PMC8984088; doi:10.3389/fgene.2022.828086)

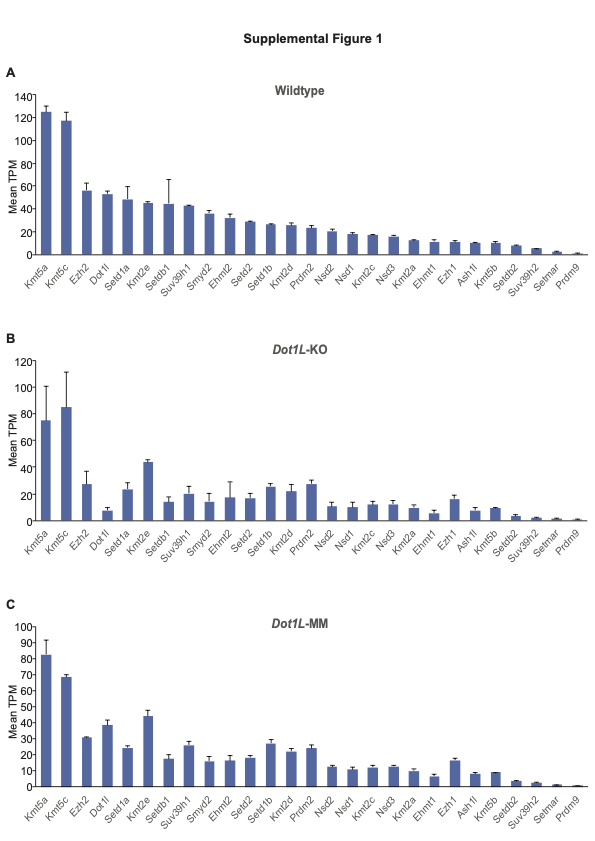

Supplement: Supplementary file 1 [file Image1.TIFF]

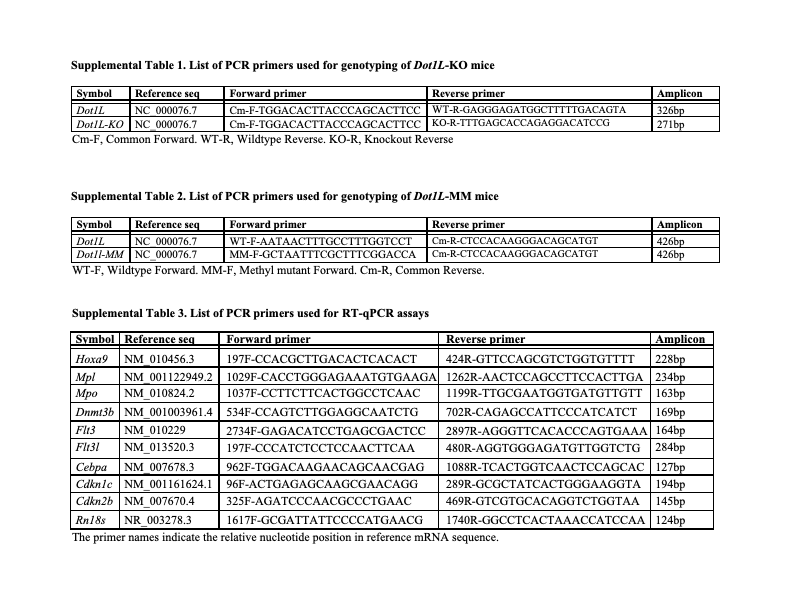

Supplement: Supplementary file 3 [file Image2.TIFF]
