# Supplementary material for: DOT1L Mediated Gene Repression in Extensively Self-Renewing Erythroblasts
Source: Front Genet. 2022 Mar 23;13:828086. doi: 10.3389/fgene.2022.828086 (PMC8984088; doi:10.3389/fgene.2022.828086)
Supplement: Supplementary file 2 [file DataSheet1.pdf]

Supplemental Table 1. List of PCR primers used for genotyping of *Dot1L*-KO mice

Symbol	Reference seq	Forward primer	Reverse primer	Amplicon
Dot1L	NC_000076.7	Cm-F-TGGACACTTACCCAGCACTTCC	WT-R-GAGGGAGATGGCTTTTGACAGTA	326bp
Dot1L-KO	NC_000076.7	Cm-F-TGGACACTTACCCAGCACTTCC	KO-R-TTTGAGCACCAGAGGACATCCG	271bp

Cm-F, Common Forward. WT-R, Wildtype Reverse. KO-R, Knockout Reverse

Supplemental Table 2. List of PCR primers used for genotyping of *Dot1L*-MM mice

Symbol	Reference seq	Forward primer	Reverse primer	Amplicon
Dot1L	NC_000076.7	WT-F-AATAACTTTGCCTTTGGTCCT	Cm-R-CTCCACAAGGGACAGCATGT	426bp
Dot1l-MM	NC_000076.7	MM-F-GCTAATTTCGCTTTCGGACCA	Cm-R-CTCCACAAGGGACAGCATGT	426bp

WT-F, Wildtype Forward. MM-F, Methyl mutant Forward. Cm-R, Common Reverse.

Supplemental Table 3. List of PCR primers used for RT-qPCR assays

Symbol	Reference seq	Forward primer	Reverse primer	Amplicon
Hoxa9	NM_010456.3	197F-CCACGCTTGACACTCACACT	424R-GTTCCAGCGTCTGGTGTTTT	228bp
Mpl	NM_001122949.2	1029F-CACCTGGGAGAAATGTGAAGA	1262R-AACTCCAGCCTTCCACTTGA	234bp
Mpo	NM_010824.2	1037F-CCTTCTTCACTGGCCTCAAC	1199R-TTGCGAATGGTGATGTTGTT	163bp
Dnmt3b	NM_001003961.4	534F-CCAGTCTTGGAGGCAATCTG	702R-CAGAGCCATTCCCATCATCT	169bp
Flt3	NM_010229	2734F-GAGACATCCTGAGCGACTCC	2897R-AGGGTTCACACCCAGTGAAA	164bp
Flt3l	NM_013520.3	197F-CCCATCTCCTCCAACTTCAA	480R-AGGTGGGAGATGTTGGTCTG	284bp
Cebpa	NM_007678.3	962F-TGGACAAGAACAGCAACGAG	1088R-TCACTGGTCAACTCCAGCAC	127bp
Cdkn1c	NM_001161624.1	96F-ACTGAGAGCAAGCGAACAGG	289R-GCGCTATCACTGGGAAGGTA	194bp
Cdkn2b	NM_007670.4	325F-AGATCCCAACGCCCTGAAC	469R-GTCGTGCACAGGTCTGGTAA	145bp
Rn18s	NR_003278.3	1617F-GCGATTATTCCCCATGAACG	1740R-GGCCTCACTAAACCATCCAA	124bp

The primer names indicate the relative nucleotide position in reference mRNA sequence.

Supplemental Table 4. Upregulated genes in *Dot1L*-KO ESRE cells in common with *Dot1L*-MM cells (n=773)

Name	Chromosome	Max group mean	Log_2 fold change	Fold change	P-value	ENSEMBL
St5	11	16.860	1.00	2.00	0.01	ENSMUSG00000020385
Gns	2	2.390	1.00	2.00	0.03	ENSMUSG00000027381
Pdk2	13	116.470	1.00	2.01	0.00	ENSMUSG00000044792
Nefh	10	55.970	1.01	2.01	0.00	ENSMUSG00000078440
Otub2	6	196.040	1.01	2.01	0.01	ENSMUSG00000025889
Plec	7	5.990	1.01	2.01	0.03	ENSMUSG00000031024
Ncf4	1	9.760	1.01	2.01	0.03	ENSMUSG00000026637
App	13	29.180	1.01	2.02	0.00	ENSMUSG00000021417
Trove2	5	26.570	1.01	2.02	0.01	ENSMUSG00000005514
Grina	5	56.280	1.01	2.02	0.01	ENSMUSG00000043510
Shld1	10	19.500	1.01	2.02	0.02	ENSMUSG00000034707
Pcmtd2	1	49.200	1.02	2.03	0.00	ENSMUSG00000033124
Ptms	8	8.570	1.02	2.03	0.01	ENSMUSG00000003575
Crybg1	4	8.060	1.03	2.04	0.00	ENSMUSG00000028803
Plekhm3	17	38.380	1.03	2.04	0.00	ENSMUSG00000039770
Trim56	11	21.290	1.03	2.04	0.00	ENSMUSG00000020894
Vim	11	45.030	1.04	2.05	0.00	ENSMUSG00000017119
44805	15	74.390	1.03	2.05	0.00	ENSMUSG00000022365
Atg4a	7	11.450	1.04	2.05	0.01	ENSMUSG00000030986
Txnip	11	134.900	1.04	2.05	0.01	ENSMUSG00000020485
Tnfrsf10b	11	9.090	1.04	2.05	0.01	ENSMUSG00000020932
Bicra	2	36.060	1.03	2.05	0.05	ENSMUSG00000017760
H1f0	9	36.910	1.05	2.06	0.00	ENSMUSG00000034908
Trim12c	11	11.380	1.04	2.06	0.00	ENSMUSG00000020142
Calcrl	10	129.100	1.04	2.06	0.00	ENSMUSG00000020235
Mfhas1	1	16.930	1.05	2.06	0.01	ENSMUSG00000016200
Fam53b	11	6.040	1.04	2.06	0.04	ENSMUSG00000038967
Serinc1	17	6.730	1.04	2.06	0.05	ENSMUSG00000024193
Naa80	1	22.790	1.05	2.07	0.00	ENSMUSG00000026027
Fam117a	X	49.180	1.05	2.07	0.00	ENSMUSG00000034160

Lgals3	7	21.230	1.05	2.07	0.00	ENSMUSG00000045282
Itga5	5	33.420	1.05	2.07	0.00	ENSMUSG00000066278
Ccng2	17	13.810	1.05	2.07	0.00	ENSMUSG00000047123
Slc43a2	11	45.300	1.06	2.08	0.00	ENSMUSG00000051510
Slc25a51	19	21.940	1.06	2.08	0.00	ENSMUSG00000024773
Akap2	7	3.880	1.06	2.08	0.05	ENSMUSG00000003752
Sgms1	15	45.500	1.06	2.09	0.00	ENSMUSG00000042351
Peg3	11	3.890	1.06	2.09	0.03	ENSMUSG00000020396
Loxl2	12	43.150	1.07	2.10	0.00	ENSMUSG00000021203
Stk17b	8	20.260	1.07	2.10	0.00	ENSMUSG00000026317
Cpeb4	11	16.450	1.07	2.10	0.00	ENSMUSG00000020715
Pim1	15	6.970	1.07	2.10	0.02	ENSMUSG00000022565
Kdm5b	15	18.210	1.07	2.10	0.02	ENSMUSG00000071715
Nudt18	3	6.920	1.07	2.10	0.03	ENSMUSG00000028042
Tns1	16	6.070	1.07	2.10	0.04	ENSMUSG00000022892
Cdr2	16	13.010	1.08	2.11	0.00	ENSMUSG00000022946
Tpm1	18	119.970	1.08	2.11	0.00	ENSMUSG00000032656
Map1lc3a	11	36.100	1.08	2.11	0.00	ENSMUSG00000003119
Flnc	15	92.810	1.07	2.11	0.00	ENSMUSG00000022452
Il6st	13	37.130	1.08	2.11	0.00	ENSMUSG00000058672
Cdc42ep4	5	55.460	1.08	2.11	0.01	ENSMUSG00000042744
Clk1	1	7.860	1.08	2.12	0.00	ENSMUSG00000018199
Anxa2	19	24.660	1.09	2.13	0.00	ENSMUSG00000010110
Fam129b	15	312.520	1.09	2.13	0.01	ENSMUSG00000022564
Gm7694	4	14.570	1.09	2.13	0.04	ENSMUSG00000050188
Timp2	2	231.520	1.10	2.14	0.00	ENSMUSG00000027078
Fbxo9	7	9.890	1.10	2.14	0.01	ENSMUSG00000025484
Gba2	13	23.230	1.10	2.14	0.03	ENSMUSG00000060147
Hecw1	2	7.850	1.10	2.14	0.04	ENSMUSG00000044991
Zfp473	4	23.720	1.10	2.15	0.00	ENSMUSG00000028559
Evi5	7	32.440	1.11	2.15	0.00	ENSMUSG00000039202
Ifnar2	7	22.520	1.10	2.15	0.00	ENSMUSG00000030847
Sfxn3	2	12.060	1.10	2.15	0.00	ENSMUSG00000027589

Gabarapl2	5	44.580	1.11	2.15	0.00	ENSMUSG00000028995
Fgfr1	2	14.820	1.10	2.15	0.01	ENSMUSG00000027230
Uap1l1	7	4.860	1.10	2.15	0.02	ENSMUSG00000030671
Pld3	16	66.290	1.11	2.16	0.00	ENSMUSG00000008393
Ezh1	12	11.760	1.11	2.16	0.00	ENSMUSG00000020653
Dedd2	7	7.000	1.11	2.16	0.00	ENSMUSG00000038520
Ctsd	7	5.960	1.11	2.16	0.00	ENSMUSG00000058761
Cstb	13	3.710	1.11	2.16	0.01	ENSMUSG00000021460
S100a11	6	53.160	1.11	2.16	0.02	ENSMUSG00000030122
Htatip2	10	2.150	1.11	2.16	0.03	ENSMUSG00000019866
Mpp1	6	3.970	1.11	2.16	0.04	ENSMUSG00000032652
Bcl2l1	16	16.210	1.11	2.16	0.05	ENSMUSG00000022500
Emp1	6	7.310	1.12	2.17	0.00	ENSMUSG00000030203
Pla2g16	9	9.520	1.12	2.17	0.00	ENSMUSG00000034563
Ulk1	1	1.860	1.12	2.17	0.02	ENSMUSG00000051344
Dennd4a	2	42.810	1.12	2.18	0.00	ENSMUSG00000027215
Fndc3b	5	18.550	1.12	2.18	0.00	ENSMUSG00000043279
Hdac5	7	15.470	1.13	2.18	0.00	ENSMUSG00000034825
Fbxo30	2	55.770	1.13	2.18	0.01	ENSMUSG00000026728
Calcoco1	4	3.490	1.12	2.18	0.04	ENSMUSG00000028927
Itgb7	7	358.590	1.13	2.19	0.01	ENSMUSG00000038539
Dnase1l1	17	8.110	1.13	2.19	0.01	ENSMUSG00000024074
Nlgn2	9	6.990	1.13	2.19	0.02	ENSMUSG00000037971
Zfp949	16	2.320	1.13	2.19	0.03	ENSMUSG00000071669
Ccdc92b	1	65.610	1.14	2.20	0.00	ENSMUSG00000026203
Tax1bp1	7	24.970	1.14	2.20	0.00	ENSMUSG00000011096
Cd68	7	25.680	1.14	2.20	0.00	ENSMUSG00000000486
Trib3	X	21.710	1.14	2.20	0.00	ENSMUSG00000079418
1810013L24Rik	18	98.160	1.14	2.20	0.00	ENSMUSG00000024404
Tmem67	3	42.310	1.14	2.20	0.00	ENSMUSG00000038393
Cdkn1c	19	22.860	1.13	2.20	0.03	ENSMUSG00000016495
Hmg20b	14	3.840	1.14	2.20	0.05	ENSMUSG00000022074
Rnh1	7	12.540	1.15	2.21	0.00	ENSMUSG00000070808

Slc30a1	15	58.010	1.15	2.21	0.00	ENSMUSG00000096210
Tinagl1	1	72.810	1.15	2.21	0.00	ENSMUSG00000049339
Zfp874b	17	23.010	1.14	2.21	0.00	ENSMUSG00000023827
Gstm5	19	12.800	1.15	2.21	0.01	ENSMUSG00000090673
Hbp1	X	36.480	1.15	2.21	0.01	ENSMUSG00000042903
Carmil1	17	31.320	1.15	2.22	0.00	ENSMUSG00000044477
Mical2	1	17.200	1.15	2.22	0.00	ENSMUSG00000034343
Rit1	11	18.360	1.15	2.22	0.00	ENSMUSG00000056938
Adipor1	15	2059.280	1.15	2.22	0.00	ENSMUSG00000022283
Ranbp10	1	13.720	1.16	2.23	0.00	ENSMUSG00000047443
Cdkn1b	15	36.840	1.16	2.23	0.03	ENSMUSG00000005125
Dnajb9	7	3.230	1.17	2.24	0.04	ENSMUSG00000057143
Mgll	1	87.910	1.17	2.25	0.00	ENSMUSG00000033159
Zkscan14	2	5.080	1.17	2.25	0.02	ENSMUSG00000059588
Abi3	8	2.250	1.17	2.25	0.04	ENSMUSG00000070056
Axl	7	17.050	1.18	2.26	0.00	ENSMUSG00000030956
Stk40	10	73.550	1.17	2.26	0.00	ENSMUSG00000019877
Plagl1	9	11.690	1.18	2.26	0.00	ENSMUSG00000032376
Ucp2	7	36.480	1.18	2.26	0.00	ENSMUSG00000078566
Xpo7	5	47.060	1.18	2.26	0.00	ENSMUSG00000025825
Glrx	9	15.430	1.18	2.26	0.00	ENSMUSG00000079334
Mmp2	1	9.290	1.18	2.26	0.00	ENSMUSG00000040848
Lpin1	10	51.020	1.18	2.26	0.03	ENSMUSG00000025351
Crebrf	X	6.150	1.19	2.27	0.03	ENSMUSG00000031266
Mturn	11	107.840	1.19	2.28	0.00	ENSMUSG00000038893
Specc1	19	12.060	1.19	2.28	0.00	ENSMUSG00000025178
Ckap4	8	34.850	1.19	2.28	0.00	ENSMUSG00000053560
Rusc2	1	5.410	1.19	2.28	0.01	ENSMUSG00000026698
Btg1	14	22.120	1.19	2.28	0.01	ENSMUSG00000050335
Tent5c	9	2.480	1.19	2.28	0.02	ENSMUSG00000041268
Ndrg4	6	5.740	1.19	2.28	0.02	ENSMUSG00000039958
Ahnak	15	7.720	1.19	2.28	0.05	ENSMUSG00000000555
Anxa1	5	23.320	1.20	2.29	0.00	ENSMUSG00000029385

Tnfrsf12a	4	11.360	1.19	2.29	0.01	ENSMUSG00000045268
Kirrel	X	11.330	1.20	2.29	0.01	ENSMUSG00000031161
Suox	11	12.700	1.20	2.30	0.00	ENSMUSG00000038178
Lhfpl2	11	5.560	1.20	2.30	0.02	ENSMUSG00000018474
Mettl27	X	9.210	1.20	2.30	0.05	ENSMUSG00000031149
Dcaf12	13	3.870	1.20	2.30	0.05	ENSMUSG00000071291
Smtn	2	32.650	1.21	2.31	0.00	ENSMUSG00000090213
Rsrp1	4	74.280	1.21	2.31	0.00	ENSMUSG00000045973
Micall1	4	4.710	1.21	2.31	0.05	ENSMUSG00000038729
Hspg2	19	15.290	1.22	2.32	0.00	ENSMUSG00000040451
Sertad3	11	12.860	1.22	2.32	0.00	ENSMUSG00000020859
Vsir	17	14.520	1.22	2.32	0.00	ENSMUSG00000024197
Fads3	7	35.860	1.22	2.32	0.01	ENSMUSG00000002265
Prnp	11	126.810	1.22	2.33	0.00	ENSMUSG00000073063
S100a6	14	10.820	1.22	2.33	0.04	ENSMUSG00000034205
Rras	1	22.040	1.22	2.34	0.00	ENSMUSG00000026094
Fstl1	7	28.050	1.23	2.34	0.00	ENSMUSG00000066406
Bmp1	11	125.680	1.23	2.34	0.00	ENSMUSG00000020300
Stag3	17	354.170	1.23	2.34	0.00	ENSMUSG00000024014
Gstm1	7	6.050	1.23	2.34	0.02	ENSMUSG00000045868
Klf6	9	20.870	1.23	2.34	0.02	ENSMUSG00000025647
Fhl1	13	21.280	1.23	2.35	0.00	ENSMUSG00000074794
Alas2	8	51.590	1.23	2.35	0.00	ENSMUSG00000031812
Ttc12	9	7.210	1.23	2.35	0.00	ENSMUSG00000032449
Plpp3	3	18.360	1.23	2.35	0.00	ENSMUSG00000027845
Nid1	1	7.430	1.24	2.36	0.00	ENSMUSG00000042207
Sertad1	11	18.570	1.24	2.36	0.00	ENSMUSG00000020400
Nos1ap	14	4.920	1.24	2.36	0.01	ENSMUSG00000045211
Ltbp2	5	7.890	1.24	2.36	0.02	ENSMUSG00000008090
Btn1a1	1	6.140	1.24	2.37	0.00	ENSMUSG00000055322
Ets1	16	12.610	1.25	2.38	0.00	ENSMUSG00000022641
Fhdc1	7	194.160	1.25	2.38	0.00	ENSMUSG00000030878
Mafk	9	93.360	1.26	2.39	0.00	ENSMUSG00000032366

Tbcel	11	71.930	1.25	2.39	0.00	ENSMUSG00000034354
Pik3r1	7	116.580	1.26	2.39	0.00	ENSMUSG00000003873
Letm2	12	11.110	1.26	2.39	0.00	ENSMUSG00000021285
Tnks1bp1	2	23.990	1.26	2.39	0.01	ENSMUSG00000027602
Slc6a8	6	3.480	1.26	2.39	0.03	ENSMUSG00000068699
Tmem131l	2	60.600	1.26	2.40	0.00	ENSMUSG00000023572
Ptgs2	11	19.760	1.27	2.40	0.01	ENSMUSG00000045176
Naprt	13	3.200	1.26	2.40	0.02	ENSMUSG00000021756
Tnfaip3	11	12.140	1.27	2.41	0.00	ENSMUSG00000041598
Ube2h	1	99.480	1.27	2.41	0.00	ENSMUSG00000026034
Prr15l	11	339.740	1.27	2.41	0.00	ENSMUSG00000018677
Hspb1	9	60.240	1.27	2.41	0.01	ENSMUSG00000032231
Igf2	2	15.110	1.27	2.41	0.02	ENSMUSG00000026796
Fermt2	1	3.510	1.27	2.41	0.02	ENSMUSG00000102752
Khnyn	11	4.710	1.27	2.41	0.04	ENSMUSG00000017466
Cd36	8	4.740	1.27	2.41	0.05	ENSMUSG00000031803
Abcc3	7	106.970	1.27	2.42	0.00	ENSMUSG00000051811
Klhl25	9	49.760	1.28	2.42	0.00	ENSMUSG00000001366
Mbd6	19	7.590	1.27	2.42	0.00	ENSMUSG00000024769
Hbb-bs	12	26.960	1.28	2.43	0.00	ENSMUSG00000021127
Cavin1	4	15.760	1.28	2.43	0.00	ENSMUSG00000028467
Fam129a	17	7.860	1.28	2.43	0.00	ENSMUSG00000056121
Dnajb4	6	6.340	1.28	2.43	0.03	ENSMUSG00000030103
Pnrc1	16	33.320	1.29	2.44	0.00	ENSMUSG00000039568
Mxd1	13	1.010	1.29	2.44	0.03	ENSMUSG00000021301
Myadm	7	1.000	1.29	2.44	0.04	ENSMUSG00000048012
Agrn	5	16.630	1.29	2.45	0.00	ENSMUSG00000011831
Ablim1	16	30.860	1.29	2.45	0.00	ENSMUSG00000022971
Ehd2	19	4.080	1.29	2.45	0.03	ENSMUSG00000025212
Col1a2	13	2.620	1.29	2.45	0.03	ENSMUSG00000041773
Smad1	12	6.970	1.30	2.45	0.04	ENSMUSG00000021250
Farp1	8	77.320	1.30	2.46	0.00	ENSMUSG00000031950
Ube2b	17	74.040	1.30	2.46	0.00	ENSMUSG00000061232

Il4ra	8	6.470	1.30	2.46	0.01	ENSMUSG00000031565
Zrsr1	2	11.000	1.30	2.46	0.03	ENSMUSG00000026956
Hba-a2	17	12.350	1.30	2.46	0.04	ENSMUSG00000007038
Per1	7	8.780	1.30	2.46	0.05	ENSMUSG00000019734
Col5a1	7	36.600	1.30	2.47	0.00	ENSMUSG00000003363
Aqp9	11	9.400	1.30	2.47	0.00	ENSMUSG00000006920
Fbxo32	8	18.340	1.30	2.47	0.01	ENSMUSG00000036840
Tagln	6	111.090	1.31	2.48	0.00	ENSMUSG00000030342
Rhoc	2	4.020	1.31	2.48	0.05	ENSMUSG00000027221
Wdr45	3	10.920	1.32	2.49	0.00	ENSMUSG00000027663
Ifngr2	7	75.120	1.31	2.49	0.00	ENSMUSG00000054499
Anxa3	7	390.030	1.32	2.49	0.00	ENSMUSG00000007891
Nrbp2	14	5.110	1.32	2.49	0.01	ENSMUSG00000021770
Dstn	10	84.120	1.32	2.49	0.01	ENSMUSG00000005054
Gstp3	2	5.390	1.32	2.49	0.01	ENSMUSG00000045319
Srxn1	3	65.340	1.32	2.49	0.03	ENSMUSG00000027907
Pink1	19	27.470	1.32	2.50	0.00	ENSMUSG00000047423
Sh3pxd2a	11	8.650	1.32	2.50	0.01	ENSMUSG00000018507
Wnt2	7	56.910	1.33	2.51	0.00	ENSMUSG00000039745
Abtb1	6	27.760	1.33	2.51	0.00	ENSMUSG00000029534
Arhgef10	X	130.890	1.33	2.51	0.00	ENSMUSG00000031402
Atg14	2	118.130	1.33	2.51	0.00	ENSMUSG00000007659
Cpeb2	3	61.250	1.33	2.51	0.00	ENSMUSG00000001865
Cyp1b1	6	9.240	1.33	2.51	0.04	ENSMUSG00000030208
Gpcpd1	19	7.550	1.34	2.52	0.00	ENSMUSG00000060675
Fn3k	5	106.250	1.33	2.52	0.00	ENSMUSG00000029512
Col11a1	9	113.160	1.34	2.52	0.00	ENSMUSG00000053641
Smtnl1	11	6.540	1.33	2.52	0.03	ENSMUSG00000023764
Slc16a10	3	4.470	1.33	2.52	0.05	ENSMUSG00000039286
Csf2rb	11	20.200	1.34	2.53	0.00	ENSMUSG00000008855
Isg15	10	46.970	1.34	2.53	0.00	ENSMUSG00000047648
Mxi1	15	38.850	1.34	2.53	0.00	ENSMUSG00000023055
Hist2h2be	15	15.730	1.34	2.53	0.00	ENSMUSG00000001281

Pmaip1	16	8.390	1.34	2.53	0.00	ENSMUSG00000032932
Acta2	X	17.540	1.34	2.53	0.00	ENSMUSG00000019088
Fbxl22	11	3.060	1.34	2.53	0.01	ENSMUSG00000051790
Dusp1	9	8.340	1.35	2.54	0.00	ENSMUSG00000032425
Snx21	18	8.200	1.34	2.54	0.01	ENSMUSG00000038418
Fars2	18	2.060	1.34	2.54	0.03	ENSMUSG00000024589
Dsc2	9	10.580	1.35	2.54	0.05	ENSMUSG00000032418
Zswim4	11	49.660	1.35	2.55	0.00	ENSMUSG00000069814
Btg2	11	6.430	1.35	2.55	0.01	ENSMUSG00000020888
Tmem98	14	0.580	1.35	2.55	0.04	ENSMUSG00000021983
Sowaha	1	21.270	1.35	2.56	0.00	ENSMUSG00000056708
Pdzk1ip1	13	20.580	1.36	2.56	0.01	ENSMUSG00000021453
Wwtr1	6	120.880	1.37	2.58	0.00	ENSMUSG00000004535
Tmem43	10	813.520	1.36	2.58	0.00	ENSMUSG00000035242
Rnasel	7	14.260	1.37	2.58	0.00	ENSMUSG00000002409
Fam214b	11	27.150	1.37	2.58	0.03	ENSMUSG00000018774
Lcp1	2	153.630	1.37	2.59	0.00	ENSMUSG00000032715
Cma1	16	32.390	1.38	2.60	0.00	ENSMUSG00000022507
Lpp	4	2.040	1.38	2.60	0.02	ENSMUSG00000049488
Tcp11l2	9	9.550	1.38	2.60	0.05	ENSMUSG00000032548
Col5a2	7	49.370	1.38	2.61	0.02	ENSMUSG00000037664
Mboat2	8	6.290	1.39	2.61	0.02	ENSMUSG00000052566
Camk2d	10	41.230	1.39	2.62	0.00	ENSMUSG00000020232
Slc48a1	7	43.500	1.39	2.62	0.00	ENSMUSG00000038650
Phospho1	1	20.650	1.39	2.62	0.00	ENSMUSG00000037434
Fam213a	4	58.100	1.39	2.62	0.00	ENSMUSG00000028776
Tob1	17	15.210	1.39	2.62	0.00	ENSMUSG00000023951
Myof	13	8.690	1.39	2.62	0.01	ENSMUSG00000059839
Gtpbp2	14	36.620	1.40	2.63	0.00	ENSMUSG00000022125
Inka2	3	72.150	1.40	2.63	0.00	ENSMUSG00000004032
Rnf128	12	35.720	1.39	2.63	0.00	ENSMUSG00000002996
Hook1	13	2.320	1.39	2.63	0.01	ENSMUSG00000021338
Gadd45b	5	12.900	1.39	2.63	0.03	ENSMUSG00000029470

Lima1	7	1.430	1.40	2.63	0.05	ENSMUSG00000038244
Trim58	3	24.360	1.40	2.64	0.00	ENSMUSG00000028057
Gm4070	1	232.910	1.40	2.64	0.00	ENSMUSG00000026457
Prkab2	8	263.300	1.40	2.64	0.00	ENSMUSG00000037415
Celf4	2	6.790	1.40	2.64	0.02	ENSMUSG00000027087
Smpdl3a	10	5.140	1.40	2.64	0.03	ENSMUSG00000001211
Slfn14	6	36.120	1.41	2.65	0.00	ENSMUSG00000003031
Col1a1	12	15.660	1.41	2.65	0.00	ENSMUSG00000014905
Kank2	6	7.850	1.41	2.65	0.00	ENSMUSG00000033174
Cbfa2t3	5	5.750	1.41	2.65	0.01	ENSMUSG00000029627
Tmcc2	11	2.400	1.41	2.65	0.02	ENSMUSG00000018381
Phf23	7	5.160	1.41	2.65	0.04	ENSMUSG00000002602
Fbn1	13	43.680	1.40	2.65	0.04	ENSMUSG00000021477
Mocos	4	51.050	1.41	2.66	0.00	ENSMUSG00000042608
Ddit3_1	15	82.340	1.41	2.66	0.00	ENSMUSG00000022453
Ppp1r15a	10	6.150	1.41	2.66	0.03	ENSMUSG00000019817
Pak6	7	707.110	1.41	2.67	0.00	ENSMUSG00000033685
Mettl7a1	14	73.600	1.42	2.67	0.00	ENSMUSG00000022100
Ubb	13	95.950	1.42	2.67	0.00	ENSMUSG00000021591
Epha4	8	11.220	1.42	2.67	0.01	ENSMUSG00000031740
Arhgef37	12	8.670	1.42	2.68	0.00	ENSMUSG00000020593
Atg16l2	17	8.030	1.42	2.68	0.00	ENSMUSG00000048249
Nfkb2	11	36.340	1.42	2.68	0.00	ENSMUSG00000000278
Actg2	6	1.070	1.42	2.68	0.05	ENSMUSG00000038065
Rab30	7	96.710	1.43	2.69	0.00	ENSMUSG00000037706
Sptbn4	11	94.050	1.43	2.69	0.00	ENSMUSG00000042331
Prokr1	10	13.050	1.43	2.69	0.02	ENSMUSG00000046841
Resf1	17	3.960	1.43	2.69	0.02	ENSMUSG00000068036
Fam213b	4	2.160	1.43	2.69	0.05	ENSMUSG00000035969
Vwa5a	10	22.800	1.43	2.70	0.00	ENSMUSG00000036478
Stard10	3	422.300	1.43	2.70	0.00	ENSMUSG00000044468
Fsd1l	1	303.540	1.43	2.70	0.00	ENSMUSG00000040713
Acot2	5	3.110	1.43	2.70	0.03	ENSMUSG00000038295

Klhl21	8	2.020	1.43	2.70	0.04	ENSMUSG00000036564
Ubap1	4	185.770	1.44	2.71	0.00	ENSMUSG00000028906
Itga3	19	9.120	1.44	2.71	0.00	ENSMUSG00000069833
Camk2a	19	17.270	1.44	2.71	0.02	ENSMUSG00000024659
Reln	17	16.550	1.44	2.71	0.05	ENSMUSG00000023905
Ddr2	3	1.190	1.45	2.72	0.04	ENSMUSG00000041734
Maged1	1	2.310	1.44	2.72	0.05	ENSMUSG00000047180
Atf3	10	3.010	1.44	2.72	0.05	ENSMUSG00000049858
Emp2	11	327.030	1.45	2.73	0.00	ENSMUSG00000018567
Padi3	14	17.240	1.45	2.73	0.00	ENSMUSG00000022180
Pard3	7	12.150	1.45	2.73	0.02	ENSMUSG00000062300
Btnl10	13	1.410	1.45	2.73	0.04	ENSMUSG00000045312
Abca1	5	25.030	1.46	2.74	0.00	ENSMUSG00000040557
Bsdc1	2	121.310	1.46	2.74	0.00	ENSMUSG00000056501
Hyal1	12	10.330	1.45	2.74	0.04	ENSMUSG00000057963
Ube2o	11	0.550	1.45	2.74	0.05	ENSMUSG00000020275
Trp53inp2	4	168.080	1.46	2.75	0.00	ENSMUSG00000028436
Spsb1	11	6.450	1.46	2.75	0.00	ENSMUSG00000020439
Ypel4	17	4.470	1.46	2.75	0.03	ENSMUSG00000037098
H2-T22	4	214.450	1.46	2.76	0.00	ENSMUSG00000037266
Gpr157	19	3848.930	1.47	2.76	0.00	ENSMUSG00000024661
Tg	15	4.840	1.47	2.76	0.00	ENSMUSG00000033039
Ubtd1	1	175.650	1.47	2.77	0.00	ENSMUSG00000026626
Kctd11	4	7.320	1.47	2.77	0.03	ENSMUSG00000028763
Kdm6b	7	68.770	1.47	2.78	0.00	ENSMUSG00000055200
Gadd45a	12	42.290	1.47	2.78	0.00	ENSMUSG00000021133
Ccdc80	10	3.600	1.48	2.78	0.00	ENSMUSG00000020101
Ccdc88a	19	5.960	1.47	2.78	0.02	ENSMUSG00000024664
Mfsd12	2	51.310	1.48	2.79	0.00	ENSMUSG00000079037
Ephx1	2	26.710	1.48	2.79	0.00	ENSMUSG00000035399
Abhd6	3	41.350	1.48	2.79	0.03	ENSMUSG00000001025
Cyp2b10	7	17.350	1.49	2.80	0.01	ENSMUSG00000038387
Postn	16	9.310	1.48	2.80	0.02	ENSMUSG00000022816

Fam160a1	14	4.140	1.49	2.80	0.04	ENSMUSG00000022098
Trafd1	11	72145.140	1.49	2.81	0.00	ENSMUSG00000069919
Sesn2	5	3.340	1.49	2.81	0.00	ENSMUSG00000036928
Csrnp2	7	7.150	1.49	2.82	0.01	ENSMUSG00000041775
Ctsf	7	8.490	1.50	2.82	0.01	ENSMUSG00000030762
Gstt2	3	25.110	1.50	2.82	0.03	ENSMUSG00000058135
Crem	13	41.490	1.50	2.83	0.00	ENSMUSG00000000078
Angptl2	15	1.530	1.50	2.83	0.04	ENSMUSG00000016624
Slc25a33	X	20.170	1.51	2.84	0.00	ENSMUSG00000023092
Plvap	X	1643.240	1.51	2.85	0.00	ENSMUSG00000025270
Abhd4	9	4.070	1.51	2.85	0.00	ENSMUSG00000040219
Hist1h1c	4	1.400	1.51	2.85	0.05	ENSMUSG00000028517
Apoe	13	13.200	1.51	2.85	0.05	ENSMUSG00000005397
Rhbdf2	7	66.460	1.51	2.86	0.00	ENSMUSG00000008384
Tmem106a	1	3.810	1.52	2.86	0.01	ENSMUSG00000038473
Fbxl3	12	1.740	1.51	2.86	0.01	ENSMUSG00000002020
Hist1h1e	2	0.780	1.52	2.86	0.05	ENSMUSG00000026923
Masp1	8	1.250	1.51	2.86	0.05	ENSMUSG00000031847
Aif1l	7	3.890	1.52	2.86	0.05	ENSMUSG00000054065
Zfp945	13	10.940	1.52	2.87	0.00	ENSMUSG00000000706
Hs3st3a1	7	8.130	1.52	2.87	0.00	ENSMUSG00000040734
Phlda1	9	1.640	1.52	2.87	0.04	ENSMUSG00000032035
Adrb2	3	117.640	1.53	2.89	0.00	ENSMUSG00000041842
Tmem88	2	46.100	1.53	2.89	0.03	ENSMUSG00000026879
Npr2	8	7.190	1.53	2.89	0.05	ENSMUSG00000031586
Ccrl2	5	83.900	1.54	2.90	0.00	ENSMUSG00000018143
Gch1	9	81.180	1.54	2.90	0.00	ENSMUSG00000037287
Ankrd33b	11	4.200	1.53	2.90	0.03	ENSMUSG00000034614
Mad2l1bp	13	21.990	1.54	2.91	0.00	ENSMUSG00000041417
Tgif1	8	2.550	1.54	2.91	0.01	ENSMUSG00000037363
Stk32a	3	1.950	1.55	2.92	0.04	ENSMUSG00000102805
Cpt1c	9	9.540	1.54	2.92	0.05	ENSMUSG00000031995
Gatm	2	2.760	1.55	2.93	0.02	ENSMUSG00000033955

Mkrn1	X	7.270	1.55	2.93	0.03	ENSMUSG00000019558
Avpi1	3	75.440	1.56	2.94	0.00	ENSMUSG00000033767
Mef2d	1	1.950	1.55	2.94	0.02	ENSMUSG00000032487
Dcn	15	2.920	1.56	2.94	0.03	ENSMUSG00000022574
Mustn1	2	2.560	1.56	2.94	0.03	ENSMUSG00000061186
Hopx	10	2.320	1.56	2.95	0.02	ENSMUSG00000019850
Rbm11	6	143.690	1.57	2.96	0.00	ENSMUSG00000039159
Mfsd4b1	11	1.270	1.56	2.96	0.03	ENSMUSG00000047040
Nodal	10	5.170	1.57	2.97	0.02	ENSMUSG00000020023
Tfap2c	5	57.560	1.57	2.97	0.03	ENSMUSG00000004951
H2-D1	11	2.680	1.57	2.98	0.02	ENSMUSG00000052949
Tbx6	5	1.690	1.58	2.99	0.04	ENSMUSG00000037979
Jund	7	110.490	1.58	2.99	0.04	ENSMUSG00000048583
Patl2	14	5.280	1.58	2.99	0.04	ENSMUSG00000037712
Plk3	14	28.780	1.58	3.00	0.00	ENSMUSG00000047153
Arid5b	5	2.100	1.58	3.00	0.02	ENSMUSG00000002944
Sema6a	11	1.040	1.58	3.00	0.03	ENSMUSG00000020865
Gm13889	9	2.850	1.59	3.01	0.01	ENSMUSG00000032470
Dnm1	7	17.010	1.60	3.02	0.00	ENSMUSG00000055652
Samd11	10	75.600	1.60	3.02	0.00	ENSMUSG00000025409
Dapk2	7	115630.810	1.60	3.02	0.00	ENSMUSG00000052305
Lpin2	11	8.390	1.59	3.02	0.01	ENSMUSG00000004044
Hist1h2ab	1	2.420	1.59	3.02	0.01	ENSMUSG00000026483
Ppp2r5b	X	5.310	1.59	3.02	0.01	ENSMUSG00000031133
Cbx7	5	1.640	1.59	3.02	0.04	ENSMUSG00000051339
Nfkbia	3	35.810	1.60	3.03	0.00	ENSMUSG00000028035
Ahnak2	4	67.200	1.60	3.03	0.00	ENSMUSG00000040128
Dstyk	6	174.010	1.60	3.03	0.00	ENSMUSG00000001156
Ptpdc1	7	20.890	1.60	3.03	0.01	ENSMUSG00000068566
Akr1b8	4	17.510	1.61	3.04	0.00	ENSMUSG00000041936
Col4a6	19	1.700	1.60	3.04	0.05	ENSMUSG00000025085
Zfp703	7	3.210	1.61	3.05	0.01	ENSMUSG00000074364
Mid2	6	18.450	1.61	3.05	0.03	ENSMUSG00000029661

Jph2	8	1.370	1.61	3.05	0.03	ENSMUSG00000031681
Inhbb	8	5.530	1.61	3.05	0.04	ENSMUSG00000031700
Pcolce2	2	3.290	1.61	3.05	0.04	ENSMUSG00000026821
Ltbp3	14	3.260	1.61	3.05	0.05	ENSMUSG00000025555
Rhbdf1	11	74.990	1.62	3.06	0.00	ENSMUSG00000020390
Fn1	7	90.980	1.61	3.06	0.00	ENSMUSG00000030748
Hist1h1d	17	55.620	1.61	3.06	0.00	ENSMUSG00000067212
Oas3	5	6.670	1.61	3.06	0.03	ENSMUSG00000029674
Tns2	11	16.320	1.62	3.07	0.00	ENSMUSG00000044068
Zfp36	11	118554.950	1.62	3.07	0.00	ENSMUSG00000069917
Mlph	11	43.580	1.62	3.08	0.00	ENSMUSG00000020893
Tgfa	2	18.220	1.62	3.08	0.00	ENSMUSG00000026837
Hist1h3b	9	3.960	1.62	3.08	0.00	ENSMUSG00000032204
Bpgm	15	0.670	1.62	3.08	0.04	ENSMUSG00000022358
Daam2	9	24.260	1.63	3.09	0.02	ENSMUSG00000032085
Fat1	3	30.480	1.63	3.09	0.02	ENSMUSG00000002233
Slc7a11	X	20.190	1.63	3.10	0.00	ENSMUSG00000039382
Met	16	11.750	1.63	3.10	0.00	ENSMUSG00000022965
Prss23	5	10.120	1.63	3.10	0.02	ENSMUSG00000029484
Hist1h2ae	15	2.250	1.63	3.10	0.02	ENSMUSG00000075590
Mcam	2	284.400	1.64	3.11	0.00	ENSMUSG00000015932
Slc6a20a	7	8.210	1.64	3.11	0.00	ENSMUSG00000030630
Thbs2	19	18.110	1.64	3.11	0.01	ENSMUSG00000058216
Elmo1	2	85.090	1.64	3.12	0.00	ENSMUSG00000032802
Mycn	4	60.730	1.64	3.12	0.00	ENSMUSG00000028756
Suco	10	4.870	1.64	3.12	0.03	ENSMUSG00000020263
Ak1	14	4.920	1.64	3.12	0.05	ENSMUSG00000022090
Slit3	19	2.320	1.64	3.13	0.01	ENSMUSG00000053617
Gja4	6	3.970	1.64	3.13	0.03	ENSMUSG00000010797
Hist2h3c2	4	12.910	1.65	3.14	0.01	ENSMUSG00000050390
Bmp8a	7	63.120	1.65	3.14	0.01	ENSMUSG00000030605
Dusp8	19	4.330	1.65	3.14	0.04	ENSMUSG00000041488
Ptger4	6	51.850	1.65	3.15	0.00	ENSMUSG00000030083

Ddit4	8	1.490	1.65	3.15	0.04	ENSMUSG00000071176
Hist1h4i	14	25.210	1.66	3.16	0.00	ENSMUSG00000037526
Ldlrad4	5	4.940	1.66	3.16	0.00	ENSMUSG00000039782
Trp53inp1	11	2.240	1.66	3.16	0.02	ENSMUSG00000001583
Sik1	17	0.770	1.66	3.16	0.05	ENSMUSG00000024087
Psca	3	19.170	1.66	3.17	0.00	ENSMUSG00000036894
Csf2rb2	2	116.020	1.66	3.17	0.00	ENSMUSG00000027346
Ccdc141	11	2.460	1.66	3.17	0.01	ENSMUSG00000025175
Itpr2	3	3.540	1.67	3.17	0.01	ENSMUSG00000027966
Smim24	2	6.090	1.67	3.17	0.01	ENSMUSG00000027077
Lama5	4	3.140	1.67	3.17	0.03	ENSMUSG00000039813
Cx3cl1	10	118.570	1.67	3.18	0.00	ENSMUSG00000019838
Notch3	15	376.220	1.67	3.19	0.00	ENSMUSG00000071713
Dyrk3	4	18.870	1.67	3.19	0.02	ENSMUSG00000035692
Tcp11l1	19	26.310	1.68	3.20	0.00	ENSMUSG00000025025
Plau	3	5.520	1.68	3.21	0.00	ENSMUSG00000068854
Slc44a1	18	6.390	1.68	3.21	0.00	ENSMUSG00000024521
Hmgcs2	19	48.350	1.68	3.21	0.01	ENSMUSG00000035783
Myocd	9	0.810	1.68	3.21	0.05	ENSMUSG00000050503
Prune2	17	38.600	1.69	3.22	0.00	ENSMUSG00000024190
Agtr1a	2	15.900	1.69	3.22	0.00	ENSMUSG00000050373
Ypel2	13	15.990	1.69	3.22	0.00	ENSMUSG00000021420
Myh11	18	2.270	1.69	3.22	0.02	ENSMUSG00000024331
Adgrf5	19	2.030	1.69	3.22	0.04	ENSMUSG00000016496
Col3a1	11	213.640	1.69	3.22	0.04	ENSMUSG00000018339
Rhov	8	16.590	1.69	3.23	0.00	ENSMUSG00000035671
Cacna2d3	1	136.530	1.69	3.23	0.00	ENSMUSG00000020423
Ccdc9b	8	8.360	1.69	3.23	0.04	ENSMUSG00000031955
Tent5b	11	2.530	1.70	3.24	0.03	ENSMUSG00000035413
Cd109	7	7663.920	1.70	3.25	0.00	ENSMUSG00000050708
Igfbp2	12	7.350	1.70	3.25	0.03	ENSMUSG00000040856
Rapgef3	15	3.770	1.70	3.25	0.04	ENSMUSG00000046761
Hr	11	15.820	1.70	3.26	0.00	ENSMUSG00000044352

Bmp4	4	25.540	1.70	3.26	0.00	ENSMUSG00000028716
Sqstm1	3	3.670	1.70	3.26	0.02	ENSMUSG00000027803
Svep1	6	25.560	1.71	3.27	0.00	ENSMUSG00000030095
Tnc	1	1.430	1.71	3.27	0.04	ENSMUSG00000041757
Nfil3	1	14.180	1.71	3.28	0.00	ENSMUSG00000066800
Nfic	13	7.820	1.71	3.28	0.03	ENSMUSG00000054889
Npl	4	114.060	1.72	3.29	0.00	ENSMUSG00000036002
Anxa8	14	39.180	1.73	3.31	0.00	ENSMUSG00000021998
Osgin1	14	21.010	1.73	3.31	0.00	ENSMUSG00000022225
BC107364	16	1.650	1.73	3.31	0.02	ENSMUSG00000033306
Htra1	3	1.130	1.73	3.31	0.04	ENSMUSG00000031286
Ypel3	10	199.180	1.73	3.32	0.00	ENSMUSG00000020034
1700017B05Rik	1	6.100	1.73	3.32	0.01	ENSMUSG00000026042
Bcl6	X	3.820	1.73	3.32	0.02	ENSMUSG00000040147
Lox	3	0.600	1.73	3.32	0.05	ENSMUSG00000053706
Hlx	12	1.610	1.74	3.33	0.00	ENSMUSG00000020646
Cacna1g	3	1.130	1.74	3.33	0.01	ENSMUSG00000053819
Pvr	4	2.410	1.74	3.33	0.05	ENSMUSG00000061859
Kcnb1	15	98.730	1.74	3.34	0.00	ENSMUSG00000081534
Fabp4	11	285.500	1.74	3.34	0.00	ENSMUSG00000050860
Lgmn	10	2.460	1.74	3.34	0.01	ENSMUSG00000020020
Gfpt2	19	102.480	1.74	3.34	0.05	ENSMUSG00000024990
Cd80	14	119.110	1.74	3.35	0.00	ENSMUSG00000021792
Tgm1	11	18.820	1.74	3.35	0.00	ENSMUSG00000037573
Aldh1a1	19	4.970	1.75	3.35	0.02	ENSMUSG00000048612
Plekha4	15	16.820	1.75	3.35	0.02	ENSMUSG00000022150
Zfp773	X	1.820	1.75	3.35	0.03	ENSMUSG00000046942
Mafa	11	2.340	1.74	3.35	0.04	ENSMUSG00000017639
Myo5c	17	164.160	1.75	3.36	0.00	ENSMUSG00000023952
Lhfp	3	9.120	1.75	3.36	0.00	ENSMUSG00000048458
Sema3a	7	15.400	1.75	3.36	0.03	ENSMUSG00000025504
Rpp25	X	49.500	1.75	3.37	0.00	ENSMUSG00000031438
Pnp2	4	1.710	1.75	3.37	0.01	ENSMUSG00000028572

Vegfd	10	8.810	1.75	3.37	0.01	ENSMUSG00000015312
Hbq1a	15	5.340	1.75	3.37	0.03	ENSMUSG00000023022
Nlrc5	9	7.860	1.75	3.37	0.04	ENSMUSG00000046402
Bbc3	11	43.010	1.76	3.38	0.00	ENSMUSG00000037124
Hist1h2bc	7	7.170	1.76	3.38	0.00	ENSMUSG00000078606
Pdk4	3	8.770	1.76	3.38	0.00	ENSMUSG00000038205
Pltp	18	3.120	1.76	3.38	0.00	ENSMUSG00000024268
Hectd2	10	3.640	1.76	3.38	0.02	ENSMUSG00000019872
Perm1	15	17.300	1.76	3.38	0.02	ENSMUSG00000036606
Cryaa	7	8.030	1.76	3.38	0.02	ENSMUSG00000042759
Ugdh	11	94.610	1.76	3.39	0.00	ENSMUSG00000082101
Nipal4	11	74.950	1.76	3.39	0.01	ENSMUSG00000001506
Gm2163	9	3.480	1.76	3.39	0.03	ENSMUSG00000032194
Gprin2	8	21.520	1.77	3.40	0.00	ENSMUSG00000006362
Eda2r	9	6.010	1.77	3.41	0.04	ENSMUSG00000037405
Msln	10	1.390	1.77	3.41	0.04	ENSMUSG00000020019
Raet1e	1	2242.630	1.77	3.42	0.00	ENSMUSG00000042066
Medag	11	56.460	1.78	3.42	0.00	ENSMUSG00000018572
Zfp874a	2	2.580	1.77	3.42	0.01	ENSMUSG00000027204
Gpat2	18	1.490	1.77	3.42	0.03	ENSMUSG00000039616
Cav1	10	349.650	1.78	3.43	0.00	ENSMUSG00000025408
Eepd1	7	787.630	1.78	3.43	0.00	ENSMUSG00000040435
Zfp365	2	4.060	1.78	3.43	0.00	ENSMUSG00000074923
Hist1h2bg	7	6.680	1.78	3.43	0.03	ENSMUSG00000030494
Hsd3b6	15	22.220	1.78	3.44	0.00	ENSMUSG00000054619
Cdkn1a	11	2657.960	1.78	3.44	0.00	ENSMUSG00000019505
Nsmf	1	2.540	1.78	3.44	0.00	ENSMUSG00000026235
Col12a1	18	1.180	1.78	3.44	0.01	ENSMUSG00000045094
Jag1	7	0.710	1.78	3.44	0.02	ENSMUSG00000047767
Peli3	19	14.610	1.79	3.45	0.00	ENSMUSG00000025225
Junb	7	1.380	1.79	3.45	0.03	ENSMUSG00000025584
Serpine1	8	3.900	1.79	3.45	0.04	ENSMUSG00000039960
Tmem88b	6	4.340	1.79	3.45	0.05	ENSMUSG00000059430

Ildr2	7	2.750	1.79	3.46	0.00	ENSMUSG00000030643
Gramd2	7	0.330	1.79	3.46	0.03	ENSMUSG00000011751
Gstp2	6	13.900	1.80	3.48	0.00	ENSMUSG00000049409
Pyroxd2	6	61.550	1.80	3.48	0.00	ENSMUSG00000032712
Fam220a_1	5	4.900	1.80	3.48	0.03	ENSMUSG00000029381
Cemip	4	1.770	1.80	3.48	0.04	ENSMUSG00000029059
Rerg	9	23.020	1.80	3.49	0.00	ENSMUSG00000023186
Fasl	10	84.290	1.80	3.49	0.00	ENSMUSG00000020184
Hist1h3d	7	19.330	1.80	3.49	0.00	ENSMUSG00000030688
Eml1	4	0.230	1.80	3.49	0.04	ENSMUSG00000054752
Bhlhe41	12	10.130	1.81	3.50	0.00	ENSMUSG00000021226
Gm4779	3	10.540	1.81	3.50	0.02	ENSMUSG00000028128
Tmem140	4	26.340	1.82	3.52	0.00	ENSMUSG00000073700
Tac2	4	105.900	1.82	3.52	0.00	ENSMUSG00000028437
Abhd3	11	7.490	1.82	3.52	0.02	ENSMUSG00000001507
Cep112	11	3.480	1.82	3.52	0.03	ENSMUSG00000020773
Hspb7	18	0.170	1.81	3.52	0.05	ENSMUSG00000071847
Hist1h4m	18	0.350	1.82	3.53	0.04	ENSMUSG00000024617
Tecta	5	12.910	1.82	3.54	0.00	ENSMUSG00000042453
D630023F18Rik	1	93.470	1.83	3.54	0.00	ENSMUSG00000041801
Mapre3	10	2.190	1.82	3.54	0.05	ENSMUSG00000006342
Gcm2	1	0.310	1.82	3.54	0.05	ENSMUSG00000026674
Zfp354b	X	30.690	1.83	3.56	0.00	ENSMUSG00000025151
Gja5	1	32.860	1.83	3.56	0.00	ENSMUSG00000026628
Gpc1	16	4.560	1.83	3.56	0.03	ENSMUSG00000022505
Serpina3n	3	1.690	1.83	3.56	0.04	ENSMUSG00000027956
Lef1	10	15.500	1.83	3.56	0.04	ENSMUSG00000035041
Isg20	4	0.840	1.84	3.57	0.05	ENSMUSG00000025328
Mmp12	8	2.710	1.84	3.58	0.00	ENSMUSG00000025812
Dst	11	3.690	1.84	3.58	0.00	ENSMUSG00000020490
Fosl2	3	1.040	1.84	3.58	0.05	ENSMUSG00000027536
Hist2h2aa2	11	40.470	1.84	3.59	0.02	ENSMUSG00000020911
Clgn	17	22.710	1.85	3.60	0.00	ENSMUSG00000024173

Trim43a	4	1.060	1.85	3.60	0.02	ENSMUSG00000015243
Olfml2b	6	12.310	1.85	3.60	0.03	ENSMUSG00000046733
AW011738	4	177.880	1.85	3.61	0.00	ENSMUSG00000040859
Ltbp4	9	2.420	1.86	3.63	0.00	ENSMUSG00000010051
Rab39b	5	2.290	1.86	3.63	0.01	ENSMUSG00000045348
Ppl	11	527.550	1.87	3.64	0.00	ENSMUSG00000020802
Plk2	4	3.140	1.86	3.64	0.00	ENSMUSG00000028943
Cystm1	2	11.160	1.87	3.65	0.00	ENSMUSG00000038375
Lexm	4	3.530	1.87	3.65	0.02	ENSMUSG00000039911
Spag8	2	244.400	1.87	3.66	0.00	ENSMUSG00000034059
Hist3h2a	17	8.130	1.87	3.66	0.00	ENSMUSG00000056116
Rundc3a	4	0.940	1.87	3.66	0.02	ENSMUSG00000047875
Rnf227	15	0.330	1.87	3.66	0.03	ENSMUSG00000053469
Lrrc15	19	6.210	1.88	3.67	0.00	ENSMUSG00000025171
Des	11	2.200	1.88	3.67	0.01	ENSMUSG00000046731
Apol11b	16	3.680	1.88	3.67	0.05	ENSMUSG00000022949
Apol10a	13	7.360	1.87	3.67	0.05	ENSMUSG00000116016
Cygb	11	45.820	1.88	3.68	0.00	ENSMUSG00000018476
Tgfb3	6	859.210	1.88	3.68	0.00	ENSMUSG00000036390
Hist1h2bb	15	0.630	1.88	3.68	0.01	ENSMUSG00000022197
Hist1h2bj	16	1.280	1.88	3.68	0.02	ENSMUSG00000022665
Unc5cl	2	2.270	1.88	3.68	0.03	ENSMUSG00000027173
Col8a1	13	3.360	1.88	3.68	0.03	ENSMUSG00000062727
Wbp2nl	2	0.880	1.88	3.69	0.04	ENSMUSG00000027171
A530064D06Rik	11	8.790	1.89	3.70	0.00	ENSMUSG00000032740
Tmem200b	10	1.360	1.89	3.70	0.03	ENSMUSG00000040502
Cyp17a1	6	14.690	1.89	3.70	0.03	ENSMUSG00000055912
Insl6	10	34.200	1.89	3.71	0.00	ENSMUSG00000034854
Pgm5	3	51.320	1.89	3.71	0.00	ENSMUSG00000068744
Lama2	1	3.630	1.89	3.71	0.01	ENSMUSG00000038776
Neo1	14	4.160	1.89	3.72	0.00	ENSMUSG00000025277
Osr1	5	3.880	1.90	3.72	0.04	ENSMUSG00000038859
Jun	7	0.770	1.90	3.72	0.05	ENSMUSG00000030483

Thy1	6	11.870	1.90	3.73	0.00	ENSMUSG00000038451
Cd163	3	52.040	1.90	3.73	0.00	ENSMUSG00000103034
1700014D04Rik	3	7.650	1.90	3.73	0.02	ENSMUSG00000027750
Bgn	X	20.530	1.90	3.74	0.00	ENSMUSG00000041688
Rnf32	3	4.800	1.90	3.74	0.00	ENSMUSG00000051000
Hist1h4n	10	13.040	1.90	3.74	0.02	ENSMUSG00000020099
P2rx7	5	26.380	1.91	3.75	0.00	ENSMUSG00000042726
Cfap69	7	0.580	1.91	3.75	0.05	ENSMUSG00000006310
Rsph9	4	162.340	1.91	3.76	0.00	ENSMUSG00000028893
Ace2	15	1.670	1.91	3.76	0.01	ENSMUSG00000044636
Tmem71	8	0.780	1.91	3.77	0.03	ENSMUSG00000002908
Hist1h3i	19	13.260	1.92	3.78	0.00	ENSMUSG00000083282
Ugt8a	10	5.610	1.92	3.78	0.00	ENSMUSG00000033318
Lama4	6	4.690	1.92	3.78	0.01	ENSMUSG00000015766
Tll1	14	0.100	1.92	3.78	0.05	ENSMUSG00000049092
Ifitm10	18	4.140	1.92	3.79	0.00	ENSMUSG00000063889
Foxf1	3	7.970	1.92	3.79	0.02	ENSMUSG00000028164
Igfbp5	8	26.140	1.92	3.79	0.03	ENSMUSG00000000303
Radil	2	1.520	1.92	3.79	0.04	ENSMUSG00000004105
Hsd11b1	11	10.730	1.92	3.79	0.04	ENSMUSG00000018569
Sspn	4	14.860	1.93	3.80	0.00	ENSMUSG00000028982
Klhl38	8	4.120	1.93	3.80	0.01	ENSMUSG00000034845
Pla2r1	3	3.560	1.93	3.80	0.04	ENSMUSG00000028064
Igfn1	5	2.250	1.93	3.80	0.05	ENSMUSG00000029330
Paqr9	14	56.020	1.93	3.81	0.00	ENSMUSG00000040997
Hist1h2ac	13	146.050	1.93	3.81	0.00	ENSMUSG00000036181
Nrtn	7	270.240	1.93	3.81	0.01	ENSMUSG00000002985
Serinc2	17	5.360	1.93	3.81	0.05	ENSMUSG00000044279
Cldn10	11	30.200	1.93	3.82	0.00	ENSMUSG00000020806
Mindy4	11	5.860	1.93	3.82	0.01	ENSMUSG00000034947
Rph3al	9	1.440	1.93	3.82	0.05	ENSMUSG00000053646
Arhgef19	14	35.800	1.94	3.83	0.00	ENSMUSG00000022124
Tmprss9	13	2.160	1.94	3.83	0.01	ENSMUSG00000051627

Cacna2d4	16	1.560	1.94	3.83	0.01	ENSMUSG00000022887
Klhl22	2	1.570	1.94	3.83	0.03	ENSMUSG00000001864
Kcnj4	8	0.820	1.94	3.83	0.04	ENSMUSG00000074215
Thegl	17	2.600	1.94	3.84	0.00	ENSMUSG00000059142
Shroom4	13	5.630	1.94	3.84	0.03	ENSMUSG00000021411
Gm10382	4	1.020	1.94	3.84	0.04	ENSMUSG00000028434
Hist1h2bf	7	0.980	1.94	3.84	0.05	ENSMUSG00000035713
Apol11a	11	0.220	1.94	3.85	0.05	ENSMUSG00000047759
Tmtc1	10	29.630	1.95	3.86	0.00	ENSMUSG00000020205
Spata31d1a	18	116.230	1.95	3.86	0.00	ENSMUSG00000045730
Grhl3	15	165.690	1.95	3.86	0.02	ENSMUSG00000064373
Hist3h2ba	15	0.420	1.95	3.86	0.02	ENSMUSG00000016028
Hist2h2aa1	11	0.870	1.95	3.86	0.03	ENSMUSG00000045377
Gm45208	11	3.900	1.95	3.86	0.03	ENSMUSG00000076435
Ackr3	4	0.630	1.95	3.86	0.04	ENSMUSG00000028469
Gm2115	12	6.080	1.95	3.86	0.04	ENSMUSG00000060716
Hist1h2be	9	43.200	1.96	3.88	0.00	ENSMUSG00000043953
Eln	14	11.340	1.96	3.88	0.00	ENSMUSG00000037580
Oprd1	15	1.430	1.96	3.88	0.01	ENSMUSG00000022237
Relb	6	7.120	1.95	3.88	0.03	ENSMUSG00000051343
Xlr4a	17	4.060	1.95	3.88	0.05	ENSMUSG00000023959
Galnt6	17	116.200	1.96	3.90	0.00	ENSMUSG00000034509
Hmox1	17	5.060	1.96	3.90	0.00	ENSMUSG00000047407
Nog	18	0.410	1.96	3.90	0.03	ENSMUSG00000039954
Adm	9	477.970	1.96	3.90	0.04	ENSMUSG00000032083
Cdkn2b	7	5.390	1.97	3.91	0.00	ENSMUSG00000007783
C1ra	2	2.260	1.97	3.91	0.01	ENSMUSG00000027199
Rapgef4	6	440.530	1.97	3.92	0.00	ENSMUSG00000029922
Klk6	19	25.710	1.97	3.92	0.00	ENSMUSG00000018821
Trim34b	10	0.160	1.97	3.92	0.03	ENSMUSG00000020181
Sox8	3	9.870	1.98	3.93	0.02	ENSMUSG00000028158
Me3	3	39.640	1.98	3.94	0.00	ENSMUSG00000001419
Hist2h2bb	10	6.050	1.98	3.94	0.02	ENSMUSG00000019929

Brdt	14	0.710	1.98	3.94	0.05	ENSMUSG00000042485
Mx2	5	2637.210	1.98	3.94	0.05	ENSMUSG00000054932
Mmp13	5	18.060	1.98	3.95	0.00	ENSMUSG00000059325
Il1rl2	X	0.310	1.98	3.95	0.05	ENSMUSG00000059493
Foxd1	18	6.010	1.99	3.96	0.03	ENSMUSG00000044393
Mettl7a2	16	0.470	1.99	3.96	0.03	ENSMUSG00000032940
Kcnj12	2	0.500	1.99	3.96	0.05	ENSMUSG00000099041
Col5a3	10	1.320	1.99	3.97	0.02	ENSMUSG00000038522
Afm	1	1.190	1.99	3.97	0.03	ENSMUSG00000026153
Mmp27	7	0.270	1.99	3.97	0.04	ENSMUSG00000009545
Bmp8b	7	7.780	1.99	3.98	0.04	ENSMUSG00000040703
Ptx3	11	1.860	1.99	3.98	0.05	ENSMUSG00000039329
Serpina3m	10	0.760	1.99	3.99	0.02	ENSMUSG00000037171
Mmel1	9	1.640	2.00	3.99	0.04	ENSMUSG00000044037
Mrgprf	11	1.950	2.00	4.00	0.01	ENSMUSG00000045667
Igfbp6	2	0.320	2.00	4.00	0.05	ENSMUSG00000028640
Ptpn14	2	1.920	2.01	4.01	0.05	ENSMUSG00000026994
Slc10a6	17	167.950	2.01	4.02	0.00	ENSMUSG00000073411
Rassf10	7	11.970	2.01	4.02	0.00	ENSMUSG00000030699
Gm45716	8	330.520	2.01	4.03	0.00	ENSMUSG00000071076
Tmem28	12	10.580	2.01	4.03	0.01	ENSMUSG00000072825
Col22a1	2	1.930	2.01	4.03	0.01	ENSMUSG00000027233
Foxe1	7	0.490	2.01	4.04	0.03	ENSMUSG00000041343
Fosl1	14	5.020	2.02	4.05	0.05	ENSMUSG00000046352
Arl4c	4	10.600	2.02	4.06	0.00	ENSMUSG00000028680
Ttc22	7	6.720	2.02	4.06	0.03	ENSMUSG00000042659
Vgf	6	2.010	2.02	4.06	0.05	ENSMUSG00000054474
Oasl2	9	353.910	2.02	4.06	0.05	ENSMUSG00000032080
Prelp	10	2.610	2.03	4.07	0.00	ENSMUSG00000019947
Mettl7a3	18	2.130	2.02	4.07	0.00	ENSMUSG00000019647
Map3k19	2	4.640	2.03	4.07	0.01	ENSMUSG00000087006
Gm43518	2	1.180	2.03	4.07	0.03	ENSMUSG00000026825
Gdf1	4	39.640	2.03	4.08	0.00	ENSMUSG00000096351

Apol9a	9	13.660	2.03	4.08	0.00	ENSMUSG00000032380
Zfp750	17	183.450	2.03	4.09	0.00	ENSMUSG00000024052
H2-Bl	6	6.920	2.04	4.10	0.02	ENSMUSG00000033542
Tex11	13	2.170	2.04	4.11	0.04	ENSMUSG00000061615
Dglucy	19	8.330	2.05	4.13	0.00	ENSMUSG00000024777
Fam196b	5	118.710	2.04	4.13	0.02	ENSMUSG00000047501
Dusp13	15	9.000	2.05	4.14	0.00	ENSMUSG00000053411
Cebpa	7	10.110	2.05	4.14	0.03	ENSMUSG00000010760
Hist1h2bq	12	23.440	2.05	4.15	0.00	ENSMUSG00000021025
Meioc	12	4.150	2.05	4.15	0.00	ENSMUSG00000072812
Nuak2	7	10.260	2.05	4.15	0.01	ENSMUSG00000003420
Tnxb	5	3.950	2.05	4.15	0.03	ENSMUSG00000070780
Npffr1	1	14.730	2.06	4.16	0.00	ENSMUSG00000042046
Pbx4	12	5.770	2.06	4.16	0.00	ENSMUSG00000021062
Col14a1	13	0.370	2.06	4.16	0.03	ENSMUSG00000038042
Spink8	6	4.160	2.06	4.17	0.01	ENSMUSG00000029762
Prrg3	10	1.810	2.06	4.17	0.03	ENSMUSG00000020121
Plac9b	X	0.390	2.06	4.18	0.03	ENSMUSG00000031273
Ankrd34b	8	10.530	2.07	4.19	0.00	ENSMUSG00000085795
Fmod	16	9.580	2.07	4.19	0.01	ENSMUSG00000022893
Tcim	4	9.430	2.07	4.19	0.05	ENSMUSG00000028591
Fbxo27	X	0.200	2.07	4.19	0.05	ENSMUSG00000000266
Lrtm2	2	1.030	2.07	4.20	0.01	ENSMUSG00000017817
Nyx	7	2.990	2.07	4.20	0.04	ENSMUSG00000000154
Gdpd3	1	0.240	2.07	4.20	0.05	ENSMUSG00000037035
Gli1	10	0.440	2.07	4.21	0.04	ENSMUSG00000039981
Prex2	9	0.160	2.07	4.21	0.05	ENSMUSG00000015354
Irf2bpl	2	13.850	2.08	4.22	0.02	ENSMUSG00000017009
Eps8l1	19	0.640	2.08	4.23	0.04	ENSMUSG00000024940
Hist1h2br	11	19.810	2.08	4.24	0.00	ENSMUSG00000020282
Gm49337	15	0.450	2.08	4.24	0.05	ENSMUSG00000061740
Hist1h2ao	X	10.950	2.09	4.25	0.04	ENSMUSG00000031410
Fam71a	1	109.590	2.09	4.26	0.00	ENSMUSG00000026193

Gdf15	13	0.580	2.09	4.26	0.01	ENSMUSG00000052565
Nnmt	18	6.200	2.10	4.27	0.03	ENSMUSG00000024395
Rsad2	5	0.310	2.09	4.27	0.03	ENSMUSG00000032661
Cd79a	11	3.100	2.10	4.28	0.04	ENSMUSG00000037335
Kcnh3	3	0.840	2.10	4.28	0.04	ENSMUSG00000049404
Lypd5	X	33.500	2.10	4.29	0.01	ENSMUSG00000055653
Galnt15	15	2.760	2.10	4.29	0.01	ENSMUSG00000037003
Itgbl1	18	29.730	2.10	4.29	0.03	ENSMUSG00000024503
Abca8b	13	2.630	2.10	4.29	0.04	ENSMUSG00000069309
Slc25a31	7	36.250	2.11	4.30	0.00	ENSMUSG00000044786
Hist1h2ad	10	4.520	2.10	4.30	0.05	ENSMUSG00000034917
Tmc3	1	3.140	2.11	4.31	0.01	ENSMUSG00000026303
Cyp2a5	6	2.240	2.11	4.31	0.01	ENSMUSG00000029999
Il1r1	13	1.760	2.11	4.31	0.03	ENSMUSG00000069267
Il1r2	6	307.300	2.11	4.32	0.00	ENSMUSG00000038871
Scg2	17	1.100	2.11	4.32	0.05	ENSMUSG00000040260
Chga	8	2.900	2.11	4.33	0.00	ENSMUSG00000070047
Aqp1	17	3.190	2.12	4.33	0.02	ENSMUSG00000050747
Spata31d1d	3	6.500	2.12	4.34	0.02	ENSMUSG00000039131
Gm14226	9	6.430	2.12	4.35	0.03	ENSMUSG00000038775
Klk1b22	3	6.410	2.13	4.36	0.00	ENSMUSG00000027737
Ybx2	5	4.270	2.13	4.36	0.01	ENSMUSG00000028970
Ly6k	9	11.060	2.13	4.37	0.01	ENSMUSG00000052911
Acsm1	10	8.150	2.13	4.37	0.02	ENSMUSG00000018166
Zfp37	13	0.250	2.13	4.38	0.01	ENSMUSG00000021313
Fam180a	6	2.630	2.14	4.39	0.00	ENSMUSG00000009376
Ephx4	7	5.240	2.14	4.40	0.00	ENSMUSG00000039405
Piwil2	13	3.940	2.14	4.40	0.02	ENSMUSG00000069272
Papolb	9	2.570	2.14	4.41	0.03	ENSMUSG00000032135
Rtn4rl2	9	0.930	2.14	4.42	0.01	ENSMUSG00000036814
Gpbar1	17	0.340	2.14	4.42	0.03	ENSMUSG00000023885
Efemp1	14	2.940	2.14	4.42	0.04	ENSMUSG00000015970
Sstr5	9	0.350	2.14	4.42	0.05	ENSMUSG00000037681

Myod1	13	6.080	2.15	4.43	0.00	ENSMUSG00000041112
Bcas1	12	5.460	2.15	4.43	0.00	ENSMUSG00000037169
Sim2	4	5.200	2.15	4.43	0.03	ENSMUSG00000029032
Il1f9	11	0.370	2.15	4.43	0.05	ENSMUSG00000023781
Tac1	1	75.350	2.15	4.44	0.00	ENSMUSG00000040297
Usp17ld	2	2.360	2.15	4.44	0.00	ENSMUSG00000026817
Foxc2	11	0.610	2.15	4.44	0.02	ENSMUSG00000056427
Prnd	3	39.450	2.15	4.44	0.03	ENSMUSG00000050359
Ms4a4d	4	3.330	2.15	4.45	0.00	ENSMUSG00000050234
Olig2	3	4.050	2.15	4.45	0.01	ENSMUSG00000081058
Prg4	4	0.670	2.15	4.45	0.02	ENSMUSG00000032726
Slc19a3	7	34.140	2.16	4.46	0.00	ENSMUSG00000037887
Ugt1a8	15	9.670	2.16	4.46	0.00	ENSMUSG00000039942
Gm9780	17	3.420	2.16	4.46	0.02	ENSMUSG00000035929
Sprr2k	10	42.390	2.16	4.47	0.00	ENSMUSG00000020108
Gpr139	11	0.470	2.16	4.47	0.03	ENSMUSG00000043099
Gm20547	3	1.720	2.17	4.49	0.03	ENSMUSG00000015850

Supplemental Table 5. Downregulated genes in *Dot1L*- KO ESRE cells in common with D*ot1L*- MM cells (n=205)

Name	Chromosome	Max group mean	Log_2 fold change	Fold change	P-value	ENSEMBL
Ncan	8	0.160	-7.35	-163.54	0.01	ENSMUSG00000002341
BC035044	6	0.640	-7.29	-156.30	0.00	ENSMUSG00000090164
Scrt2	2	0.270	-7.06	-133.05	0.01	ENSMUSG00000060257
Cacng4	11	0.260	-7.04	-131.20	0.01	ENSMUSG00000020723
Mpo	11	37.630	-6.88	-118.14	0.00	ENSMUSG00000009350
Dcx	X	0.260	-6.64	-99.52	0.00	ENSMUSG00000031285
Igfbpl1	4	0.230	-6.59	-96.45	0.02	ENSMUSG00000035551
Brsk2	7	0.110	-6.53	-92.38	0.02	ENSMUSG00000053046
Nr2f1	13	0.130	-6.53	-92.38	0.02	ENSMUSG00000069171
Pax2	19	0.120	-6.53	-92.38	0.02	ENSMUSG00000004231
Astn1	1	0.070	-6.50	-90.35	0.02	ENSMUSG00000026587
Ptpn5	7	0.170	-6.46	-88.32	0.02	ENSMUSG00000030854
Prl2b1	13	0.620	-6.42	-85.57	0.01	ENSMUSG00000069258
Il12a	3	0.360	-6.28	-77.54	0.02	ENSMUSG00000027776
Flt3	5	0.810	-6.20	-73.38	0.00	ENSMUSG00000042817
Sprr1b	3	0.580	-6.13	-70.01	0.03	ENSMUSG00000048455
Prtn3	10	7.170	-6.09	-68.28	0.00	ENSMUSG00000057729
Rab3c	13	0.050	-6.09	-67.97	0.03	ENSMUSG00000021700
Apba2	7	0.110	-5.85	-57.80	0.03	ENSMUSG00000030519
Nhlh2	3	0.070	-5.75	-53.73	0.04	ENSMUSG00000048540
Gm32687	10	0.140	-5.72	-52.66	0.03	ENSMUSG00000112640
B3gat1	9	0.070	-5.57	-47.62	0.04	ENSMUSG00000045994
Dcc	18	0.110	-5.51	-45.45	0.00	ENSMUSG00000060534
Actl6b	5	0.160	-5.38	-41.51	0.05	ENSMUSG00000029712
Gm9833	3	0.140	-5.34	-40.44	0.05	ENSMUSG00000049230
Bex6	16	0.210	-5.05	-33.10	0.05	ENSMUSG00000075269
Trim67	8	0.080	-4.83	-28.36	0.01	ENSMUSG00000036913
Plppr3	10	1.030	-4.81	-28.09	0.00	ENSMUSG00000035835
Chl1	6	0.070	-4.77	-27.33	0.00	ENSMUSG00000030077
Stmn3	2	0.550	-4.69	-25.80	0.01	ENSMUSG00000027581

Map2	1	0.340	-4.68	-25.67	0.00	ENSMUSG00000015222
Lrrc4b	7	0.240	-4.59	-24.13	0.01	ENSMUSG00000047085
Lhx1	11	0.160	-4.47	-22.20	0.02	ENSMUSG00000018698
Gria2	3	0.050	-4.41	-21.30	0.01	ENSMUSG00000033981
Fxyd2	9	1.820	-4.35	-20.43	0.00	ENSMUSG00000059412
Atcay	10	0.130	-4.33	-20.11	0.02	ENSMUSG00000034958
44807	15	0.340	-4.25	-19.00	0.00	ENSMUSG00000022456
Elavl3	9	0.220	-4.07	-16.76	0.00	ENSMUSG00000003410
Myt1	2	0.120	-3.99	-15.84	0.02	ENSMUSG00000010505
Shd	17	0.180	-3.92	-15.09	0.03	ENSMUSG00000039154
Nfasc	1	0.080	-3.88	-14.67	0.01	ENSMUSG00000026442
Stmn2	3	0.800	-3.86	-14.49	0.01	ENSMUSG00000027500
Apc2	10	0.310	-3.82	-14.09	0.00	ENSMUSG00000020135
Celf3	3	0.280	-3.81	-14.03	0.02	ENSMUSG00000028137
Nefl	14	0.220	-3.70	-12.98	0.02	ENSMUSG00000022055
Chrna4	2	0.180	-3.61	-12.20	0.01	ENSMUSG00000027577
Gng3	19	0.170	-3.55	-11.70	0.05	ENSMUSG00000071658
Zfp979	4	2.320	-3.53	-11.55	0.00	ENSMUSG00000066000
St8sia1	6	0.090	-3.50	-11.30	0.01	ENSMUSG00000030283
Psma8	18	0.990	-3.41	-10.60	0.00	ENSMUSG00000036743
Mug1	6	0.090	-3.38	-10.40	0.02	ENSMUSG00000059908
Exoc3l2	7	1.210	-3.20	-9.19	0.00	ENSMUSG00000011263
Klk8	7	1.520	-3.13	-8.76	0.00	ENSMUSG00000064023
St8sia4	1	0.210	-3.12	-8.72	0.00	ENSMUSG00000040710
Nsg2	11	0.250	-3.04	-8.25	0.03	ENSMUSG00000020297
Serpinb10	1	2.290	-3.03	-8.18	0.00	ENSMUSG00000092572
Serpina3f	12	0.560	-2.88	-7.37	0.01	ENSMUSG00000066363
Ccr9	9	0.090	-2.80	-6.95	0.03	ENSMUSG00000029530
Gm49391	13	5.780	-2.75	-6.74	0.00	ENSMUSG00000114432
Serpina3g	12	2.640	-2.75	-6.74	0.00	ENSMUSG00000041481
Mpl	4	9.910	-2.73	-6.61	0.00	ENSMUSG00000006389
Tmprss7	16	0.360	-2.53	-5.79	0.02	ENSMUSG00000033177
Amd2	10	4.840	-2.53	-5.78	0.00	ENSMUSG00000063953

Slc35d3	10	5.700	-2.50	-5.67	0.00	ENSMUSG00000050473
Crtam	9	0.290	-2.48	-5.58	0.01	ENSMUSG00000032021
Ripor2	13	1.260	-2.40	-5.28	0.00	ENSMUSG00000036006
Myct1	10	1.240	-2.38	-5.22	0.01	ENSMUSG00000046916
Traf3ip3	1	0.860	-2.38	-5.20	0.01	ENSMUSG00000037318
Fst	13	1.130	-2.35	-5.11	0.04	ENSMUSG00000021765
Slco4a1	2	4.060	-2.34	-5.06	0.00	ENSMUSG00000038963
Cdh17	4	0.180	-2.34	-5.06	0.04	ENSMUSG00000028217
Trpc6	9	0.630	-2.33	-5.03	0.01	ENSMUSG00000031997
Plcb1	2	0.200	-2.28	-4.86	0.03	ENSMUSG00000051177
Mef2c	13	2.360	-2.25	-4.77	0.00	ENSMUSG00000005583
Med12l	3	0.950	-2.25	-4.77	0.00	ENSMUSG00000056476
Robo3	9	2.050	-2.24	-4.72	0.00	ENSMUSG00000032128
F2rl3	8	3.790	-2.22	-4.66	0.00	ENSMUSG00000050147
Cth	3	1.700	-2.21	-4.64	0.01	ENSMUSG00000028179
Ptpn22	3	1.360	-2.17	-4.51	0.02	ENSMUSG00000027843
Gucy1a1	3	1.920	-2.16	-4.45	0.00	ENSMUSG00000033910
Arhgap15	2	0.840	-2.15	-4.43	0.01	ENSMUSG00000049744
Dnmt3b	2	3.730	-2.14	-4.42	0.00	ENSMUSG00000027478
Tmem163	1	0.710	-2.14	-4.42	0.02	ENSMUSG00000026347
Meis1	11	1.430	-2.14	-4.40	0.00	ENSMUSG00000020160
Ripor3	2	1.990	-2.13	-4.38	0.00	ENSMUSG00000074577
Plcg2	8	9.960	-2.07	-4.20	0.00	ENSMUSG00000034330
Dlg2	7	0.360	-2.05	-4.14	0.02	ENSMUSG00000052572
Slamf1	1	4.100	-2.04	-4.12	0.00	ENSMUSG00000015316
Cd69	6	0.720	-2.03	-4.08	0.04	ENSMUSG00000030156
Sla2	2	3.110	-2.02	-4.06	0.00	ENSMUSG00000027636
Cd226	18	1.290	-2.01	-4.02	0.01	ENSMUSG00000034028
Treml1	17	21.970	-2.00	-4.01	0.00	ENSMUSG00000023993
Draxin	4	0.590	-1.98	-3.95	0.02	ENSMUSG00000029005
Gbx2	1	0.550	-1.93	-3.81	0.05	ENSMUSG00000034486
44810	X	2.220	-1.92	-3.79	0.00	ENSMUSG00000050379
Cmah	13	0.520	-1.92	-3.77	0.01	ENSMUSG00000016756

Tnfsf14	17	4.510	-1.90	-3.72	0.01	ENSMUSG00000005824
Peg12	7	1.940	-1.88	-3.68	0.02	ENSMUSG00000070526
Rab37	11	2.180	-1.87	-3.66	0.01	ENSMUSG00000020732
Gp1bb	16	21.440	-1.86	-3.64	0.00	ENSMUSG00000050761
F5	1	3.720	-1.86	-3.63	0.00	ENSMUSG00000026579
Mmrn1	6	6.550	-1.86	-3.63	0.00	ENSMUSG00000054641
Capn3	2	0.720	-1.83	-3.55	0.05	ENSMUSG00000079110
Hhex	19	3.860	-1.82	-3.53	0.01	ENSMUSG00000024986
Gp5	16	22.080	-1.80	-3.49	0.00	ENSMUSG00000047953
Gmfg	7	5.900	-1.80	-3.48	0.01	ENSMUSG00000060791
Fli1	9	8.690	-1.79	-3.46	0.00	ENSMUSG00000016087
Prkca	11	4.140	-1.76	-3.38	0.00	ENSMUSG00000050965
Prodh	16	1.720	-1.75	-3.37	0.02	ENSMUSG00000003526
Fmnl1	11	4.810	-1.74	-3.34	0.00	ENSMUSG00000055805
Bahcc1	11	2.230	-1.73	-3.31	0.00	ENSMUSG00000039741
P2rx1	11	10.910	-1.72	-3.29	0.00	ENSMUSG00000020787
Rbpms2	9	10.140	-1.72	-3.28	0.00	ENSMUSG00000032387
Serpinb2	1	5.940	-1.72	-3.28	0.01	ENSMUSG00000062345
Prelid2	18	9.180	-1.71	-3.28	0.02	ENSMUSG00000056671
Slc22a3	17	1.070	-1.71	-3.28	0.03	ENSMUSG00000023828
Dctd	8	4.890	-1.69	-3.23	0.01	ENSMUSG00000031562
mt-Nd3	MT	577.770	-1.69	-3.22	0.00	ENSMUSG00000064360
Ubash3a	17	1.710	-1.67	-3.18	0.01	ENSMUSG00000042345
Mfng	15	3.120	-1.66	-3.16	0.02	ENSMUSG00000018169
Kcna3	3	1.930	-1.65	-3.15	0.04	ENSMUSG00000047959
Ptprcap	19	4.410	-1.64	-3.11	0.02	ENSMUSG00000045826
Itgb3	11	86.020	-1.62	-3.06	0.00	ENSMUSG00000020689
Slc24a5	2	3.850	-1.61	-3.06	0.03	ENSMUSG00000035183
Mtss1l	8	3.500	-1.60	-3.04	0.00	ENSMUSG00000033763
Ccdc116	16	2.420	-1.60	-3.04	0.01	ENSMUSG00000022768
Rasal3	17	3.530	-1.60	-3.04	0.01	ENSMUSG00000052142
Nlrp6	7	2.240	-1.60	-3.02	0.01	ENSMUSG00000038745
Ccdc85c	12	4.820	-1.59	-3.01	0.00	ENSMUSG00000084883

Gp1ba	11	14.750	-1.57	-2.98	0.00	ENSMUSG00000050675
Gp9	6	37.980	-1.55	-2.92	0.00	ENSMUSG00000030054
Clec1b	6	18.500	-1.55	-2.92	0.01	ENSMUSG00000030159
Mrap	16	10.450	-1.54	-2.91	0.02	ENSMUSG00000039956
Dok2	14	11.250	-1.54	-2.90	0.00	ENSMUSG00000022102
Rasgrp2	19	5.500	-1.53	-2.89	0.00	ENSMUSG00000032946
Gp6	7	1.510	-1.52	-2.86	0.04	ENSMUSG00000078810
Rpp40	13	1.480	-1.52	-2.86	0.04	ENSMUSG00000021418
Adgrg3	8	3.100	-1.51	-2.85	0.04	ENSMUSG00000060470
Zfp991	4	9.090	-1.51	-2.84	0.01	ENSMUSG00000067916
Lrrc32	7	2.910	-1.50	-2.82	0.01	ENSMUSG00000090958
Gucy1b1	3	3.300	-1.46	-2.75	0.02	ENSMUSG00000028005
Kcnk6	7	4.170	-1.45	-2.74	0.01	ENSMUSG00000046410
Rps6ka6	X	5.290	-1.44	-2.72	0.01	ENSMUSG00000025665
Pde5a	3	3.340	-1.44	-2.71	0.01	ENSMUSG00000053965
Sla	15	9.930	-1.43	-2.70	0.00	ENSMUSG00000022372
Ppbp	5	20.660	-1.42	-2.68	0.00	ENSMUSG00000029372
Ankrd13b	11	4.780	-1.42	-2.68	0.01	ENSMUSG00000037907
Unc119	11	18.560	-1.41	-2.66	0.00	ENSMUSG00000002058
Tnik	3	1.630	-1.41	-2.65	0.01	ENSMUSG00000027692
Impa2	18	2.620	-1.40	-2.64	0.02	ENSMUSG00000024525
Stk26	X	1.910	-1.40	-2.64	0.05	ENSMUSG00000031112
Klhl6	16	7.440	-1.38	-2.61	0.01	ENSMUSG00000043008
Rnf219	14	1.980	-1.38	-2.61	0.05	ENSMUSG00000022120
Bmi1	2	6.930	-1.37	-2.58	0.01	ENSMUSG00000026739
Cdk6	5	7.360	-1.36	-2.57	0.00	ENSMUSG00000040274
Itgal	7	3.600	-1.36	-2.57	0.01	ENSMUSG00000030830
Agap2	10	1.990	-1.35	-2.55	0.02	ENSMUSG00000025422
Spns2	11	16.280	-1.35	-2.54	0.00	ENSMUSG00000040447
Sms	X	11.510	-1.35	-2.54	0.00	ENSMUSG00000071708
Lat	7	10.940	-1.34	-2.54	0.03	ENSMUSG00000030742
Ptger3	3	13.960	-1.34	-2.53	0.00	ENSMUSG00000040016
Mrvi1	7	9.920	-1.32	-2.50	0.00	ENSMUSG00000005611

Smyd5	6	13.740	-1.30	-2.47	0.00	ENSMUSG00000033706
Ydjc	16	4.730	-1.30	-2.47	0.02	ENSMUSG00000041774
Lrmp	6	28.090	-1.30	-2.46	0.00	ENSMUSG00000030263
Slc25a13	6	6.950	-1.27	-2.41	0.01	ENSMUSG00000015112
Atp2a3	11	24.700	-1.26	-2.40	0.00	ENSMUSG00000020788
Shmt1	11	8.160	-1.26	-2.40	0.02	ENSMUSG00000020534
Kcnab2	4	4.560	-1.26	-2.39	0.01	ENSMUSG00000028931
Rasa3	8	14.330	-1.24	-2.35	0.00	ENSMUSG00000031453
Hmga1b	11	154.650	-1.22	-2.33	0.00	ENSMUSG00000078249
Lin28b	10	13.290	-1.21	-2.32	0.00	ENSMUSG00000063804
Thbs1	2	154.090	-1.21	-2.31	0.00	ENSMUSG00000040152
Pdk1	2	24.630	-1.20	-2.30	0.00	ENSMUSG00000006494
Alox12	11	16.560	-1.20	-2.29	0.00	ENSMUSG00000000320
Mylk	16	3.600	-1.19	-2.29	0.01	ENSMUSG00000022836
Suclg2	6	12.310	-1.19	-2.28	0.00	ENSMUSG00000061838
Rrp15	1	25.070	-1.19	-2.28	0.01	ENSMUSG00000001305
F2rl2	13	7.810	-1.18	-2.27	0.02	ENSMUSG00000021675
Eno1	4	178.420	-1.17	-2.25	0.00	ENSMUSG00000063524
Riox1	12	11.030	-1.16	-2.24	0.02	ENSMUSG00000046791
Bzw2	12	29.370	-1.15	-2.22	0.00	ENSMUSG00000020547
Psd4	2	3.580	-1.15	-2.22	0.01	ENSMUSG00000026979
Zfp992	4	4.730	-1.15	-2.22	0.02	ENSMUSG00000070605
Cdca7	2	25.420	-1.13	-2.19	0.00	ENSMUSG00000055612
Anp32a	9	34.980	-1.13	-2.18	0.00	ENSMUSG00000032249
Fastkd2	1	6.530	-1.13	-2.18	0.01	ENSMUSG00000025962
Igf2bp1	11	10.530	-1.11	-2.16	0.00	ENSMUSG00000013415
Gphn	12	7.320	-1.11	-2.16	0.02	ENSMUSG00000047454
Polr3g	13	6.840	-1.11	-2.16	0.05	ENSMUSG00000035834
Phgdh	3	75.230	-1.10	-2.14	0.00	ENSMUSG00000053398
Slc38a1	15	12.310	-1.10	-2.14	0.00	ENSMUSG00000023169
Trmt6	2	14.050	-1.10	-2.14	0.02	ENSMUSG00000037376
Cdca7l	12	6.430	-1.10	-2.14	0.03	ENSMUSG00000021175
Tpi1	6	212.280	-1.09	-2.12	0.00	ENSMUSG00000023456

Chd7	4	14.770	-1.07	-2.10	0.00	ENSMUSG00000041235
Vwf	6	13.250	-1.07	-2.10	0.00	ENSMUSG00000001930
Chst11	10	6.610	-1.06	-2.08	0.01	ENSMUSG00000034612
Enoph1	5	10.040	-1.06	-2.08	0.04	ENSMUSG00000029326
Bin2	15	37.920	-1.04	-2.06	0.00	ENSMUSG00000098112
Map4k1	7	5.730	-1.04	-2.06	0.04	ENSMUSG00000037337
Bend3	10	4.300	-1.03	-2.05	0.03	ENSMUSG00000038214
Ppif	14	47.020	-1.03	-2.04	0.00	ENSMUSG00000021868
Trmt61a	12	8.790	-1.03	-2.04	0.04	ENSMUSG00000060950
Elf1	14	20.450	-1.00	-2.00	0.00	ENSMUSG00000036461

Supplemental Table 6. Upregulated genes in *Dot1L-* KO ESRE cells that are unique to these mutant cells (n=635)

Gene Name	Chromosome	Max group mean	Log_2 fold change	Fold change	P-value	ENSEMBL
Clk4	13	61.020	2.17	4.50	0.00	ENSMUSG00000060639
Bcl2l11	18	4.720	2.17	4.50	0.00	ENSMUSG00000024544
Isca1	4	28.940	2.17	4.51	0.00	ENSMUSG00000028211
Dohh	17	10.260	2.17	4.51	0.00	ENSMUSG00000024042
Snca	15	35.750	2.17	4.51	0.00	ENSMUSG00000022598
Traf5	15	134.060	2.18	4.53	0.00	ENSMUSG00000071714
Eci2	2	1.350	2.18	4.53	0.00	ENSMUSG00000044033
Por	1	3.890	2.18	4.53	0.02	ENSMUSG00000026211
Hscb	9	2.680	2.18	4.53	0.04	ENSMUSG00000003309
Atg9a	6	6.720	2.18	4.54	0.00	ENSMUSG00000030287
Crtc1	10	3.220	2.18	4.54	0.01	ENSMUSG00000078439
Nipal3	2	17.900	2.19	4.55	0.00	ENSMUSG00000015647
Ypel5	8	1.240	2.19	4.56	0.01	ENSMUSG00000031778
Vamp2	5	0.410	2.19	4.56	0.02	ENSMUSG00000060961
Nbr1	9	13.890	2.19	4.57	0.00	ENSMUSG00000032323
Derl1	17	0.740	2.19	4.57	0.00	ENSMUSG00000038146
Dhx32	1	104.300	2.20	4.58	0.00	ENSMUSG00000016526
Supt4a	2	5.850	2.19	4.58	0.00	ENSMUSG00000027175
Gfap	14	4.090	2.20	4.58	0.00	ENSMUSG00000021822
Ctsa	16	3.270	2.20	4.58	0.02	ENSMUSG00000051065
Sidt2	4	3.520	2.20	4.58	0.03	ENSMUSG00000044254
Slc1a4	10	0.410	2.20	4.59	0.02	ENSMUSG00000009654
Fzr1	4	8.120	2.20	4.60	0.00	ENSMUSG00000028412
Syt14	3	4.310	2.20	4.61	0.00	ENSMUSG00000027875
Phf1	X	15.500	2.20	4.61	0.00	ENSMUSG00000031434
Stradb	X	42.560	2.21	4.61	0.01	ENSMUSG00000052854
Ogt	19	0.580	2.21	4.62	0.03	ENSMUSG00000061451
Tmem86b	11	4.030	2.21	4.62	0.03	ENSMUSG00000034427
Vps37b	11	0.090	2.21	4.62	0.05	ENSMUSG00000020542
Ticam1	2	10.380	2.21	4.63	0.01	ENSMUSG00000018459

Mafg	2	0.990	2.21	4.64	0.04	ENSMUSG00000048186
Atg2a	19	15.060	2.22	4.65	0.00	ENSMUSG00000039126
Itpkc	9	39.770	2.22	4.66	0.02	ENSMUSG00000032068
Grap2	13	0.450	2.22	4.66	0.02	ENSMUSG00000049115
Cln8	11	5.320	2.22	4.67	0.00	ENSMUSG00000018427
Ern1	16	0.480	2.23	4.68	0.01	ENSMUSG00000018830
Zbtb7b	10	0.120	2.23	4.68	0.04	ENSMUSG00000019852
Dop1b	3	1.240	2.23	4.69	0.01	ENSMUSG00000028088
44623	17	0.660	2.23	4.69	0.02	ENSMUSG00000056492
Cdk12	1	22.700	2.23	4.70	0.00	ENSMUSG00000026043
Smdt1	2	0.970	2.23	4.70	0.01	ENSMUSG00000034226
Tubb2a	3	2.540	2.23	4.70	0.02	ENSMUSG00000074604
Hectd4	14	1.490	2.24	4.72	0.00	ENSMUSG00000021991
Stx5a	2	0.620	2.24	4.72	0.02	ENSMUSG00000045838
Lsm10	5	3.810	2.24	4.72	0.02	ENSMUSG00000029189
Ube2l6	4	0.480	2.24	4.72	0.03	ENSMUSG00000046694
Bet1l	9	1.240	2.24	4.73	0.01	ENSMUSG00000046186
Serpinb6a	1	6.930	2.24	4.74	0.00	ENSMUSG00000039323
Osbpl9	15	0.290	2.25	4.75	0.03	ENSMUSG00000022469
Abhd2	14	0.430	2.25	4.76	0.01	ENSMUSG00000022096
Bag3	14	1.350	2.25	4.76	0.03	ENSMUSG00000021835
Fam126a	11	552.190	2.25	4.77	0.00	ENSMUSG00000015837
Creb3l1	4	0.190	2.25	4.77	0.02	ENSMUSG00000028369
Pde3b	13	1.540	2.25	4.77	0.04	ENSMUSG00000069266
Carhsp1	7	5.500	2.26	4.78	0.00	ENSMUSG00000110040
Klf11	4	5.620	2.26	4.78	0.01	ENSMUSG00000028364
Tbc1d17	5	1.020	2.26	4.78	0.04	ENSMUSG00000047963
Rnf169	13	2.670	2.26	4.78	0.05	ENSMUSG00000021456
Auh	1	4.950	2.26	4.79	0.02	ENSMUSG00000026638
Crebl2	11	2.260	2.26	4.79	0.03	ENSMUSG00000020469
Litaf	13	7.720	2.26	4.80	0.00	ENSMUSG00000056749
Dusp16	1	3.710	2.26	4.80	0.00	ENSMUSG00000026482
Ccpg1	5	0.330	2.27	4.81	0.05	ENSMUSG00000040584

Cd82	10	7.830	2.27	4.82	0.00	ENSMUSG00000055053
Nrip3	1	15.210	2.27	4.82	0.00	ENSMUSG00000042684
Padi2	14	5.320	2.27	4.82	0.00	ENSMUSG00000021950
Atf5	8	1.460	2.27	4.82	0.01	ENSMUSG00000074063
Crim1	3	0.250	2.27	4.83	0.05	ENSMUSG00000046317
1110032A03Rik	11	6.580	2.28	4.85	0.01	ENSMUSG00000020758
Snx29	17	7.110	2.28	4.85	0.01	ENSMUSG00000024371
Dnajb2	7	6.480	2.28	4.86	0.00	ENSMUSG00000006205
Akt1s1	7	1.200	2.28	4.86	0.01	ENSMUSG00000030787
Riok3	14	6.020	2.28	4.86	0.01	ENSMUSG00000033060
Plgrkt	4	1.810	2.28	4.86	0.03	ENSMUSG00000062157
Retreg2	4	2.580	2.28	4.86	0.03	ENSMUSG00000028841
Agpat4	11	2.520	2.28	4.86	0.04	ENSMUSG00000020427
Gm340	7	256.680	2.28	4.87	0.00	ENSMUSG00000042675
Foxo4	7	2.090	2.28	4.87	0.01	ENSMUSG00000038763
Zfand3	1	75.270	2.28	4.87	0.03	ENSMUSG00000005681
Ube2f	9	119.700	2.29	4.89	0.00	ENSMUSG00000032300
Acbd4	14	1.140	2.29	4.90	0.02	ENSMUSG00000035121
Pabpc1	16	4.310	2.30	4.91	0.00	ENSMUSG00000022508
Erfe	18	14.950	2.30	4.92	0.00	ENSMUSG00000024529
Ndrg1	X	9.610	2.30	4.92	0.00	ENSMUSG00000051159
Cnppd1	1	1.590	2.30	4.92	0.05	ENSMUSG00000026278
Usp3	1	8.470	2.31	4.95	0.00	ENSMUSG00000039377
Bnip3	11	0.490	2.31	4.95	0.00	ENSMUSG00000020866
Iscu	7	6.160	2.31	4.95	0.00	ENSMUSG00000040511
Sft2d2	2	2.000	2.31	4.95	0.03	ENSMUSG00000000308
Cd63	13	0.230	2.31	4.95	0.05	ENSMUSG00000060969
Gla	2	0.110	2.31	4.97	0.03	ENSMUSG00000050556
Pi4k2a	10	0.500	2.32	4.99	0.02	ENSMUSG00000020151
Ier2	9	0.680	2.32	4.99	0.03	ENSMUSG00000035274
Pigc	19	5.180	2.32	4.99	0.03	ENSMUSG00000025207
Dmxl2	3	4.040	2.32	5.00	0.00	ENSMUSG00000062515
Etfbkmt	12	224.690	2.32	5.01	0.00	ENSMUSG00000021190

Zfp691	11	3.010	2.32	5.01	0.00	ENSMUSG00000020363
Hdac6	16	1.190	2.32	5.01	0.00	ENSMUSG00000075122
Chd3	14	1.370	2.33	5.01	0.00	ENSMUSG00000022218
Praf2	3	3.920	2.32	5.01	0.01	ENSMUSG00000045934
Zfp58	11	6.090	2.32	5.01	0.02	ENSMUSG00000035775
Tmem189	19	10.920	2.33	5.02	0.00	ENSMUSG00000053279
Spag9	18	212.970	2.33	5.02	0.02	ENSMUSG00000061808
Plin3	12	0.350	2.33	5.04	0.05	ENSMUSG00000042734
Hbq1b	12	2.100	2.34	5.05	0.02	ENSMUSG00000042724
Akap13	7	1.590	2.34	5.07	0.00	ENSMUSG00000040428
Gvin1	7	1.200	2.34	5.07	0.03	ENSMUSG00000042246
Shisa5	3	0.860	2.34	5.08	0.05	ENSMUSG00000039865
Arrdc3	1	0.690	2.35	5.09	0.04	ENSMUSG00000049608
Map1lc3b	7	0.840	2.35	5.10	0.00	ENSMUSG00000063535
Slc25a36	10	1.770	2.35	5.11	0.01	ENSMUSG00000020183
Dclre1b	7	1.900	2.35	5.11	0.02	ENSMUSG00000054161
Tnip1	16	7.270	2.36	5.12	0.02	ENSMUSG00000060459
Fgfrl1	10	12.040	2.36	5.13	0.02	ENSMUSG00000020080
Bbx	15	0.140	2.36	5.14	0.05	ENSMUSG00000047591
Mtmr3	9	1.650	2.36	5.15	0.00	ENSMUSG00000033590
Bax	X	0.900	2.36	5.15	0.01	ENSMUSG00000046774
Ppp1r13b	3	1.230	2.36	5.15	0.01	ENSMUSG00000048332
Ccndbp1	2	0.770	2.37	5.16	0.04	ENSMUSG00000027188
Borcs6	5	0.060	2.37	5.16	0.05	ENSMUSG00000028883
Slc25a39	9	2.820	2.37	5.17	0.01	ENSMUSG00000062309
B3gnt3	19	2.950	2.37	5.17	0.01	ENSMUSG00000025194
Cox6b2	14	5.200	2.37	5.18	0.00	ENSMUSG00000068417
Cdc42bpg	X	0.910	2.38	5.20	0.01	ENSMUSG00000031380
Zfp36l1	2	0.450	2.38	5.20	0.02	ENSMUSG00000027420
Fez2	11	1.010	2.38	5.21	0.05	ENSMUSG00000000753
Bhlhe40	11	25.560	2.38	5.22	0.00	ENSMUSG00000020295
Ubald1	8	0.030	2.39	5.23	0.04	ENSMUSG00000074151
Enc1	7	218.740	2.39	5.26	0.00	ENSMUSG00000002083

Fos	15	0.920	2.40	5.28	0.04	ENSMUSG00000000530
H2-K1	13	72.060	2.41	5.30	0.00	ENSMUSG00000018102
Neu1	6	3.550	2.40	5.30	0.00	ENSMUSG00000019577
Tmc4	2	4.600	2.41	5.30	0.00	ENSMUSG00000039157
Siah1a	2	3.140	2.41	5.30	0.00	ENSMUSG00000017754
Cd9	8	0.660	2.41	5.30	0.04	ENSMUSG00000033249
Chst1	19	3.440	2.42	5.33	0.00	ENSMUSG00000041180
Zmat3	4	1.020	2.41	5.33	0.00	ENSMUSG00000078486
Samd8	3	20.880	2.41	5.33	0.03	ENSMUSG00000058952
Proser2	17	0.370	2.42	5.34	0.05	ENSMUSG00000024041
AI837181	5	38.810	2.42	5.35	0.00	ENSMUSG00000029201
Trpv2	7	0.630	2.42	5.35	0.03	ENSMUSG00000054662
St7	17	2.570	2.42	5.35	0.04	ENSMUSG00000059481
Cpa3	13	13.150	2.42	5.36	0.02	ENSMUSG00000034918
Sfi1	11	0.130	2.42	5.36	0.05	ENSMUSG00000020411
Hspa13	4	0.210	2.42	5.37	0.04	ENSMUSG00000095779
Egr1	14	0.150	2.43	5.38	0.01	ENSMUSG00000071531
Nedd4l	3	1.560	2.43	5.39	0.03	ENSMUSG00000027931
Me1	X	8.790	2.44	5.41	0.00	ENSMUSG00000034457
Dvl2	19	0.280	2.43	5.41	0.04	ENSMUSG00000067545
Atp8a2	6	0.110	2.44	5.42	0.03	ENSMUSG00000052861
Ier5	X	0.940	2.44	5.42	0.04	ENSMUSG00000067771
Gadd45g	17	3.320	2.44	5.43	0.00	ENSMUSG00000063011
Oaz1	10	0.130	2.44	5.43	0.05	ENSMUSG00000053219
Dyrk1b	6	0.390	2.45	5.45	0.01	ENSMUSG00000030134
Slco2a1	14	0.190	2.45	5.45	0.04	ENSMUSG00000021997
Hook2	16	1.220	2.45	5.46	0.01	ENSMUSG00000005958
Vegfa	7	58.650	2.45	5.47	0.02	ENSMUSG00000002992
Cln5	16	25.250	2.45	5.48	0.02	ENSMUSG00000022875
P2rx4	5	0.170	2.46	5.50	0.03	ENSMUSG00000029659
Itgav	13	7.550	2.46	5.51	0.00	ENSMUSG00000069206
Agpat3	6	2.800	2.46	5.51	0.02	ENSMUSG00000038167
Ctsl	2	0.310	2.46	5.51	0.02	ENSMUSG00000046338

Naga	17	0.170	2.46	5.51	0.03	ENSMUSG00000061126
Scpep1	6	6.260	2.47	5.52	0.00	ENSMUSG00000007655
Cd81	9	2.680	2.47	5.53	0.00	ENSMUSG00000036611
Afdn	10	0.240	2.47	5.54	0.01	ENSMUSG00000037855
Creg1	13	6.690	2.47	5.55	0.00	ENSMUSG00000058385
Atg9b	4	0.700	2.47	5.55	0.02	ENSMUSG00000012123
Epb41	11	0.250	2.48	5.57	0.02	ENSMUSG00000038560
Neurl3	3	51.600	2.48	5.58	0.00	ENSMUSG00000027869
Gabarap	12	2.970	2.48	5.58	0.02	ENSMUSG00000066438
Slc7a8	11	0.850	2.48	5.58	0.03	ENSMUSG00000013483
Nectin2	17	367.920	2.48	5.59	0.00	ENSMUSG00000023067
Cebpb	2	11.160	2.48	5.60	0.00	ENSMUSG00000006476
Itpk1	17	0.750	2.49	5.60	0.04	ENSMUSG00000037446
Rel	9	3.600	2.49	5.61	0.00	ENSMUSG00000032332
Rab11fip3	2	0.560	2.49	5.61	0.00	ENSMUSG00000027276
Fth1	19	0.430	2.50	5.65	0.01	ENSMUSG00000024901
Ppp2r5a	1	0.140	2.50	5.65	0.04	ENSMUSG00000091476
Susd6	7	15.390	2.50	5.66	0.01	ENSMUSG00000030739
Oser1	12	20.340	2.50	5.67	0.02	ENSMUSG00000021278
Hba-a1	11	0.340	2.51	5.69	0.03	ENSMUSG00000000782
Mapk1ip1	8	147.120	2.51	5.70	0.00	ENSMUSG00000052837
Aqp8	5	62.210	2.51	5.70	0.00	ENSMUSG00000037411
Phf21b	4	0.240	2.51	5.70	0.02	ENSMUSG00000073680
Notch1	1	0.170	2.51	5.71	0.02	ENSMUSG00000040612
1700030J22Rik	9	0.280	2.51	5.71	0.05	ENSMUSG00000074259
Pkp3	19	2.180	2.52	5.72	0.01	ENSMUSG00000038155
Ppp1r13l	19	0.640	2.52	5.74	0.01	ENSMUSG00000060224
Gsn	2	0.200	2.52	5.75	0.05	ENSMUSG00000033368
Rbpms	3	0.470	2.53	5.77	0.03	ENSMUSG00000033882
Pik3ip1	10	5.460	2.53	5.77	0.03	ENSMUSG00000035852
Gm37240	5	181.970	2.53	5.79	0.00	ENSMUSG00000118332
St14	7	0.830	2.53	5.79	0.00	ENSMUSG00000052353
Sfmbt2	6	0.160	2.53	5.79	0.05	ENSMUSG00000030222

Tmcc3	2	1.120	2.54	5.80	0.01	ENSMUSG00000027253
Rnf157	2	1.010	2.54	5.81	0.02	ENSMUSG00000044349
Ccdc92	1	2.040	2.54	5.82	0.00	ENSMUSG00000000817
Mras	12	0.890	2.54	5.82	0.01	ENSMUSG00000036523
Arhgef6	7	0.410	2.54	5.82	0.03	ENSMUSG00000025480
2900026A02Rik	1	6.120	2.54	5.83	0.02	ENSMUSG00000048126
Gpt2	11	1.120	2.54	5.83	0.02	ENSMUSG00000034227
Ralgds	13	3.320	2.55	5.84	0.00	ENSMUSG00000099583
H2-T23	12	0.620	2.55	5.85	0.01	ENSMUSG00000058070
Limk1	8	0.860	2.55	5.85	0.04	ENSMUSG00000033579
Fah	17	2.800	2.55	5.86	0.05	ENSMUSG00000024225
Appl2	6	0.980	2.55	5.87	0.00	ENSMUSG00000030256
Pdlim2	12	44.310	2.55	5.87	0.01	ENSMUSG00000020609
C77080	3	8.710	2.55	5.87	0.02	ENSMUSG00000027954
Mfge8	9	0.670	2.55	5.87	0.03	ENSMUSG00000032079
Stx3	X	1.660	2.56	5.89	0.00	ENSMUSG00000045010
Tnk1	16	1.370	2.56	5.90	0.02	ENSMUSG00000022868
Rap2b	6	17.820	2.57	5.93	0.00	ENSMUSG00000057137
Tbc1d2	10	2.980	2.57	5.94	0.00	ENSMUSG00000025400
Cd274	2	12.260	2.58	5.96	0.01	ENSMUSG00000026726
Gpx3	15	0.110	2.58	5.98	0.05	ENSMUSG00000022383
Bcar1	3	11.170	2.58	5.99	0.02	ENSMUSG00000040600
Ftl1	18	0.210	2.58	5.99	0.04	ENSMUSG00000002475
Dlk1	7	34.930	2.58	6.00	0.01	ENSMUSG00000040564
Fam83h	11	0.080	2.59	6.00	0.04	ENSMUSG00000020728
Plekha6	4	2.760	2.59	6.01	0.01	ENSMUSG00000006221
Dsp	13	6.020	2.59	6.03	0.01	ENSMUSG00000069306
Glt28d2	9	1.480	2.59	6.04	0.00	ENSMUSG00000037705
Maob	1	0.290	2.59	6.04	0.01	ENSMUSG00000044816
B430305J03Rik	5	3.990	2.60	6.05	0.00	ENSMUSG00000029166
Patj	13	0.540	2.60	6.08	0.01	ENSMUSG00000021362
Usp44	7	4.060	2.60	6.08	0.01	ENSMUSG00000001249
Rbp4	11	0.260	2.61	6.10	0.03	ENSMUSG00000020335

Dab2	19	0.490	2.61	6.10	0.04	ENSMUSG00000024992
Mageb16	3	0.040	2.61	6.10	0.05	ENSMUSG00000057123
Rab11fip4	1	2.800	2.61	6.13	0.00	ENSMUSG00000034220
Eps8l2	19	4.160	2.62	6.13	0.03	ENSMUSG00000025196
Rbp1	1	0.840	2.62	6.15	0.01	ENSMUSG00000026715
Plxnb2	17	0.120	2.62	6.15	0.04	ENSMUSG00000039518
Apobr	12	1.190	2.63	6.17	0.01	ENSMUSG00000021091
Icam1	9	0.680	2.63	6.17	0.05	ENSMUSG00000032357
Ntn4	12	0.270	2.63	6.19	0.03	ENSMUSG00000010529
Rhpn2	3	0.110	2.64	6.22	0.03	ENSMUSG00000027985
Pde8a	17	1.620	2.64	6.22	0.05	ENSMUSG00000046070
Rhou	7	55.690	2.65	6.28	0.00	ENSMUSG00000039236
Shroom3	14	2.650	2.65	6.28	0.02	ENSMUSG00000021751
Mdm2	9	0.170	2.65	6.28	0.02	ENSMUSG00000049723
F3	1	5.120	2.65	6.30	0.00	ENSMUSG00000026131
Trim47	5	5.990	2.66	6.31	0.00	ENSMUSG00000029135
Apcdd1	3	56.380	2.66	6.32	0.00	ENSMUSG00000063954
Phlda3	3	0.190	2.67	6.38	0.05	ENSMUSG00000059994
Susd2	8	0.700	2.68	6.39	0.00	ENSMUSG00000002190
Tmem144	2	6.250	2.68	6.39	0.00	ENSMUSG00000027070
Creb3l3	13	5.180	2.68	6.39	0.01	ENSMUSG00000021678
Chmp4c	9	0.420	2.67	6.39	0.02	ENSMUSG00000090693
Krt19	2	3.980	2.68	6.41	0.02	ENSMUSG00000037254
Tpsab1	19	0.270	2.68	6.42	0.05	ENSMUSG00000067279
Gprc5a	1	0.680	2.69	6.44	0.01	ENSMUSG00000038463
Nyap1	13	0.260	2.69	6.44	0.05	ENSMUSG00000021579
Espn	4	32.450	2.69	6.45	0.00	ENSMUSG00000078349
Clic6	7	0.620	2.69	6.45	0.00	ENSMUSG00000040488
Gm49496	X	0.190	2.69	6.45	0.02	ENSMUSG00000031202
Pdzd2	16	0.990	2.69	6.46	0.00	ENSMUSG00000039457
Depdc7	3	58.770	2.69	6.46	0.02	ENSMUSG00000028001
Hist1h2bk	15	0.180	2.70	6.48	0.02	ENSMUSG00000036218
Prrg4	13	15.290	2.70	6.52	0.00	ENSMUSG00000021701

44629	7	1.530	2.71	6.54	0.01	ENSMUSG00000002771
Tmem150a	18	16.180	2.71	6.56	0.00	ENSMUSG00000046727
Psrc1	4	1.840	2.71	6.56	0.00	ENSMUSG00000054362
Baiap2l1	4	0.160	2.71	6.56	0.04	ENSMUSG00000066196
Spsb2	11	37.020	2.72	6.59	0.00	ENSMUSG00000078851
Gm8797	11	66.670	2.72	6.59	0.00	ENSMUSG00000006575
Amot	11	0.570	2.72	6.59	0.01	ENSMUSG00000043419
Unc5b	16	0.060	2.72	6.60	0.05	ENSMUSG00000052316
Zbtb32	1	3.190	2.73	6.61	0.00	ENSMUSG00000026208
Kcnn1	15	9.950	2.73	6.63	0.00	ENSMUSG00000091694
Eps8	15	0.080	2.73	6.63	0.04	ENSMUSG00000050982
Gpr137c	11	0.220	2.73	6.63	0.05	ENSMUSG00000018919
Manba	11	0.380	2.73	6.64	0.02	ENSMUSG00000020810
Cdh1	12	0.790	2.74	6.68	0.01	ENSMUSG00000021253
Cldn7	7	2.100	2.74	6.68	0.01	ENSMUSG00000053175
Sema4a	13	2.630	2.74	6.69	0.01	ENSMUSG00000075031
Cds1	9	828.610	2.74	6.70	0.01	ENSMUSG00000032554
Crb3	5	2.220	2.74	6.70	0.01	ENSMUSG00000029370
Plxnb1	13	5.160	2.75	6.71	0.00	ENSMUSG00000069300
Gm10643	17	2.150	2.75	6.72	0.00	ENSMUSG00000043592
Pxdc1	16	1.020	2.75	6.75	0.00	ENSMUSG00000068196
Epb41l4b	15	0.200	2.76	6.76	0.03	ENSMUSG00000022455
Usp35	X	0.350	2.76	6.76	0.03	ENSMUSG00000079428
Selenop	17	4.590	2.76	6.78	0.01	ENSMUSG00000007034
Celsr1	17	0.050	2.76	6.78	0.04	ENSMUSG00000043939
Acsf2	X	1.060	2.76	6.78	0.04	ENSMUSG00000037010
Plekhh1	17	5.090	2.76	6.79	0.00	ENSMUSG00000048905
Rab11fip5	4	0.370	2.76	6.79	0.03	ENSMUSG00000070720
Clic5	19	0.080	2.77	6.80	0.04	ENSMUSG00000003555
Apoa1	12	1.110	2.77	6.80	0.05	ENSMUSG00000071177
Nav3	14	0.150	2.77	6.81	0.05	ENSMUSG00000022220
Mttp	12	1.560	2.77	6.82	0.02	ENSMUSG00000004791
Afp	7	0.140	2.78	6.85	0.04	ENSMUSG00000030543

Nhs	19	0.610	2.78	6.87	0.03	ENSMUSG00000050957
Dsg2	1	0.120	2.78	6.88	0.05	ENSMUSG00000010311
Gm28035	19	0.260	2.79	6.91	0.01	ENSMUSG00000041731
Fam135a	15	0.580	2.79	6.92	0.00	ENSMUSG00000023032
Kcnq1	10	0.120	2.79	6.93	0.01	ENSMUSG00000019899
Cyp2s1	9	4.760	2.80	6.96	0.00	ENSMUSG00000032340
Tex19.1	3	80.670	2.80	6.96	0.01	ENSMUSG00000033831
Als2cl	12	0.460	2.81	7.01	0.01	ENSMUSG00000048387
Smtnl2	19	0.680	2.82	7.04	0.02	ENSMUSG00000024922
Galnt3	4	94.740	2.82	7.05	0.00	ENSMUSG00000052684
Cep170b	9	0.320	2.82	7.05	0.01	ENSMUSG00000032011
Ankrd42	6	0.050	2.82	7.05	0.04	ENSMUSG00000008845
Gjb2	7	1.210	2.82	7.05	0.05	ENSMUSG00000074336
Arrdc4	X	0.840	2.82	7.06	0.04	ENSMUSG00000031382
Thnsl2	2	0.810	2.82	7.07	0.01	ENSMUSG00000068079
Apoa4	13	0.730	2.83	7.09	0.00	ENSMUSG00000051054
Arhgef5	X	13.240	2.83	7.10	0.00	ENSMUSG00000031375
Cldn4	13	0.440	2.83	7.12	0.04	ENSMUSG00000052485
Phlda2	11	0.190	2.83	7.13	0.04	ENSMUSG00000019368
Fcgrt	12	5.390	2.84	7.15	0.01	ENSMUSG00000066366
Rbm47	5	0.140	2.84	7.16	0.03	ENSMUSG00000029130
Rab15	18	0.160	2.84	7.16	0.04	ENSMUSG00000037346
Srgap1	13	9.720	2.84	7.18	0.00	ENSMUSG00000069305
Adamts1	5	1.020	2.85	7.22	0.00	ENSMUSG00000029468
Pramef12	15	1.070	2.85	7.23	0.04	ENSMUSG00000022149
Slc22a18	5	0.060	2.86	7.24	0.04	ENSMUSG00000040473
Zc3h12d	14	0.120	2.86	7.24	0.04	ENSMUSG00000057606
Sdc4	14	0.700	2.86	7.25	0.02	ENSMUSG00000072572
Cyp2d22	17	16.850	2.86	7.28	0.00	ENSMUSG00000023966
Nxf7	1	0.700	2.87	7.29	0.01	ENSMUSG00000043629
Lims2	13	0.250	2.87	7.30	0.04	ENSMUSG00000036110
Hand1	16	4.100	2.87	7.31	0.01	ENSMUSG00000022871
Rarres1	X	0.620	2.87	7.34	0.01	ENSMUSG00000015405

Gpc3	9	0.430	2.88	7.34	0.04	ENSMUSG00000044976
Spink1	15	1.950	2.88	7.35	0.00	ENSMUSG00000036944
Hist1h2an	13	3.850	2.88	7.39	0.01	ENSMUSG00000101972
Tjp3	17	1.450	2.89	7.41	0.02	ENSMUSG00000073418
Trim15	3	0.320	2.89	7.43	0.00	ENSMUSG00000032854
Gipc2	10	0.890	2.89	7.44	0.00	ENSMUSG00000019846
Vill	8	0.170	2.90	7.44	0.01	ENSMUSG00000053626
Abcb1b	2	0.330	2.90	7.46	0.03	ENSMUSG00000027560
Lamb2	7	0.840	2.90	7.47	0.00	ENSMUSG00000045777
Erbb3	4	0.630	2.90	7.47	0.02	ENSMUSG00000029074
Ryr2	8	0.740	2.91	7.49	0.01	ENSMUSG00000042812
Chdh	1	5.320	2.91	7.50	0.00	ENSMUSG00000026185
Esyt3	5	0.610	2.91	7.50	0.00	ENSMUSG00000029576
Arhgef16	1	0.660	2.91	7.50	0.00	ENSMUSG00000016194
Hes7	6	0.080	2.91	7.50	0.03	ENSMUSG00000030255
Sprr1a	15	0.240	2.91	7.51	0.02	ENSMUSG00000022357
H2-Q4	4	2.240	2.91	7.53	0.04	ENSMUSG00000001604
Hic1	2	0.440	2.91	7.54	0.00	ENSMUSG00000054580
Adamtsl4	X	0.930	2.92	7.55	0.02	ENSMUSG00000054034
Obsl1	1	0.160	2.93	7.60	0.00	ENSMUSG00000051985
Ap1m2	9	24.430	2.93	7.61	0.00	ENSMUSG00000064225
Slc4a4	13	3.370	2.93	7.61	0.00	ENSMUSG00000069270
Cyp11a1	17	16.790	2.93	7.62	0.00	ENSMUSG00000039481
Mb21d2	4	8.080	2.93	7.63	0.00	ENSMUSG00000023232
Pcsk9	3	4.290	2.94	7.66	0.01	ENSMUSG00000074489
Oit3	14	0.340	2.94	7.67	0.01	ENSMUSG00000022132
Morc4	6	0.100	2.95	7.71	0.01	ENSMUSG00000038022
Nrk	14	0.500	2.95	7.71	0.01	ENSMUSG00000079364
Tmem151a	11	1.770	2.95	7.73	0.00	ENSMUSG00000020847
Myo15b	4	0.120	2.95	7.74	0.02	ENSMUSG00000028919
Slc13a3	10	0.580	2.95	7.75	0.00	ENSMUSG00000059406
Bend7	19	0.190	2.95	7.75	0.05	ENSMUSG00000090369
Plet1	6	0.280	2.96	7.79	0.00	ENSMUSG00000041460

Arfgef3	10	1.000	2.96	7.79	0.03	ENSMUSG00000001155
Fmo5	14	0.350	2.97	7.83	0.02	ENSMUSG00000079391
Mgst2	5	0.300	2.97	7.83	0.03	ENSMUSG00000091897
Sel1l3	16	12.840	2.97	7.85	0.00	ENSMUSG00000022750
Hist1h4b	15	0.080	2.97	7.86	0.05	ENSMUSG00000044216
Gm49369	5	0.200	2.98	7.87	0.03	ENSMUSG00000029248
Stbd1	12	2.220	2.98	7.88	0.00	ENSMUSG00000021097
Fbp2	12	3.190	2.98	7.89	0.00	ENSMUSG00000021223
Irf6	7	0.230	2.98	7.90	0.04	ENSMUSG00000003273
Myl7	X	3.820	2.99	7.92	0.00	ENSMUSG00000068270
Rgl1	12	0.140	2.99	7.94	0.05	ENSMUSG00000021263
Abcb1a	10	3.950	2.99	7.96	0.01	ENSMUSG00000006345
Itgb4	5	0.620	2.99	7.96	0.01	ENSMUSG00000072612
C2	7	0.180	3.00	7.98	0.03	ENSMUSG00000025726
Lyve1	4	0.580	3.00	7.99	0.02	ENSMUSG00000028778
Lmo7	13	3.360	3.00	8.00	0.00	ENSMUSG00000069268
Ifnlr1	15	0.620	3.00	8.01	0.00	ENSMUSG00000091650
Cnksr1	7	0.500	3.01	8.05	0.00	ENSMUSG00000038292
Igfbp3	15	1.860	3.01	8.08	0.01	ENSMUSG00000023484
Alpk3	8	0.090	3.02	8.14	0.02	ENSMUSG00000064325
Apoa2	6	12.930	3.03	8.18	0.00	ENSMUSG00000030306
Neil2	14	0.150	3.05	8.25	0.04	ENSMUSG00000091472
Cited1	2	2.750	3.05	8.26	0.04	ENSMUSG00000046804
Bok	4	1.170	3.05	8.30	0.00	ENSMUSG00000028339
Ckmt1	13	0.110	3.06	8.33	0.01	ENSMUSG00000050876
Irx1	4	0.150	3.06	8.33	0.02	ENSMUSG00000037188
Ptprr	12	10.870	3.06	8.36	0.00	ENSMUSG00000071178
Tpbg	14	0.830	3.07	8.39	0.01	ENSMUSG00000061068
Sema4g	3	0.350	3.07	8.41	0.03	ENSMUSG00000068547
Mtmr11	11	11.810	3.07	8.43	0.00	ENSMUSG00000056895
Krt20	3	72.550	3.08	8.46	0.00	ENSMUSG00000064220
Ttr	13	2.050	3.08	8.47	0.01	ENSMUSG00000069308
Ttc9	11	1.270	3.08	8.47	0.02	ENSMUSG00000020681

Map3k9	X	0.340	3.09	8.49	0.02	ENSMUSG00000109493
Tmc7	11	1.130	3.09	8.51	0.00	ENSMUSG00000020486
Slc44a3	1	44.780	3.09	8.52	0.00	ENSMUSG00000044337
Gpr55	4	0.050	3.09	8.52	0.05	ENSMUSG00000028602
Cpm	7	0.030	3.09	8.53	0.04	ENSMUSG00000097789
Fam83e	13	1.530	3.09	8.54	0.00	ENSMUSG00000047246
Kng2	4	0.080	3.09	8.54	0.04	ENSMUSG00000028738
Hkdc1	5	0.110	3.10	8.56	0.02	ENSMUSG00000029675
8030474K03Rik	4	0.040	3.11	8.61	0.04	ENSMUSG00000050511
Pamr1	7	12.280	3.11	8.62	0.00	ENSMUSG00000002983
Abcc2	X	1.780	3.11	8.63	0.00	ENSMUSG00000079845
Bfsp1	15	6.290	3.12	8.72	0.00	ENSMUSG00000037280
Serpinf1	X	0.070	3.13	8.77	0.05	ENSMUSG00000047686
Acvrl1	X	1.920	3.13	8.78	0.02	ENSMUSG00000083695
Fam102a	6	0.280	3.14	8.80	0.04	ENSMUSG00000029868
Hsf4	8	3.670	3.14	8.84	0.00	ENSMUSG00000034472
Cfi	8	915.130	3.15	8.85	0.00	ENSMUSG00000005413
Ano9	11	0.050	3.15	8.88	0.05	ENSMUSG00000052921
Plg	13	0.030	3.16	8.97	0.04	ENSMUSG00000033063
Cdhr2	11	0.780	3.17	8.99	0.00	ENSMUSG00000048616
Npr1	7	1.530	3.17	9.00	0.01	ENSMUSG00000030790
Olfr1419	4	15.510	3.17	9.01	0.00	ENSMUSG00000073802
Dnah6	6	0.160	3.18	9.05	0.02	ENSMUSG00000055172
Gm14685	3	2.650	3.18	9.06	0.01	ENSMUSG00000074207
Rasgef1a	8	0.940	3.19	9.10	0.01	ENSMUSG00000031651
Lrrc63	2	3.600	3.19	9.14	0.00	ENSMUSG00000049044
Ephb3	7	0.290	3.19	9.15	0.03	ENSMUSG00000050063
Apoc2	8	0.780	3.20	9.16	0.04	ENSMUSG00000031980
Kng1	7	0.380	3.20	9.20	0.01	ENSMUSG00000090215
Plekhg6	17	0.090	3.20	9.21	0.02	ENSMUSG00000024176
Cyp4f39	3	60.770	3.20	9.22	0.02	ENSMUSG00000068893
Crybg2	7	0.030	3.20	9.22	0.04	ENSMUSG00000030621
Sp6	2	0.410	3.21	9.23	0.01	ENSMUSG00000015090

Plekhd1	7	0.100	3.21	9.23	0.03	ENSMUSG00000050425
Card14	3	3.990	3.21	9.24	0.00	ENSMUSG00000105827
Tulp1	3	4.960	3.21	9.24	0.03	ENSMUSG00000074445
Catspere2	6	0.110	3.21	9.27	0.04	ENSMUSG00000010796
Myh14	8	0.160	3.21	9.27	0.04	ENSMUSG00000050097
Amn	5	0.490	3.21	9.28	0.00	ENSMUSG00000029279
Tcf7	14	0.060	3.22	9.29	0.03	ENSMUSG00000071540
Trim69	16	1.320	3.23	9.36	0.00	ENSMUSG00000023341
Rbm46	13	4.310	3.23	9.36	0.00	ENSMUSG00000049539
Misp	9	0.560	3.23	9.36	0.00	ENSMUSG00000050578
Lrp4	13	0.820	3.24	9.45	0.02	ENSMUSG00000042379
Snhg11	1	5.740	3.24	9.48	0.00	ENSMUSG00000070942
Greb1	3	3.680	3.25	9.49	0.01	ENSMUSG00000078664
Syce1	13	0.050	3.25	9.49	0.03	ENSMUSG00000078302
Col6a3	15	1.950	3.26	9.59	0.00	ENSMUSG00000056487
Foxj1	1	3.620	3.26	9.61	0.00	ENSMUSG00000026639
Fa2h	11	0.350	3.27	9.63	0.00	ENSMUSG00000042529
Clps	9	0.360	3.28	9.75	0.00	ENSMUSG00000004098
Apob	15	0.430	3.29	9.78	0.02	ENSMUSG00000049152
Efna1	5	0.140	3.29	9.78	0.02	ENSMUSG00000029369
Apoa5	9	0.180	3.30	9.83	0.01	ENSMUSG00000070323
Ahsg	4	0.040	3.30	9.86	0.05	ENSMUSG00000002384
Cubn	3	0.380	3.30	9.88	0.01	ENSMUSG00000027832
Ppara	9	0.130	3.31	9.89	0.02	ENSMUSG00000032297
Eps8l3	12	0.130	3.31	9.91	0.03	ENSMUSG00000079012
Apoc1	4	0.360	3.31	9.92	0.01	ENSMUSG00000076436
Hpn	6	3.210	3.31	9.94	0.01	ENSMUSG00000009281
Pde6c	17	0.660	3.32	9.98	0.02	ENSMUSG00000023987
Cpn1	7	0.040	3.32	10.02	0.04	ENSMUSG00000074109
Serpinc1	9	1.840	3.33	10.04	0.01	ENSMUSG00000010064
Cdsn	3	96.620	3.34	10.12	0.01	ENSMUSG00000033860
Tinag	7	0.120	3.35	10.18	0.02	ENSMUSG00000095276
Gm266	4	17.560	3.35	10.21	0.00	ENSMUSG00000058183

Igfals	7	0.140	3.36	10.24	0.01	ENSMUSG00000031070
Acox2	15	1.330	3.37	10.33	0.00	ENSMUSG00000023046
Fcrl1	1	12.980	3.37	10.34	0.00	ENSMUSG00000026604
Lrp2	5	0.210	3.37	10.36	0.01	ENSMUSG00000029321
F2rl1	17	0.290	3.38	10.40	0.01	ENSMUSG00000091614
Itih2	7	0.200	3.39	10.47	0.00	ENSMUSG00000098132
Ppp1r3c	2	0.190	3.39	10.47	0.02	ENSMUSG00000037279
Lrrc14b	11	7.120	3.39	10.49	0.00	ENSMUSG00000110344
Fga	11	0.730	3.40	10.58	0.01	ENSMUSG00000040938
Pdzrn4	X	0.090	3.42	10.67	0.01	ENSMUSG00000071719
Grin2d	9	0.020	3.43	10.75	0.02	ENSMUSG00000062296
Tm4sf5	15	0.140	3.44	10.86	0.02	ENSMUSG00000093789
Bcl3	15	0.020	3.44	10.88	0.04	ENSMUSG00000079022
Trf	4	0.050	3.45	10.90	0.04	ENSMUSG00000070990
Rassf6	3	0.610	3.46	10.97	0.00	ENSMUSG00000074195
Tceal7	19	14.260	3.47	11.12	0.00	ENSMUSG00000024912
Slc44a4	8	0.080	3.48	11.14	0.05	ENSMUSG00000108900
Apln	1	9.070	3.48	11.15	0.00	ENSMUSG00000049866
4930539E08Rik	4	0.300	3.48	11.19	0.02	ENSMUSG00000034919
Serpina1d	17	0.090	3.50	11.34	0.02	ENSMUSG00000023914
Adcy4	5	129.160	3.51	11.36	0.00	ENSMUSG00000037428
Pgf	5	50.410	3.51	11.40	0.00	ENSMUSG00000029561
Mesp2	1	0.550	3.51	11.40	0.00	ENSMUSG00000041577
Optc	16	0.110	3.52	11.46	0.04	ENSMUSG00000022938
Slc4a8	14	1.670	3.52	11.50	0.00	ENSMUSG00000025557
Fgb	5	0.180	3.53	11.54	0.04	ENSMUSG00000091255
Ovol1	5	0.260	3.54	11.60	0.01	ENSMUSG00000062077
Apoc4	11	0.820	3.54	11.60	0.01	ENSMUSG00000020826
Asb11	2	0.280	3.54	11.65	0.01	ENSMUSG00000068452
Tcf15	15	0.920	3.55	11.70	0.00	ENSMUSG00000058057
Tmem171	13	3.310	3.55	11.75	0.00	ENSMUSG00000052180
Sec14l4	3	2.980	3.56	11.77	0.00	ENSMUSG00000036853
Serpina1a	9	0.150	3.56	11.83	0.02	ENSMUSG00000054978

Hrh4	1	0.330	3.58	11.92	0.00	ENSMUSG00000051590
C9	2	0.780	3.59	12.03	0.01	ENSMUSG00000038751
Colq	5	0.370	3.59	12.07	0.02	ENSMUSG00000105875
Slc39a2	8	0.110	3.61	12.18	0.03	ENSMUSG00000109523
1700019D03Rik	8	0.020	3.62	12.30	0.05	ENSMUSG00000031789
Slc17a2	15	0.100	3.62	12.30	0.05	ENSMUSG00000057346
Fetub	11	13.710	3.63	12.34	0.00	ENSMUSG00000039238
Wdr72	5	0.060	3.63	12.36	0.04	ENSMUSG00000029015
C4b	17	0.510	3.66	12.67	0.01	ENSMUSG00000073406
Dok5	X	0.230	3.67	12.70	0.00	ENSMUSG00000009670
Ttll10	12	0.600	3.67	12.74	0.00	ENSMUSG00000021185
Tcea3	6	0.100	3.67	12.76	0.02	ENSMUSG00000030359
Tceal5	11	0.030	3.68	12.78	0.03	ENSMUSG00000069911
Bglap3	12	0.050	3.68	12.81	0.03	ENSMUSG00000047415
Gm3558	14	1.560	3.69	12.91	0.00	ENSMUSG00000021768
4933411K16Rik	7	47.320	3.71	13.07	0.00	ENSMUSG00000034957
Ftcd	3	0.140	3.71	13.13	0.05	ENSMUSG00000027761
Gm2974	19	0.090	3.71	13.13	0.05	ENSMUSG00000025197
Gm17019	7	0.090	3.72	13.20	0.01	ENSMUSG00000058662
Clmn	X	0.060	3.72	13.21	0.04	ENSMUSG00000079534
Papln	4	0.320	3.73	13.26	0.04	ENSMUSG00000059816
Car11	13	19.200	3.73	13.28	0.00	ENSMUSG00000069307
Degs2	11	0.180	3.73	13.29	0.00	ENSMUSG00000051455
Ggt1	11	0.410	3.73	13.30	0.02	ENSMUSG00000046697
Slc28a1	2	7.790	3.78	13.72	0.00	ENSMUSG00000026831
Hcrtr1	1	4.730	3.78	13.74	0.00	ENSMUSG00000009772
Ccdc155	11	0.150	3.79	13.82	0.02	ENSMUSG00000057967
Prph	17	0.150	3.80	13.94	0.00	ENSMUSG00000033327
Hhip	10	0.410	3.81	14.03	0.00	ENSMUSG00000020090
Gm3739	8	4.220	3.82	14.16	0.00	ENSMUSG00000031860
Phgr1	16	1.420	3.83	14.20	0.00	ENSMUSG00000022821
Col15a1	15	1.100	3.83	14.25	0.01	ENSMUSG00000022371
Serpina1b	9	0.760	3.84	14.31	0.00	ENSMUSG00000050074

Mcpt4	X	0.410	3.85	14.39	0.00	ENSMUSG00000033361
Clca4a	5	0.200	3.85	14.44	0.01	ENSMUSG00000005373
Hist1h2bp	14	1.480	3.86	14.52	0.00	ENSMUSG00000072674
Ace	6	0.700	3.86	14.52	0.00	ENSMUSG00000068587
44808	13	0.090	3.87	14.60	0.00	ENSMUSG00000045034
Tnfrsf8	1	0.230	3.87	14.61	0.00	ENSMUSG00000041559
Tas1r2	8	0.390	3.88	14.76	0.01	ENSMUSG00000056313
Rtl3	7	0.060	3.88	14.76	0.03	ENSMUSG00000037463
Rnf138rt1	6	0.490	3.89	14.83	0.00	ENSMUSG00000055003
Trpv6	X	7.630	3.90	14.93	0.00	ENSMUSG00000051228
Rasd2	1	1.220	3.91	15.03	0.01	ENSMUSG00000009633
Arhgef15	7	10.740	3.92	15.10	0.00	ENSMUSG00000030703
Cntnap3	6	0.460	3.92	15.13	0.01	ENSMUSG00000030244
Adh1	17	14.410	3.92	15.18	0.01	ENSMUSG00000024365
Triml1	10	0.270	3.93	15.21	0.00	ENSMUSG00000025407
Agt	1	0.130	3.93	15.27	0.00	ENSMUSG00000048960
Sprr2a2	12	16.500	3.94	15.33	0.00	ENSMUSG00000034168
Ptgds	7	0.560	3.96	15.52	0.00	ENSMUSG00000006154
Mrgprb2	13	5.460	3.96	15.55	0.00	ENSMUSG00000069303
Sprr2a3	4	0.300	3.97	15.62	0.01	ENSMUSG00000111410
Asz1	13	5.380	3.99	15.91	0.00	ENSMUSG00000094248
Ces2b	1	0.190	4.00	16.02	0.00	ENSMUSG00000091017
3425401B19Rik	8	10.740	4.01	16.06	0.00	ENSMUSG00000038508
Hist1h1a	9	0.170	4.01	16.10	0.03	ENSMUSG00000032271
Esm1	17	5.760	4.02	16.18	0.00	ENSMUSG00000067235
Sprr2a1	12	32.960	4.02	16.21	0.00	ENSMUSG00000020641
Lamb3	7	0.870	4.03	16.34	0.00	ENSMUSG00000003379
Ugt3a2	15	1.880	4.04	16.49	0.00	ENSMUSG00000037579
Celf6	6	0.070	4.05	16.53	0.01	ENSMUSG00000030249
Oxct2a	13	0.070	4.06	16.69	0.02	ENSMUSG00000021620
Rarres2	17	0.230	4.06	16.72	0.00	ENSMUSG00000073407
Pgc	7	0.150	4.09	16.98	0.01	ENSMUSG00000030484
Mrgprx2	14	0.040	4.14	17.59	0.01	ENSMUSG00000021903

Slc38a3	13	0.050	4.14	17.60	0.01	ENSMUSG00000021319
Fgg	1	0.080	4.15	17.75	0.02	ENSMUSG00000039224
Gfy	14	0.050	4.15	17.80	0.01	ENSMUSG00000032925
Gm17657	11	1.830	4.19	18.22	0.00	ENSMUSG00000020620
Ovol2	3	0.410	4.22	18.67	0.00	ENSMUSG00000069041
Slc16a11	13	4.100	4.26	19.20	0.00	ENSMUSG00000071478
Trank1	7	0.720	4.27	19.24	0.00	ENSMUSG00000038540
Methig1	6	0.640	4.27	19.26	0.00	ENSMUSG00000029695
Clca4b	7	0.130	4.29	19.52	0.01	ENSMUSG00000005547
Ccdc194	2	0.150	4.32	19.97	0.01	ENSMUSG00000023391
Mep1a	1	5.320	4.32	20.00	0.00	ENSMUSG00000026072
Fam3b	1	6.530	4.41	21.23	0.00	ENSMUSG00000026073
Slc15a1	1	2.780	4.46	21.94	0.00	ENSMUSG00000050711
Speer4e	12	15.430	4.47	22.16	0.00	ENSMUSG00000021194
Trim54	6	92.150	4.48	22.32	0.00	ENSMUSG00000004655
Nos2	7	0.090	4.49	22.41	0.02	ENSMUSG00000058976
Duox2	13	0.080	4.55	23.35	0.01	ENSMUSG00000043986
Serpinb6c	17	0.290	4.55	23.48	0.01	ENSMUSG00000073399
Mcoln3	11	0.210	4.58	23.98	0.01	ENSMUSG00000025165
Kbtbd13	2	0.440	4.59	24.13	0.00	ENSMUSG00000084897
Ptk6	1	0.130	4.60	24.21	0.01	ENSMUSG00000067006
Cngb1	7	3.040	4.75	26.94	0.00	ENSMUSG00000060177
Slc26a5	11	0.510	4.81	27.96	0.00	ENSMUSG00000018554
Pzp	15	1.200	4.82	28.19	0.00	ENSMUSG00000044678
Gpr68	7	0.190	4.82	28.27	0.01	ENSMUSG00000033533
Aadac	4	2.700	4.89	29.63	0.00	ENSMUSG00000028389
Cyp2c23	6	0.420	4.93	30.42	0.00	ENSMUSG00000030364
Olfr69	6	2.270	5.03	32.64	0.00	ENSMUSG00000047420
Gm5640	4	0.090	5.12	34.77	0.05	ENSMUSG00000028635
Shisal2a	3	0.090	5.21	37.03	0.05	ENSMUSG00000063767
Enpp7	2	0.010	5.22	37.19	0.05	ENSMUSG00000050558
1700007K13Rik	5	0.480	5.22	37.32	0.00	ENSMUSG00000033805
Fgf18	3	0.200	5.29	39.22	0.05	ENSMUSG00000046259

Hgd	4	0.070	5.29	39.22	0.05	ENSMUSG00000029656
Mlxipl	6	0.030	5.29	39.22	0.05	ENSMUSG00000047228
Mgam	8	0.060	5.29	39.22	0.05	ENSMUSG00000055730
G0s2	15	0.070	5.29	39.22	0.05	ENSMUSG00000022579
Gys2	15	0.040	5.31	39.66	0.05	ENSMUSG00000022144
Cyp21a1	10	0.100	5.32	40.01	0.04	ENSMUSG00000020216
H2-Q10	14	0.030	5.32	40.03	0.04	ENSMUSG00000033644
Abcc9	7	0.030	5.34	40.38	0.05	ENSMUSG00000049848
Acot12	19	0.040	5.35	40.82	0.05	ENSMUSG00000024846
Gm6034	5	0.030	5.36	40.94	0.04	ENSMUSG00000074817
Sfrp4	10	0.070	5.36	40.94	0.04	ENSMUSG00000046922
D1Pas1	1	0.130	5.39	41.93	0.04	ENSMUSG00000005339
Aass	5	0.070	5.40	42.31	0.05	ENSMUSG00000055235
Dlx2	16	0.260	5.40	42.31	0.05	ENSMUSG00000110573
Usp17lc	2	21.730	5.47	44.28	0.00	ENSMUSG00000050896
Trim40	7	0.090	5.50	45.39	0.05	ENSMUSG00000078815
Sectm1a	17	0.120	5.51	45.64	0.04	ENSMUSG00000045027
Serpinb5	11	0.080	5.52	45.80	0.04	ENSMUSG00000072963
Clec2h	9	0.040	5.54	46.57	0.04	ENSMUSG00000032446
Edn2	1	0.170	5.55	46.82	0.04	ENSMUSG00000064272
S100a7a	16	0.180	5.56	47.04	0.03	ENSMUSG00000071552
Prokr2	11	2.530	5.61	48.72	0.00	ENSMUSG00000020467
Sprr2h	9	0.090	5.66	50.58	0.03	ENSMUSG00000041737
C8b	14	0.080	5.75	53.67	0.03	ENSMUSG00000021933
A2ml1	17	0.410	5.76	54.13	0.00	ENSMUSG00000050824
Ces2a	1	0.040	5.77	54.66	0.04	ENSMUSG00000025991
Gpihbp1	7	0.540	5.77	54.70	0.00	ENSMUSG00000009471
Gdnf	2	0.070	5.78	55.02	0.03	ENSMUSG00000013523
Jsrp1	4	0.090	5.99	63.68	0.03	ENSMUSG00000028940
Ceacam19	16	0.060	6.01	64.66	0.02	ENSMUSG00000062713
Cst6	2	0.150	6.02	64.90	0.02	ENSMUSG00000044103
Gpr6	6	0.150	6.09	68.28	0.02	ENSMUSG00000061762
Fcer1a	7	0.100	6.12	69.61	0.02	ENSMUSG00000057321

Wdr86	8	0.090	6.26	76.86	0.02	ENSMUSG00000046714
Gm5485	2	0.110	6.69	103.44	0.01	ENSMUSG00000027338
Cacng6	19	0.270	6.71	104.67	0.01	ENSMUSG00000024678
Prss22	16	0.140	6.75	107.32	0.01	ENSMUSG00000039830
Gm10447	1	0.090	6.76	108.26	0.01	ENSMUSG00000006014
Eomes	1	0.160	6.93	122.17	0.01	ENSMUSG00000038496
Tigit	1	0.180	6.98	125.81	0.01	ENSMUSG00000089675
Tmem45b	14	0.470	7.48	178.85	0.00	ENSMUSG00000094800
Gucy1b2	3	1.240	7.63	197.52	0.00	ENSMUSG00000054215
Cps1	7	0.190	7.98	252.47	0.00	ENSMUSG00000066197
Hes2	17	0.370	8.45	348.82	0.00	ENSMUSG00000092511

Supplemental Table 7. Downregulated genes in *Dot1L*-KO ESRE cells that are unique to these mutant cells (n=177

Name	Chromosome	Max group mean	Log_2 fold change	Fold change	P-value	ENSEMBL
Ctsg	14	1.560	-7.91	-241.28	0.00	ENSMUSG00000040314
Prl3b1	13	1.120	-7.51	-182.64	0.00	ENSMUSG00000038891
Prl7d1	13	0.310	-7.45	-174.74	0.00	ENSMUSG00000021348
Prl2c3	13	0.830	-7.09	-136.61	0.01	ENSMUSG00000056457
Ctsq	13	0.620	-6.98	-126.46	0.01	ENSMUSG00000021439
Gm26920	7	0.290	-6.81	-112.36	0.01	ENSMUSG00000058447
Ctsj	13	2.850	-6.54	-93.07	0.00	ENSMUSG00000055298
Fnd3c2	X	0.160	-6.46	-87.92	0.01	ENSMUSG00000073012
Cdk5r2	1	0.210	-6.40	-84.25	0.02	ENSMUSG00000090071
Ear6	14	0.550	-6.38	-83.25	0.01	ENSMUSG00000062148
Gm49387	14	0.310	-6.35	-81.60	0.02	ENSMUSG00000115022
Ngfr	11	0.160	-6.33	-80.18	0.02	ENSMUSG00000000120
Scg3	9	0.200	-6.13	-70.01	0.03	ENSMUSG00000032181
Gm49776	16	0.720	-6.12	-69.38	0.02	ENSMUSG00000116925
Adam11	11	0.090	-6.04	-65.87	0.02	ENSMUSG00000020926
Prl2c5	13	0.400	-6.03	-65.22	0.02	ENSMUSG00000055360
Ctsr	13	0.300	-5.99	-63.67	0.02	ENSMUSG00000055679
Prl2c2	13	0.420	-5.95	-61.81	0.02	ENSMUSG00000079092
Hoxd10	2	0.230	-5.90	-59.83	0.03	ENSMUSG00000050368
Ntrk2	13	0.020	-5.80	-55.81	0.03	ENSMUSG00000055254
Cadps	14	0.040	-5.63	-49.69	0.04	ENSMUSG00000054423
Stra6	9	0.070	-5.63	-49.57	0.03	ENSMUSG00000032327
Oas1g	5	0.140	-5.50	-45.36	0.03	ENSMUSG00000066861
Caskin1	17	0.050	-5.44	-43.53	0.04	ENSMUSG00000033597
Gpm6a	8	0.090	-5.38	-41.51	0.05	ENSMUSG00000031517
Hoxa9	6	0.300	-5.32	-40.08	0.00	ENSMUSG00000038227
Lbx1	19	0.110	-5.30	-39.48	0.05	ENSMUSG00000025216
Fgf7	2	0.080	-5.23	-37.44	0.05	ENSMUSG00000027208
Trem1	17	0.280	-5.15	-35.48	0.00	ENSMUSG00000042265
Pkib	10	0.040	-5.13	-35.12	0.05	ENSMUSG00000019876

Epx	11	0.080	-5.05	-33.10	0.05	ENSMUSG00000052234
Spata9	13	0.170	-5.05	-33.08	0.05	ENSMUSG00000021590
Stfa1	16	1.180	-4.40	-21.13	0.00	ENSMUSG00000071562
Clec12a	6	0.720	-4.37	-20.68	0.00	ENSMUSG00000053063
Brinp1	4	0.150	-4.33	-20.10	0.01	ENSMUSG00000028351
Snap25	2	0.200	-3.97	-15.70	0.03	ENSMUSG00000027273
Rbfox3	11	0.070	-3.88	-14.71	0.04	ENSMUSG00000025576
Ghrh	2	0.560	-3.84	-14.34	0.03	ENSMUSG00000027643
Elane	10	0.520	-3.71	-13.12	0.01	ENSMUSG00000020125
Klhl4	X	0.080	-3.65	-12.57	0.03	ENSMUSG00000025597
Hoxc10	15	0.790	-3.63	-12.38	0.02	ENSMUSG00000022484
Ms4a3	19	0.690	-3.62	-12.33	0.01	ENSMUSG00000024681
Snap91	9	0.100	-3.49	-11.22	0.04	ENSMUSG00000033419
Gm4724	2	0.870	-3.44	-10.87	0.00	ENSMUSG00000078897
Hoxa3	6	0.090	-3.37	-10.33	0.05	ENSMUSG00000079560
Gpr174	X	0.280	-3.29	-9.80	0.00	ENSMUSG00000073008
Zfp981	4	0.120	-3.28	-9.72	0.02	ENSMUSG00000056300
Ccm2l	2	0.330	-3.25	-9.50	0.01	ENSMUSG00000027474
Prss35	9	0.720	-3.21	-9.26	0.01	ENSMUSG00000033491
Scn3a	2	0.050	-3.09	-8.50	0.04	ENSMUSG00000057182
Pdzph1	17	0.080	-3.06	-8.36	0.03	ENSMUSG00000024227
Pou3f3	1	0.080	-3.05	-8.30	0.04	ENSMUSG00000045515
Zfhx4	3	0.090	-3.04	-8.20	0.02	ENSMUSG00000025255
Gm26965	13	0.360	-3.00	-7.99	0.04	ENSMUSG00000097565
Dpysl4	7	0.430	-2.85	-7.21	0.04	ENSMUSG00000025478
Csf3r	4	0.970	-2.85	-7.19	0.00	ENSMUSG00000028859
Stx1b	7	0.140	-2.84	-7.14	0.05	ENSMUSG00000030806
Sell	1	0.390	-2.81	-7.03	0.00	ENSMUSG00000026581
Crmp1	5	1.080	-2.80	-6.94	0.01	ENSMUSG00000029121
Has2	15	0.130	-2.78	-6.88	0.03	ENSMUSG00000022367
Ebf3	7	0.140	-2.75	-6.73	0.05	ENSMUSG00000010476
Hoxa7	6	0.140	-2.72	-6.61	0.04	ENSMUSG00000038236
Kcnk12	17	0.360	-2.72	-6.60	0.02	ENSMUSG00000050138

Clec5a	6	0.220	-2.70	-6.50	0.02	ENSMUSG00000029915
Ebi3	17	1.530	-2.66	-6.33	0.00	ENSMUSG00000003206
Olfm4	14	0.810	-2.64	-6.25	0.00	ENSMUSG00000022026
P2ry12	3	0.190	-2.62	-6.17	0.04	ENSMUSG00000036353
Gm10131	8	5.610	-2.58	-5.99	0.01	ENSMUSG00000063412
Phactr1	13	0.090	-2.55	-5.84	0.05	ENSMUSG00000054728
Cd52	4	3.490	-2.52	-5.72	0.01	ENSMUSG00000000682
Asxl3	18	0.060	-2.50	-5.66	0.03	ENSMUSG00000045215
Nefm	14	0.280	-2.50	-5.66	0.04	ENSMUSG00000022054
Rapsn	2	0.440	-2.49	-5.60	0.04	ENSMUSG00000002104
Atp8b4	2	0.550	-2.46	-5.50	0.01	ENSMUSG00000060131
S100a8	3	9.990	-2.40	-5.28	0.00	ENSMUSG00000056054
Gm14325	2	1.120	-2.40	-5.27	0.01	ENSMUSG00000095362
Ticam2	18	0.150	-2.39	-5.25	0.05	ENSMUSG00000056130
Tifab	13	0.440	-2.33	-5.03	0.02	ENSMUSG00000049625
A930033H	10	5.110	-2.29	-4.89	0.00	ENSMUSG00000090622
BC100530	16	3.050	-2.28	-4.86	0.01	ENSMUSG00000071561
Hp	8	4.980	-2.27	-4.81	0.00	ENSMUSG00000031722
Rnf17	14	0.700	-2.26	-4.79	0.01	ENSMUSG00000000365
Ccr2	9	0.150	-2.25	-4.75	0.04	ENSMUSG00000049103
Dot1l	10	26.100	-2.22	-4.65	0.00	ENSMUSG00000061589
Mctp1	13	0.500	-2.17	-4.50	0.02	ENSMUSG00000021596
Rgs7bp	13	0.240	-2.16	-4.47	0.02	ENSMUSG00000021719
Gpr141	13	0.490	-2.15	-4.45	0.03	ENSMUSG00000053101
2210418O	2	1.220	-2.10	-4.29	0.01	ENSMUSG00000078894
Angpt1	15	1.310	-2.09	-4.26	0.00	ENSMUSG00000022309
Clec4a2	6	0.280	-2.08	-4.23	0.05	ENSMUSG00000030148
Sardh	2	0.840	-2.03	-4.07	0.02	ENSMUSG00000009614
Card9	2	1.140	-2.01	-4.03	0.03	ENSMUSG00000026928
mt-Atp8	MT	1481.130	-1.96	-3.89	0.00	ENSMUSG00000064356
Ftl1-ps1	13	213.590	-1.96	-3.88	0.00	ENSMUSG00000062382
Il16	7	0.250	-1.92	-3.78	0.04	ENSMUSG00000001741
Igsf6	7	1.560	-1.89	-3.70	0.03	ENSMUSG00000035004

Rhobtb3	13	2.700	-1.85	-3.60	0.00	ENSMUSG00000021589
Zfp982	4	1.220	-1.85	-3.60	0.05	ENSMUSG00000078496
Zfp820	17	0.520	-1.81	-3.50	0.05	ENSMUSG00000069743
Sash3	X	3.800	-1.78	-3.44	0.01	ENSMUSG00000031101
Zfp990	4	2.300	-1.77	-3.41	0.03	ENSMUSG00000078503
Lama1	17	6.620	-1.77	-3.41	0.03	ENSMUSG00000032796
Kcnq5	1	0.430	-1.69	-3.24	0.05	ENSMUSG00000028033
Zfp946	17	1.210	-1.69	-3.22	0.04	ENSMUSG00000071266
Mpeg1	19	4.200	-1.68	-3.21	0.01	ENSMUSG00000046805
S100a9	3	11.120	-1.63	-3.10	0.03	ENSMUSG00000056071
Srl	16	0.970	-1.62	-3.08	0.03	ENSMUSG00000022519
Gm13212	4	1.350	-1.61	-3.06	0.04	ENSMUSG00000078502
Ncf1	5	3.820	-1.55	-2.94	0.01	ENSMUSG00000015950
Zfp984	4	5.450	-1.52	-2.86	0.01	ENSMUSG00000078495
Emb	13	3.950	-1.50	-2.83	0.01	ENSMUSG00000021728
Nfam1	15	0.750	-1.48	-2.80	0.04	ENSMUSG00000058099
Limd2	11	6.000	-1.47	-2.78	0.00	ENSMUSG00000040699
Tbxas1	6	2.710	-1.47	-2.78	0.04	ENSMUSG00000029925
Gdf3	6	10.260	-1.46	-2.76	0.01	ENSMUSG00000030117
Srgap3	6	1.290	-1.43	-2.69	0.02	ENSMUSG00000030257
Nlrx1	9	1.450	-1.42	-2.68	0.05	ENSMUSG00000032109
Zbed3	13	3.950	-1.41	-2.65	0.05	ENSMUSG00000041995
Slc24a3	2	2.740	-1.39	-2.62	0.03	ENSMUSG00000063873
Arhgap30	1	3.940	-1.37	-2.59	0.01	ENSMUSG00000048865
Psmb10	8	12.270	-1.34	-2.53	0.01	ENSMUSG00000031897
Lyz2	10	10.500	-1.32	-2.50	0.03	ENSMUSG00000069516
Gm4631	2	2.070	-1.32	-2.50	0.05	ENSMUSG00000078899
Hgh1	15	8.060	-1.31	-2.49	0.02	ENSMUSG00000022554
Hmga1	17	97.720	-1.27	-2.41	0.00	ENSMUSG00000046711
Sh3bgrl	X	12.960	-1.23	-2.35	0.00	ENSMUSG00000031246
Ctbp2	7	5.630	-1.23	-2.34	0.01	ENSMUSG00000030970
Celf2	2	1.840	-1.23	-2.34	0.02	ENSMUSG00000002107
CT030661	17	3.280	-1.22	-2.33	0.04	ENSMUSG00000117284

Etv6	6	5.970	-1.22	-2.32	0.00	ENSMUSG00000030199
Wdfy4	14	1.760	-1.20	-2.30	0.02	ENSMUSG00000051506
Slc35g1	19	4.430	-1.20	-2.30	0.03	ENSMUSG00000044026
Zfp770	2	2.550	-1.20	-2.30	0.05	ENSMUSG00000040321
Sort1	3	2.760	-1.18	-2.27	0.02	ENSMUSG00000068747
Itpr1	6	2.220	-1.16	-2.24	0.02	ENSMUSG00000030102
Naf1	8	10.930	-1.16	-2.23	0.01	ENSMUSG00000014907
Nars2	7	3.860	-1.15	-2.23	0.03	ENSMUSG00000018995
Lmo4	3	9.050	-1.14	-2.21	0.02	ENSMUSG00000028266
Cpne7	8	21.840	-1.13	-2.19	0.01	ENSMUSG00000034796
Hbb-bh1	7	224.390	-1.13	-2.18	0.00	ENSMUSG00000052217
Prdm11	2	2.000	-1.12	-2.18	0.01	ENSMUSG00000075028
Pla2g4a	1	5.300	-1.12	-2.18	0.03	ENSMUSG00000056220
Eef1e1	13	12.510	-1.12	-2.17	0.01	ENSMUSG00000001707
Zfp593	4	7.560	-1.11	-2.16	0.04	ENSMUSG00000028840
Sssca1_2	19	30.720	-1.10	-2.15	0.03	ENSMUSG00000079478
Kbtbd8	6	3.050	-1.11	-2.15	0.05	ENSMUSG00000030031
Hacd1	2	12.700	-1.09	-2.13	0.02	ENSMUSG00000063275
Tsfm	10	9.010	-1.09	-2.13	0.03	ENSMUSG00000040521
Isoc1	18	11.600	-1.08	-2.12	0.03	ENSMUSG00000024601
Pip4k2b	11	9.740	-1.08	-2.11	0.00	ENSMUSG00000018547
Coro1a	7	20.260	-1.08	-2.11	0.02	ENSMUSG00000030707
Twf2	9	9.510	-1.08	-2.11	0.03	ENSMUSG00000023277
Timm8a1	X	33.090	-1.06	-2.09	0.01	ENSMUSG00000048007
Slc19a1	10	7.770	-1.07	-2.09	0.02	ENSMUSG00000001436
Cox10	11	7.360	-1.06	-2.09	0.03	ENSMUSG00000042148
Ube2e1	14	16.460	-1.06	-2.09	0.05	ENSMUSG00000021774
Rrp9	9	32.600	-1.06	-2.08	0.00	ENSMUSG00000041506
mt-Nd2	MT	839.290	-1.05	-2.08	0.01	ENSMUSG00000064345
Egln1	8	24.510	-1.05	-2.07	0.00	ENSMUSG00000031987
Ddx10	9	13.210	-1.05	-2.07	0.02	ENSMUSG00000053289
Tmed5	5	3.370	-1.05	-2.07	0.04	ENSMUSG00000063406
Vps13a	19	4.680	-1.05	-2.06	0.00	ENSMUSG00000046230

Npm1	11	491.580	-1.04	-2.06	0.01	ENSMUSG00000057113
Ptpn4	1	1.680	-1.04	-2.06	0.04	ENSMUSG00000026384
Ttc27	17	9.110	-1.03	-2.05	0.02	ENSMUSG00000024078
Mapre2	18	7.900	-1.03	-2.04	0.00	ENSMUSG00000024277
Setdb1	3	21.830	-1.02	-2.03	0.00	ENSMUSG00000015697
Tomm5	4	54.680	-1.02	-2.03	0.01	ENSMUSG00000078713
Heatr1	13	16.950	-1.01	-2.02	0.00	ENSMUSG00000050244
Dcaf4	12	31.610	-1.02	-2.02	0.00	ENSMUSG00000021222
Fam49b	15	7.970	-1.02	-2.02	0.02	ENSMUSG00000022378
Psen2	1	11.900	-1.02	-2.02	0.05	ENSMUSG00000010609
Dock8	19	9.220	-1.01	-2.01	0.01	ENSMUSG00000052085
Gtf2h2	13	8.440	-1.00	-2.01	0.05	ENSMUSG00000021639
Thumpd1	7	22.820	-1.00	-2.00	0.01	ENSMUSG00000030942
Pnpt1	11	16.810	-1.00	-2.00	0.02	ENSMUSG00000020464
Slc7a6	8	8.700	-1.00	-2.00	0.02	ENSMUSG00000031904

Supplemental Table 8. Upregulated genes in *Dot1L-MM* ESRE cells in common with *Dot1L-KO* cells (n=756)

Gene Name	Chromosome	Max group mean	Log_2 fold change	Fold change	P-value	ENSEMBL
Aqp1	6	0.000	4.47	22.19	0.00	ENSMUSG00000004655
Tmtc1	6	0.000	2.92	7.55	0.00	ENSMUSG00000030306
Paqr9	9	0.000	2.98	7.90	0.00	ENSMUSG00000064225
Rsad2	12	0.000	4.78	27.51	0.00	ENSMUSG00000020641
Chga	12	0.000	4.61	24.41	0.00	ENSMUSG00000021194
Isg20	7	0.000	2.50	5.65	0.00	ENSMUSG00000039236
Ptpn14	1	0.000	2.95	7.73	0.00	ENSMUSG00000026604
Il1rl2	1	0.000	3.49	11.23	0.00	ENSMUSG00000070942
Il1r1	1	0.000	3.76	13.50	0.00	ENSMUSG00000026072
Hist1h2bq	13	0.000	3.35	10.20	0.00	ENSMUSG00000069307
Hist3h2a	11	0.000	2.69	6.45	0.00	ENSMUSG00000078851
Vgf	5	0.000	3.20	9.19	0.00	ENSMUSG00000037428
Ackr3	1	0.000	2.64	6.24	0.00	ENSMUSG00000044337
Rtn4rl2	2	0.000	4.00	15.97	0.00	ENSMUSG00000050896
Jun	4	0.000	2.45	5.48	0.00	ENSMUSG00000052684
Dapk2	9	0.000	2.60	6.08	0.00	ENSMUSG00000032380
Hsd3b6	3	0.000	2.28	4.86	0.00	ENSMUSG00000027869
Serpine1	5	0.000	2.62	6.14	0.00	ENSMUSG00000037411
Prune2	19	0.000	2.24	4.74	0.00	ENSMUSG00000039126
Sowaha	11	0.000	1.97	3.93	0.00	ENSMUSG00000044352
Dst	1	0.000	2.38	5.20	0.00	ENSMUSG00000026131
AW011738	4	0.000	2.37	5.18	0.00	ENSMUSG00000078349
Klhl22	16	0.000	2.62	6.16	0.00	ENSMUSG00000022750
Hopx	5	0.000	2.35	5.08	0.00	ENSMUSG00000059325
Abca8b	11	0.000	3.39	10.48	0.00	ENSMUSG00000020620
Gm45716	11	0.000	3.12	8.71	0.00	ENSMUSG00000110344
Cebpa	7	0.000	2.69	6.47	0.00	ENSMUSG00000034957
Zfp874a	13	0.000	2.39	5.25	0.00	ENSMUSG00000069206
Tmem140	6	0.000	2.24	4.71	0.00	ENSMUSG00000057137
Col11a1	3	0.000	3.50	11.34	0.00	ENSMUSG00000027966

Ugdh	5	0.000	2.10	4.28	0.00	ENSMUSG00000029201
Oasl2	5	0.000	3.22	9.31	0.00	ENSMUSG00000029561
Plagl1	10	0.000	3.21	9.27	0.00	ENSMUSG00000019817
Masp1	16	0.000	3.82	14.10	0.00	ENSMUSG00000022887
Shroom4	X	0.000	2.60	6.04	0.00	ENSMUSG00000068270
Fstl1	16	0.000	3.16	8.95	0.00	ENSMUSG00000022816
Hist2h2aa1	3	0.000	2.73	6.63	0.00	ENSMUSG00000064220
Cd36	5	0.000	2.66	6.33	0.00	ENSMUSG00000002944
Ccdc92b	11	0.000	1.91	3.75	0.00	ENSMUSG00000069814
Col5a1	2	0.000	3.00	8.01	0.00	ENSMUSG00000026837
Snx21	2	0.000	1.83	3.55	0.00	ENSMUSG00000050373
Nyx	X	0.000	2.70	6.52	0.00	ENSMUSG00000051228
Rapgef4	2	0.000	2.74	6.68	0.00	ENSMUSG00000049044
Dusp8	7	0.000	1.97	3.91	0.00	ENSMUSG00000037887
Igfbp2	1	0.000	3.38	10.41	0.00	ENSMUSG00000039323
Acta2	19	0.000	3.53	11.58	0.00	ENSMUSG00000035783
Hist3h2ba	11	0.000	3.27	9.63	0.00	ENSMUSG00000056895
Mmel1	4	0.000	2.52	5.74	0.00	ENSMUSG00000058183
Prnp	2	0.000	1.62	3.08	0.00	ENSMUSG00000079037
Clgn	8	0.000	3.87	14.58	0.00	ENSMUSG00000002190
Rnasel	1	0.000	1.63	3.08	0.00	ENSMUSG00000066800
Jph2	2	0.000	3.61	12.25	0.00	ENSMUSG00000017817
Efemp1	11	0.000	4.67	25.43	0.00	ENSMUSG00000020467
Samd11	4	0.000	1.69	3.22	0.00	ENSMUSG00000096351
Slc44a1	4	0.000	1.70	3.26	0.00	ENSMUSG00000028412
Galnt6	15	0.000	2.58	5.99	0.00	ENSMUSG00000037280
Mettl7a1	15	0.000	1.58	2.99	0.00	ENSMUSG00000054619
Hist1h2bc	13	0.000	1.96	3.90	0.00	ENSMUSG00000018102
Rundc3a	11	0.000	2.27	4.81	0.00	ENSMUSG00000006575
Col8a1	16	0.000	3.86	14.51	0.00	ENSMUSG00000068196
Tns1	1	0.000	2.09	4.27	0.00	ENSMUSG00000055322
Irf2bpl	12	0.000	3.02	8.10	0.00	ENSMUSG00000034168
Rhbdf1	11	0.000	1.86	3.64	0.00	ENSMUSG00000020282

Zfp37	4	0.000	3.63	12.41	0.00	ENSMUSG00000028389
Mfhas1	8	0.000	2.44	5.41	0.00	ENSMUSG00000070056
Rsph9	17	0.000	2.68	6.41	0.00	ENSMUSG00000023966
Fbn1	2	0.000	3.02	8.09	0.00	ENSMUSG00000027204
Mboat2	12	0.000	2.30	4.92	0.00	ENSMUSG00000020646
Ypel4	2	0.000	1.85	3.60	0.00	ENSMUSG00000034059
Hist2h2aa2	3	0.000	2.55	5.87	0.00	ENSMUSG00000063954
Vwa5a	9	0.000	1.60	3.04	0.00	ENSMUSG00000023186
Relb	7	0.000	2.46	5.49	0.00	ENSMUSG00000002983
Itpr2	6	0.000	1.75	3.37	0.00	ENSMUSG00000030287
Csf2rb2	15	0.000	1.85	3.61	0.00	ENSMUSG00000071714
Myh11	16	0.000	4.05	16.52	0.00	ENSMUSG00000018830
Pld3	7	0.000	1.59	3.00	0.00	ENSMUSG00000003363
Prss23	7	0.000	3.14	8.79	0.00	ENSMUSG00000039405
Cystm1	18	0.000	2.01	4.03	0.00	ENSMUSG00000046727
Arl4c	1	0.000	2.39	5.25	0.00	ENSMUSG00000049866
Celf4	18	0.000	2.29	4.88	0.00	ENSMUSG00000024268
Tcp11l1	2	0.000	1.96	3.90	0.00	ENSMUSG00000027175
Ypel3	7	0.000	1.85	3.62	0.00	ENSMUSG00000042675
Lgmn	12	0.000	1.82	3.52	0.00	ENSMUSG00000021190
Timp2	11	0.000	2.60	6.07	0.00	ENSMUSG00000017466
Trim58	11	0.000	1.58	2.98	0.00	ENSMUSG00000037124
Lox	18	0.000	2.90	7.45	0.00	ENSMUSG00000024529
Vsir	10	0.000	2.05	4.13	0.00	ENSMUSG00000020101
Klk1b22	7	0.000	4.77	27.37	0.00	ENSMUSG00000060177
Ehd2	7	0.000	2.64	6.22	0.00	ENSMUSG00000074364
Col5a2	1	0.000	3.18	9.06	0.00	ENSMUSG00000026042
Suco	1	0.000	1.86	3.63	0.00	ENSMUSG00000040297
Khnyn	14	0.000	1.49	2.80	0.00	ENSMUSG00000047153
Stard10	7	0.000	1.81	3.51	0.00	ENSMUSG00000030688
Elmo1	13	0.000	1.82	3.52	0.00	ENSMUSG00000041112
Il1r2	1	0.000	3.25	9.51	0.00	ENSMUSG00000026073
Fam220a_1	5	0.000	1.85	3.60	0.00	ENSMUSG00000118332

Hmox1	8	0.000	2.25	4.75	0.00	ENSMUSG00000005413
Gdf15	8	0.000	2.93	7.64	0.00	ENSMUSG00000038508
Igfbp5	1	0.000	2.80	6.97	0.00	ENSMUSG00000026185
Lama2	10	0.000	4.53	23.14	0.00	ENSMUSG00000019899
Rnf128	X	0.000	1.53	2.89	0.00	ENSMUSG00000031438
Hist1h1c	13	0.000	1.77	3.41	0.00	ENSMUSG00000036181
Pla2r1	2	0.000	3.75	13.45	0.00	ENSMUSG00000054580
Bbc3	7	0.000	1.91	3.76	0.00	ENSMUSG00000002083
Btn1a1	13	0.000	1.62	3.07	0.00	ENSMUSG00000000706
Mad2l1bp	17	0.000	1.64	3.11	0.00	ENSMUSG00000034509
Bpgm	6	0.000	1.83	3.55	0.00	ENSMUSG00000038871
Gpc1	1	0.000	2.52	5.75	0.00	ENSMUSG00000034220
Klf6	13	0.000	1.47	2.77	0.00	ENSMUSG00000000078
Lpin1	12	0.000	1.37	2.59	0.00	ENSMUSG00000020593
Fosl2	5	0.000	2.18	4.53	0.00	ENSMUSG00000029135
Cdkn2b	4	0.000	2.45	5.45	0.00	ENSMUSG00000073802
Tagln	9	0.000	3.01	8.03	0.00	ENSMUSG00000032085
Mx2	16	0.000	3.25	9.49	0.00	ENSMUSG00000023341
Pard3	8	0.000	2.07	4.20	0.00	ENSMUSG00000025812
Cemip	7	0.000	3.40	10.57	0.00	ENSMUSG00000052353
Slc25a33	4	0.000	1.95	3.88	0.00	ENSMUSG00000028982
Thy1	9	0.000	4.32	20.01	0.00	ENSMUSG00000032011
Arid5b	10	0.000	2.30	4.91	0.00	ENSMUSG00000019947
Gch1	14	0.000	1.80	3.48	0.00	ENSMUSG00000037580
Mgll	6	0.000	1.48	2.79	0.00	ENSMUSG00000033174
Cx3cl1	8	0.000	3.14	8.81	0.00	ENSMUSG00000031778
Abtb1	6	0.000	1.41	2.66	0.00	ENSMUSG00000030083
Sqstm1	11	0.000	1.99	3.97	0.00	ENSMUSG00000015837
Abhd4	14	0.000	1.55	2.93	0.00	ENSMUSG00000040997
Mfsd12	10	0.000	1.47	2.78	0.00	ENSMUSG00000034854
Loxl2	14	0.000	2.75	6.72	0.00	ENSMUSG00000034205
Neo1	9	0.000	2.16	4.47	0.00	ENSMUSG00000032340
Ldlrad4	18	0.000	2.20	4.59	0.00	ENSMUSG00000024544

Svep1	4	0.000	3.78	13.71	0.00	ENSMUSG00000028369
Cd163	6	0.000	5.30	39.52	0.00	ENSMUSG00000008845
Bsdc1	4	0.000	1.64	3.13	0.00	ENSMUSG00000040859
Fabp4	3	0.000	2.85	7.20	0.00	ENSMUSG00000062515
Fmod	1	0.000	4.85	28.76	0.00	ENSMUSG00000041559
Tll1	8	0.000	4.13	17.46	0.00	ENSMUSG00000053626
Adrb2	18	0.000	1.69	3.24	0.00	ENSMUSG00000045730
Fam213a	14	0.000	1.46	2.75	0.00	ENSMUSG00000021792
Hmgcs2	3	0.000	2.72	6.60	0.00	ENSMUSG00000027875
Dstyk	1	0.000	1.48	2.80	0.00	ENSMUSG00000042046
Dcn	10	0.000	3.26	9.60	0.00	ENSMUSG00000019929
Gtpbp2	17	0.000	1.64	3.13	0.00	ENSMUSG00000023952
Pgm5	19	0.000	4.29	19.62	0.00	ENSMUSG00000041731
Cav1	6	0.000	2.73	6.64	0.00	ENSMUSG00000007655
Fosl1	19	0.000	2.38	5.19	0.00	ENSMUSG00000024912
Btnl10	11	0.000	2.10	4.30	0.00	ENSMUSG00000020490
Gm4070	7	0.000	1.48	2.78	0.00	ENSMUSG00000078606
Htra1	7	0.000	2.94	7.69	0.00	ENSMUSG00000006205
Ltbp4	7	0.000	3.53	11.58	0.00	ENSMUSG00000040488
Rph3al	11	0.000	2.74	6.70	0.00	ENSMUSG00000020847
Hsd11b1	1	0.000	3.97	15.64	0.00	ENSMUSG00000016194
Junb	8	0.000	1.82	3.53	0.00	ENSMUSG00000052837
Fn3k	11	0.000	2.11	4.31	0.00	ENSMUSG00000025175
Hlx	1	0.000	1.97	3.90	0.00	ENSMUSG00000039377
Tmem71	15	0.000	2.74	6.66	0.00	ENSMUSG00000036944
Medag	5	0.000	4.10	17.16	0.00	ENSMUSG00000029659
Hist1h2ac	13	0.000	2.81	7.03	0.00	ENSMUSG00000069270
Ppp2r5b	19	0.000	1.77	3.42	0.00	ENSMUSG00000024777
Prokr1	6	0.000	1.39	2.62	0.00	ENSMUSG00000049409
Pltp	2	0.000	3.06	8.36	0.00	ENSMUSG00000017754
Cdkn1b	6	0.000	1.48	2.79	0.00	ENSMUSG00000003031
Meioc	11	0.000	4.39	21.00	0.00	ENSMUSG00000051455
Lpin2	17	0.000	1.65	3.14	0.00	ENSMUSG00000024052

App	16	0.000	2.10	4.28	0.00	ENSMUSG00000022892
Slc7a11	3	0.000	1.62	3.07	0.00	ENSMUSG00000027737
Isg15	4	0.000	2.65	6.28	0.00	ENSMUSG00000035692
Wdr45	X	0.000	1.41	2.67	0.00	ENSMUSG00000039382
Sertad3	7	0.000	1.46	2.76	0.00	ENSMUSG00000055200
Gdpd3	7	0.000	3.60	12.13	0.00	ENSMUSG00000030703
Ccrl2	9	0.000	1.59	3.01	0.00	ENSMUSG00000043953
Zrsr1	11	0.000	1.40	2.64	0.00	ENSMUSG00000044068
Ccng2	5	0.000	1.19	2.28	0.00	ENSMUSG00000029385
Maged1	X	0.000	1.53	2.89	0.00	ENSMUSG00000025151
Ube2b	11	0.000	1.42	2.68	0.00	ENSMUSG00000020390
Oas3	5	0.000	3.48	11.16	0.00	ENSMUSG00000032661
Flnc	6	0.000	2.03	4.10	0.00	ENSMUSG00000068699
Trp53inp1	4	0.000	1.38	2.60	0.00	ENSMUSG00000028211
Abhd6	14	0.000	2.09	4.26	0.00	ENSMUSG00000025277
Crem	18	0.000	1.80	3.48	0.00	ENSMUSG00000063889
Ctsf	19	0.000	1.74	3.33	0.00	ENSMUSG00000083282
Reln	5	0.000	1.32	2.50	0.00	ENSMUSG00000042453
H1f0	15	0.000	1.25	2.38	0.00	ENSMUSG00000096210
Kdm5b	1	0.000	1.20	2.29	0.00	ENSMUSG00000042207
1700017B05Rik	9	0.000	1.80	3.48	0.00	ENSMUSG00000032300
Lhfp	3	0.000	3.19	9.14	0.00	ENSMUSG00000048332
Lgals3	14	0.000	1.74	3.35	0.00	ENSMUSG00000050335
Axl	7	0.000	2.50	5.66	0.00	ENSMUSG00000002602
Nuak2	1	0.000	2.43	5.39	0.00	ENSMUSG00000009772
Naa80	9	0.000	1.17	2.25	0.00	ENSMUSG00000079334
Smpdl3a	10	0.000	2.22	4.66	0.00	ENSMUSG00000019872
Gba2	4	0.000	1.27	2.41	0.00	ENSMUSG00000028467
Mmp2	8	0.000	1.66	3.17	0.00	ENSMUSG00000031740
H2-D1	17	0.000	1.51	2.85	0.00	ENSMUSG00000073411
Ccdc141	2	0.000	1.82	3.52	0.00	ENSMUSG00000044033
Col1a1	11	0.000	2.78	6.85	0.00	ENSMUSG00000001506
Ccdc9b	2	0.000	3.01	8.05	0.00	ENSMUSG00000045838

Sh3pxd2a	19	0.000	2.33	5.03	0.00	ENSMUSG00000053617
Btg2	1	0.000	1.50	2.84	0.00	ENSMUSG00000020423
Pbx4	8	0.000	2.97	7.85	0.00	ENSMUSG00000031860
Mxd1	6	0.000	1.52	2.88	0.00	ENSMUSG00000001156
Hist1h4i	13	0.000	1.71	3.27	0.00	ENSMUSG00000060639
Hist1h2br	13	0.000	3.33	10.05	0.00	ENSMUSG00000069303
Atg4a	X	0.000	1.18	2.27	0.00	ENSMUSG00000079418
Atf3	1	0.000	1.43	2.69	0.00	ENSMUSG00000026628
Sgms1	19	0.000	1.19	2.28	0.00	ENSMUSG00000040451
Ube2h	6	0.000	1.48	2.79	0.00	ENSMUSG00000039159
Nfil3	13	0.000	1.97	3.93	0.00	ENSMUSG00000056749
Ahnak	19	0.000	1.71	3.27	0.00	ENSMUSG00000069833
Stk32a	18	0.000	2.98	7.91	0.00	ENSMUSG00000039954
Slc16a10	10	0.000	1.53	2.89	0.00	ENSMUSG00000019838
Zswim4	8	0.000	1.35	2.55	0.00	ENSMUSG00000035671
S100a11	3	0.000	1.95	3.88	0.00	ENSMUSG00000027907
Ddit4	10	0.000	1.52	2.86	0.00	ENSMUSG00000020108
Mef2d	3	0.000	1.36	2.57	0.00	ENSMUSG00000001419
Atg14	14	0.000	1.29	2.45	0.00	ENSMUSG00000037526
Acot2	12	0.000	1.75	3.36	0.00	ENSMUSG00000021226
Eps8l1	7	0.000	4.23	18.81	0.00	ENSMUSG00000006154
Slc6a20a	9	0.000	2.71	6.55	0.00	ENSMUSG00000036814
Ankrd33b	15	0.000	2.24	4.74	0.00	ENSMUSG00000022237
Slfn14	11	0.000	1.50	2.83	0.00	ENSMUSG00000082101
Anxa8	14	0.000	2.65	6.27	0.00	ENSMUSG00000021950
Dnajb9	12	0.000	1.38	2.60	0.00	ENSMUSG00000014905
Col12a1	9	0.000	2.73	6.64	0.00	ENSMUSG00000032332
Zfp750	11	0.000	2.04	4.12	0.00	ENSMUSG00000039238
Nfkb2	19	0.000	1.34	2.53	0.00	ENSMUSG00000025225
Gstt2	10	0.000	1.87	3.64	0.00	ENSMUSG00000033318
Dnajb4	3	0.000	1.36	2.58	0.00	ENSMUSG00000028035
Itgb7	15	0.000	1.33	2.52	0.00	ENSMUSG00000001281
Pik3r1	13	0.000	1.27	2.42	0.00	ENSMUSG00000041417

Dusp1	17	0.000	1.43	2.69	0.00	ENSMUSG00000024190
Bcl6	16	0.000	1.89	3.70	0.00	ENSMUSG00000022508
Ccdc88a	11	0.000	1.28	2.44	0.00	ENSMUSG00000032740
Zfp773	7	0.000	2.43	5.40	0.00	ENSMUSG00000063535
Fbxl3	14	0.000	1.41	2.66	0.00	ENSMUSG00000022124
Zfp703	8	0.000	1.59	3.00	0.00	ENSMUSG00000085795
Kdm6b	11	0.000	1.39	2.63	0.00	ENSMUSG00000018476
Fhl1	X	0.000	1.28	2.43	0.00	ENSMUSG00000023092
P2rx7	5	0.000	2.58	5.97	0.00	ENSMUSG00000029468
1-Sep	7	0.000	1.21	2.31	0.00	ENSMUSG00000000486
Hist1h3d	13	0.000	2.74	6.70	0.00	ENSMUSG00000099583
Tmcc2	1	0.000	1.52	2.86	0.00	ENSMUSG00000042066
Trp53inp2	2	0.000	1.54	2.90	0.00	ENSMUSG00000038375
Jag1	2	0.000	2.78	6.85	0.00	ENSMUSG00000027276
Serinc2	4	0.000	1.93	3.81	0.00	ENSMUSG00000023232
Unc5cl	17	0.000	2.88	7.38	0.00	ENSMUSG00000043592
Ptgs2	1	0.000	2.08	4.23	0.00	ENSMUSG00000032487
Slit3	11	0.000	3.04	8.25	0.00	ENSMUSG00000056427
Per1	11	0.000	1.21	2.32	0.00	ENSMUSG00000020893
Tcp11l2	10	0.000	1.07	2.71	0.00	ENSMUSG00000020034
Hdac5	11	0.000	1.44	2.10	0.00	ENSMUSG00000008855
Nfic	10	0.000	1.57	2.97	0.00	ENSMUSG00000055053
Mmp27	9	0.000	4.24	18.93	0.00	ENSMUSG00000070323
Cdc42ep4	11	0.000	1.19	2.28	0.00	ENSMUSG00000041598
Dyrk3	1	0.000	1.52	2.88	0.00	ENSMUSG00000016526
Zfp36	7	0.000	1.67	3.18	0.00	ENSMUSG00000044786
Ugt8a	3	0.000	3.15	8.87	0.00	ENSMUSG00000032854
Mafk	5	0.000	1.31	2.47	0.00	ENSMUSG00000018143
Ahnak2	12	0.000	2.07	4.20	0.00	ENSMUSG00000072812
Mcam	9	0.000	3.28	9.74	0.00	ENSMUSG00000032135
Resf1	6	0.000	1.38	2.61	0.00	ENSMUSG00000032712
Bicra	7	0.000	1.01	2.01	0.00	ENSMUSG00000070808
Ildr2	1	0.000	3.44	10.83	0.00	ENSMUSG00000040612

Lhfpl2	13	0.000	2.06	4.18	0.00	ENSMUSG00000045312
Dglucy	12	0.000	3.07	8.39	0.00	ENSMUSG00000021185
Hist1h2ad	13	0.000	3.62	12.31	0.00	ENSMUSG00000071478
Prkab2	3	0.000	1.23	2.34	0.00	ENSMUSG00000038205
Rit1	3	0.000	1.15	2.23	0.00	ENSMUSG00000028057
Rab30	7	0.000	1.58	2.99	0.00	ENSMUSG00000030643
Rsrp1	4	0.000	1.36	2.56	0.00	ENSMUSG00000037266
Gcm2	13	0.000	3.33	10.05	0.00	ENSMUSG00000021362
Sfxn3	19	0.000	1.82	3.54	0.00	ENSMUSG00000025212
Postn	3	0.000	2.83	7.13	0.00	ENSMUSG00000027750
Mettl27	5	0.000	1.12	2.17	0.00	ENSMUSG00000040557
Phlda1	10	0.000	1.29	2.45	0.00	ENSMUSG00000020205
Col3a1	1	0.000	2.89	7.43	0.00	ENSMUSG00000026043
Mkrn1	6	0.000	1.48	2.79	0.00	ENSMUSG00000029922
Abcc3	11	0.000	2.24	4.73	0.00	ENSMUSG00000020865
Fbxo30	10	0.000	1.19	2.29	0.00	ENSMUSG00000047648
Vim	2	0.000	1.67	3.18	0.00	ENSMUSG00000026728
Slc43a2	11	0.000	1.12	2.18	0.00	ENSMUSG00000038178
Csf2rb	15	0.000	1.36	2.56	0.00	ENSMUSG00000071713
Gadd45a	6	0.000	1.49	2.81	0.00	ENSMUSG00000036390
Klhl25	7	0.000	1.30	2.47	0.00	ENSMUSG00000055652
Hist1h2ao	13	0.000	3.62	12.30	0.00	ENSMUSG00000094248
Bgn	X	0.000	3.02	8.09	0.00	ENSMUSG00000031375
Npr2	4	0.000	2.84	7.15	0.00	ENSMUSG00000028469
Ezh1	11	0.000	1.08	2.12	0.00	ENSMUSG00000006920
Smtnl1	2	0.000	1.95	3.86	0.00	ENSMUSG00000027077
Hr	14	0.000	2.67	6.36	0.00	ENSMUSG00000022096
Jund	8	0.000	1.52	2.87	0.00	ENSMUSG00000071076
Hyal1	9	0.000	1.87	3.65	0.00	ENSMUSG00000010051
Fam214b	4	0.000	1.34	2.53	0.00	ENSMUSG00000036002
Hbp1	12	0.000	1.19	2.28	0.00	ENSMUSG00000002996
Tnxb	17	0.000	3.31	9.92	0.00	ENSMUSG00000033327
Kirrel	3	0.000	2.10	4.28	0.00	ENSMUSG00000041734

Agrn	4	0.000	1.29	2.45	0.00	ENSMUSG00000041936
Tent5b	4	0.000	3.06	8.34	0.00	ENSMUSG00000046694
Xpo7	14	0.000	1.41	2.66	0.00	ENSMUSG00000022100
Lcp1	14	0.000	1.23	2.34	0.00	ENSMUSG00000021998
Ube2o	11	0.000	1.42	2.68	0.00	ENSMUSG00000020802
Cavin1	11	0.000	1.86	3.64	0.00	ENSMUSG00000004044
Fam53b	7	0.000	1.09	2.13	0.00	ENSMUSG00000030956
Ubap1	4	0.000	1.33	2.51	0.00	ENSMUSG00000028437
Ddit3_1	10	0.000	1.32	2.50	0.00	ENSMUSG00000025408
Btg1	10	0.000	1.11	2.15	0.00	ENSMUSG00000036478
Gstp3	19	0.000	1.68	3.20	0.00	ENSMUSG00000058216
Mxi1	19	0.000	1.29	2.44	0.00	ENSMUSG00000025025
Anxa1	19	0.000	1.81	3.50	0.00	ENSMUSG00000024659
Stk17b	1	0.000	1.10	2.14	0.00	ENSMUSG00000026094
Plk3	4	0.000	1.59	3.00	0.00	ENSMUSG00000028680
Ptpdc1	13	0.000	2.78	6.85	0.00	ENSMUSG00000038042
Rnh1	7	0.000	1.10	2.15	0.00	ENSMUSG00000038650
Serpina3n	12	0.000	2.99	7.96	0.00	ENSMUSG00000021091
Kcnh3	15	0.000	2.57	5.92	0.00	ENSMUSG00000037579
Hist2h2be	3	0.000	1.80	3.47	0.00	ENSMUSG00000068854
Klhl21	4	0.000	1.23	2.34	0.00	ENSMUSG00000073700
Tgfb3	12	0.000	3.25	9.55	0.00	ENSMUSG00000021253
Dnase1l1	X	0.000	1.26	2.39	0.00	ENSMUSG00000019088
Aldh1a1	19	0.000	1.58	3.00	0.00	ENSMUSG00000053279
Ptger4	15	0.000	1.62	3.07	0.00	ENSMUSG00000039942
Cdkn1c	7	0.000	1.97	3.92	0.00	ENSMUSG00000037664
Cd109	9	0.000	2.52	5.72	0.00	ENSMUSG00000046186
Tpm1	9	0.000	1.23	2.35	0.00	ENSMUSG00000032366
Thbs2	17	0.000	3.02	8.09	0.00	ENSMUSG00000023885
Pdzk1ip1	4	0.000	1.54	2.91	0.00	ENSMUSG00000028716
Fhdc1	3	0.000	1.28	2.42	0.00	ENSMUSG00000041842
Phospho1	11	0.000	1.35	2.55	0.00	ENSMUSG00000050860
Lef1	3	0.000	3.81	14.01	0.00	ENSMUSG00000027985

Arhgef19	4	0.000	3.85	14.46	0.00	ENSMUSG00000028919
Sesn2	4	0.000	1.46	2.75	0.00	ENSMUSG00000028893
Pnrc1	4	0.000	1.21	2.32	0.00	ENSMUSG00000040128
Hmg20b	10	0.000	1.01	2.02	0.00	ENSMUSG00000020232
Trove2	1	0.000	1.01	2.02	0.00	ENSMUSG00000018199
Fn1	1	0.000	2.32	4.99	0.00	ENSMUSG00000026193
Tbx6	7	0.000	1.65	3.13	0.00	ENSMUSG00000030699
Zfp945	17	0.000	1.78	3.44	0.00	ENSMUSG00000059142
Nrtn	17	0.000	2.12	4.34	0.00	ENSMUSG00000039481
Ccdc80	16	0.000	2.46	5.49	0.00	ENSMUSG00000022665
Prrg3	X	0.000	3.35	10.21	0.00	ENSMUSG00000033361
Fars2	13	0.000	1.22	2.33	0.00	ENSMUSG00000021420
Fam117a	11	0.000	1.04	2.06	0.00	ENSMUSG00000038893
Aqp9	9	0.000	1.66	3.16	0.00	ENSMUSG00000032204
Slc48a1	15	0.000	1.17	2.25	0.00	ENSMUSG00000081534
Sspn	6	0.000	4.04	16.46	0.00	ENSMUSG00000030255
Htatip2	7	0.000	1.11	2.16	0.00	ENSMUSG00000039745
Trim34b	7	0.000	3.75	13.48	0.00	ENSMUSG00000090215
Il4ra	7	0.000	1.29	2.45	0.00	ENSMUSG00000030748
Plac9b	14	0.000	3.77	13.66	0.00	ENSMUSG00000072674
Pnp2	14	0.000	2.25	4.75	0.00	ENSMUSG00000068417
Crebrf	17	0.000	1.13	2.20	0.00	ENSMUSG00000048249
Gatm	2	0.000	2.26	4.79	0.00	ENSMUSG00000027199
Cdkn1a	17	0.000	1.49	2.80	0.00	ENSMUSG00000023067
Hbq1a	11	0.000	1.76	3.38	0.00	ENSMUSG00000020295
Ifngr2	16	0.000	1.27	2.41	0.00	ENSMUSG00000022965
Tbcel	9	0.000	1.26	2.39	0.00	ENSMUSG00000037287
Pcmtd2	2	0.000	1.04	2.05	0.00	ENSMUSG00000027589
Gpcpd1	2	0.000	1.38	2.61	0.00	ENSMUSG00000027346
Cpt1c	7	0.001	1.62	3.08	0.00	ENSMUSG00000007783
Pink1	4	0.001	1.19	2.29	0.00	ENSMUSG00000028756
Angptl2	2	0.001	2.79	6.94	0.00	ENSMUSG00000004105
Hist1h2be	13	0.001	2.65	6.28	0.00	ENSMUSG00000047246

Foxf1	8	0.001	3.38	10.38	0.00	ENSMUSG00000042812
Bmp1	14	0.001	2.27	4.82	0.00	ENSMUSG00000022098
Raet1e	10	0.001	3.74	13.33	0.00	ENSMUSG00000053219
Ctsd	7	0.001	1.28	2.42	0.00	ENSMUSG00000007891
Gabarapl2	8	0.001	1.09	2.12	0.00	ENSMUSG00000031950
Trim56	5	0.001	1.02	2.03	0.00	ENSMUSG00000043279
Sertad1	7	0.001	1.32	2.50	0.00	ENSMUSG00000008384
Tmem67	4	0.001	1.62	3.06	0.00	ENSMUSG00000049488
Fam180a	6	0.001	3.22	9.30	0.00	ENSMUSG00000047420
Otub2	12	0.001	1.05	2.06	0.00	ENSMUSG00000021203
Ifitm10	7	0.001	2.59	6.01	0.00	ENSMUSG00000045777
Clk1	1	0.001	1.12	2.18	0.00	ENSMUSG00000026034
Prr15l	11	0.001	2.04	4.12	0.00	ENSMUSG00000047040
Agtr1a	13	0.001	2.65	6.28	0.00	ENSMUSG00000049115
Abca1	4	0.001	2.23	4.68	0.00	ENSMUSG00000015243
Srxn1	2	0.001	1.14	2.20	0.00	ENSMUSG00000032802
Serinc1	10	0.001	1.03	2.04	0.00	ENSMUSG00000019877
Evi5	5	0.001	1.04	2.06	0.00	ENSMUSG00000011831
Apol11b	15	0.001	1.62	3.08	0.00	ENSMUSG00000091694
Dennd4a	9	0.001	1.23	2.34	0.00	ENSMUSG00000053641
Ptx3	3	0.001	3.54	11.64	0.00	ENSMUSG00000027832
Stag3	5	0.001	1.44	2.72	0.00	ENSMUSG00000036928
Fbxo9	9	0.001	1.07	2.09	0.00	ENSMUSG00000001366
Me3	7	0.001	4.59	24.03	0.00	ENSMUSG00000030621
Tac2	10	0.001	2.45	5.46	0.00	ENSMUSG00000025400
Calcoco1	15	0.001	1.05	2.07	0.00	ENSMUSG00000023055
Tmem131l	3	0.001	1.18	2.27	0.00	ENSMUSG00000033767
Sstr5	17	0.001	4.29	19.63	0.00	ENSMUSG00000050824
Ak1	2	0.001	2.22	4.67	0.00	ENSMUSG00000026817
Hist1h2bf	13	0.001	2.71	6.56	0.00	ENSMUSG00000069268
Trafd1	5	0.001	1.14	2.20	0.00	ENSMUSG00000042726
Msln	17	0.001	2.40	5.29	0.00	ENSMUSG00000063011
Slc30a1	1	0.001	1.19	2.28	0.00	ENSMUSG00000037434

Inhbb	1	0.001	2.89	7.42	0.00	ENSMUSG00000037035
Ypel2	11	0.001	1.49	2.81	0.00	ENSMUSG00000018427
Ttc12	9	0.001	1.47	2.77	0.00	ENSMUSG00000040219
Nfkbia	12	0.001	1.47	2.78	0.00	ENSMUSG00000021025
Dusp13	14	0.001	2.67	6.35	0.00	ENSMUSG00000021768
1810013L24Rik	16	0.001	1.14	2.20	0.00	ENSMUSG00000022507
Gstm5	3	0.001	1.09	2.13	0.00	ENSMUSG00000004032
Hspb7	4	0.001	2.72	6.57	0.00	ENSMUSG00000006221
Fat1	8	0.001	2.19	4.55	0.00	ENSMUSG00000070047
Tmem98	11	0.001	2.08	4.23	0.00	ENSMUSG00000035413
Plvap	8	0.001	1.79	3.46	0.00	ENSMUSG00000034845
Plk2	13	0.001	1.63	3.08	0.00	ENSMUSG00000021701
Naprt	15	0.001	1.89	3.71	0.00	ENSMUSG00000022574
Glrx	13	0.001	1.46	2.76	0.00	ENSMUSG00000021591
Fads3	19	0.001	1.67	3.19	0.00	ENSMUSG00000024664
Nsmf	2	0.001	1.50	2.82	0.00	ENSMUSG00000006476
Ybx2	11	0.001	3.88	14.75	0.00	ENSMUSG00000018554
Hist1h3i	13	0.001	3.00	8.00	0.00	ENSMUSG00000101972
Slc25a51	4	0.001	1.05	2.07	0.00	ENSMUSG00000045973
Rerg	6	0.001	3.54	11.62	0.00	ENSMUSG00000030222
Gm14226	2	0.001	3.40	10.56	0.00	ENSMUSG00000084897
Fam213b	4	0.001	2.38	5.20	0.00	ENSMUSG00000029059
Gfpt2	11	0.001	1.82	3.54	0.00	ENSMUSG00000020363
Cpeb4	11	0.001	1.19	2.29	0.00	ENSMUSG00000020300
Hist1h1d	13	0.001	2.12	4.36	0.00	ENSMUSG00000052565
Slc10a6	5	0.001	3.91	15.02	0.00	ENSMUSG00000029321
Camk2d	3	0.001	1.73	3.32	0.00	ENSMUSG00000053819
Brdt	5	0.001	2.59	6.01	0.00	ENSMUSG00000029279
Plau	14	0.001	2.03	4.09	0.00	ENSMUSG00000021822
Hecw1	13	0.002	1.38	2.60	0.00	ENSMUSG00000021301
Pla2g16	19	0.002	1.03	2.05	0.00	ENSMUSG00000060675
Hspg2	4	0.002	2.09	4.27	0.00	ENSMUSG00000028763
Cyp2b10	7	0.002	2.65	6.30	0.00	ENSMUSG00000030483

Cyp2a5	7	0.002	4.78	27.54	0.00	ENSMUSG00000005547
Tob1	11	0.002	1.19	2.28	0.00	ENSMUSG00000037573
Tcim	8	0.002	4.10	17.19	0.00	ENSMUSG00000056313
Prnd	2	0.002	8.18	289.20	0.00	ENSMUSG00000027338
Smtn	11	0.002	1.33	2.52	0.00	ENSMUSG00000020439
Lpp	16	0.002	2.08	4.24	0.00	ENSMUSG00000033306
Pim1	17	0.002	1.21	2.31	0.00	ENSMUSG00000024014
Mrgprf	7	0.002	3.74	13.36	0.00	ENSMUSG00000031070
Ets1	9	0.002	1.82	3.54	0.00	ENSMUSG00000032035
Cldn10	14	0.002	2.88	7.38	0.00	ENSMUSG00000022132
Serpina3m	12	0.002	4.28	19.46	0.00	ENSMUSG00000079012
Col22a1	15	0.002	4.98	31.63	0.00	ENSMUSG00000079022
Hectd2	19	0.002	1.56	2.95	0.00	ENSMUSG00000041180
Dstn	2	0.002	1.24	2.36	0.00	ENSMUSG00000015932
St5	7	0.002	1.10	2.14	0.00	ENSMUSG00000031024
Tent5c	3	0.002	1.16	2.23	0.00	ENSMUSG00000044468
Aif1l	2	0.002	2.47	5.53	0.00	ENSMUSG00000001864
Nrbp2	15	0.002	1.60	3.02	0.00	ENSMUSG00000075590
Rhbdf2	11	0.002	1.17	2.25	0.00	ENSMUSG00000020806
Ankrd34b	13	0.002	3.81	13.99	0.00	ENSMUSG00000045034
Tmem43	6	0.002	1.00	2.00	0.00	ENSMUSG00000030095
Fbxo27	7	0.002	4.87	29.28	0.00	ENSMUSG00000037463
Hist2h3c2	3	0.002	2.33	5.04	0.00	ENSMUSG00000081058
Hist1h2bj	13	0.002	2.49	5.62	0.00	ENSMUSG00000069300
Cacna1g	11	0.002	2.08	4.24	0.00	ENSMUSG00000020866
Dedd2	7	0.002	1.04	2.05	0.00	ENSMUSG00000054499
Map3k19	1	0.002	2.68	6.41	0.00	ENSMUSG00000051590
Nodal	10	0.002	2.32	4.98	0.00	ENSMUSG00000037171
Stk40	4	0.002	1.00	2.00	0.00	ENSMUSG00000042608
Emp1	6	0.002	1.85	3.60	0.00	ENSMUSG00000030208
Ndrg4	8	0.002	1.63	3.10	0.00	ENSMUSG00000036564
Trim12c	7	0.002	1.43	2.70	0.00	ENSMUSG00000057143
Mpp1	X	0.002	1.00	2.01	0.00	ENSMUSG00000031402

Ppl	16	0.002	2.41	5.30	0.00	ENSMUSG00000039457
Bcl2l1	2	0.002	1.10	2.15	0.00	ENSMUSG00000007659
Hist1h4n	13	0.002	2.17	4.49	0.00	ENSMUSG00000069305
Ifnar2	16	0.002	1.05	2.07	0.00	ENSMUSG00000022971
Des	1	0.002	2.20	4.60	0.00	ENSMUSG00000026208
Rab39b	X	0.002	2.83	7.13	0.00	ENSMUSG00000031202
Tex11	X	0.002	3.22	9.30	0.00	ENSMUSG00000009670
Zfp949	9	0.002	1.17	2.25	0.00	ENSMUSG00000032425
Tfap2c	2	0.003	2.51	5.68	0.00	ENSMUSG00000028640
Ulk1	5	0.003	1.09	2.13	0.00	ENSMUSG00000029512
Alas2	X	0.003	1.11	2.16	0.00	ENSMUSG00000025270
Phf23	11	0.003	1.19	2.29	0.00	ENSMUSG00000018572
Mbd6	10	0.003	1.09	2.13	0.00	ENSMUSG00000025409
Ranbp10	8	0.003	1.15	2.22	0.00	ENSMUSG00000037415
Ltbp3	19	0.003	2.66	6.31	0.00	ENSMUSG00000024940
Epha4	1	0.003	1.60	3.04	0.00	ENSMUSG00000026235
Ppp1r15a	7	0.003	1.24	2.36	0.00	ENSMUSG00000040435
Ephx4	5	0.003	3.88	14.72	0.00	ENSMUSG00000033805
Nefh	11	0.003	1.27	2.41	0.00	ENSMUSG00000020396
Hbb-bs	7	0.003	1.14	2.20	0.00	ENSMUSG00000052305
Cbx7	15	0.003	1.43	2.70	0.00	ENSMUSG00000053411
Tg	15	0.003	2.06	4.16	0.00	ENSMUSG00000053469
Adipor1	1	0.003	1.05	2.07	0.00	ENSMUSG00000026457
Ucp2	7	0.003	1.07	2.10	0.00	ENSMUSG00000033685
Igf2	7	0.003	2.16	4.46	0.00	ENSMUSG00000048583
Sema3a	5	0.003	3.07	8.38	0.00	ENSMUSG00000028883
Cdr2	7	0.003	1.10	2.14	0.00	ENSMUSG00000030878
Bmp4	14	0.003	2.74	6.66	0.00	ENSMUSG00000021835
Calcrl	2	0.003	1.24	2.36	0.00	ENSMUSG00000059588
Gja4	4	0.003	1.91	3.76	0.00	ENSMUSG00000050234
Sox8	17	0.003	3.60	12.11	0.00	ENSMUSG00000024176
Trib3	2	0.003	1.08	2.11	0.00	ENSMUSG00000032715
Cbfa2t3	8	0.003	1.15	2.22	0.00	ENSMUSG00000006362

Akap2	4	0.003	1.55	2.93	0.00	ENSMUSG00000038729
Peg3	7	0.003	1.23	2.35	0.00	ENSMUSG00000002265
Tmem28	X	0.003	3.59	12.07	0.00	ENSMUSG00000071719
Patl2	2	0.003	1.84	3.59	0.00	ENSMUSG00000027233
Klhl38	15	0.003	3.18	9.07	0.00	ENSMUSG00000022357
Grina	15	0.003	1.02	2.03	0.00	ENSMUSG00000022564
Perm1	4	0.004	1.98	3.94	0.00	ENSMUSG00000078486
Tnks1bp1	2	0.004	1.73	3.31	0.00	ENSMUSG00000033955
Eln	5	0.004	3.56	11.75	0.00	ENSMUSG00000029675
Ckap4	10	0.004	1.51	2.84	0.00	ENSMUSG00000046841
Hba-a2	11	0.004	1.15	2.21	0.00	ENSMUSG00000069917
Myof	19	0.004	1.90	3.73	0.00	ENSMUSG00000048612
Tax1bp1	6	0.004	1.05	2.07	0.00	ENSMUSG00000004535
Lama5	2	0.004	1.65	3.14	0.00	ENSMUSG00000015647
Nudt18	14	0.004	1.25	2.37	0.00	ENSMUSG00000045211
Plec	15	0.004	1.16	2.24	0.00	ENSMUSG00000022565
Micall1	15	0.004	1.17	2.25	0.00	ENSMUSG00000033039
Dcaf12	4	0.004	1.11	2.15	0.00	ENSMUSG00000028436
Scg2	1	0.004	2.14	4.39	0.00	ENSMUSG00000050711
Mapre3	5	0.004	1.91	3.75	0.00	ENSMUSG00000029166
Kctd11	11	0.004	1.77	3.42	0.00	ENSMUSG00000046731
Mettl7a2	15	0.004	2.47	5.53	0.00	ENSMUSG00000056487
Inka2	3	0.004	1.05	2.07	0.00	ENSMUSG00000048458
Vegfd	X	0.004	2.30	4.93	0.00	ENSMUSG00000031380
Lexm	4	0.004	2.10	4.29	0.00	ENSMUSG00000054362
Zfp365	10	0.004	2.55	5.84	0.00	ENSMUSG00000037855
Itgbl1	14	0.004	4.52	23.00	0.00	ENSMUSG00000032925
Olfml2b	1	0.004	2.75	6.74	0.00	ENSMUSG00000038463
Rnf227	11	0.004	2.54	5.81	0.00	ENSMUSG00000043419
Tinagl1	4	0.004	1.22	2.32	0.00	ENSMUSG00000028776
Cd68	11	0.004	1.44	2.71	0.00	ENSMUSG00000018774
Akr1b8	6	0.005	2.04	4.10	0.00	ENSMUSG00000029762
Ace2	X	0.005	2.80	6.99	0.00	ENSMUSG00000015405

Foxd1	13	0.005	3.83	14.19	0.00	ENSMUSG00000078302
Cacna2d3	14	0.005	1.75	3.36	0.00	ENSMUSG00000021991
Tgif1	17	0.005	1.33	2.51	0.00	ENSMUSG00000047407
Txnip	3	0.005	1.01	2.01	0.00	ENSMUSG00000038393
Tnc	4	0.005	2.31	4.95	0.01	ENSMUSG00000028364
Ubb	11	0.005	1.10	2.15	0.01	ENSMUSG00000019505
Gm9780	14	0.005	6.99	126.78	0.01	ENSMUSG00000094800
Tgm1	14	0.005	1.97	3.91	0.01	ENSMUSG00000022218
Smad1	8	0.005	1.58	3.00	0.01	ENSMUSG00000031681
Mlph	1	0.006	2.01	4.03	0.01	ENSMUSG00000026303
Fasl	1	0.006	1.89	3.72	0.01	ENSMUSG00000000817
Il6st	13	0.006	1.23	2.35	0.01	ENSMUSG00000021756
Sik1	17	0.006	1.21	2.31	0.01	ENSMUSG00000024042
Fbxl22	9	0.006	1.86	3.63	0.01	ENSMUSG00000050503
Ptms	6	0.006	1.18	2.26	0.01	ENSMUSG00000030122
Specc1	11	0.006	1.03	2.05	0.01	ENSMUSG00000042331
Spsb1	4	0.006	2.02	4.07	0.01	ENSMUSG00000039911
Npffr1	10	0.006	2.70	6.48	0.01	ENSMUSG00000020090
Nlgn2	11	0.006	1.29	2.44	0.01	ENSMUSG00000051790
Gm2115	7	0.006	3.63	12.36	0.01	ENSMUSG00000097789
Gm20547	17	0.006	7.42	171.18	0.01	ENSMUSG00000092511
Gprin2	14	0.006	2.37	5.18	0.01	ENSMUSG00000071531
Hist1h3b	13	0.006	2.26	4.79	0.01	ENSMUSG00000069267
Nlrc5	8	0.006	2.97	7.82	0.01	ENSMUSG00000074151
Cpeb2	5	0.007	1.05	2.07	0.01	ENSMUSG00000039782
Anxa2	9	0.007	1.30	2.46	0.01	ENSMUSG00000032231
Ly6k	15	0.007	3.44	10.87	0.01	ENSMUSG00000044678
Rhov	2	0.007	2.12	4.35	0.01	ENSMUSG00000034226
Zfp874b	13	0.007	1.14	2.21	0.01	ENSMUSG00000059839
Spag8	4	0.007	3.16	8.96	0.01	ENSMUSG00000066196
Sema6a	18	0.007	1.42	2.67	0.01	ENSMUSG00000019647
Mustn1	14	0.007	2.34	5.05	0.01	ENSMUSG00000042485
Tgfa	6	0.007	2.05	4.15	0.01	ENSMUSG00000029999

Cyp17a1	19	0.007	3.23	9.41	0.01	ENSMUSG00000003555
Tmem88	11	0.007	2.10	4.28	0.01	ENSMUSG00000045377
Igfn1	1	0.008	2.35	5.11	0.01	ENSMUSG00000051985
S100a6	3	0.008	1.69	3.22	0.01	ENSMUSG00000001025
Prelp	1	0.008	2.33	5.01	0.01	ENSMUSG00000041577
Atg16l2	7	0.008	1.74	3.34	0.01	ENSMUSG00000047767
Eepd1	9	0.008	1.74	3.34	0.01	ENSMUSG00000036611
Bmp8a	4	0.008	2.10	4.28	0.01	ENSMUSG00000032726
Hist2h2bb	3	0.008	2.32	5.01	0.01	ENSMUSG00000105827
Insl6	19	0.008	2.97	7.86	0.01	ENSMUSG00000050957
Anxa3	5	0.008	1.62	3.07	0.01	ENSMUSG00000029484
Ftl1-ps1	13	0.008	1.53	2.88	0.01	ENSMUSG00000062382
Col1a2	6	0.008	1.91	3.75	0.01	ENSMUSG00000029661
Psca	15	0.008	1.59	3.02	0.01	ENSMUSG00000022598
Hs3st3a1	11	0.008	2.23	4.68	0.01	ENSMUSG00000047759
Hist1h1e	13	0.008	1.76	3.40	0.01	ENSMUSG00000051627
Lypd5	7	0.008	3.52	11.45	0.01	ENSMUSG00000030484
Ugt1a8	1	0.008	6.78	110.21	0.01	ENSMUSG00000089675
Carmil1	13	0.008	1.26	2.40	0.01	ENSMUSG00000021338
Fbxo32	15	0.008	1.81	3.51	0.01	ENSMUSG00000022358
Letm2	8	0.009	1.20	2.29	0.01	ENSMUSG00000037363
Gm45208	X	0.009	3.11	8.62	0.01	ENSMUSG00000109493
Hist1h4m	13	0.009	2.31	4.97	0.01	ENSMUSG00000069306
Tmem88b	4	0.009	2.30	4.92	0.01	ENSMUSG00000073680
Lima1	15	0.009	1.86	3.64	0.01	ENSMUSG00000023022
Daam2	17	0.009	2.47	5.55	0.01	ENSMUSG00000040260
Crybg1	10	0.009	1.10	2.15	0.01	ENSMUSG00000019866
Eda2r	X	0.009	1.16	2.23	0.01	ENSMUSG00000034457
Myadm	7	0.009	1.60	3.04	0.01	ENSMUSG00000068566
Mturn	6	0.009	1.50	2.82	0.01	ENSMUSG00000038065
Apol10a	15	0.009	3.13	8.76	0.01	ENSMUSG00000050982
Mindy4	6	0.009	2.70	6.50	0.01	ENSMUSG00000038022
Adm	7	0.009	2.83	7.10	0.01	ENSMUSG00000030790

Gns	10	0.009	1.10	2.15	0.01	ENSMUSG00000034707
Eml1	12	0.010	2.24	4.74	0.01	ENSMUSG00000058070
Galnt15	14	0.010	4.09	17.05	0.01	ENSMUSG00000021903
Plekhm3	1	0.010	2.30	2.10	0.01	ENSMUSG00000051344
D630023F18Rik	1	0.010	2.50	4.80	0.01	ENSMUSG00000044816
Fam129a	1	0.010	2.33	2.54	0.01	ENSMUSG00000026483
Nos1ap	1	0.010	1.98	2.18	0.01	ENSMUSG00000038473
Fam129b	2	0.010	1.24	2.36	0.01	ENSMUSG00000026796
Pak6	2	0.010	1.27	2.23	0.01	ENSMUSG00000074923
Map1lc3a	2	0.010	1.66	2.00	0.01	ENSMUSG00000027602
Slc25a31	3	0.010	1.57	8.53	0.01	ENSMUSG00000069041
Fam160a1	3	0.010	1.88	2.25	0.01	ENSMUSG00000051000
Gm2163	4	0.010	1.11	5.13	0.01	ENSMUSG00000095779
Fsd1l	4	0.010	2.75	3.61	0.01	ENSMUSG00000054752
Tmem200b	4	0.010	2.72	6.87	0.01	ENSMUSG00000070720
Radil	5	0.010	1.69	3.93	0.01	ENSMUSG00000029576
Wnt2	6	0.010	2.29	3.15	0.01	ENSMUSG00000010797
Cd79a	7	0.010	1.96	5.78	0.01	ENSMUSG00000003379
Sptbn4	7	0.010	2.78	3.30	0.01	ENSMUSG00000011751
Zfp473	7	0.010	6.34	2.28	0.01	ENSMUSG00000048012
Gpr139	7	0.010	1.50	81.23	0.01	ENSMUSG00000066197
Fgfr1	8	0.010	3.30	2.16	0.01	ENSMUSG00000031565
Gdf1	8	0.010	1.13	17.25	0.01	ENSMUSG00000109523
Mmp12	9	0.010	1.42	6.21	0.01	ENSMUSG00000049723
Col5a3	9	0.010	1.18	5.67	0.01	ENSMUSG00000004098
Mfsd4b1	10	0.010	1.85	3.29	0.01	ENSMUSG00000038522
Nog	11	0.010	1.51	5.31	0.01	ENSMUSG00000048616
Cygb	11	0.010	2.43	6.17	0.01	ENSMUSG00000020810
Ltbp2	12	0.010	1.47	2.19	0.01	ENSMUSG00000002020
Hist1h2bg	13	0.010	2.08	3.49	0.01	ENSMUSG00000058385
Spata31d1a	13	0.010	3.09	6.01	0.01	ENSMUSG00000050876
1700014D04Rik	13	0.010	2.36	3.91	0.01	ENSMUSG00000051054
Ncf4	15	0.010	2.41	2.03	0.01	ENSMUSG00000071715

Rapgef3	15	0.010	1.51	4.91	0.01	ENSMUSG00000022469
Mettl7a3	15	0.010	4.15	5.41	0.01	ENSMUSG00000058057
Igfbp6	15	0.010	1.57	5.07	0.01	ENSMUSG00000023046
Adgrf5	17	0.010	1.49	4.04	0.01	ENSMUSG00000056492
A530064D06Rik	17	0.010	2.68	8.35	0.01	ENSMUSG00000043939
Dsc2	18	0.010	4.11	2.76	0.01	ENSMUSG00000024331
Arhgef37	18	0.010	2.34	2.94	0.01	ENSMUSG00000045094
Avpi1	19	0.010	2.75	2.35	0.01	ENSMUSG00000018821
Npl	1	0.020	1.78	2.56	0.02	ENSMUSG00000042684
Gm7694	1	0.020	2.13	2.06	0.02	ENSMUSG00000102752
Ephx1	1	0.020	1.59	3.01	0.02	ENSMUSG00000038776
Gm13889	2	0.020	2.81	3.05	0.02	ENSMUSG00000087006
Shld1	2	0.020	6.24	2.12	0.02	ENSMUSG00000044991
Sprr2k	3	0.020	1.93	72.15	0.02	ENSMUSG00000054215
Gstm1	3	0.020	1.87	2.68	0.02	ENSMUSG00000058135
Hook1	4	0.020	1.88	2.45	0.02	ENSMUSG00000028572
Plpp3	4	0.020	2.29	3.06	0.02	ENSMUSG00000028517
Oprd1	4	0.020	1.44	9.34	0.02	ENSMUSG00000050511
Grhl3	4	0.020	1.18	7.02	0.02	ENSMUSG00000037188
Rnf32	5	0.020	1.78	7.27	0.02	ENSMUSG00000029130
Zkscan14	5	0.020	1.11	2.04	0.02	ENSMUSG00000029627
Pdk4	6	0.020	1.75	3.43	0.02	ENSMUSG00000019577
Cacna2d4	6	0.020	2.14	3.63	0.02	ENSMUSG00000041460
Lrtm2	6	0.020	3.05	3.97	0.02	ENSMUSG00000055003
Bhlhe41	6	0.020	1.61	3.31	0.02	ENSMUSG00000030256
Klk6	7	0.020	2.23	8.18	0.02	ENSMUSG00000050063
Rras	7	0.020	1.95	2.36	0.02	ENSMUSG00000038387
Plekha4	7	0.020	2.36	2.78	0.02	ENSMUSG00000040428
Osgin1	8	0.020	1.30	3.43	0.02	ENSMUSG00000074063
Foxc2	8	0.020	2.51	75.83	0.02	ENSMUSG00000046714
Kank2	9	0.020	1.92	3.36	0.02	ENSMUSG00000032194
Nnmt	9	0.020	1.73	15.05	0.02	ENSMUSG00000032271
Myo5c	9	0.020	3.91	2.85	0.02	ENSMUSG00000033590

Spink8	9	0.020	2.85	7.21	0.02	ENSMUSG00000050074
Tmprss9	10	0.020	2.31	3.86	0.02	ENSMUSG00000059406
Gadd45b	10	0.020	2.03	2.47	0.02	ENSMUSG00000015312
Fam196b	11	0.020	2.46	13.52	0.02	ENSMUSG00000069911
Zfp354b	11	0.020	1.21	5.15	0.02	ENSMUSG00000020335
Itga3	11	0.020	1.80	3.22	0.02	ENSMUSG00000001507
Mycn	12	0.020	2.02	2.09	0.02	ENSMUSG00000037169
Hist1h2ae	13	0.020	1.47	3.60	0.02	ENSMUSG00000069272
Hist1h2bb	13	0.020	2.04	4.08	0.02	ENSMUSG00000075031
Spata31d1d	13	0.020	3.17	12.59	0.02	ENSMUSG00000043986
Cma1	14	0.020	1.08	2.28	0.02	ENSMUSG00000022225
Tnfrsf10b	14	0.020	2.91	2.19	0.02	ENSMUSG00000022074
Farp1	14	0.020	2.64	3.36	0.02	ENSMUSG00000025555
Kcnj4	15	0.020	3.55	10.14	0.02	ENSMUSG00000044216
Csrnp2	15	0.020	1.23	2.76	0.02	ENSMUSG00000044636
Tns2	15	0.020	2.34	3.15	0.02	ENSMUSG00000037003
Lrrc15	16	0.020	1.99	7.74	0.02	ENSMUSG00000052316
Olig2	16	0.020	1.76	62.18	0.02	ENSMUSG00000039830
Tnfrsf12a	17	0.020	1.01	2.96	0.02	ENSMUSG00000023905
Mocos	18	0.020	1.08	3.09	0.02	ENSMUSG00000039616
Pmaip1	18	0.020	3.80	2.41	0.02	ENSMUSG00000024521
Gstp2	19	0.020	2.30	3.54	0.02	ENSMUSG00000038155
Pyroxd2	19	0.020	1.30	4.42	0.02	ENSMUSG00000060224
Slc6a8	X	0.020	3.22	2.91	0.02	ENSMUSG00000019558
Gm4779	X	0.020	1.89	3.43	0.02	ENSMUSG00000045010
Prex2	1	0.030	1.22	6.23	0.03	ENSMUSG00000048960
Gpbar1	1	0.030	1.33	52.13	0.03	ENSMUSG00000064272
Prg4	1	0.030	2.76	46.08	0.03	ENSMUSG00000006014
Gpat2	2	0.030	1.75	4.19	0.03	ENSMUSG00000046338
Kcnb1	2	0.030	2.07	4.20	0.03	ENSMUSG00000050556
Wwtr1	3	0.030	1.94	2.59	0.03	ENSMUSG00000027803
Rhoc	3	0.030	1.85	2.57	0.03	ENSMUSG00000002233
Gm49337	4	0.030	2.18	9.89	0.03	ENSMUSG00000111410

Cfap69	5	0.030	2.30	6.58	0.03	ENSMUSG00000040473
Hspb1	5	0.030	1.91	2.78	0.03	ENSMUSG00000004951
C1ra	6	0.030	2.56	6.24	0.03	ENSMUSG00000055172
Pvr	7	0.030	3.55	3.09	0.03	ENSMUSG00000040511
Tecta	9	0.030	2.02	2.64	0.03	ENSMUSG00000037705
Gramd2	9	0.030	2.60	5.48	0.03	ENSMUSG00000074259
Trim43a	9	0.030	1.53	4.41	0.03	ENSMUSG00000090693
Tnfaip3	10	0.030	2.42	2.18	0.03	ENSMUSG00000019850
Cstb	10	0.030	3.32	2.02	0.03	ENSMUSG00000005054
Smim24	10	0.030	1.66	3.02	0.03	ENSMUSG00000078439
Gli1	10	0.030	1.54	5.41	0.03	ENSMUSG00000025407
Kcnj12	11	0.030	1.66	4.50	0.03	ENSMUSG00000042529
Pdk2	11	0.030	1.01	2.01	0.03	ENSMUSG00000038967
Cep112	11	0.030	2.20	5.41	0.03	ENSMUSG00000020728
Osr1	12	0.030	1.56	4.60	0.03	ENSMUSG00000048387
Nid1	13	0.030	1.64	2.97	0.03	ENSMUSG00000005397
Fermt2	14	0.030	2.39	2.93	0.03	ENSMUSG00000037712
Apol11a	15	0.030	1.59	4.00	0.03	ENSMUSG00000091650
Wbp2nl	15	0.030	2.11	5.87	0.03	ENSMUSG00000022455
Emp2	16	0.030	1.30	3.11	0.03	ENSMUSG00000022505
H2-T22	17	0.030	3.31	2.11	0.03	ENSMUSG00000056116
Peli3	19	0.030	1.10	3.52	0.03	ENSMUSG00000024901
Xlr4a	X	0.030	3.63	4.29	0.03	ENSMUSG00000079845
Slc19a3	1	0.040	3.32	39.60	0.04	ENSMUSG00000038496
Fam71a	1	0.040	2.65	6.69	0.04	ENSMUSG00000091017
Dnm1	2	0.040	1.64	3.30	0.04	ENSMUSG00000026825
Foxe1	4	0.040	1.50	9.97	0.04	ENSMUSG00000070990
Gpr157	4	0.040	1.31	2.79	0.04	ENSMUSG00000047875
Thegl	5	0.040	5.31	6.00	0.04	ENSMUSG00000029248
Afm	5	0.040	3.32	6.51	0.04	ENSMUSG00000029369
Gm43518	5	0.040	1.09	7.24	0.04	ENSMUSG00000105875
Gm10382	5	0.040	1.86	4.31	0.04	ENSMUSG00000072612
Tac1	6	0.040	2.14	43.13	0.04	ENSMUSG00000061762

Met	6	0.040	2.74	2.13	0.04	ENSMUSG00000009376
Apoe	7	0.040	5.47	2.52	0.04	ENSMUSG00000002985
Myod1	7	0.040	1.20	8.03	0.04	ENSMUSG00000009471
Tmc3	7	0.040	1.55	4.05	0.04	ENSMUSG00000038540
Usp17ld	7	0.040	2.02	43.56	0.04	ENSMUSG00000057321
Rassf10	7	0.040	3.55	4.65	0.04	ENSMUSG00000098132
Acsm1	7	0.040	1.14	11.69	0.04	ENSMUSG00000033533
Arhgef10	8	0.040	1.56	2.61	0.04	ENSMUSG00000071176
Mmp13	9	0.040	1.19	3.78	0.04	ENSMUSG00000050578
Rpp25	9	0.040	1.71	3.04	0.04	ENSMUSG00000062309
Lama4	10	0.040	1.52	3.68	0.04	ENSMUSG00000019846
Tmem106a	11	0.040	5.44	2.58	0.04	ENSMUSG00000034947
Hist1h2ab	13	0.040	1.37	3.68	0.04	ENSMUSG00000061615
Piwil2	14	0.040	1.62	39.73	0.04	ENSMUSG00000033644
Has2	15	0.040	3.18	3.67	0.04	ENSMUSG00000022367
Cd80	16	0.040	5.31	2.63	0.04	ENSMUSG00000075122
Rbm11	16	0.040	2.49	3.08	0.04	ENSMUSG00000032940
Sim2	16	0.040	1.46	39.73	0.04	ENSMUSG00000062713
Abhd3	18	0.040	1.35	5.14	0.04	ENSMUSG00000002475
Camk2a	18	0.040	2.36	2.87	0.04	ENSMUSG00000024617
Ms4a4d	19	0.040	1.70	46.93	0.04	ENSMUSG00000024678
Ubtd1	19	0.040	1.78	2.29	0.04	ENSMUSG00000025171
Col4a6	X	0.040	1.95	3.23	0.04	ENSMUSG00000031273
Il1f9	2	0.050	1.78	36.97	0.05	ENSMUSG00000044103
Uap1l1	2	0.050	2.36	2.05	0.05	ENSMUSG00000026956
Bcas1	2	0.050	5.19	38.88	0.05	ENSMUSG00000013523
Ttc22	4	0.050	2.15	5.80	0.05	ENSMUSG00000034919
Papolb	5	0.050	5.20	36.76	0.05	ENSMUSG00000074817
Abi3	11	0.050	1.42	2.03	0.05	ENSMUSG00000018381
Col14a1	15	0.050	1.02	6.12	0.05	ENSMUSG00000022371
Cryaa	17	0.050	3.18	4.34	0.05	ENSMUSG00000024041
Notch3	17	0.050	1.32	2.50	0.05	ENSMUSG00000038146
H2-Bl	17	0.050	2.48	5.59	0.05	ENSMUSG00000073406

Supplemental Table 9. Downregulated genes in *Dot1L-MM* ESRE cells in common with D*ot1L-KO* cells (n=198)

Name	Chromosome	Max group mean	Log_2 fold change	Fold change	P-value	ENSEMBL
Atp2a3	11	24.700	-1.85	-3.61	0.00	ENSMUSG00000020788
Itgb3	11	86.020	-2.33	-5.03	0.00	ENSMUSG00000020689
Alox12	11	16.560	-2.51	-5.68	0.00	ENSMUSG00000000320
Hmga1b	11	154.650	-2.06	-4.17	0.00	ENSMUSG00000078249
Mpl	4	9.910	-3.01	-8.08	0.00	ENSMUSG00000006389
Mpo	11	37.630	-2.60	-6.05	0.00	ENSMUSG00000009350
Gp5	16	22.080	-2.62	-6.15	0.00	ENSMUSG00000047953
Treml1	17	21.970	-2.89	-7.41	0.00	ENSMUSG00000023993
Gp1bb	16	21.440	-2.35	-5.09	0.00	ENSMUSG00000050761
Rasgrp2	19	5.500	-2.48	-5.56	0.00	ENSMUSG00000032946
Vwf	6	13.250	-1.66	-3.15	0.00	ENSMUSG00000001930
Slc35d3	10	5.700	-2.94	-7.66	0.00	ENSMUSG00000050473
F2rl2	13	7.810	-2.29	-4.90	0.00	ENSMUSG00000021675
Mmrn1	6	6.550	-2.57	-5.94	0.00	ENSMUSG00000054641
Gp1ba	11	14.750	-2.02	-4.06	0.00	ENSMUSG00000050675
Exoc3l2	7	1.210	-3.71	-13.06	0.00	ENSMUSG00000011263
Mrvi1	7	9.920	-1.66	-3.17	0.00	ENSMUSG00000005611
Ppbp	5	20.660	-2.27	-4.81	0.00	ENSMUSG00000029372
Anp32a	9	34.980	-1.23	-2.34	0.00	ENSMUSG00000032249
Clec1b	6	18.500	-2.31	-4.97	0.00	ENSMUSG00000030159
Gp9	6	37.980	-2.08	-4.24	0.00	ENSMUSG00000030054
Robo3	9	2.050	-3.11	-8.66	0.00	ENSMUSG00000032128
F5	1	3.720	-2.20	-4.60	0.00	ENSMUSG00000026579
Ppif	14	47.020	-1.26	-2.39	0.00	ENSMUSG00000021868
P2rx1	11	10.910	-2.25	-4.74	0.00	ENSMUSG00000020787
Prkca	11	4.140	-1.87	-3.66	0.00	ENSMUSG00000050965
Med12l	3	0.950	-2.52	-5.73	0.00	ENSMUSG00000056476
Rbpms2	9	10.140	-1.92	-3.78	0.00	ENSMUSG00000032387
Lrmp	6	28.090	-1.45	-2.73	0.00	ENSMUSG00000030263
Flt3	5	0.810	-3.62	-12.33	0.00	ENSMUSG00000042817

Serpinb2	1	5.940	-2.61	-6.12	0.00	ENSMUSG00000062345
Dnmt3b	2	3.730	-2.16	-4.46	0.00	ENSMUSG00000027478
Fli1	9	8.690	-1.96	-3.88	0.00	ENSMUSG00000016087
Slc38a1	15	12.310	-1.37	-2.58	0.00	ENSMUSG00000023169
Rasa3	8	14.330	-1.21	-2.32	0.00	ENSMUSG00000031453
Trpc6	9	0.630	-3.16	-8.95	0.00	ENSMUSG00000031997
Pdk1	2	24.630	-1.19	-2.27	0.00	ENSMUSG00000006494
Ripor2	13	1.260	-2.44	-5.45	0.00	ENSMUSG00000036006
Tnfsf14	17	4.510	-2.71	-6.55	0.00	ENSMUSG00000005824
Tpi1	6	212.280	-1.21	-2.31	0.00	ENSMUSG00000023456
Gucy1a1	3	1.920	-2.49	-5.62	0.00	ENSMUSG00000033910
Mylk	16	3.600	-1.79	-3.45	0.00	ENSMUSG00000022836
Bahcc1	11	2.230	-1.80	-3.48	0.00	ENSMUSG00000039741
Spns2	11	16.280	-1.61	-3.06	0.00	ENSMUSG00000040447
Slamf1	1	4.100	-2.39	-5.23	0.00	ENSMUSG00000015316
Ripor3	2	1.990	-2.52	-5.74	0.00	ENSMUSG00000074577
Lat	7	10.940	-2.11	-4.31	0.00	ENSMUSG00000030742
Bin2	15	37.920	-1.08	-2.11	0.00	ENSMUSG00000098112
Tnik	3	1.630	-1.74	-3.35	0.00	ENSMUSG00000027692
Psma8	18	0.990	-3.29	-9.76	0.00	ENSMUSG00000036743
Pde5a	3	3.340	-1.60	-3.02	0.00	ENSMUSG00000053965
Bzw2	12	29.370	-1.09	-2.13	0.00	ENSMUSG00000020547
Chd7	4	14.770	-1.04	-2.05	0.00	ENSMUSG00000041235
F2rl3	8	3.790	-2.42	-5.35	0.00	ENSMUSG00000050147
6-Sep	X	2.220	-1.81	-3.50	0.00	ENSMUSG00000050379
Cdk6	5	7.360	-1.10	-2.14	0.00	ENSMUSG00000040274
Zfp979	4	2.320	-2.59	-6.04	0.00	ENSMUSG00000066000
Cd226	18	1.290	-2.36	-5.14	0.00	ENSMUSG00000034028
Map2	1	0.340	-4.28	-19.45	0.00	ENSMUSG00000015222
Phgdh	3	75.230	-1.08	-2.11	0.00	ENSMUSG00000053398
Plcg2	8	9.960	-1.52	-2.86	0.00	ENSMUSG00000034330
Lin28b	10	13.290	-1.23	-2.35	0.00	ENSMUSG00000063804
Dcx	X	0.260	-5.34	-40.59	0.00	ENSMUSG00000031285

Sla	15	9.930	-1.60	-3.04	0.00	ENSMUSG00000022372
Ccdc85c	12	4.820	-1.58	-2.98	0.00	ENSMUSG00000084883
Dlg2	7	0.360	-2.63	-6.18	0.00	ENSMUSG00000052572
Ubash3a	17	1.710	-1.83	-3.55	0.00	ENSMUSG00000042345
Mef2c	13	2.360	-1.60	-3.03	0.00	ENSMUSG00000005583
Sms	X	11.510	-1.31	-2.49	0.00	ENSMUSG00000071708
Itgal	7	3.600	-1.65	-3.13	0.00	ENSMUSG00000030830
Capn3	2	0.720	-2.80	-6.98	0.00	ENSMUSG00000079110
Ptger3	3	13.960	-1.28	-2.44	0.00	ENSMUSG00000040016
Serpinb10	1	2.290	-2.73	-6.65	0.00	ENSMUSG00000092572
Psd4	2	3.580	-1.24	-2.37	0.00	ENSMUSG00000026979
Plppr3	10	1.030	-2.67	-6.36	0.00	ENSMUSG00000035835
Gucy1b1	3	3.300	-2.01	-4.03	0.00	ENSMUSG00000028005
Myct1	10	1.240	-2.49	-5.63	0.00	ENSMUSG00000046916
Sla2	2	3.110	-2.06	-4.17	0.00	ENSMUSG00000027636
Gp6	7	1.510	-2.07	-4.21	0.00	ENSMUSG00000078810
Igf2bp1	11	10.530	-1.13	-2.19	0.00	ENSMUSG00000013415
Slco4a1	2	4.060	-2.39	-5.24	0.00	ENSMUSG00000038963
Elf1	14	20.450	-1.04	-2.06	0.00	ENSMUSG00000036461
Rasal3	17	3.530	-1.75	-3.37	0.00	ENSMUSG00000052142
Mtss1l	8	3.500	-1.45	-2.73	0.00	ENSMUSG00000033763
Meis1	11	1.430	-2.01	-4.02	0.00	ENSMUSG00000020160
Tmprss7	16	0.360	-3.46	-11.02	0.00	ENSMUSG00000033177
Rab37	11	2.180	-2.03	-4.07	0.00	ENSMUSG00000020732
Dok2	14	11.250	-1.49	-2.80	0.00	ENSMUSG00000022102
Rps6ka6	X	5.290	-1.41	-2.66	0.00	ENSMUSG00000025665
Kcnk6	7	4.170	-1.45	-2.73	0.00	ENSMUSG00000046410
Bmi1	2	6.930	-1.35	-2.55	0.00	ENSMUSG00000026739
Cdca7	2	25.420	-1.00	-2.01	0.00	ENSMUSG00000055612
Slc22a3	17	1.070	-2.27	-4.84	0.00	ENSMUSG00000023828
Nfasc	1	0.080	-5.22	-37.32	0.00	ENSMUSG00000026442
Serpina3g	12	2.640	-2.13	-4.39	0.00	ENSMUSG00000041481
St8sia4	1	0.210	-2.23	-4.70	0.00	ENSMUSG00000040710

Unc119	11	18.560	-1.21	-2.31	0.00	ENSMUSG00000002058
Mfng	15	3.120	-1.90	-3.73	0.00	ENSMUSG00000018169
Gphn	12	7.320	-1.24	-2.36	0.00	ENSMUSG00000047454
Stmn2	3	0.800	-3.75	-13.43	0.00	ENSMUSG00000027500
Trmt6	2	14.050	-1.10	-2.15	0.00	ENSMUSG00000037376
Fxyd2	9	1.820	-3.27	-9.66	0.00	ENSMUSG00000059412
Prl2b1	13	0.620	-4.69	-25.88	0.00	ENSMUSG00000069258
Kcna3	3	1.930	-2.09	-4.26	0.00	ENSMUSG00000047959
Tmem163	1	0.710	-2.26	-4.80	0.00	ENSMUSG00000026347
3-Sep	15	0.340	-3.63	-12.35	0.00	ENSMUSG00000022456
BC035044	6	0.640	-2.94	-7.68	0.00	ENSMUSG00000090164
St8sia1	6	0.090	-3.28	-9.71	0.00	ENSMUSG00000030283
Apc2	10	0.310	-2.68	-6.41	0.00	ENSMUSG00000020135
Lrrc32	7	2.910	-1.47	-2.76	0.00	ENSMUSG00000090958
Suclg2	6	12.310	-1.02	-2.03	0.01	ENSMUSG00000061838
Smyd5	6	13.740	-1.12	-2.17	0.01	ENSMUSG00000033706
Chl1	6	0.070	-3.61	-12.20	0.01	ENSMUSG00000030077
Ncan	8	0.160	-7.49	-179.71	0.01	ENSMUSG00000002341
Agap2	10	1.990	-1.55	-2.92	0.01	ENSMUSG00000025422
Mug1	6	0.090	-4.38	-20.87	0.01	ENSMUSG00000059908
Dcc	18	0.110	-7.43	-173.01	0.01	ENSMUSG00000060534
Ankrd13b	11	4.780	-1.17	-2.25	0.01	ENSMUSG00000037907
Mrap	16	10.450	-1.59	-3.01	0.01	ENSMUSG00000039956
Myt1	2	0.120	-4.79	-27.64	0.01	ENSMUSG00000010505
Ccr9	9	0.090	-3.77	-13.65	0.01	ENSMUSG00000029530
Thbs1	2	154.090	-1.06	-2.08	0.01	ENSMUSG00000040152
Chrna4	2	0.180	-7.17	-143.94	0.01	ENSMUSG00000027577
Impa2	18	2.620	-1.37	-2.58	0.01	ENSMUSG00000024525
Ccdc116	16	2.420	-1.62	-3.08	0.01	ENSMUSG00000022768
Serpina3f	12	0.560	-2.56	-5.89	0.01	ENSMUSG00000066363
Crtam	9	0.290	-2.28	-4.86	0.01	ENSMUSG00000032021
mt-Nd3	MT	577.770	-1.04	-2.06	0.01	ENSMUSG00000064360
Rrp15	1	25.070	-1.07	-2.10	0.01	ENSMUSG00000001305

Prelid2	18	9.180	-1.68	-3.20	0.01	ENSMUSG00000056671
Prtn3	10	7.170	-1.82	-3.54	0.01	ENSMUSG00000057729
Eno1	4	178.420	-1.01	-2.01	0.01	ENSMUSG00000063524
Lhx1	11	0.160	-4.78	-27.38	0.01	ENSMUSG00000018698
Nlrp6	7	2.240	-1.51	-2.85	0.01	ENSMUSG00000038745
Arhgap15	2	0.840	-1.89	-3.70	0.01	ENSMUSG00000049744
Klhl6	16	7.440	-1.28	-2.43	0.01	ENSMUSG00000043008
Draxin	4	0.590	-1.98	-3.94	0.01	ENSMUSG00000029005
Bend3	10	4.300	-1.06	-2.09	0.01	ENSMUSG00000038214
Polr3g	13	6.840	-1.18	-2.27	0.01	ENSMUSG00000035834
Klk8	7	1.520	-2.18	-4.52	0.01	ENSMUSG00000064023
Slc25a13	6	6.950	-1.21	-2.32	0.01	ENSMUSG00000015112
Shmt1	11	8.160	-1.14	-2.20	0.01	ENSMUSG00000020534
Cacng4	11	0.260	-3.63	-12.37	0.01	ENSMUSG00000020723
Fastkd2	1	6.530	-1.08	-2.12	0.01	ENSMUSG00000025962
Scrt2	2	0.270	-3.60	-12.16	0.01	ENSMUSG00000060257
Stmn3	2	0.550	-6.63	-99.22	0.01	ENSMUSG00000027581
Il12a	3	0.360	-6.42	-85.49	0.01	ENSMUSG00000027776
Gria2	3	0.050	-3.74	-13.40	0.01	ENSMUSG00000033981
Celf3	3	0.280	-3.73	-13.25	0.01	ENSMUSG00000028137
Igfbpl1	4	0.230	-6.73	-105.93	0.01	ENSMUSG00000035551
Map4k1	7	5.730	-1.14	-2.20	0.01	ENSMUSG00000037337
Brsk2	7	0.110	-4.04	-16.48	0.01	ENSMUSG00000053046
Trim67	8	0.080	-6.76	-108.17	0.01	ENSMUSG00000036913
Chst11	10	6.610	-1.00	-2.01	0.01	ENSMUSG00000034612
Riox1	12	11.030	-1.05	-2.07	0.01	ENSMUSG00000046791
Cdca7l	12	6.430	-1.11	-2.16	0.01	ENSMUSG00000021175
Gm49391	13	5.780	-1.56	-2.94	0.01	ENSMUSG00000114432
Nefl	14	0.220	-3.35	-10.19	0.01	ENSMUSG00000022055
Ptprcap	19	4.410	-1.62	-3.08	0.01	ENSMUSG00000045826
Astn1	1	0.070	-3.92	-15.13	0.02	ENSMUSG00000026587
Slc24a5	2	3.850	-1.46	-2.74	0.02	ENSMUSG00000035183
Plcb1	2	0.200	-2.19	-4.55	0.02	ENSMUSG00000051177

Sprr1b	3	0.580	-6.26	-76.86	0.02	ENSMUSG00000048455
Cth	3	1.700	-1.77	-3.41	0.02	ENSMUSG00000028179
Zfp992	4	4.730	-1.15	-2.23	0.02	ENSMUSG00000070605
Cd69	6	0.720	-2.08	-4.22	0.02	ENSMUSG00000030156
Gmfg	7	5.900	-1.47	-2.78	0.02	ENSMUSG00000060791
Lrrc4b	7	0.240	-3.59	-12.08	0.02	ENSMUSG00000047085
Ptpn5	7	0.170	-6.60	-96.98	0.02	ENSMUSG00000030854
Adgrg3	8	3.100	-1.49	-2.80	0.02	ENSMUSG00000060470
Amd2	10	4.840	-1.25	-2.38	0.02	ENSMUSG00000063953
Atcay	10	0.130	-6.26	-76.86	0.02	ENSMUSG00000034958
Fmnl1	11	4.810	-1.11	-2.16	0.02	ENSMUSG00000055805
Trmt61a	12	8.790	-1.11	-2.16	0.02	ENSMUSG00000060950
Fst	13	1.130	-2.51	-5.69	0.02	ENSMUSG00000021765
Ydjc	16	4.730	-1.10	-2.14	0.02	ENSMUSG00000041774
Shd	17	0.180	-4.02	-16.23	0.02	ENSMUSG00000039154
Hhex	19	3.860	-1.37	-2.58	0.02	ENSMUSG00000024986
Traf3ip3	1	0.860	-1.60	-3.03	0.03	ENSMUSG00000037318
Nhlh2	3	0.070	-5.88	-58.97	0.03	ENSMUSG00000048540
Ptpn22	3	1.360	-1.87	-3.65	0.03	ENSMUSG00000027843
Cdh17	4	0.180	-2.19	-4.55	0.03	ENSMUSG00000028217
Zfp991	4	9.090	-1.10	-2.15	0.03	ENSMUSG00000067916
Kcnab2	4	4.560	-1.01	-2.01	0.03	ENSMUSG00000028931
Enoph1	5	10.040	-1.01	-2.02	0.03	ENSMUSG00000029326
Peg12	7	1.940	-1.55	-2.93	0.03	ENSMUSG00000070526
Apba2	7	0.110	-5.99	-63.44	0.03	ENSMUSG00000030519
Gm32687	10	0.140	-5.87	-58.51	0.03	ENSMUSG00000112640
Cmah	13	0.520	-1.43	-2.70	0.03	ENSMUSG00000016756
Nr2f1	13	0.130	-3.15	-8.90	0.03	ENSMUSG00000069171
Rab3c	13	0.050	-3.57	-11.86	0.03	ENSMUSG00000021700
Prodh	16	1.720	-1.40	-2.64	0.03	ENSMUSG00000003526
Dctd	8	4.890	-1.07	-2.11	0.04	ENSMUSG00000031562
Elavl3	9	0.220	-2.54	-5.80	0.04	ENSMUSG00000003410
B3gat1	9	0.070	-5.71	-52.26	0.04	ENSMUSG00000045994

Nsg2	11	0.250	-2.57	-5.94	0.04	ENSMUSG00000020297
Rpp40	13	1.480	-1.28	-2.43	0.04	ENSMUSG00000021418
Pax2	19	0.120	-3.09	-8.50	0.04	ENSMUSG00000004231

Supplemental Table 10. Upregulated genes in *Dot1L-MM* ESRE cells that are unique to these mutant cells (n=893)

Gene Name	Chromosome	Max group mean	Log_2 fold change	Fold change	P-value	ENSEMBL
Tspo2	17	0.000	2.88	7.38	0.00	ENSMUSG00000023995
Nacad	11	0.000	3.01	8.07	0.00	ENSMUSG00000041073
Inhba	13	0.000	3.80	13.91	0.00	ENSMUSG00000041324
Cdh2	18	0.000	3.35	10.22	0.00	ENSMUSG00000024304
Cald1	6	0.000	2.57	5.93	0.00	ENSMUSG00000029761
Zim1	7	0.000	3.18	9.04	0.00	ENSMUSG00000002266
Tgfb2	1	0.000	3.12	8.67	0.00	ENSMUSG00000039239
Adamts2	11	0.000	3.20	9.19	0.00	ENSMUSG00000036545
Actg2	6	0.000	3.75	13.48	0.00	ENSMUSG00000059430
Rgs5	1	0.000	4.03	16.39	0.00	ENSMUSG00000026678
Gas6	8	0.000	2.47	5.55	0.00	ENSMUSG00000031451
Vgll3	16	0.000	3.14	8.83	0.00	ENSMUSG00000091243
Cxcl12	6	0.000	3.20	9.18	0.00	ENSMUSG00000061353
Col4a5	X	0.000	2.89	7.44	0.00	ENSMUSG00000031274
Tpm2	4	0.000	3.08	8.46	0.00	ENSMUSG00000028464
Adam12	7	0.000	3.08	8.44	0.00	ENSMUSG00000054555
Gpnmb	6	0.000	2.12	4.34	0.00	ENSMUSG00000029816
Megf6	4	0.000	4.12	17.34	0.00	ENSMUSG00000057751
Adamts5	16	0.000	3.62	12.33	0.00	ENSMUSG00000022894
Zic3	X	0.000	4.68	25.64	0.00	ENSMUSG00000067860
Hand2	8	0.000	2.89	7.41	0.00	ENSMUSG00000038193
Ccn4	15	0.000	2.64	6.24	0.00	ENSMUSG00000005124
Lmod1	1	0.000	3.88	14.72	0.00	ENSMUSG00000048096
Adam19	11	0.000	2.74	6.69	0.00	ENSMUSG00000011256
Ifit1	19	0.000	2.14	4.39	0.00	ENSMUSG00000034459
Amotl1	9	0.000	2.55	5.84	0.00	ENSMUSG00000013076
Rmc1	18	0.000	1.34	2.52	0.00	ENSMUSG00000024410
Adam8	7	0.000	2.28	4.85	0.00	ENSMUSG00000025473
Erv3	2	0.000	4.30	19.65	0.00	ENSMUSG00000037482
C1qc	4	0.000	2.18	4.52	0.00	ENSMUSG00000036896

Lrrc75b	10	0.000	3.91	15.05	0.00	ENSMUSG00000046807
Hs6st2	X	0.000	2.40	5.29	0.00	ENSMUSG00000062184
Loxl4	19	0.000	3.73	13.30	0.00	ENSMUSG00000025185
Grem2	1	0.000	3.25	9.51	0.00	ENSMUSG00000050069
Cyp1b1	17	0.000	3.08	8.46	0.00	ENSMUSG00000024087
Lrp1	10	0.000	2.18	4.55	0.00	ENSMUSG00000040249
Csf1	3	0.000	2.68	6.40	0.00	ENSMUSG00000014599
Hoxa11	6	0.000	3.93	15.29	0.00	ENSMUSG00000038210
Pdlim3	8	0.000	4.48	22.38	0.00	ENSMUSG00000031636
Igfbp7	5	0.000	3.10	8.55	0.00	ENSMUSG00000036256
Crispld2	8	0.000	2.44	5.42	0.00	ENSMUSG00000031825
Lpl	8	0.000	1.41	2.66	0.00	ENSMUSG00000015568
Pcolce	5	0.000	2.82	7.07	0.00	ENSMUSG00000029718
Adgre1	17	0.000	2.03	4.10	0.00	ENSMUSG00000004730
Ddr2	1	0.000	3.13	8.77	0.00	ENSMUSG00000026674
Tbx20	9	0.000	3.16	8.92	0.00	ENSMUSG00000031965
C1qa	4	0.000	2.36	5.13	0.00	ENSMUSG00000036887
Serpine2	1	0.000	2.11	4.32	0.00	ENSMUSG00000026249
Zdbf2	1	0.000	2.54	5.82	0.00	ENSMUSG00000027520
Ece1	4	0.000	2.20	4.58	0.00	ENSMUSG00000057530
Ncam1	9	0.000	2.36	5.15	0.00	ENSMUSG00000039542
Myocd	11	0.000	3.83	14.24	0.00	ENSMUSG00000020542
Nipal4	11	0.000	4.31	19.86	0.00	ENSMUSG00000020411
Ptn	6	0.000	3.03	8.15	0.00	ENSMUSG00000029838
Fbln2	6	0.000	2.26	4.78	0.00	ENSMUSG00000064080
Fndc1	17	0.000	3.96	15.59	0.00	ENSMUSG00000071984
Mrc2	11	0.000	2.62	6.17	0.00	ENSMUSG00000020695
C3ar1	6	0.000	1.84	3.58	0.00	ENSMUSG00000040552
Itgb5	16	0.000	2.33	5.02	0.00	ENSMUSG00000022817
Vcam1	3	0.000	3.68	12.85	0.00	ENSMUSG00000027962
Cmklr1	5	0.000	2.87	7.32	0.00	ENSMUSG00000042190
Kif26b	1	0.000	3.11	8.62	0.00	ENSMUSG00000026494
Mamdc2	19	0.000	2.93	7.63	0.00	ENSMUSG00000033207

Lynx1	15	0.000	4.50	22.56	0.00	ENSMUSG00000022594
Adamtsl3	7	0.000	5.35	40.69	0.00	ENSMUSG00000070469
Nuak1	10	0.000	2.62	6.15	0.00	ENSMUSG00000020032
Ccn5	2	0.000	4.08	16.88	0.00	ENSMUSG00000027656
Psap	10	0.000	1.16	2.24	0.00	ENSMUSG00000004207
Enho	4	0.000	3.51	11.42	0.00	ENSMUSG00000028445
Mical2	7	0.000	2.50	5.66	0.00	ENSMUSG00000038244
Gprc5b	7	0.000	2.08	4.23	0.00	ENSMUSG00000008734
Clca3a1	3	0.000	2.57	5.95	0.00	ENSMUSG00000056025
Wnt5a	14	0.000	3.08	8.44	0.00	ENSMUSG00000021994
Dtna	18	0.000	3.74	13.35	0.00	ENSMUSG00000024302
Myl9	2	0.000	2.28	4.85	0.00	ENSMUSG00000067818
Mafb	2	0.000	2.51	5.70	0.00	ENSMUSG00000074622
Ccn3	15	0.000	4.62	24.55	0.00	ENSMUSG00000037362
C1qb	4	0.000	1.97	3.90	0.00	ENSMUSG00000036905
Siglecg	7	0.000	3.33	10.06	0.00	ENSMUSG00000030468
Nrp1	8	0.000	2.45	5.45	0.00	ENSMUSG00000025810
Thsd7a	6	0.000	2.88	7.37	0.00	ENSMUSG00000032625
Zcchc24	14	0.000	2.25	4.74	0.00	ENSMUSG00000055538
Adamts12	15	0.000	2.91	7.52	0.00	ENSMUSG00000047497
Wscd2	5	0.000	3.88	14.74	0.00	ENSMUSG00000063430
Vcan	13	0.000	2.62	6.15	0.00	ENSMUSG00000021614
Rbms3	9	0.000	2.82	7.07	0.00	ENSMUSG00000039607
Tnnt2	1	0.000	2.63	6.20	0.00	ENSMUSG00000026414
Itga11	9	0.000	4.82	28.24	0.00	ENSMUSG00000032243
Tcfl5	2	0.000	3.74	13.34	0.00	ENSMUSG00000038932
Ripply1	X	0.000	3.74	13.34	0.00	ENSMUSG00000072945
Parva	7	0.000	2.30	4.94	0.00	ENSMUSG00000030770
Prrx1	1	0.000	3.33	10.06	0.00	ENSMUSG00000026586
Adcy3	12	0.000	2.40	5.29	0.00	ENSMUSG00000020654
Pitx2	3	0.000	2.68	6.41	0.00	ENSMUSG00000028023
Tnnt3	7	0.000	3.69	12.86	0.00	ENSMUSG00000061723
Palld	8	0.001	2.09	4.26	0.00	ENSMUSG00000058056

Nptx2	5	0.001	3.75	13.48	0.00	ENSMUSG00000059991
Fscn1	5	0.001	2.19	4.55	0.00	ENSMUSG00000029581
Cavin3	7	0.001	2.59	6.04	0.00	ENSMUSG00000037060
Spon1	7	0.001	2.76	6.79	0.00	ENSMUSG00000038156
Nfib	4	0.001	2.63	6.19	0.00	ENSMUSG00000008575
Fndc5	4	0.001	2.67	6.35	0.00	ENSMUSG00000001334
Marveld1	19	0.001	2.13	4.37	0.00	ENSMUSG00000044345
Fam20a	11	0.001	3.73	13.31	0.00	ENSMUSG00000020614
Tbx4	11	0.001	3.13	8.78	0.00	ENSMUSG00000000094
Olfr78	7	0.001	4.53	23.10	0.00	ENSMUSG00000043366
Slc37a2	9	0.001	1.95	3.87	0.00	ENSMUSG00000032122
Loxl1	9	0.001	2.41	5.30	0.00	ENSMUSG00000032334
Marcks	10	0.001	1.80	3.48	0.00	ENSMUSG00000069662
Adamts15	9	0.001	2.72	6.57	0.00	ENSMUSG00000033453
Aff2	X	0.001	2.66	6.34	0.00	ENSMUSG00000031189
Tmem119	5	0.001	2.85	7.23	0.00	ENSMUSG00000054675
Trabd2b	4	0.001	2.79	6.90	0.00	ENSMUSG00000070867
Mmp14	14	0.001	2.18	4.54	0.00	ENSMUSG00000000957
Itga5	15	0.001	1.70	3.24	0.00	ENSMUSG00000000555
Kcnv2	19	0.001	4.49	22.45	0.00	ENSMUSG00000047298
Ust	10	0.001	2.71	6.54	0.00	ENSMUSG00000047712
Hoxa13	6	0.001	3.75	13.44	0.00	ENSMUSG00000038203
Dkk3	7	0.001	3.19	9.12	0.00	ENSMUSG00000030772
Ctss	3	0.001	1.87	3.65	0.00	ENSMUSG00000038642
Padi3	4	0.001	2.59	6.03	0.00	ENSMUSG00000025328
Mill2	7	0.001	3.44	10.88	0.00	ENSMUSG00000040987
Anxa5	3	0.001	1.43	2.70	0.00	ENSMUSG00000027712
Ankrd1	19	0.001	2.58	5.99	0.00	ENSMUSG00000024803
Ccn2	10	0.001	2.02	4.06	0.00	ENSMUSG00000019997
Shisa4	1	0.001	2.75	6.74	0.00	ENSMUSG00000041889
Ly6a	15	0.001	2.37	5.16	0.00	ENSMUSG00000075602
Sulf2	2	0.001	2.28	4.86	0.00	ENSMUSG00000006800
Apol9a	15	0.001	5.56	47.07	0.00	ENSMUSG00000057346

Grn	11	0.001	1.35	2.56	0.00	ENSMUSG00000034708
Dkk2	3	0.001	3.41	10.63	0.00	ENSMUSG00000028031
Cpne2	8	0.001	2.57	5.92	0.00	ENSMUSG00000034361
Msrb3	10	0.001	2.26	4.78	0.00	ENSMUSG00000051236
Cd248	19	0.001	1.70	3.25	0.00	ENSMUSG00000056481
Tubb6	18	0.001	1.83	3.57	0.00	ENSMUSG00000001473
Syde1	10	0.001	2.58	5.99	0.00	ENSMUSG00000032714
Fstl3	10	0.001	2.49	5.61	0.00	ENSMUSG00000020325
Hmgcll1	9	0.001	2.87	7.32	0.00	ENSMUSG00000007908
Phactr2	10	0.001	1.62	3.08	0.00	ENSMUSG00000062866
Scrn1	6	0.001	2.28	4.87	0.00	ENSMUSG00000019124
Plxna1	6	0.001	1.08	2.12	0.00	ENSMUSG00000030084
Nav2	7	0.001	1.42	2.67	0.00	ENSMUSG00000052512
Sdc2	15	0.001	2.84	7.15	0.00	ENSMUSG00000022261
Pdgfc	3	0.001	2.69	6.45	0.00	ENSMUSG00000028019
Col16a1	4	0.001	2.39	5.24	0.00	ENSMUSG00000040690
Ms4a6d	19	0.001	2.31	4.98	0.00	ENSMUSG00000024679
Il6ra	3	0.001	1.43	2.69	0.00	ENSMUSG00000027947
Ahi1	10	0.001	1.21	2.31	0.00	ENSMUSG00000019986
Pitx1	13	0.001	2.85	7.21	0.00	ENSMUSG00000021506
Bdnf	2	0.001	3.13	8.74	0.00	ENSMUSG00000048482
Slc1a6	10	0.001	4.66	25.29	0.00	ENSMUSG00000005357
Gbgt1	2	0.001	4.31	19.90	0.00	ENSMUSG00000026829
Cnn1	9	0.001	3.09	8.51	0.00	ENSMUSG00000001349
Pls3	X	0.001	1.61	3.04	0.00	ENSMUSG00000016382
Adam23	1	0.001	2.78	6.87	0.00	ENSMUSG00000025964
Mrc1	2	0.001	1.80	3.49	0.00	ENSMUSG00000026712
Nbl1	4	0.001	2.42	5.36	0.00	ENSMUSG00000041120
Adam22	5	0.001	2.48	5.58	0.00	ENSMUSG00000040537
Ngp	9	0.001	2.30	4.94	0.00	ENSMUSG00000032484
C1qtnf2	11	0.001	3.57	11.87	0.00	ENSMUSG00000046491
Tcp11	17	0.001	3.72	13.21	0.00	ENSMUSG00000062859
Ngf	3	0.001	3.75	13.48	0.00	ENSMUSG00000027859

Numbl	7	0.001	1.69	3.23	0.00	ENSMUSG00000063160
Trem2	17	0.001	2.23	4.68	0.00	ENSMUSG00000023992
Ldb3	14	0.001	3.53	11.54	0.00	ENSMUSG00000021798
Mxra8	4	0.001	2.26	4.80	0.00	ENSMUSG00000029070
Mgst1	6	0.001	2.65	6.27	0.00	ENSMUSG00000008540
Apol6	15	0.001	4.91	30.12	0.00	ENSMUSG00000033576
Klf14	6	0.001	3.51	11.43	0.00	ENSMUSG00000073209
2310022B05Rik	8	0.001	1.01	2.02	0.00	ENSMUSG00000031983
Actn1	12	0.001	1.53	2.88	0.00	ENSMUSG00000015143
Cdk18	1	0.001	2.20	4.59	0.00	ENSMUSG00000026437
Cdh13	8	0.002	3.05	8.26	0.00	ENSMUSG00000031841
Aox1	1	0.002	3.46	11.00	0.00	ENSMUSG00000063558
C1qtnf1	11	0.002	2.73	6.64	0.00	ENSMUSG00000017446
Tex15	8	0.002	2.32	4.99	0.00	ENSMUSG00000009628
Xdh	17	0.002	2.42	5.36	0.00	ENSMUSG00000024066
Rgs4	1	0.002	3.26	9.60	0.00	ENSMUSG00000038530
Hspb8	5	0.002	1.48	2.78	0.00	ENSMUSG00000041548
Kcnmb1	11	0.002	3.00	7.99	0.00	ENSMUSG00000020155
Fzd1	5	0.002	2.84	7.18	0.00	ENSMUSG00000044674
Vasn	16	0.002	2.27	4.82	0.00	ENSMUSG00000039646
Tppp3	8	0.002	2.05	4.14	0.00	ENSMUSG00000014846
Tsku	7	0.002	2.41	5.30	0.00	ENSMUSG00000049580
Pea15a	1	0.002	1.72	3.30	0.00	ENSMUSG00000013698
Ifi204	1	0.002	2.46	5.49	0.00	ENSMUSG00000073489
Plekhg4	8	0.002	3.36	10.30	0.00	ENSMUSG00000014782
Ly6c1	15	0.002	3.63	12.37	0.00	ENSMUSG00000079018
Pappa	4	0.002	2.63	6.18	0.00	ENSMUSG00000028370
Eya4	10	0.002	2.99	7.92	0.00	ENSMUSG00000010461
Dclk2	3	0.002	1.34	2.54	0.00	ENSMUSG00000028078
Arhgap31	16	0.002	1.58	2.99	0.00	ENSMUSG00000022799
Cpa6	1	0.002	4.22	18.58	0.00	ENSMUSG00000042501
Cpe	8	0.002	1.94	3.84	0.00	ENSMUSG00000037852
Evx1	6	0.002	5.18	36.37	0.00	ENSMUSG00000005503

Nectin1	9	0.002	1.92	3.79	0.00	ENSMUSG00000032012
Selenon	4	0.002	1.56	2.94	0.00	ENSMUSG00000050989
Msr1	8	0.002	2.40	5.29	0.00	ENSMUSG00000025044
Prag1	8	0.002	2.23	4.71	0.00	ENSMUSG00000050271
Npc1	18	0.002	1.15	2.22	0.00	ENSMUSG00000024413
Gpr50	X	0.002	3.75	13.47	0.00	ENSMUSG00000056380
Dusp27	1	0.002	3.30	9.85	0.00	ENSMUSG00000026564
Serpinb9b	13	0.002	2.13	4.37	0.00	ENSMUSG00000021403
Lum	10	0.002	2.63	6.21	0.00	ENSMUSG00000036446
Fzd2	11	0.002	2.42	5.36	0.00	ENSMUSG00000050288
Amotl2	9	0.002	1.76	3.38	0.00	ENSMUSG00000032531
Il17rc	6	0.002	2.82	7.05	0.00	ENSMUSG00000030281
Piezo2	18	0.002	3.05	8.29	0.00	ENSMUSG00000041482
Rspo3	10	0.002	3.41	10.64	0.00	ENSMUSG00000019880
Coro6	11	0.002	3.53	11.52	0.00	ENSMUSG00000020836
Fam149a	8	0.002	2.65	6.29	0.00	ENSMUSG00000070044
Plin4	17	0.002	7.72	210.57	0.00	ENSMUSG00000002831
Gas1	13	0.002	2.38	5.20	0.00	ENSMUSG00000052957
Gpx7	4	0.002	2.87	7.31	0.00	ENSMUSG00000028597
Snai2	16	0.002	2.90	7.46	0.00	ENSMUSG00000022676
Fkbp10	11	0.002	2.13	4.37	0.00	ENSMUSG00000001555
Tgfbi	13	0.002	2.35	5.10	0.00	ENSMUSG00000035493
Tro	X	0.002	2.40	5.29	0.00	ENSMUSG00000025272
Tead1	7	0.003	2.03	4.10	0.00	ENSMUSG00000055320
Mocs1	17	0.003	1.09	2.14	0.00	ENSMUSG00000064120
Ssc5d	7	0.003	2.50	5.64	0.00	ENSMUSG00000035279
Igf2r	17	0.003	1.19	2.29	0.00	ENSMUSG00000023830
Asb12	X	0.003	3.36	10.26	0.00	ENSMUSG00000031204
Tmem184a	5	0.003	1.21	2.32	0.00	ENSMUSG00000036687
Tpbgl	7	0.003	2.26	4.80	0.00	ENSMUSG00000096606
Rab34	11	0.003	2.22	4.65	0.00	ENSMUSG00000002059
Irs1	1	0.003	2.49	5.60	0.00	ENSMUSG00000055980
Siglec1	2	0.003	2.17	4.51	0.00	ENSMUSG00000027322

Ak5	3	0.003	2.98	7.89	0.00	ENSMUSG00000039058
C5ar1	7	0.003	2.08	4.21	0.00	ENSMUSG00000049130
Suox	10	0.003	1.63	3.10	0.00	ENSMUSG00000049858
Ccn1	3	0.003	1.92	3.78	0.00	ENSMUSG00000028195
Nrep	18	0.003	2.54	5.81	0.00	ENSMUSG00000042834
Pcdh18	3	0.003	2.79	6.91	0.00	ENSMUSG00000037892
Ghrhr	6	0.003	3.95	15.44	0.00	ENSMUSG00000004654
Efemp2	19	0.003	2.16	4.47	0.00	ENSMUSG00000024909
Efr3b	12	0.003	2.36	5.14	0.00	ENSMUSG00000020658
Hk3	13	0.003	1.91	3.75	0.00	ENSMUSG00000025877
Sdk1	5	0.003	2.52	5.72	0.00	ENSMUSG00000039683
Mest	6	0.003	1.55	2.92	0.00	ENSMUSG00000051855
Nox4	7	0.003	3.23	9.41	0.00	ENSMUSG00000030562
Spire2	8	0.003	1.97	3.93	0.00	ENSMUSG00000010154
Heg1	16	0.003	1.26	2.40	0.00	ENSMUSG00000075254
Crlf1	8	0.003	2.05	4.14	0.00	ENSMUSG00000007888
Ms4a14	19	0.003	3.15	8.89	0.00	ENSMUSG00000099398
Mxra7	11	0.003	2.33	5.01	0.00	ENSMUSG00000020814
Anpep	7	0.003	1.21	2.31	0.00	ENSMUSG00000039062
Sh3tc2	18	0.003	1.04	2.06	0.00	ENSMUSG00000045629
Pianp	6	0.003	2.99	7.93	0.00	ENSMUSG00000030329
Tnfrsf1b	4	0.004	1.47	2.77	0.00	ENSMUSG00000028599
Ndn	7	0.004	2.10	4.29	0.00	ENSMUSG00000033585
Hrasls	16	0.004	4.15	17.79	0.00	ENSMUSG00000022525
Slc2a4	11	0.004	1.23	2.35	0.00	ENSMUSG00000018566
Irgm1	11	0.004	1.25	2.38	0.00	ENSMUSG00000046879
Lipa	19	0.004	1.08	2.11	0.00	ENSMUSG00000024781
Htra3	5	0.004	2.67	6.36	0.00	ENSMUSG00000029096
Cbr3	16	0.004	2.98	7.90	0.00	ENSMUSG00000022947
Gpr176	2	0.004	2.72	6.59	0.00	ENSMUSG00000040133
Oas2	5	0.004	2.56	5.90	0.00	ENSMUSG00000032690
Chst2	9	0.004	2.58	5.98	0.00	ENSMUSG00000033350
Apol9b	15	0.004	3.13	8.75	0.00	ENSMUSG00000068246

Sorbs3	14	0.004	2.10	4.30	0.00	ENSMUSG00000022091
Fgf15	7	0.004	2.89	7.43	0.00	ENSMUSG00000031073
Tead2	7	0.004	2.26	4.79	0.00	ENSMUSG00000030796
Lipo3	19	0.004	2.27	4.84	0.00	ENSMUSG00000024766
C1qtnf6	15	0.004	2.33	5.04	0.00	ENSMUSG00000022440
Klhl30	1	0.004	2.69	6.44	0.00	ENSMUSG00000026308
Kcnab1	3	0.004	2.82	7.07	0.00	ENSMUSG00000027827
Ugt1a6a	1	0.004	3.10	8.60	0.00	ENSMUSG00000054545
Klf4	4	0.004	1.74	3.34	0.00	ENSMUSG00000003032
Filip1l	16	0.004	2.05	4.13	0.00	ENSMUSG00000043336
Adamts9	6	0.004	1.90	3.73	0.00	ENSMUSG00000030022
Cpeb1	7	0.004	2.47	5.54	0.00	ENSMUSG00000025586
Cacna2d1	5	0.004	2.41	5.30	0.00	ENSMUSG00000040118
Gpr1	1	0.004	3.19	9.11	0.00	ENSMUSG00000046856
Cpne5	17	0.004	1.49	2.81	0.00	ENSMUSG00000024008
Hnmt	2	0.004	3.44	10.84	0.00	ENSMUSG00000026986
Chil3	3	0.005	2.89	7.43	0.00	ENSMUSG00000040809
Metrnl	11	0.005	2.27	4.82	0.00	ENSMUSG00000039208
Zfp287	11	0.005	2.96	7.77	0.00	ENSMUSG00000005267
Lipe	7	0.005	1.19	2.28	0.00	ENSMUSG00000003123
Igf1	10	0.005	1.76	3.39	0.00	ENSMUSG00000020053
Fmn1	2	0.005	1.94	3.83	0.00	ENSMUSG00000044042
Epb41l3	17	0.005	2.26	4.80	0.00	ENSMUSG00000024044
Synpo2	3	0.005	2.00	4.01	0.00	ENSMUSG00000050315
Fbln5	12	0.005	2.57	5.92	0.00	ENSMUSG00000021186
Sema3c	5	0.005	2.44	5.42	0.00	ENSMUSG00000028780
P2ry6	7	0.005	1.94	3.85	0.00	ENSMUSG00000048779
Rab7b	1	0.005	1.87	3.66	0.00	ENSMUSG00000052688
Arc	15	0.005	2.62	6.15	0.01	ENSMUSG00000022602
Ifi207	1	0.005	1.83	3.55	0.01	ENSMUSG00000073490
Gja5	3	0.005	3.24	9.48	0.01	ENSMUSG00000057123
Sdc3	4	0.005	1.84	3.58	0.01	ENSMUSG00000025743
Nes	3	0.005	2.23	4.69	0.01	ENSMUSG00000004891

Akap12	10	0.005	1.78	3.43	0.01	ENSMUSG00000038587
Phldb1	9	0.005	1.19	2.28	0.01	ENSMUSG00000048537
Flrt2	12	0.005	2.11	4.33	0.01	ENSMUSG00000047414
Slc7a10	7	0.005	4.41	21.23	0.01	ENSMUSG00000030495
Mid2	X	0.005	2.53	5.78	0.01	ENSMUSG00000000266
Mfap5	6	0.005	4.67	25.53	0.01	ENSMUSG00000030116
Tlr8	X	0.005	2.79	6.90	0.01	ENSMUSG00000040522
Lpar4	X	0.006	3.05	8.28	0.01	ENSMUSG00000049929
Bend6	1	0.006	3.85	14.45	0.01	ENSMUSG00000042182
Gxylt2	6	0.006	3.11	8.64	0.01	ENSMUSG00000030074
Gli3	13	0.006	2.32	4.98	0.01	ENSMUSG00000021318
Parp14	16	0.006	1.36	2.57	0.01	ENSMUSG00000034422
Efs	14	0.006	2.71	6.55	0.01	ENSMUSG00000022203
Plxdc1	11	0.006	2.41	5.32	0.01	ENSMUSG00000017417
Ms4a4a	19	0.006	2.18	4.52	0.01	ENSMUSG00000101389
Azin2	4	0.006	2.31	4.96	0.01	ENSMUSG00000028789
Pde4dip	3	0.006	1.25	2.38	0.01	ENSMUSG00000038170
Dlc1	8	0.006	1.61	3.04	0.01	ENSMUSG00000031523
Ifit3b	19	0.006	2.80	6.95	0.01	ENSMUSG00000062488
Ppp1r14a	7	0.006	2.99	7.92	0.01	ENSMUSG00000037166
Speg	1	0.006	2.02	4.05	0.01	ENSMUSG00000026207
Larp6	9	0.006	4.44	21.74	0.01	ENSMUSG00000034839
Cmtm3	8	0.006	1.65	3.14	0.01	ENSMUSG00000031875
Art3	5	0.006	2.48	5.58	0.01	ENSMUSG00000034842
Gbp2	3	0.006	2.42	5.35	0.01	ENSMUSG00000028270
3830417A13Rik	X	0.006	3.07	8.40	0.01	ENSMUSG00000031179
Antxr2	5	0.006	1.75	3.36	0.01	ENSMUSG00000029338
Serpinh1	7	0.007	1.80	3.47	0.01	ENSMUSG00000070436
Steap2	5	0.007	2.26	4.80	0.01	ENSMUSG00000015653
Col8a2	4	0.007	2.53	5.79	0.01	ENSMUSG00000056174
Lats2	14	0.007	1.20	2.30	0.01	ENSMUSG00000021959
Lrrc55	2	0.007	3.32	9.99	0.01	ENSMUSG00000075224
Lmcd1	6	0.007	2.35	5.10	0.01	ENSMUSG00000057604

Plcd1	9	0.007	2.16	4.46	0.01	ENSMUSG00000010660
Pmepa1	2	0.007	2.08	4.22	0.01	ENSMUSG00000038400
P4ha3	7	0.007	2.09	4.26	0.01	ENSMUSG00000051048
Cd300ld5	11	0.007	2.98	7.91	0.01	ENSMUSG00000089722
Pdzrn3	6	0.007	2.17	4.51	0.01	ENSMUSG00000035357
Igdcc4	9	0.007	2.34	5.06	0.01	ENSMUSG00000032816
Padi1	4	0.007	2.90	7.48	0.01	ENSMUSG00000025329
Map6	7	0.007	2.26	4.80	0.01	ENSMUSG00000055407
Mchr1	15	0.007	3.13	8.77	0.01	ENSMUSG00000050164
Pparg	6	0.007	1.96	3.89	0.01	ENSMUSG00000000440
Naalad2	9	0.007	3.32	9.98	0.01	ENSMUSG00000043943
Sgsm1	5	0.007	2.43	5.39	0.01	ENSMUSG00000042216
Gpr153	4	0.007	2.40	5.27	0.01	ENSMUSG00000042804
Tmem26	10	0.008	3.45	10.92	0.01	ENSMUSG00000060044
Gbp6	5	0.008	3.06	8.37	0.01	ENSMUSG00000104713
Samd4	14	0.008	1.83	3.56	0.01	ENSMUSG00000021838
Mcc	18	0.008	2.22	4.66	0.01	ENSMUSG00000071856
Parm1	5	0.008	1.62	3.06	0.01	ENSMUSG00000034981
Hspb2	9	0.008	3.15	8.89	0.01	ENSMUSG00000038086
Ccl2	11	0.008	2.34	5.06	0.01	ENSMUSG00000035385
Lgals3bp	11	0.008	1.66	3.15	0.01	ENSMUSG00000033880
Slc11a1	1	0.008	1.87	3.65	0.01	ENSMUSG00000026177
Hsbp1l1	18	0.008	3.33	10.07	0.01	ENSMUSG00000078963
Sipa1l2	8	0.008	1.46	2.74	0.01	ENSMUSG00000001995
Nexn	3	0.008	2.36	5.14	0.01	ENSMUSG00000039103
Corin	5	0.008	3.00	7.98	0.01	ENSMUSG00000005220
Klhdc8a	1	0.008	2.54	5.82	0.01	ENSMUSG00000042115
Dse	10	0.008	1.81	3.51	0.01	ENSMUSG00000039497
Uggt2	14	0.008	2.29	4.91	0.01	ENSMUSG00000042104
Mmp23	4	0.008	2.43	5.41	0.01	ENSMUSG00000029061
Edn1	13	0.008	2.83	7.09	0.01	ENSMUSG00000021367
Cdh3	8	0.008	2.03	4.08	0.01	ENSMUSG00000061048
Dbn1	13	0.008	1.86	3.63	0.01	ENSMUSG00000034675

Pdgfrb	18	0.009	1.48	2.79	0.01	ENSMUSG00000024620
N4bp2l1	5	0.009	1.08	2.11	0.01	ENSMUSG00000041132
Pkd2	5	0.009	1.43	2.69	0.01	ENSMUSG00000034462
Insc	7	0.009	3.35	10.21	0.01	ENSMUSG00000048782
Man1c1	4	0.009	1.59	3.01	0.01	ENSMUSG00000037306
Adamts14	10	0.009	2.33	5.03	0.01	ENSMUSG00000059901
Gpr137b	13	0.009	1.45	2.74	0.01	ENSMUSG00000021306
Asb4	6	0.009	2.57	5.95	0.01	ENSMUSG00000042607
Sgk1	10	0.009	1.53	2.88	0.01	ENSMUSG00000019970
Ror1	4	0.009	2.94	7.66	0.01	ENSMUSG00000035305
Ms4a6c	19	0.009	1.72	3.29	0.01	ENSMUSG00000079419
Cc2d2a	5	0.009	2.31	4.97	0.01	ENSMUSG00000039765
Col7a1	9	0.009	1.83	3.56	0.01	ENSMUSG00000025650
Sult5a1	8	0.009	1.71	3.26	0.01	ENSMUSG00000000739
Tlr13	X	0.009	1.78	3.44	0.01	ENSMUSG00000033777
Nfatc4	14	0.009	2.15	4.44	0.01	ENSMUSG00000023411
Pnma2	14	0.009	3.16	8.95	0.01	ENSMUSG00000046204
Gnao1	8	0.010	2.17	4.51	0.01	ENSMUSG00000031748
Limch1	5	0.010	2.26	4.80	0.01	ENSMUSG00000037736
Cdk15	1	0.010	3.56	11.80	0.01	ENSMUSG00000026023
Tspyl5	15	0.010	2.77	6.83	0.01	ENSMUSG00000038984
Ptgfrn	3	0.010	1.68	3.21	0.01	ENSMUSG00000027864
Bmper	9	0.010	2.36	5.15	0.01	ENSMUSG00000031963
Tcaf2	6	0.010	2.38	5.21	0.01	ENSMUSG00000029851
Trpc4	3	0.010	2.85	7.23	0.01	ENSMUSG00000027748
Sulf1	1	0.010	1.07	5.33	0.01	ENSMUSG00000016918
Nrp2	1	0.010	1.16	3.36	0.01	ENSMUSG00000025969
Pid1	1	0.010	4.01	3.95	0.01	ENSMUSG00000045658
Twist2	1	0.010	1.02	5.93	0.01	ENSMUSG00000007805
Mr1	1	0.010	1.56	4.46	0.01	ENSMUSG00000026471
Tnr	1	0.010	1.75	4.56	0.01	ENSMUSG00000015829
Ifi211	1	0.010	1.35	5.26	0.01	ENSMUSG00000026536
Adamtsl2	2	0.010	2.19	7.47	0.01	ENSMUSG00000036040

Abtb2	2	0.010	3.36	2.07	0.01	ENSMUSG00000032724
Zfp334	2	0.010	3.22	4.24	0.01	ENSMUSG00000017667
Prr9	3	0.010	2.27	10.28	0.01	ENSMUSG00000056270
Dpyd	3	0.010	1.72	7.88	0.01	ENSMUSG00000033308
Cnn3	3	0.010	1.19	2.42	0.01	ENSMUSG00000053931
Ttll7	3	0.010	1.66	3.89	0.01	ENSMUSG00000036745
Mmp16	4	0.010	1.42	5.59	0.01	ENSMUSG00000028226
Hrct1	4	0.010	2.48	5.45	0.01	ENSMUSG00000071001
Reck	4	0.010	2.64	3.48	0.01	ENSMUSG00000028476
Mpdz	4	0.010	2.69	2.81	0.01	ENSMUSG00000028402
Plod1	4	0.010	1.69	2.55	0.01	ENSMUSG00000019055
Nppb	4	0.010	3.81	6.41	0.01	ENSMUSG00000029019
Fam114a1	5	0.010	2.42	3.79	0.01	ENSMUSG00000029185
Ttc28	5	0.010	2.44	2.27	0.01	ENSMUSG00000033209
Pdgfa	5	0.010	2.59	3.99	0.01	ENSMUSG00000025856
Akr1b7	6	0.010	3.06	9.29	0.01	ENSMUSG00000052131
Parp12	6	0.010	2.60	2.97	0.01	ENSMUSG00000038507
Dennd2a	6	0.010	1.76	3.69	0.01	ENSMUSG00000038456
Prickle2	6	0.010	2.42	4.82	0.01	ENSMUSG00000030020
Cd4	6	0.010	2.26	77.88	0.01	ENSMUSG00000023274
Mgp	6	0.010	1.12	7.75	0.01	ENSMUSG00000030218
Pla2g4c	7	0.010	2.40	2.32	0.01	ENSMUSG00000033847
Lgi4	7	0.010	1.35	12.59	0.01	ENSMUSG00000036560
Atp10a	7	0.010	1.16	3.16	0.01	ENSMUSG00000025324
Inppl1	7	0.010	1.35	2.23	0.01	ENSMUSG00000032737
Irf7	7	0.010	1.77	2.67	0.01	ENSMUSG00000025498
Osbpl5	7	0.010	1.75	3.27	0.01	ENSMUSG00000037606
Adam9	8	0.010	3.74	2.23	0.01	ENSMUSG00000031555
Lonrf1	8	0.010	2.27	2.08	0.01	ENSMUSG00000039633
Tenm3	8	0.010	2.63	4.91	0.01	ENSMUSG00000031561
Maf	8	0.010	1.80	2.55	0.01	ENSMUSG00000055435
Map10	8	0.010	2.80	5.59	0.01	ENSMUSG00000050930
Cadm1	9	0.010	6.47	2.84	0.01	ENSMUSG00000032076

Il18	9	0.010	2.03	6.72	0.01	ENSMUSG00000039217
Cryab	9	0.010	2.57	3.02	0.01	ENSMUSG00000032060
Unc13c	9	0.010	2.16	6.43	0.01	ENSMUSG00000062151
Htr1b	9	0.010	1.05	6.58	0.01	ENSMUSG00000049511
Parp3	9	0.010	1.17	3.41	0.01	ENSMUSG00000023249
Ltf	9	0.010	2.98	3.37	0.01	ENSMUSG00000032496
Stac	9	0.010	2.45	13.37	0.01	ENSMUSG00000032502
Glt8d2	10	0.010	2.95	12.81	0.01	ENSMUSG00000020251
Osbp2	11	0.010	3.65	4.38	0.01	ENSMUSG00000020435
Adra1b	11	0.010	1.15	9.84	0.01	ENSMUSG00000050541
Sparc	11	0.010	2.50	3.24	0.01	ENSMUSG00000018593
Lypd8	11	0.010	3.68	14.04	0.01	ENSMUSG00000013643
Sat2	11	0.010	2.13	6.42	0.01	ENSMUSG00000069835
Bcl6b	11	0.010	2.68	4.82	0.01	ENSMUSG00000000317
Kif1c	11	0.010	1.96	2.11	0.01	ENSMUSG00000020821
Slfn8	11	0.010	1.60	5.65	0.01	ENSMUSG00000035208
Copz2	11	0.010	2.57	3.88	0.01	ENSMUSG00000018672
Nags	11	0.010	1.97	3.03	0.01	ENSMUSG00000048217
Cbx2	11	0.010	2.01	2.84	0.01	ENSMUSG00000025577
Etv1	12	0.010	2.60	5.93	0.01	ENSMUSG00000004151
Ifi27l2a	12	0.010	1.23	5.36	0.01	ENSMUSG00000079017
Bdkrb2	12	0.010	2.90	5.44	0.01	ENSMUSG00000021070
Gm3453	14	0.010	1.80	16.10	0.01	ENSMUSG00000090643
Nid2	14	0.010	1.92	2.85	0.01	ENSMUSG00000021806
Cfap70	14	0.010	1.18	6.95	0.01	ENSMUSG00000039543
Rbp3	14	0.010	1.97	17.77	0.01	ENSMUSG00000041534
Rnase4	14	0.010	6.28	2.67	0.01	ENSMUSG00000021876
Nynrin	14	0.010	1.22	3.23	0.01	ENSMUSG00000075592
Dzip1	14	0.010	2.53	2.96	0.01	ENSMUSG00000042156
Myo10	15	0.010	1.05	2.27	0.01	ENSMUSG00000022272
Gpr20	15	0.010	1.59	88.70	0.01	ENSMUSG00000045281
Mroh6	15	0.010	1.72	5.27	0.01	ENSMUSG00000098678
Phldb2	16	0.010	2.34	3.62	0.01	ENSMUSG00000033149

Mdga1	17	0.010	1.00	2.59	0.01	ENSMUSG00000043557
9130008F23Rik	17	0.010	2.48	5.02	0.01	ENSMUSG00000054951
Apobec2	17	0.010	2.00	6.06	0.01	ENSMUSG00000040694
Arhgap28	17	0.010	1.71	2.49	0.01	ENSMUSG00000024043
Pcdhgc3	18	0.010	1.08	5.54	0.01	ENSMUSG00000102918
Prdm6	18	0.010	2.40	6.07	0.01	ENSMUSG00000069378
Fam189a2	19	0.010	1.86	5.06	0.01	ENSMUSG00000071604
Dmrt2	19	0.010	1.37	6.71	0.01	ENSMUSG00000048138
Entpd1	19	0.010	1.32	3.38	0.01	ENSMUSG00000048120
Blnk	19	0.010	2.47	5.18	0.01	ENSMUSG00000061132
Plp2	X	0.010	2.37	2.23	0.01	ENSMUSG00000031146
Timp1	X	0.010	1.15	4.08	0.01	ENSMUSG00000001131
Fhl2	1	0.020	1.63	3.51	0.02	ENSMUSG00000008136
Plcl1	1	0.020	1.86	4.57	0.02	ENSMUSG00000038349
Pm20d1	1	0.020	1.61	5.34	0.02	ENSMUSG00000042251
Fcgr4	1	0.020	1.58	3.79	0.02	ENSMUSG00000059089
Fcgr3	1	0.020	2.24	2.02	0.02	ENSMUSG00000059498
Ifi209	1	0.020	3.22	5.50	0.02	ENSMUSG00000043263
Ifi208	1	0.020	1.92	15.59	0.02	ENSMUSG00000066677
Pld5	1	0.020	1.25	11.72	0.02	ENSMUSG00000055214
Rxra	2	0.020	2.17	2.11	0.02	ENSMUSG00000015846
Lcn2	2	0.020	1.58	3.20	0.02	ENSMUSG00000026822
Crb2	2	0.020	3.19	4.58	0.02	ENSMUSG00000035403
Tanc1	2	0.020	3.22	2.41	0.02	ENSMUSG00000035168
Nr1h3	2	0.020	2.13	3.93	0.02	ENSMUSG00000002108
Pax6	2	0.020	1.68	4.49	0.02	ENSMUSG00000027168
Map1a	2	0.020	2.25	2.70	0.02	ENSMUSG00000027254
Mertk	2	0.020	3.06	3.10	0.02	ENSMUSG00000014361
Sirpa	2	0.020	1.22	2.03	0.02	ENSMUSG00000037902
Snta1	2	0.020	2.77	3.34	0.02	ENSMUSG00000027488
Pex5l	3	0.020	2.57	9.01	0.02	ENSMUSG00000027674
Ccdc39	3	0.020	2.37	13.89	0.02	ENSMUSG00000027676
Arhgef26	3	0.020	1.69	4.09	0.02	ENSMUSG00000036885

Slc22a15	3	0.020	1.53	3.01	0.02	ENSMUSG00000033147
Wnt2b	3	0.020	3.34	4.84	0.02	ENSMUSG00000027840
Samd13	3	0.020	2.95	10.57	0.02	ENSMUSG00000048652
Wls	3	0.020	2.11	2.31	0.02	ENSMUSG00000028173
Rusc2	4	0.020	1.41	3.00	0.02	ENSMUSG00000035969
Lpar1	4	0.020	2.62	3.67	0.02	ENSMUSG00000038668
Slc31a2	4	0.020	2.42	2.35	0.02	ENSMUSG00000066152
Orm1	4	0.020	1.36	9.12	0.02	ENSMUSG00000039196
Bnc2	4	0.020	1.97	6.19	0.02	ENSMUSG00000028487
Adamtsl1	4	0.020	1.43	3.62	0.02	ENSMUSG00000066113
Gm13285	4	0.020	1.08	5.33	0.02	ENSMUSG00000095101
Cyp2j6	4	0.020	2.63	4.91	0.02	ENSMUSG00000052914
Cachd1	4	0.020	1.72	2.34	0.02	ENSMUSG00000028532
Ptch2	4	0.020	3.02	5.69	0.02	ENSMUSG00000028681
Bmp8b	4	0.020	3.92	14.12	0.02	ENSMUSG00000002384
Smpdl3b	4	0.020	1.78	4.37	0.02	ENSMUSG00000028885
Sfn	4	0.020	3.22	3.29	0.02	ENSMUSG00000047281
Trim63	4	0.020	1.73	5.19	0.02	ENSMUSG00000028834
Htr1d	4	0.020	3.00	12.57	0.02	ENSMUSG00000070687
Mfap2	4	0.020	1.75	4.88	0.02	ENSMUSG00000060572
Angptl7	4	0.020	2.44	8.09	0.02	ENSMUSG00000028989
Kcnk3	5	0.020	6.24	15.17	0.02	ENSMUSG00000049265
Tlr6	5	0.020	1.56	5.06	0.02	ENSMUSG00000051498
Apbb2	5	0.020	3.76	3.21	0.02	ENSMUSG00000029207
Arhgap24	5	0.020	1.11	4.76	0.02	ENSMUSG00000057315
Spp1	5	0.020	1.93	3.43	0.02	ENSMUSG00000029304
Gbp9	5	0.020	3.05	3.71	0.02	ENSMUSG00000029298
Pla2g1b	5	0.020	1.85	8.35	0.02	ENSMUSG00000029522
Ephb4	5	0.020	3.65	3.45	0.02	ENSMUSG00000029710
Stard13	5	0.020	3.30	2.71	0.02	ENSMUSG00000016128
Tfpi2	6	0.020	1.13	5.72	0.02	ENSMUSG00000029664
Chrm2	6	0.020	6.20	6.62	0.02	ENSMUSG00000045613
Hoxa10	6	0.020	1.63	5.09	0.02	ENSMUSG00000000938

Fkbp14	6	0.020	1.27	3.78	0.02	ENSMUSG00000038074
Aicda	6	0.020	1.78	9.32	0.02	ENSMUSG00000040627
Clec4a3	6	0.020	2.20	6.27	0.02	ENSMUSG00000043832
Six5	7	0.020	2.27	3.47	0.02	ENSMUSG00000040841
Plekhg2	7	0.020	3.82	2.26	0.02	ENSMUSG00000037552
Acp7	7	0.020	1.03	7.99	0.02	ENSMUSG00000037469
Clip3	7	0.020	2.35	3.78	0.02	ENSMUSG00000013921
Sbsn	7	0.020	1.34	3.37	0.02	ENSMUSG00000046056
Wtip	7	0.020	2.85	3.25	0.02	ENSMUSG00000036459
Rcn3	7	0.020	1.88	2.47	0.02	ENSMUSG00000019539
Slco2b1	7	0.020	1.51	2.75	0.02	ENSMUSG00000030737
Arhgef17	7	0.020	4.02	3.32	0.02	ENSMUSG00000032875
Rras2	7	0.020	5.96	2.53	0.02	ENSMUSG00000055723
Tgfb1i1	7	0.020	1.54	2.32	0.02	ENSMUSG00000030782
Mtnr1a	8	0.020	1.27	63.72	0.02	ENSMUSG00000054764
Tlr3	8	0.020	1.74	5.42	0.02	ENSMUSG00000031639
Bst2	8	0.020	2.03	2.61	0.02	ENSMUSG00000046718
Nkd1	8	0.020	6.17	4.50	0.02	ENSMUSG00000031661
Adamts18	8	0.020	3.40	75.79	0.02	ENSMUSG00000053399
Zcchc14	8	0.020	2.37	2.69	0.02	ENSMUSG00000061410
Yap1	9	0.020	2.86	3.29	0.02	ENSMUSG00000053110
Trim29	9	0.020	3.03	6.81	0.02	ENSMUSG00000032013
Cspg4	9	0.020	2.17	3.64	0.02	ENSMUSG00000032911
Fam81a	9	0.020	1.43	6.17	0.02	ENSMUSG00000032224
Myo1e	9	0.020	1.72	2.96	0.02	ENSMUSG00000032220
Tbx18	9	0.020	2.62	6.15	0.02	ENSMUSG00000032419
Pcolce2	9	0.020	1.86	4.11	0.02	ENSMUSG00000015354
Samd5	10	0.020	1.42	4.42	0.02	ENSMUSG00000060487
Adgrg6	10	0.020	1.89	3.62	0.02	ENSMUSG00000039116
Raet1d	10	0.020	2.52	5.94	0.02	ENSMUSG00000078452
Tcf21	10	0.020	1.06	61.08	0.02	ENSMUSG00000045680
Traf3ip2	10	0.020	1.98	3.73	0.02	ENSMUSG00000019842
Mfsd4b3	10	0.020	1.19	7.21	0.02	ENSMUSG00000071335

Ggt5	10	0.020	2.97	4.70	0.02	ENSMUSG00000006344
Aldh1l2	10	0.020	1.46	4.36	0.02	ENSMUSG00000020256
Selenom	11	0.020	1.56	3.00	0.02	ENSMUSG00000075702
Slc35e4	11	0.020	1.73	2.68	0.02	ENSMUSG00000048807
Castor1	11	0.020	1.06	2.95	0.02	ENSMUSG00000020424
Flt4	11	0.020	3.96	4.06	0.02	ENSMUSG00000020357
Pmp22	11	0.020	2.41	2.15	0.02	ENSMUSG00000018217
Hs3st3b1	11	0.020	1.23	3.71	0.02	ENSMUSG00000070407
Cxcl16	11	0.020	2.52	3.38	0.02	ENSMUSG00000018920
Tax1bp3	11	0.020	2.73	2.12	0.02	ENSMUSG00000040158
Rflnb	11	0.020	1.92	2.72	0.02	ENSMUSG00000020846
Slfn9	11	0.020	2.65	2.16	0.02	ENSMUSG00000069793
Skap1	11	0.020	5.99	4.80	0.02	ENSMUSG00000057058
Tubg2	11	0.020	1.38	5.73	0.02	ENSMUSG00000045007
Acot6	12	0.020	1.45	3.82	0.02	ENSMUSG00000043487
Ift43	12	0.020	1.11	3.84	0.02	ENSMUSG00000007867
Ifi27	12	0.020	2.42	3.80	0.02	ENSMUSG00000064215
Itgb8	12	0.020	1.54	8.27	0.02	ENSMUSG00000025321
Hist1h3c	13	0.020	2.00	3.69	0.02	ENSMUSG00000069310
Serpinb9	13	0.020	1.86	2.89	0.02	ENSMUSG00000045827
Serpinb9g	13	0.020	2.70	5.35	0.02	ENSMUSG00000057726
Nrn1	13	0.020	1.81	7.49	0.02	ENSMUSG00000039114
Adamts16	13	0.020	1.89	6.22	0.02	ENSMUSG00000049538
Hexb	13	0.020	1.24	2.01	0.02	ENSMUSG00000021665
Gpx8	13	0.020	1.48	3.94	0.02	ENSMUSG00000021760
Flnb	14	0.020	1.46	2.16	0.02	ENSMUSG00000025278
Dlg5	14	0.020	1.86	2.90	0.02	ENSMUSG00000021782
Grid1	14	0.020	2.04	9.31	0.02	ENSMUSG00000041078
Slc7a7	14	0.020	2.14	2.78	0.02	ENSMUSG00000000958
Scara3	14	0.020	1.94	9.85	0.02	ENSMUSG00000034463
Slitrk6	14	0.020	2.64	7.85	0.02	ENSMUSG00000045871
Laptm4b	15	0.020	2.51	2.89	0.02	ENSMUSG00000022257
Zfpm2	15	0.020	1.54	5.13	0.02	ENSMUSG00000022306

Mal2	15	0.020	2.19	4.94	0.02	ENSMUSG00000024479
Eppk1	15	0.020	1.04	4.01	0.02	ENSMUSG00000115388
Rbfox2	15	0.020	1.92	2.86	0.02	ENSMUSG00000033565
Csdc2	15	0.020	1.68	4.08	0.02	ENSMUSG00000042109
Lrrk2	15	0.020	1.42	4.11	0.02	ENSMUSG00000036273
Scarf2	16	0.020	1.79	3.66	0.02	ENSMUSG00000012017
St6gal1	16	0.020	1.86	2.89	0.02	ENSMUSG00000022885
Meltf	16	0.020	1.57	6.22	0.02	ENSMUSG00000022780
Cd200	16	0.020	1.51	4.57	0.02	ENSMUSG00000022661
Abi3bp	16	0.020	5.93	16.23	0.02	ENSMUSG00000035258
Tiam2	17	0.020	2.26	4.73	0.02	ENSMUSG00000023800
Fgd2	17	0.020	1.65	3.63	0.02	ENSMUSG00000024013
Cyp4f16	17	0.020	1.53	3.43	0.02	ENSMUSG00000048440
Ptchd4	17	0.020	2.19	73.41	0.02	ENSMUSG00000042256
Efna5	17	0.020	1.78	3.70	0.02	ENSMUSG00000048915
Dsc3	18	0.020	1.01	9.32	0.02	ENSMUSG00000059898
Synpo	18	0.020	1.02	3.79	0.02	ENSMUSG00000043079
Lipg	18	0.020	1.59	3.32	0.02	ENSMUSG00000053846
Myrf	19	0.020	3.65	3.12	0.02	ENSMUSG00000036098
Pcsk5	19	0.020	1.70	4.32	0.02	ENSMUSG00000024713
Nkx2-3	19	0.020	1.75	8.26	0.02	ENSMUSG00000044220
Ablim1	19	0.020	2.63	3.27	0.02	ENSMUSG00000025085
Gpc4	X	0.020	1.90	2.38	0.02	ENSMUSG00000031119
Rtl8b	X	0.020	1.30	2.65	0.02	ENSMUSG00000067924
Gabre	X	0.020	2.03	5.70	0.02	ENSMUSG00000031340
Efnb1	X	0.020	1.53	2.90	0.02	ENSMUSG00000031217
Srpx2	X	0.020	1.82	6.50	0.02	ENSMUSG00000031253
Trpc5os	X	0.020	1.64	6.14	0.02	ENSMUSG00000072934
Pir	X	0.020	1.71	2.09	0.02	ENSMUSG00000031379
1500015O10Rik	1	0.030	1.66	47.25	0.03	ENSMUSG00000026051
Raph1	1	0.030	1.43	2.33	0.03	ENSMUSG00000026014
Myl1	1	0.030	5.81	6.72	0.03	ENSMUSG00000061816
Sp100	1	0.030	1.07	3.37	0.03	ENSMUSG00000026222

Glrp1	1	0.030	2.39	6.19	0.03	ENSMUSG00000062310
Sh3bp4	1	0.030	1.40	3.15	0.03	ENSMUSG00000036206
Marco	1	0.030	2.18	4.29	0.03	ENSMUSG00000026390
Gpr161	1	0.030	1.34	3.42	0.03	ENSMUSG00000040836
Pogk	1	0.030	3.06	2.07	0.03	ENSMUSG00000040596
Adamts4	1	0.030	3.66	4.12	0.03	ENSMUSG00000006403
Rgs7	1	0.030	2.13	46.15	0.03	ENSMUSG00000026527
Itga8	2	0.030	2.10	2.96	0.03	ENSMUSG00000026768
Armc3	2	0.030	1.02	46.15	0.03	ENSMUSG00000037683
Ppp1r26	2	0.030	2.64	5.35	0.03	ENSMUSG00000035829
Chst14	2	0.030	5.70	2.70	0.03	ENSMUSG00000074916
Foxs1	2	0.030	2.04	6.10	0.03	ENSMUSG00000074676
Fndc3b	3	0.030	1.42	2.68	0.03	ENSMUSG00000039286
Cd5l	3	0.030	1.71	6.13	0.03	ENSMUSG00000015854
Adam15	3	0.030	2.52	2.20	0.03	ENSMUSG00000028041
Kcnn3	3	0.030	2.64	5.24	0.03	ENSMUSG00000000794
Crct1	3	0.030	1.88	6.40	0.03	ENSMUSG00000027913
Hao2	3	0.030	2.00	46.04	0.03	ENSMUSG00000027870
Gstm2	3	0.030	1.88	3.84	0.03	ENSMUSG00000040562
Tram1l1	3	0.030	1.45	6.14	0.03	ENSMUSG00000044528
Ccl27a	4	0.030	2.14	3.63	0.03	ENSMUSG00000073888
Gm13305	4	0.030	1.86	55.99	0.03	ENSMUSG00000073876
Palm2	4	0.030	1.21	9.97	0.03	ENSMUSG00000090053
Cntln	4	0.030	1.69	2.45	0.03	ENSMUSG00000038070
Guca2b	4	0.030	1.89	6.91	0.03	ENSMUSG00000032978
Asap3	4	0.030	2.55	3.46	0.03	ENSMUSG00000036995
Rap1gap	4	0.030	1.99	3.16	0.03	ENSMUSG00000041351
Fblim1	4	0.030	1.74	3.28	0.03	ENSMUSG00000006219
Errfi1	4	0.030	1.57	2.21	0.03	ENSMUSG00000028967
Steap1	5	0.030	5.63	4.93	0.03	ENSMUSG00000015652
Plb1	5	0.030	5.85	5.73	0.03	ENSMUSG00000029134
Uchl1	5	0.030	1.85	4.06	0.03	ENSMUSG00000029223
Ereg	5	0.030	1.78	49.36	0.03	ENSMUSG00000029377

Tbx5	5	0.030	1.63	57.61	0.03	ENSMUSG00000018263
Oas1a	5	0.030	1.65	3.35	0.03	ENSMUSG00000052776
Hip1	5	0.030	1.90	2.48	0.03	ENSMUSG00000039959
Zcwpw1	5	0.030	1.91	2.47	0.03	ENSMUSG00000037108
Cav2	6	0.030	2.45	3.60	0.03	ENSMUSG00000000058
Wnt16	6	0.030	1.12	46.15	0.03	ENSMUSG00000029671
Creb3l2	6	0.030	1.99	2.51	0.03	ENSMUSG00000038648
Tmsb10	6	0.030	2.39	2.10	0.03	ENSMUSG00000079523
Loxl3	6	0.030	1.01	2.97	0.03	ENSMUSG00000000693
Plxnd1	6	0.030	2.43	2.14	0.03	ENSMUSG00000030123
Bcat1	6	0.030	2.09	3.58	0.03	ENSMUSG00000030268
Rassf8	6	0.030	1.96	3.43	0.03	ENSMUSG00000030259
Amn1	6	0.030	2.77	2.90	0.03	ENSMUSG00000068250
Lair1	7	0.030	2.16	3.69	0.03	ENSMUSG00000055541
Lrfn3	7	0.030	1.64	4.28	0.03	ENSMUSG00000036957
Scn1b	7	0.030	1.82	3.14	0.03	ENSMUSG00000019194
Kctd15	7	0.030	2.07	4.01	0.03	ENSMUSG00000030499
Trpm4	7	0.030	2.61	3.61	0.03	ENSMUSG00000038260
Arpin	7	0.030	2.62	3.74	0.03	ENSMUSG00000039043
Scnn1b	7	0.030	2.72	5.51	0.03	ENSMUSG00000030873
Lpar2	8	0.030	2.02	2.69	0.03	ENSMUSG00000031861
Cdh11	8	0.030	1.47	3.77	0.03	ENSMUSG00000031673
Nol3	8	0.030	1.92	3.76	0.03	ENSMUSG00000014776
Tsnaxip1	8	0.030	1.25	3.79	0.03	ENSMUSG00000031893
Rgl3	9	0.030	1.62	5.23	0.03	ENSMUSG00000040146
Jam3	9	0.030	1.12	3.67	0.03	ENSMUSG00000031990
Adamts8	9	0.030	1.52	5.21	0.03	ENSMUSG00000031994
Arhgap32	9	0.030	1.47	2.37	0.03	ENSMUSG00000041444
Cdon	9	0.030	5.63	3.15	0.03	ENSMUSG00000038119
Kdelc2	9	0.030	2.00	3.07	0.03	ENSMUSG00000034487
Cib2	9	0.030	1.08	4.53	0.03	ENSMUSG00000037493
Islr	9	0.030	1.91	5.04	0.03	ENSMUSG00000037206
Lrrc49	9	0.030	1.37	2.73	0.03	ENSMUSG00000047766

Camp	9	0.030	5.53	3.52	0.03	ENSMUSG00000038357
Tgfbr2	9	0.030	1.05	2.17	0.03	ENSMUSG00000032440
Lyzl4	9	0.030	5.53	10.84	0.03	ENSMUSG00000032530
Perp	10	0.030	5.53	2.14	0.03	ENSMUSG00000019851
E030030I06Rik	10	0.030	1.37	4.16	0.03	ENSMUSG00000097327
Slc18b1	10	0.030	1.14	3.21	0.03	ENSMUSG00000037455
Col6a1	10	0.030	1.86	3.50	0.03	ENSMUSG00000001119
1500009L16Rik	10	0.030	5.53	4.84	0.03	ENSMUSG00000087651
Ptprb	10	0.030	1.57	3.97	0.03	ENSMUSG00000020154
Gabra1	11	0.030	2.10	10.81	0.03	ENSMUSG00000010803
Pdlim4	11	0.030	2.46	4.32	0.03	ENSMUSG00000020388
Wnt9a	11	0.030	1.43	3.63	0.03	ENSMUSG00000000126
Mmp28	11	0.030	2.33	5.25	0.03	ENSMUSG00000020682
Ccl6	11	0.030	1.68	3.42	0.03	ENSMUSG00000018927
Naglu	11	0.030	2.44	2.31	0.03	ENSMUSG00000001751
Slc26a11	11	0.030	1.58	2.88	0.03	ENSMUSG00000039908
Pxdn	12	0.030	1.57	3.00	0.03	ENSMUSG00000020674
Zc2hc1c	12	0.030	1.49	6.77	0.03	ENSMUSG00000045064
Jdp2	12	0.030	3.70	2.94	0.03	ENSMUSG00000034271
Rapgef5	12	0.030	1.86	3.22	0.03	ENSMUSG00000041992
Serpinb6b	13	0.030	5.63	2.53	0.03	ENSMUSG00000042842
Adtrp	13	0.030	1.37	7.80	0.03	ENSMUSG00000058022
Adgrv1	13	0.030	1.08	6.90	0.03	ENSMUSG00000069170
Plpp1	13	0.030	2.96	2.81	0.03	ENSMUSG00000021759
Hcn1	13	0.030	1.41	8.34	0.03	ENSMUSG00000021730
Ptprg	14	0.030	5.56	2.76	0.03	ENSMUSG00000021745
Gdf10	14	0.030	1.77	5.89	0.03	ENSMUSG00000021943
Npy4r	14	0.030	2.61	49.44	0.03	ENSMUSG00000048337
Rnase2a	14	0.030	2.68	12.60	0.03	ENSMUSG00000047222
Adra1a	14	0.030	1.29	4.26	0.03	ENSMUSG00000045875
Gfra2	14	0.030	1.79	3.88	0.03	ENSMUSG00000022103
Ednrb	14	0.030	1.74	4.52	0.03	ENSMUSG00000022122
Sema5a	15	0.030	1.30	4.39	0.03	ENSMUSG00000022231

Nipal2	15	0.030	1.84	4.05	0.03	ENSMUSG00000038879
Cthrc1	15	0.030	2.38	3.70	0.03	ENSMUSG00000054196
Samd12	15	0.030	3.44	12.99	0.03	ENSMUSG00000058656
Tnfrsf11b	15	0.030	1.81	6.81	0.03	ENSMUSG00000063727
Slc38a4	15	0.030	1.77	4.46	0.03	ENSMUSG00000022464
Grasp	15	0.030	1.55	3.62	0.03	ENSMUSG00000000531
Zdhhc8	16	0.030	2.15	2.46	0.03	ENSMUSG00000060166
Gm49333	16	0.030	1.98	12.40	0.03	ENSMUSG00000115293
BC117090	16	0.030	1.91	11.70	0.03	ENSMUSG00000079594
Ip6k3	17	0.030	2.75	49.69	0.03	ENSMUSG00000024210
Abcg1	17	0.030	2.63	2.11	0.03	ENSMUSG00000024030
Tff3	17	0.030	5.52	3.75	0.03	ENSMUSG00000024029
Tap1	17	0.030	1.36	2.58	0.03	ENSMUSG00000037321
Slc25a27	17	0.030	2.79	3.11	0.03	ENSMUSG00000023912
Dync2li1	17	0.030	1.14	3.98	0.03	ENSMUSG00000024253
Snx24	18	0.030	1.31	3.34	0.03	ENSMUSG00000024535
Best1	19	0.030	1.10	4.45	0.03	ENSMUSG00000037418
Dagla	19	0.030	2.06	3.94	0.03	ENSMUSG00000035735
Ms4a7	19	0.030	2.28	3.33	0.03	ENSMUSG00000024672
Ch25h	19	0.030	3.43	7.80	0.03	ENSMUSG00000050370
Tdrd1	19	0.030	2.17	6.07	0.03	ENSMUSG00000025081
Tspan7	X	0.030	2.96	3.77	0.03	ENSMUSG00000058254
Plac1	X	0.030	2.79	5.88	0.03	ENSMUSG00000061082
Spin4	X	0.030	2.56	2.65	0.03	ENSMUSG00000071722
Itm2a	X	0.030	1.76	2.59	0.03	ENSMUSG00000031239
Esx1	X	0.030	2.10	3.38	0.03	ENSMUSG00000023443
Mum1l1	X	0.030	1.82	2.89	0.03	ENSMUSG00000042515
Tbc1d8b	X	0.030	1.74	2.03	0.03	ENSMUSG00000042473
Xkr4	1	0.040	1.83	5.02	0.04	ENSMUSG00000051951
Gulp1	1	0.040	1.33	3.54	0.04	ENSMUSG00000056870
Ugt1a7c	1	0.040	1.43	2.51	0.04	ENSMUSG00000090124
Rbm44	1	0.040	2.11	9.97	0.04	ENSMUSG00000070732
Soat1	1	0.040	1.72	2.06	0.04	ENSMUSG00000026600

Gata3	2	0.040	1.92	3.78	0.04	ENSMUSG00000015619
Slc39a12	2	0.040	1.46	39.72	0.04	ENSMUSG00000036949
Il1rn	2	0.040	1.27	4.50	0.04	ENSMUSG00000026981
Ntng2	2	0.040	1.59	3.25	0.04	ENSMUSG00000035513
Phyhd1	2	0.040	5.54	3.29	0.04	ENSMUSG00000079484
Rnd3	2	0.040	5.55	2.86	0.04	ENSMUSG00000017144
Cd302	2	0.040	1.20	4.16	0.04	ENSMUSG00000060703
Sestd1	2	0.040	1.72	2.78	0.04	ENSMUSG00000042272
Them7	2	0.040	1.49	5.49	0.04	ENSMUSG00000055312
Sqor	2	0.040	1.58	3.51	0.04	ENSMUSG00000005803
Rin2	2	0.040	1.32	2.81	0.04	ENSMUSG00000001768
Atp9a	2	0.040	2.14	2.98	0.04	ENSMUSG00000027546
Hist2h3c1	3	0.040	2.63	2.49	0.04	ENSMUSG00000093769
BC107364	3	0.040	1.48	4.42	0.04	ENSMUSG00000046317
Hsd3b3	3	0.040	1.58	44.21	0.04	ENSMUSG00000062410
Slc6a17	3	0.040	2.58	6.33	0.04	ENSMUSG00000027894
Palmd	3	0.040	1.93	6.20	0.04	ENSMUSG00000033377
Fabp2	3	0.040	5.43	5.60	0.04	ENSMUSG00000023057
Gbp3	3	0.040	2.22	3.86	0.04	ENSMUSG00000028268
Orm3	4	0.040	1.38	39.86	0.04	ENSMUSG00000028359
Artn	4	0.040	1.40	2.29	0.04	ENSMUSG00000028539
Fabp3	4	0.040	1.97	2.76	0.04	ENSMUSG00000028773
Sema3e	5	0.040	2.52	2.70	0.04	ENSMUSG00000063531
Slc5a1	5	0.040	1.56	10.89	0.04	ENSMUSG00000011034
Spon2	5	0.040	2.20	2.99	0.04	ENSMUSG00000037379
Cpz	5	0.040	1.72	3.68	0.04	ENSMUSG00000036596
Evc	5	0.040	5.31	2.48	0.04	ENSMUSG00000029122
Sparcl1	5	0.040	1.07	5.30	0.04	ENSMUSG00000029309
Mfsd7a	5	0.040	1.78	3.81	0.04	ENSMUSG00000029490
Crybb1	5	0.040	1.79	6.20	0.04	ENSMUSG00000029343
Mdfic	6	0.040	1.05	2.93	0.04	ENSMUSG00000041390
Nagk	6	0.040	1.92	2.46	0.04	ENSMUSG00000034744
Anxa4	6	0.040	1.72	2.36	0.04	ENSMUSG00000029994

Sox5	6	0.040	1.81	3.99	0.04	ENSMUSG00000041540
Saa3	7	0.040	2.41	3.30	0.04	ENSMUSG00000040026
Pde2a	7	0.040	1.24	4.69	0.04	ENSMUSG00000110195
Sbf2	7	0.040	2.23	2.40	0.04	ENSMUSG00000038371
Tnfrsf23	7	0.040	1.53	2.82	0.04	ENSMUSG00000037613
Npy1r	8	0.040	1.73	4.57	0.04	ENSMUSG00000036437
Slc27a1	8	0.040	1.64	2.65	0.04	ENSMUSG00000031808
Large1	8	0.040	1.63	2.51	0.04	ENSMUSG00000004383
Kctd19	8	0.040	5.31	6.26	0.04	ENSMUSG00000051648
Casp1	9	0.040	1.58	3.87	0.04	ENSMUSG00000025888
Chrnb4	9	0.040	2.36	42.17	0.04	ENSMUSG00000035200
Neil1	9	0.040	2.02	2.84	0.04	ENSMUSG00000032298
Cgas	9	0.040	1.69	2.39	0.04	ENSMUSG00000032344
Foxl2	9	0.040	2.33	9.09	0.04	ENSMUSG00000050397
Hebp2	10	0.040	3.32	3.92	0.04	ENSMUSG00000019853
Pde7b	10	0.040	5.31	3.65	0.04	ENSMUSG00000019990
Gm48551	10	0.040	2.06	2.74	0.04	ENSMUSG00000113262
Csrp2	10	0.040	2.66	2.48	0.04	ENSMUSG00000020186
Aebp1	11	0.040	1.95	3.44	0.04	ENSMUSG00000020473
Zbtb4	11	0.040	5.32	2.54	0.04	ENSMUSG00000018750
Doc2b	11	0.040	3.44	5.75	0.04	ENSMUSG00000020848
Slfn2	11	0.040	2.86	2.58	0.04	ENSMUSG00000072620
Krt13	11	0.040	1.30	36.66	0.04	ENSMUSG00000044041
Jup	11	0.040	2.00	2.20	0.04	ENSMUSG00000001552
Plcd3	11	0.040	1.27	2.88	0.04	ENSMUSG00000020937
Ttyh2	11	0.040	1.33	2.41	0.04	ENSMUSG00000034714
Gprc5c	11	0.040	1.95	2.95	0.04	ENSMUSG00000051043
Cd300c2	11	0.040	5.40	3.32	0.04	ENSMUSG00000044811
Dact1	12	0.040	1.50	3.11	0.04	ENSMUSG00000044548
Hhipl1	12	0.040	1.61	2.95	0.04	ENSMUSG00000021260
Hist1h4c	13	0.040	1.87	3.09	0.04	ENSMUSG00000060678
Serpinb9f	13	0.040	1.88	4.61	0.04	ENSMUSG00000038327
Ror2	13	0.040	2.51	5.68	0.04	ENSMUSG00000021464

Plac9a	14	0.040	5.50	45.40	0.04	ENSMUSG00000095304
Gdf2	14	0.040	1.71	4.75	0.04	ENSMUSG00000072625
Ephx2	14	0.040	1.96	3.30	0.04	ENSMUSG00000022040
Amigo2	15	0.040	1.52	3.28	0.04	ENSMUSG00000048218
Hes1	16	0.040	1.32	2.52	0.04	ENSMUSG00000022528
Igsf11	16	0.040	2.46	3.88	0.04	ENSMUSG00000022790
Mmp25	17	0.040	1.88	9.09	0.04	ENSMUSG00000023903
Rsph1	17	0.040	1.31	39.73	0.04	ENSMUSG00000024033
Cbs	17	0.040	2.63	3.85	0.04	ENSMUSG00000024039
H2-Ab1	17	0.040	1.09	3.01	0.04	ENSMUSG00000073421
Aif1	17	0.040	1.33	4.79	0.04	ENSMUSG00000024397
Mymx	17	0.040	3.01	46.54	0.04	ENSMUSG00000079471
Srd5a2	17	0.040	2.19	9.97	0.04	ENSMUSG00000038541
Ston1	17	0.040	1.25	2.99	0.04	ENSMUSG00000033855
Colec12	18	0.040	1.88	2.28	0.04	ENSMUSG00000036103
Sh3rf2	18	0.040	5.20	4.06	0.04	ENSMUSG00000057719
Ppic	18	0.040	2.25	2.10	0.04	ENSMUSG00000024538
Megf10	18	0.040	1.87	3.44	0.04	ENSMUSG00000024593
Fbn2	18	0.040	2.26	3.28	0.04	ENSMUSG00000024598
Aldh3b1	19	0.040	1.09	3.12	0.04	ENSMUSG00000024885
Ms4a6b	19	0.040	2.17	3.46	0.04	ENSMUSG00000024677
Il33	19	0.040	1.47	5.13	0.04	ENSMUSG00000024810
Papss2	19	0.040	2.70	2.12	0.04	ENSMUSG00000024899
Plce1	19	0.040	3.18	3.64	0.04	ENSMUSG00000024998
Afap1l2	19	0.040	1.37	4.40	0.04	ENSMUSG00000025083
Rtl8a	X	0.040	1.33	2.49	0.04	ENSMUSG00000067925
Stard8	X	0.040	1.39	2.13	0.04	ENSMUSG00000031216
Fam178b	1	0.050	1.18	4.19	0.05	ENSMUSG00000046337
Mixl1	1	0.050	2.14	36.46	0.05	ENSMUSG00000026497
Eng	2	0.050	1.95	2.56	0.05	ENSMUSG00000026814
Lypd6	2	0.050	1.54	3.42	0.05	ENSMUSG00000050447
Ryr3	2	0.050	2.61	11.16	0.05	ENSMUSG00000057378
Grem1	2	0.050	2.07	4.98	0.05	ENSMUSG00000074934

Cldn11	3	0.050	5.21	36.81	0.05	ENSMUSG00000037625
Fam198b	3	0.050	1.35	2.27	0.05	ENSMUSG00000027955
Paqr6	3	0.050	1.78	2.59	0.05	ENSMUSG00000041423
Cd160	3	0.050	2.32	10.10	0.05	ENSMUSG00000038304
Cttnbp2nl	3	0.050	5.28	2.67	0.05	ENSMUSG00000062127
Arsj	3	0.050	5.20	10.73	0.05	ENSMUSG00000046561
Col24a1	3	0.050	3.34	4.45	0.05	ENSMUSG00000028197
Pakap	4	0.050	3.42	4.66	0.05	ENSMUSG00000089945
Padi4	4	0.050	2.54	5.48	0.05	ENSMUSG00000025330
Cpa4	6	0.050	3.18	9.09	0.05	ENSMUSG00000039070
Tmem176b	6	0.050	1.43	2.70	0.05	ENSMUSG00000029810
Antxr1	6	0.050	1.54	4.40	0.05	ENSMUSG00000033420
P3h3	6	0.050	5.26	3.43	0.05	ENSMUSG00000023191
Plaur	7	0.050	2.31	2.37	0.05	ENSMUSG00000046223
Tmem86a	7	0.050	1.52	2.91	0.05	ENSMUSG00000010307
Nr2f2	7	0.050	2.09	3.56	0.05	ENSMUSG00000030551
Cyb5r2	7	0.050	2.10	38.39	0.05	ENSMUSG00000048065
Syt17	7	0.050	1.14	5.14	0.05	ENSMUSG00000058420
Slc5a2	7	0.050	2.17	2.83	0.05	ENSMUSG00000030781
2210011C24Rik	8	0.050	2.03	3.85	0.05	ENSMUSG00000074217
Plod2	9	0.050	1.23	2.51	0.05	ENSMUSG00000032374
Clec10a	11	0.050	1.37	2.83	0.05	ENSMUSG00000000318
Gsdma	11	0.050	2.22	2.91	0.05	ENSMUSG00000017204
Hist1h2bl	13	0.050	2.45	4.94	0.05	ENSMUSG00000094338
Sox4	13	0.050	1.83	2.14	0.05	ENSMUSG00000076431
Ogdhl	14	0.050	1.50	2.87	0.05	ENSMUSG00000021913
Klhl33	14	0.050	1.50	9.09	0.05	ENSMUSG00000090799
Mafa	15	0.050	1.10	4.24	0.05	ENSMUSG00000047591
Ddr1	17	0.050	1.45	2.74	0.05	ENSMUSG00000003534
Capn11	17	0.050	2.66	6.33	0.05	ENSMUSG00000058626
Plekhh2	17	0.050	2.90	4.28	0.05	ENSMUSG00000040852
Rhoq	17	0.050	2.23	2.21	0.05	ENSMUSG00000024143
Ankrd29	18	0.050	1.20	4.51	0.05	ENSMUSG00000057766

Cabyr	18	0.050	1.04	4.08	0.05	ENSMUSG00000024430
Cyp2c70	19	0.050	3.48	7.47	0.05	ENSMUSG00000060613
DXBay18	X	0.050	1.25	4.69	0.05	ENSMUSG00000071745
Nhsl2	X	0.050	1.33	2.34	0.05	ENSMUSG00000079481
Armcx2	X	0.050	2.12	2.30	0.05	ENSMUSG00000033436

Supplemental Table 11. Downregulated genes in *Dot1L-* MM ESRE cells that are unique to these mutant cells (n=130)

Name	Chromosome	Max group mean	Log_2 fold change	Fold change	P-value	ENSEMBL
Ptprj	2	9.630	-1.23	-2.34	0.00	ENSMUSG00000025314
Elovl6	3	11.510	-1.35	-2.55	0.00	ENSMUSG00000041220
Pf4	5	212.790	-1.67	-3.17	0.00	ENSMUSG00000029373
Itga2b	11	82.050	-1.43	-2.69	0.00	ENSMUSG00000034664
Ctla2a	13	54.440	-1.56	-2.95	0.00	ENSMUSG00000044258
Shank3	15	1.750	-1.87	-3.66	0.00	ENSMUSG00000022623
Ptpn7	1	21.750	-1.14	-2.21	0.00	ENSMUSG00000031506
Kit	5	26.840	-1.20	-2.30	0.00	ENSMUSG00000005672
Mpig6b	17	3.270	-2.25	-4.75	0.00	ENSMUSG00000073414
Gm45837	7	0.290	-5.74	-53.48	0.00	ENSMUSG00000030653
Gbx2	1	0.550	-3.76	-13.52	0.00	ENSMUSG00000034486
Ubash3b	9	6.190	-1.33	-2.52	0.00	ENSMUSG00000032020
Pecam1	11	3.750	-1.74	-3.33	0.00	ENSMUSG00000020717
Ly6g6f	17	6.080	-2.36	-5.15	0.00	ENSMUSG00000034923
Slc6a4	11	19.190	-1.45	-2.74	0.00	ENSMUSG00000020838
Pfkl	10	38.990	-1.00	-2.01	0.00	ENSMUSG00000020277
Clec9a	6	0.330	-3.42	-10.74	0.00	ENSMUSG00000046080
Gabrg3	7	0.090	-3.52	-11.46	0.00	ENSMUSG00000055026
St3gal6	16	11.420	-1.01	-2.01	0.00	ENSMUSG00000022747
Ldha	7	222.860	-1.10	-2.15	0.00	ENSMUSG00000063229
Gnaz	10	4.390	-1.56	-2.94	0.00	ENSMUSG00000040009
Rgs10	7	30.400	-1.27	-2.42	0.00	ENSMUSG00000030844
Gipr	7	1.170	-2.91	-7.49	0.00	ENSMUSG00000030406
Ptpre	7	3.080	-1.43	-2.70	0.00	ENSMUSG00000041836
Ankrd28	14	6.090	-1.06	-2.08	0.00	ENSMUSG00000014496
Gm28048	11	1.090	-2.43	-5.38	0.00	ENSMUSG00000098650
Adra2a	19	1.020	-2.09	-4.26	0.00	ENSMUSG00000033717
Rnf165	18	0.140	-3.85	-14.39	0.00	ENSMUSG00000025427
Tuba8	6	5.370	-1.54	-2.91	0.00	ENSMUSG00000030137
Polr2k	15	21.480	-1.60	-3.02	0.00	ENSMUSG00000045996

Mid1	X	3.860	-1.74	-3.33	0.00	ENSMUSG00000035299
Rab27b	18	5.310	-1.25	-2.38	0.00	ENSMUSG00000024511
Gpatch4	3	17.580	-1.06	-2.09	0.00	ENSMUSG00000028069
Galk1	11	34.290	-1.05	-2.07	0.01	ENSMUSG00000020766
Cd96	16	0.240	-3.28	-9.69	0.01	ENSMUSG00000022657
Tie1	4	1.050	-2.01	-4.04	0.01	ENSMUSG00000033191
Icam2	11	2.690	-1.93	-3.81	0.01	ENSMUSG00000001029
Pkm	9	215.600	-1.05	-2.08	0.01	ENSMUSG00000032294
Zic1	9	0.150	-7.08	-135.00	0.01	ENSMUSG00000032368
Acap1	11	7.150	-1.21	-2.32	0.01	ENSMUSG00000001588
Gbe1	16	3.580	-1.24	-2.35	0.01	ENSMUSG00000022707
Arhgap6	X	1.840	-1.28	-2.43	0.01	ENSMUSG00000031355
Mettl1	10	11.840	-1.06	-2.08	0.01	ENSMUSG00000006732
Il10ra	9	7.380	-1.20	-2.29	0.01	ENSMUSG00000032089
Cd34	1	7.690	-1.19	-2.29	0.01	ENSMUSG00000016494
Susd1	4	7.310	-1.04	-2.06	0.01	ENSMUSG00000038578
Otop1	5	0.170	-3.77	-13.61	0.01	ENSMUSG00000051596
Tesc	5	4.940	-1.69	-3.23	0.01	ENSMUSG00000029359
Akr1b3	6	64.620	-1.10	-2.14	0.01	ENSMUSG00000001642
Gimap6	6	0.590	-2.17	-4.50	0.01	ENSMUSG00000047867
Gm3055	10	0.080	-3.21	-9.22	0.01	ENSMUSG00000094622
Ctla2b	13	4.380	-1.68	-3.20	0.01	ENSMUSG00000074874
D16Ertd472e	16	1.850	-1.33	-2.51	0.01	ENSMUSG00000022864
Pglyrp2	17	0.240	-2.59	-6.00	0.01	ENSMUSG00000079563
Kif21b	1	3.850	-1.14	-2.21	0.02	ENSMUSG00000041642
Rgs18	1	11.240	-1.08	-2.12	0.02	ENSMUSG00000026357
Cass4	2	0.530	-1.85	-3.61	0.02	ENSMUSG00000074570
P2ry1	3	5.160	-1.02	-2.03	0.02	ENSMUSG00000027765
Elavl4	4	0.100	-3.67	-12.72	0.02	ENSMUSG00000028546
Zfp534	4	0.300	-2.31	-4.95	0.02	ENSMUSG00000062518
Ajap1	4	1.120	-2.20	-4.59	0.02	ENSMUSG00000039546
Cxcl5	5	0.280	-2.66	-6.33	0.02	ENSMUSG00000029371
Ctnna2	6	0.080	-4.38	-20.75	0.02	ENSMUSG00000063063

Txnrd3	6	5.220	-1.06	-2.08	0.02	ENSMUSG00000000811
Nova2	7	0.160	-2.92	-7.58	0.02	ENSMUSG00000030411
F10	8	2.420	-1.46	-2.75	0.02	ENSMUSG00000031444
Tma16	8	5.160	-1.03	-2.05	0.02	ENSMUSG00000025591
Gm10282	8	15.270	-1.25	-2.38	0.02	ENSMUSG00000070713
Gm6793	8	13.360	-1.02	-2.02	0.02	ENSMUSG00000092086
Kcnj5	9	0.640	-1.65	-3.14	0.02	ENSMUSG00000032034
Timm9	12	5.250	-1.01	-2.01	0.02	ENSMUSG00000021079
Vash1	12	1.980	-1.58	-2.99	0.02	ENSMUSG00000021256
Mtx3	13	1.660	-1.31	-2.48	0.02	ENSMUSG00000021704
Ltb4r2	14	2.130	-1.86	-3.62	0.02	ENSMUSG00000040432
Rab26	17	0.120	-2.97	-7.83	0.02	ENSMUSG00000079657
Nrxn2	19	0.140	-2.63	-6.21	0.02	ENSMUSG00000033768
Tmem223	19	15.490	-1.21	-2.31	0.02	ENSMUSG00000117924
Lypd1	1	1.280	-1.34	-2.53	0.03	ENSMUSG00000026344
6820408C15Rik	2	0.190	-2.75	-6.75	0.03	ENSMUSG00000032680
Gm14440	2	0.090	-5.64	-49.97	0.03	ENSMUSG00000078901
Rhoh	5	0.180	-1.92	-3.79	0.03	ENSMUSG00000029204
Fam69a	5	4.670	-1.05	-2.07	0.03	ENSMUSG00000029270
Ttyh1	7	0.080	-5.77	-54.48	0.03	ENSMUSG00000030428
Mcf2l	8	0.400	-1.62	-3.07	0.03	ENSMUSG00000031442
Rab27a	9	2.600	-1.31	-2.48	0.03	ENSMUSG00000032202
Adora2a	10	0.760	-1.62	-3.08	0.03	ENSMUSG00000020178
Sez6	11	0.080	-5.77	-54.48	0.03	ENSMUSG00000000632
Fam169a	13	3.000	-1.01	-2.01	0.03	ENSMUSG00000041817
Arl15	13	2.260	-1.34	-2.53	0.03	ENSMUSG00000042348
Rnf219	14	1.980	-1.29	-2.45	0.03	ENSMUSG00000022120
Ciita	16	0.030	-3.47	-11.11	0.03	ENSMUSG00000022504
Reep2	18	1.200	-1.70	-3.26	0.03	ENSMUSG00000038555
Trub1	19	2.770	-1.08	-2.12	0.03	ENSMUSG00000025086
Stk26	X	1.910	-1.39	-2.62	0.03	ENSMUSG00000031112
Susd4	1	0.870	-1.48	-2.79	0.04	ENSMUSG00000038576
Dusp2	2	1.350	-1.62	-3.08	0.04	ENSMUSG00000027368

Vstm2l	2	0.250	-3.88	-14.71	0.04	ENSMUSG00000037843
Ada	2	6.520	-1.01	-2.01	0.04	ENSMUSG00000017697
Gpr171	3	0.280	-2.07	-4.19	0.04	ENSMUSG00000050075
Actl6b	5	0.160	-5.51	-45.55	0.04	ENSMUSG00000029712
Gimap5	6	0.180	-2.64	-6.24	0.04	ENSMUSG00000043505
Lhfpl4	6	0.080	-2.32	-5.00	0.04	ENSMUSG00000042873
Tnnt1	7	0.910	-1.73	-3.32	0.04	ENSMUSG00000064179
Ptgir	7	1.340	-1.40	-2.64	0.04	ENSMUSG00000043017
Pagr1b	7	1.370	-2.44	-5.43	0.04	ENSMUSG00000092534
Prr36	8	0.140	-2.62	-6.13	0.04	ENSMUSG00000064125
Arhgap18	10	2.840	-1.05	-2.08	0.04	ENSMUSG00000039031
Tet1	10	0.630	-1.25	-2.37	0.04	ENSMUSG00000047146
Atp23	10	3.630	-1.37	-2.58	0.04	ENSMUSG00000025436
Camkk1	11	1.070	-1.47	-2.77	0.04	ENSMUSG00000020785
Znhit3	11	12.780	-1.06	-2.08	0.04	ENSMUSG00000020526
Cuedc1	11	3.880	-1.07	-2.10	0.04	ENSMUSG00000018378
Acr	15	0.140	-5.27	-38.72	0.04	ENSMUSG00000022622
Adcy9	16	2.400	-1.02	-2.03	0.04	ENSMUSG00000005580
Bex6	16	0.210	-5.19	-36.49	0.04	ENSMUSG00000075269
Gng3	19	0.170	-3.62	-12.31	0.04	ENSMUSG00000071658
Il2rg	X	7.820	-1.07	-2.10	0.04	ENSMUSG00000031304
P2ry10	X	0.170	-1.96	-3.90	0.04	ENSMUSG00000050921
mt-Nd6	MT	46.280	-1.06	-2.09	0.04	ENSMUSG00000064368
Gm9833	3	0.140	-5.50	-45.14	0.05	ENSMUSG00000049230
Kirrel2	7	0.200	-2.43	-5.37	0.05	ENSMUSG00000036915
Il20rb	9	0.900	-1.44	-2.71	0.05	ENSMUSG00000044244
Adat2	10	6.080	-1.16	-2.23	0.05	ENSMUSG00000019808
Mgat5b	11	0.060	-5.44	-43.32	0.05	ENSMUSG00000043857
Unc79	12	0.040	-3.17	-9.00	0.05	ENSMUSG00000021198
Mccc2	13	4.100	-1.13	-2.19	0.05	ENSMUSG00000021646
Rnf180	13	0.320	-1.56	-2.95	0.05	ENSMUSG00000021720
Ranbp3l	15	0.050	-5.19	-36.56	0.05	ENSMUSG00000048424
Kcng2	18	0.520	-1.70	-3.24	0.05	ENSMUSG00000059852

Pank1	19	0.960	-1.19	-2.29	0.05	ENSMUSG00000033610

Supplemental Table 12. Upregulated genes in *Dot1L*-MM ESRE cells compared to *Dot1L*-KO cells (n=233)

Name	Chromosome	Max group mean	Log_2 fold change	Fold change	P-value	ENSEMBL
Tubb6	18	11.96	1.00	2.00	0.05	ENSMUSG00000001473
App	16	14.25	1.00	2.00	0.01	ENSMUSG00000022892
Ehd2	7	7.93	1.01	2.01	0.04	ENSMUSG00000074364
Tcirg1	19	5.14	1.03	2.05	0.02	ENSMUSG00000001750
Tcaf1	6	1.52	1.05	2.06	0.05	ENSMUSG00000036667
Rnaset2b	17	3.41	1.04	2.06	0.05	ENSMUSG00000094724
Arhgap30	1	2.67	1.04	2.06	0.04	ENSMUSG00000048865
Tead1	7	2.78	1.05	2.07	0.05	ENSMUSG00000055320
Cd36	5	5.31	1.06	2.08	0.02	ENSMUSG00000002944
Axl	7	12.61	1.06	2.09	0.04	ENSMUSG00000002602
Cpe	8	7.57	1.07	2.10	0.05	ENSMUSG00000037852
Ckb	12	8.40	1.08	2.12	0.05	ENSMUSG00000001270
mt-Atp8	MT	686.88	1.09	2.12	0.01	ENSMUSG00000064356
Ece1	4	6.23	1.09	2.13	0.02	ENSMUSG00000057530
Rnf213	11	4.26	1.09	2.14	0.00	ENSMUSG00000070327
Tyrobp	7	22.40	1.11	2.15	0.04	ENSMUSG00000030579
Zcchc24	14	2.46	1.12	2.17	0.05	ENSMUSG00000055538
Igfbp2	1	19.24	1.12	2.17	0.05	ENSMUSG00000039323
Clgn	8	1.72	1.14	2.20	0.05	ENSMUSG00000002190
Fscn1	5	14.96	1.15	2.21	0.03	ENSMUSG00000029581
Wnt4	4	2.96	1.16	2.23	0.03	ENSMUSG00000036856
Adamts9	6	2.06	1.16	2.23	0.02	ENSMUSG00000030022
Irgm1	11	11.92	1.16	2.23	0.01	ENSMUSG00000046879
Lpl	8	14.90	1.16	2.24	0.00	ENSMUSG00000015568
Fbln2	6	26.96	1.17	2.25	0.01	ENSMUSG00000064080
Ugt1a7c	1	1.50	1.19	2.28	0.05	ENSMUSG00000090124
Rcn3	7	15.57	1.19	2.28	0.02	ENSMUSG00000019539
Sulf2	2	4.31	1.20	2.29	0.04	ENSMUSG00000006800
Cd300a	11	1.23	1.20	2.30	0.05	ENSMUSG00000034652
Snrpn	7	5.01	1.20	2.30	0.03	ENSMUSG00000102252

Cd84	1	1.24	1.21	2.31	0.04	ENSMUSG00000038147
Abcd2	15	1.29	1.21	2.31	0.04	ENSMUSG00000055782
Tgfb1i1	7	3.95	1.21	2.31	0.01	ENSMUSG00000030782
Ifit1	19	7.01	1.21	2.32	0.01	ENSMUSG00000034459
Rab7b	1	1.66	1.22	2.33	0.05	ENSMUSG00000052688
Itgb5	16	11.09	1.22	2.33	0.02	ENSMUSG00000022817
Fbn1	2	7.33	1.22	2.33	0.01	ENSMUSG00000027204
Slfn5	11	1.24	1.23	2.34	0.04	ENSMUSG00000054404
Irf7	7	6.10	1.23	2.34	0.02	ENSMUSG00000025498
Csrp2	10	4.37	1.24	2.36	0.04	ENSMUSG00000020186
Clca3a1	3	1.93	1.25	2.37	0.05	ENSMUSG00000056025
Hmga2	10	11.79	1.25	2.37	0.01	ENSMUSG00000056758
Mfhas1	8	6.67	1.25	2.37	0.01	ENSMUSG00000070056
Dcn	10	16.61	1.25	2.38	0.04	ENSMUSG00000019929
Dtx4	19	1.36	1.26	2.39	0.04	ENSMUSG00000039982
Ccdc102a	8	2.26	1.26	2.40	0.05	ENSMUSG00000063605
Lrp1	10	10.84	1.26	2.40	0.01	ENSMUSG00000040249
Elk3	10	1.61	1.27	2.41	0.05	ENSMUSG00000008398
Unc93b1	19	4.65	1.27	2.41	0.04	ENSMUSG00000036908
Serpinb9	13	1.91	1.28	2.43	0.03	ENSMUSG00000045827
Ddr2	1	1.01	1.29	2.45	0.05	ENSMUSG00000026674
Myl9	2	19.65	1.29	2.45	0.04	ENSMUSG00000067818
Mertk	2	2.11	1.30	2.47	0.04	ENSMUSG00000014361
Timp2	11	14.59	1.31	2.47	0.00	ENSMUSG00000017466
Epb41l3	17	2.18	1.31	2.48	0.04	ENSMUSG00000024044
Mrc1	2	4.10	1.31	2.48	0.02	ENSMUSG00000026712
Myrf	19	1.49	1.31	2.49	0.04	ENSMUSG00000036098
Ifitm3	7	13.55	1.31	2.49	0.04	ENSMUSG00000025492
Crispld2	8	4.86	1.33	2.51	0.01	ENSMUSG00000031825
Stab1	14	4.80	1.32	2.51	0.01	ENSMUSG00000042286
Wls	3	5.48	1.33	2.52	0.01	ENSMUSG00000028173
Tagln	9	69.27	1.34	2.53	0.03	ENSMUSG00000032085
Col5a1	2	55.55	1.35	2.55	0.00	ENSMUSG00000026837

Sirpa	2	8.34	1.35	2.55	0.00	ENSMUSG00000037902
Ldlrad3	2	1.13	1.36	2.57	0.05	ENSMUSG00000048058
Oas3	5	1.04	1.37	2.58	0.05	ENSMUSG00000032661
Tmem184a	5	6.43	1.37	2.58	0.00	ENSMUSG00000036687
Map6	7	1.34	1.37	2.59	0.04	ENSMUSG00000055407
Vcan	13	1.91	1.38	2.60	0.04	ENSMUSG00000021614
Pmepa1	2	2.57	1.38	2.61	0.04	ENSMUSG00000038400
Serpine2	1	11.87	1.39	2.62	0.01	ENSMUSG00000026249
Fkbp10	11	11.98	1.39	2.62	0.01	ENSMUSG00000001555
Msrb3	10	1.99	1.40	2.64	0.03	ENSMUSG00000051236
Cyp1b1	17	2.60	1.40	2.64	0.03	ENSMUSG00000024087
Dpysl3	18	1.62	1.41	2.65	0.05	ENSMUSG00000024501
Fstl3	10	4.49	1.41	2.65	0.03	ENSMUSG00000020325
Cpne5	17	2.69	1.41	2.66	0.01	ENSMUSG00000024008
C5ar1	7	1.92	1.42	2.67	0.03	ENSMUSG00000049130
Col5a2	1	19.35	1.42	2.67	0.01	ENSMUSG00000026042
Parp12	6	3.09	1.42	2.68	0.01	ENSMUSG00000038507
Zdbf2	1	1.29	1.42	2.68	0.01	ENSMUSG00000027520
Nrp1	8	6.07	1.43	2.70	0.02	ENSMUSG00000025810
Col4a5	X	3.75	1.43	2.70	0.01	ENSMUSG00000031274
Glipr1	10	9.99	1.44	2.72	0.01	ENSMUSG00000056888
Itm2a	X	6.81	1.45	2.74	0.02	ENSMUSG00000031239
Rhoj	12	1.02	1.47	2.76	0.03	ENSMUSG00000046768
Gpc4	X	7.87	1.47	2.76	0.00	ENSMUSG00000031119
Thy1	9	1.06	1.47	2.78	0.05	ENSMUSG00000032011
Slfn2	11	4.28	1.49	2.80	0.02	ENSMUSG00000072620
Ncf1	5	3.12	1.49	2.81	0.01	ENSMUSG00000015950
Sox4	13	3.73	1.49	2.81	0.00	ENSMUSG00000076431
Ncam1	9	6.29	1.49	2.81	0.00	ENSMUSG00000039542
Loxl2	14	35.01	1.50	2.82	0.01	ENSMUSG00000034205
C3ar1	6	7.54	1.50	2.82	0.00	ENSMUSG00000040552
Ndn	7	2.32	1.50	2.83	0.02	ENSMUSG00000033585
Pcolce	5	9.65	1.51	2.85	0.01	ENSMUSG00000029718

Amotl1	9	4.19	1.52	2.86	0.00	ENSMUSG00000013076
Jph2	2	3.60	1.52	2.87	0.01	ENSMUSG00000017817
C1qa	4	21.12	1.52	2.87	0.01	ENSMUSG00000036887
Tmem119	5	1.56	1.53	2.88	0.04	ENSMUSG00000054675
Gas6	8	38.72	1.55	2.92	0.00	ENSMUSG00000031451
Irf8	8	1.04	1.55	2.93	0.05	ENSMUSG00000041515
Aff2	X	1.27	1.57	2.98	0.02	ENSMUSG00000031189
Lgals3bp	11	10.23	1.58	2.98	0.01	ENSMUSG00000033880
Mrc2	11	2.59	1.59	3.01	0.01	ENSMUSG00000020695
Gpx7	4	2.13	1.61	3.05	0.05	ENSMUSG00000028597
Csf1	3	5.42	1.61	3.06	0.00	ENSMUSG00000014599
Cald1	6	19.57	1.61	3.06	0.00	ENSMUSG00000029761
Atp10a	7	1.34	1.62	3.08	0.01	ENSMUSG00000025324
Slc37a2	9	1.89	1.62	3.08	0.01	ENSMUSG00000032122
Vgll3	16	2.18	1.64	3.11	0.00	ENSMUSG00000091243
Dennd2a	6	1.06	1.65	3.13	0.02	ENSMUSG00000038456
Scrn1	6	1.14	1.64	3.13	0.01	ENSMUSG00000019124
Fstl1	16	35.70	1.65	3.14	0.00	ENSMUSG00000022816
Azin2	4	1.07	1.65	3.15	0.03	ENSMUSG00000028789
Igfbp7	5	30.90	1.66	3.17	0.00	ENSMUSG00000036256
Lyz2	10	11.09	1.66	3.17	0.00	ENSMUSG00000069516
Mmp23	4	1.66	1.67	3.19	0.05	ENSMUSG00000029061
Nradd	9	1.15	1.69	3.22	0.05	ENSMUSG00000032491
Cd53	3	2.33	1.69	3.22	0.01	ENSMUSG00000040747
Adam19	11	4.99	1.69	3.22	0.00	ENSMUSG00000011256
Hrct1	4	1.00	1.71	3.28	0.05	ENSMUSG00000071001
Ifi204	1	1.77	1.72	3.29	0.02	ENSMUSG00000073489
Fzd2	11	2.42	1.73	3.31	0.01	ENSMUSG00000050288
Ptk7	17	2.62	1.73	3.32	0.02	ENSMUSG00000023972
Hp	8	2.95	1.73	3.32	0.02	ENSMUSG00000031722
Cxcl12	6	6.17	1.74	3.34	0.00	ENSMUSG00000061353
Nuak1	10	2.75	1.75	3.35	0.00	ENSMUSG00000020032
Ccn4	15	7.85	1.75	3.36	0.00	ENSMUSG00000005124

Tmem86a	7	1.95	1.75	3.37	0.02	ENSMUSG00000010307
Ms4a7	19	1.43	1.76	3.38	0.03	ENSMUSG00000024672
Lsp1	7	1.62	1.77	3.40	0.02	ENSMUSG00000018819
Adam8	7	6.90	1.76	3.40	0.00	ENSMUSG00000025473
Plagl1	10	23.72	1.77	3.40	0.00	ENSMUSG00000019817
Itgam	7	1.63	1.77	3.42	0.01	ENSMUSG00000030786
Parp3	9	1.46	1.78	3.43	0.01	ENSMUSG00000023249
Cd300c2	11	2.11	1.78	3.44	0.04	ENSMUSG00000044811
Tpm2	4	12.39	1.79	3.46	0.00	ENSMUSG00000028464
Tlr13	X	1.82	1.79	3.47	0.01	ENSMUSG00000033777
Ccn5	2	1.28	1.80	3.48	0.05	ENSMUSG00000027656
Myh11	16	2.06	1.80	3.48	0.01	ENSMUSG00000018830
Ms4a4a	19	6.12	1.80	3.49	0.02	ENSMUSG00000101389
Gpr153	4	1.06	1.81	3.50	0.02	ENSMUSG00000042804
C1qb	4	30.43	1.81	3.51	0.00	ENSMUSG00000036905
Acta2	19	196.49	1.82	3.53	0.00	ENSMUSG00000035783
Col11a1	3	16.35	1.82	3.53	0.00	ENSMUSG00000027966
Trabd2b	4	1.21	1.82	3.54	0.01	ENSMUSG00000070867
Pitx2	3	2.00	1.83	3.55	0.01	ENSMUSG00000028023
Clip3	7	2.10	1.84	3.58	0.01	ENSMUSG00000013921
A930033H14Rik	10	3.13	1.84	3.58	0.00	ENSMUSG00000090622
Masp1	16	6.52	1.85	3.60	0.00	ENSMUSG00000022887
P2ry6	7	2.57	1.86	3.62	0.00	ENSMUSG00000048779
Lcn2	2	2.70	1.89	3.70	0.01	ENSMUSG00000026822
Mafb	2	2.71	1.89	3.71	0.00	ENSMUSG00000074622
Zim1	7	5.50	1.90	3.73	0.00	ENSMUSG00000002266
Gm49450	15	27.19	1.93	3.80	0.04	ENSMUSG00000116358
Tbx20	9	1.82	1.93	3.80	0.00	ENSMUSG00000031965
Gm10131	8	2.99	1.93	3.81	0.03	ENSMUSG00000063412
Actg2	6	18.50	1.93	3.81	0.01	ENSMUSG00000059430
Tspo2	17	98.44	1.95	3.85	0.00	ENSMUSG00000023995
Lmod1	1	1.53	1.95	3.86	0.01	ENSMUSG00000048096
Ankrd1	19	5.68	1.95	3.88	0.01	ENSMUSG00000024803

Tgfbi	13	12.03	1.96	3.88	0.01	ENSMUSG00000035493
Spp1	5	9.83	1.96	3.89	0.00	ENSMUSG00000029304
C1qc	4	33.23	1.96	3.89	0.00	ENSMUSG00000036896
Slc15a3	19	1.94	1.98	3.95	0.01	ENSMUSG00000024737
Mdga1	17	1.03	1.98	3.95	0.00	ENSMUSG00000043557
Adgre1	17	5.96	1.98	3.95	0.00	ENSMUSG00000004730
Ccl9	11	2.62	1.99	3.96	0.00	ENSMUSG00000019122
Clec4a1	6	1.18	2.00	4.00	0.01	ENSMUSG00000049037
Oas1a	5	1.19	2.00	4.00	0.01	ENSMUSG00000052776
Nbl1	4	5.37	2.00	4.01	0.00	ENSMUSG00000041120
Camp	9	1.41	2.03	4.08	0.02	ENSMUSG00000038357
Snai2	16	1.47	2.06	4.16	0.02	ENSMUSG00000022676
Ifi207	1	3.08	2.06	4.16	0.00	ENSMUSG00000073490
Mpeg1	19	4.61	2.06	4.18	0.00	ENSMUSG00000046805
Gpnmb	6	65.25	2.06	4.18	0.00	ENSMUSG00000029816
Ms4a6b	19	1.48	2.08	4.22	0.01	ENSMUSG00000024677
Fcgr1	3	1.34	2.08	4.24	0.01	ENSMUSG00000015947
Mfap2	4	3.51	2.09	4.25	0.01	ENSMUSG00000060572
Adamts2	11	6.66	2.09	4.25	0.00	ENSMUSG00000036545
Tgfb2	1	2.96	2.09	4.26	0.00	ENSMUSG00000039239
Loxl1	9	5.62	2.10	4.28	0.00	ENSMUSG00000032334
Ccl3	11	3.02	2.10	4.29	0.01	ENSMUSG00000000982
Hand2	8	8.14	2.10	4.30	0.00	ENSMUSG00000038193
Cmklr1	5	3.31	2.12	4.34	0.00	ENSMUSG00000042190
Rtp4	16	1.06	2.12	4.35	0.02	ENSMUSG00000033355
Bcl2a1b	9	1.53	2.14	4.41	0.03	ENSMUSG00000089929
Gm5483	16	1.78	2.17	4.51	0.02	ENSMUSG00000079597
Dot1l	10	21.47	2.17	4.51	0.00	ENSMUSG00000061589
Adamts5	16	1.06	2.18	4.55	0.00	ENSMUSG00000022894
Trem2	17	8.00	2.19	4.56	0.00	ENSMUSG00000023992
Ccl2	11	3.55	2.19	4.57	0.01	ENSMUSG00000035385
Ptn	6	4.67	2.19	4.58	0.00	ENSMUSG00000029838
Ctss	3	17.03	2.22	4.65	0.00	ENSMUSG00000038642

Capn6	X	1.08	2.22	4.67	0.00	ENSMUSG00000067276
Adamts15	9	1.12	2.23	4.68	0.00	ENSMUSG00000033453
Stfa2l1	16	2.57	2.25	4.76	0.01	ENSMUSG00000059657
Adam12	7	2.54	2.27	4.84	0.00	ENSMUSG00000054555
Cd200	16	1.04	2.28	4.87	0.02	ENSMUSG00000022661
Adcy3	12	1.21	2.30	4.91	0.00	ENSMUSG00000020654
Cthrc1	15	4.53	2.30	4.92	0.00	ENSMUSG00000054196
Mamdc2	19	2.97	2.30	4.92	0.00	ENSMUSG00000033207
Igf1	10	1.02	2.31	4.95	0.00	ENSMUSG00000020053
Mmp8	9	1.78	2.32	5.00	0.00	ENSMUSG00000005800
Hk3	13	2.34	2.35	5.08	0.00	ENSMUSG00000025877
Ms4a6c	19	3.56	2.36	5.14	0.00	ENSMUSG00000079419
Ifi27l2a	12	2.38	2.37	5.18	0.01	ENSMUSG00000079017
Ms4a6d	19	5.03	2.38	5.20	0.00	ENSMUSG00000024679
Ngp	9	6.23	2.39	5.25	0.00	ENSMUSG00000032484
Igsf6	7	1.89	2.39	5.26	0.00	ENSMUSG00000035004
Spon1	7	1.31	2.41	5.31	0.00	ENSMUSG00000038156
Ifit3	19	1.89	2.42	5.34	0.00	ENSMUSG00000074896
Nrep	18	3.31	2.43	5.38	0.00	ENSMUSG00000042834
Inhba	13	3.11	2.52	5.75	0.00	ENSMUSG00000041324
Cdh2	18	8.44	2.53	5.77	0.00	ENSMUSG00000024304
S100a9	3	18.07	2.57	5.95	0.00	ENSMUSG00000056071
Pitx1	13	1.47	2.77	6.81	0.00	ENSMUSG00000021506
Nacad	11	10.21	2.79	6.90	0.00	ENSMUSG00000041073
BC100530	16	3.96	2.86	7.26	0.00	ENSMUSG00000071561
Grem2	1	2.86	3.19	9.10	0.00	ENSMUSG00000050069
S100a8	3	15.00	3.19	9.13	0.00	ENSMUSG00000056054
3830417A13Rik	X	1.08	3.19	9.14	0.00	ENSMUSG00000031179
Rgs5	1	1.29	3.22	9.30	0.00	ENSMUSG00000026678
Ftl1-ps1	13	503.73	3.46	11.02	0.00	ENSMUSG00000062382
Erv3	2	1.74	3.48	11.18	0.00	ENSMUSG00000037482
Hoxa11	6	1.06	3.61	12.20	0.00	ENSMUSG00000038210
Enho	4	1.86	3.84	14.32	0.00	ENSMUSG00000028445

Fcgr4	1	1.58	3.91	15.01	0.00	ENSMUSG00000059089
Prtn3	10	1.73	4.25	18.96	0.00	ENSMUSG00000057729
Mpo	11	5.30	4.26	19.10	0.00	ENSMUSG00000009350
Ctsj	13	2.73	6.79	110.40	0.00	ENSMUSG00000055298
Ctsq	13	1.47	8.50	362.79	0.00	ENSMUSG00000021439

Supplemental Table 13. Downregulated genes in D*ot1L-* MM ESRE cells compared to *Dot1L-* KO cells (n=126)

Name	Chromosome	Max group mean	Log_2 fold change	Fold change	P-value	ENSEMBL
Sik1	17	10.26	-1.00	-2.00	0.02	ENSMUSG00000024042
Nsmf	2	11.16	-1.01	-2.01	0.03	ENSMUSG00000006476
Cdkn1a	17	367.92	-1.01	-2.02	0.03	ENSMUSG00000023067
Serinc2	4	8.08	-1.03	-2.04	0.03	ENSMUSG00000023232
Amot	X	20.53	-1.03	-2.04	0.03	ENSMUSG00000041688
Cebpa	7	47.32	-1.04	-2.05	0.03	ENSMUSG00000034957
Psrc1	3	51.32	-1.04	-2.06	0.00	ENSMUSG00000068744
Met	6	2.63	-1.09	-2.12	0.03	ENSMUSG00000009376
Cpa3	3	61.25	-1.10	-2.14	0.01	ENSMUSG00000001865
Plk2	13	15.29	-1.10	-2.14	0.03	ENSMUSG00000021701
Mycn	12	5.46	-1.11	-2.16	0.01	ENSMUSG00000037169
Arl4c	1	9.07	-1.11	-2.16	0.01	ENSMUSG00000049866
Fosl1	19	14.26	-1.12	-2.17	0.03	ENSMUSG00000024912
Apol11b	15	9.95	-1.12	-2.18	0.02	ENSMUSG00000091694
Tspan13	12	6.08	-1.13	-2.19	0.05	ENSMUSG00000020577
Gm47283	Y	142.59	-1.15	-2.21	0.00	ENSMUSG00000096768
Sh3bgrl2	9	2.42	-1.14	-2.21	0.02	ENSMUSG00000032261
Cttnbp2	6	1.46	-1.16	-2.23	0.04	ENSMUSG00000000416
H2-T24	17	18.52	-1.17	-2.25	0.01	ENSMUSG00000053835
Ctsc	7	8.64	-1.18	-2.27	0.03	ENSMUSG00000030560
Nyx	X	7.63	-1.22	-2.32	0.01	ENSMUSG00000051228
Tecta	9	1.48	-1.22	-2.33	0.04	ENSMUSG00000037705
H2-T23	17	55.62	-1.22	-2.34	0.00	ENSMUSG00000067212
Gm10638	8	11.12	-1.23	-2.34	0.02	ENSMUSG00000074178
Zfp37	4	2.70	-1.27	-2.42	0.03	ENSMUSG00000028389
Nyap1	5	2.29	-1.28	-2.43	0.04	ENSMUSG00000045348
Aqp8	7	8.49	-1.29	-2.44	0.01	ENSMUSG00000030762
Eda2r	X	8.79	-1.30	-2.46	0.00	ENSMUSG00000034457
Morc4	X	15.50	-1.30	-2.46	0.05	ENSMUSG00000031434
Alox12	11	4.87	-1.33	-2.52	0.01	ENSMUSG00000000320

Abcb1b	5	4.27	-1.37	-2.58	0.04	ENSMUSG00000028970
Nuak2	1	4.73	-1.38	-2.60	0.00	ENSMUSG00000009772
Gm8797	3	52.04	-1.39	-2.62	0.03	ENSMUSG00000103034
Vegfa	17	15.21	-1.41	-2.65	0.00	ENSMUSG00000023951
Cited1	X	9.61	-1.41	-2.65	0.03	ENSMUSG00000051159
Maob	X	3.82	-1.41	-2.66	0.02	ENSMUSG00000040147
Ak4	4	1.45	-1.42	-2.68	0.05	ENSMUSG00000028527
Rtn4rl2	2	21.73	-1.49	-2.80	0.00	ENSMUSG00000050896
Espn	4	3.14	-1.48	-2.80	0.00	ENSMUSG00000028943
Gm16867	14	17.88	-1.48	-2.80	0.05	ENSMUSG00000093954
Cldn4	5	118.71	-1.49	-2.80	0.05	ENSMUSG00000047501
Kcnh3	15	1.88	-1.50	-2.83	0.02	ENSMUSG00000037579
Fam102a	2	4.60	-1.52	-2.87	0.04	ENSMUSG00000039157
Dsg2	18	6.01	-1.54	-2.90	0.05	ENSMUSG00000044393
Muc13	16	15.48	-1.55	-2.93	0.05	ENSMUSG00000022824
Zfp750	11	13.71	-1.60	-3.02	0.00	ENSMUSG00000039238
Papln	12	3.19	-1.63	-3.09	0.05	ENSMUSG00000021223
F2rl1	13	5.18	-1.64	-3.11	0.05	ENSMUSG00000021678
Myh14	7	15.39	-1.64	-3.11	0.05	ENSMUSG00000030739
Itpk1	12	10.33	-1.67	-3.18	0.02	ENSMUSG00000057963
Lrp2	2	6.25	-1.67	-3.19	0.03	ENSMUSG00000027070
Kng1	16	25.25	-1.68	-3.20	0.05	ENSMUSG00000022875
Ntn4	10	1.39	-1.73	-3.31	0.01	ENSMUSG00000020019
Grin2d	7	1.53	-1.73	-3.31	0.03	ENSMUSG00000002771
Plet1	9	39.77	-1.73	-3.32	0.02	ENSMUSG00000032068
Alpk3	7	2.09	-1.74	-3.34	0.02	ENSMUSG00000038763
Tpsab1	17	22.71	-1.77	-3.41	0.00	ENSMUSG00000024173
Car4	11	9.39	-1.78	-3.44	0.04	ENSMUSG00000000805
Amn	12	20.34	-1.81	-3.49	0.05	ENSMUSG00000021278
Fam180a	6	2.27	-1.83	-3.55	0.02	ENSMUSG00000047420
Sprr1a	3	39.45	-1.86	-3.62	0.04	ENSMUSG00000050359
4930539E08Rik	17	5.09	-1.86	-3.64	0.04	ENSMUSG00000048905
Hpn	7	4.06	-1.91	-3.75	0.02	ENSMUSG00000001249

Tjp3	10	4.52	-1.93	-3.82	0.04	ENSMUSG00000034917
Slc3a1	17	1.08	-1.94	-3.83	0.04	ENSMUSG00000024131
Igsf23	7	4.83	-1.94	-3.83	0.04	ENSMUSG00000040498
Slc15a1	14	1.67	-1.95	-3.86	0.05	ENSMUSG00000025557
Sema4g	19	5.18	-1.95	-3.87	0.04	ENSMUSG00000025207
Apoc2	7	58.65	-1.96	-3.90	0.04	ENSMUSG00000002992
Hist1h1a	13	4.31	-1.98	-3.96	0.02	ENSMUSG00000049539
Abcc2	19	2.95	-1.99	-3.98	0.01	ENSMUSG00000025194
Mcoln3	3	2.98	-1.99	-3.98	0.02	ENSMUSG00000036853
Nt5e	9	2.71	-2.01	-4.02	0.01	ENSMUSG00000032420
Cblc	7	1.66	-2.02	-4.04	0.04	ENSMUSG00000040525
Myl7	11	2.26	-2.02	-4.05	0.02	ENSMUSG00000020469
Igfbp1	11	47.91	-2.04	-4.11	0.03	ENSMUSG00000020429
Cubn	2	12.26	-2.04	-4.12	0.01	ENSMUSG00000026726
Mid1	X	3.75	-2.08	-4.22	0.00	ENSMUSG00000035299
Bglap3	3	4.29	-2.08	-4.23	0.05	ENSMUSG00000074489
Clmn	12	2.22	-2.10	-4.28	0.02	ENSMUSG00000021097
Foxj1	11	1.12	-2.10	-4.28	0.03	ENSMUSG00000034227
Gjb1	X	2.71	-2.11	-4.32	0.05	ENSMUSG00000047797
Cfi	3	20.88	-2.13	-4.37	0.02	ENSMUSG00000058952
Apoa2	1	75.27	-2.13	-4.38	0.02	ENSMUSG00000005681
Vtn	11	4.77	-2.13	-4.38	0.05	ENSMUSG00000017344
Habp2	19	4.77	-2.16	-4.47	0.02	ENSMUSG00000025075
Fetub	16	4.10	-2.16	-4.47	0.03	ENSMUSG00000022871
Rasd2	8	3.67	-2.17	-4.49	0.01	ENSMUSG00000034472
Pcsk9	4	3.52	-2.17	-4.51	0.02	ENSMUSG00000044254
Lamb3	1	3.62	-2.17	-4.51	0.02	ENSMUSG00000026639
Slc7a9	7	2.52	-2.17	-4.51	0.03	ENSMUSG00000030492
Sprr2a1	3	3.68	-2.18	-4.53	0.04	ENSMUSG00000078664
Trf	9	828.61	-2.19	-4.55	0.02	ENSMUSG00000032554
Serpina1a	12	5.39	-2.19	-4.58	0.02	ENSMUSG00000066366
Apob	12	44.31	-2.20	-4.60	0.01	ENSMUSG00000020609
Cadm4	7	1.58	-2.21	-4.62	0.02	ENSMUSG00000054793

Cpn1	19	4.16	-2.23	-4.69	0.03	ENSMUSG00000025196
Serpind1	16	3.58	-2.24	-4.71	0.04	ENSMUSG00000022766
Fgb	3	80.67	-2.26	-4.79	0.02	ENSMUSG00000033831
Ace	11	1.27	-2.27	-4.83	0.03	ENSMUSG00000020681
Ehf	2	1.17	-2.28	-4.87	0.03	ENSMUSG00000012350
Ckmt1	2	2.00	-2.30	-4.92	0.01	ENSMUSG00000000308
Ahsg	16	1.37	-2.30	-4.94	0.01	ENSMUSG00000022868
Scg2	1	2.78	-2.34	-5.08	0.00	ENSMUSG00000050711
1700007K13Rik	2	7.79	-2.38	-5.22	0.00	ENSMUSG00000026831
Rarres2	6	3.21	-2.41	-5.32	0.05	ENSMUSG00000009281
Ggt1	10	3.95	-2.41	-5.33	0.01	ENSMUSG00000006345
RPS10-NUDT3	17	1.18	-2.43	-5.40	0.05	ENSMUSG00000117338
A2m	6	6.28	-2.48	-5.57	0.02	ENSMUSG00000030111
Fga	3	58.77	-2.51	-5.70	0.01	ENSMUSG00000028001
Npr1	3	1.56	-2.56	-5.91	0.01	ENSMUSG00000027931
Snhg11	2	1.01	-2.58	-5.98	0.00	ENSMUSG00000044349
Serpina1d	12	1.11	-2.63	-6.19	0.02	ENSMUSG00000071177
Plg	17	2.57	-2.67	-6.38	0.01	ENSMUSG00000059481
Efna3	3	1.09	-2.67	-6.38	0.01	ENSMUSG00000028039
Sprr2a2	3	60.77	-2.80	-6.95	0.01	ENSMUSG00000068893
Steap4	5	1.93	-2.88	-7.37	0.02	ENSMUSG00000012428
Sprr2a3	3	4.96	-2.92	-7.55	0.01	ENSMUSG00000074445
Comp	8	3.42	-2.96	-7.79	0.00	ENSMUSG00000031849
Efna1	3	8.71	-3.03	-8.16	0.00	ENSMUSG00000027954
Slc38a3	9	1.84	-3.03	-8.17	0.01	ENSMUSG00000010064
Fgg	3	96.62	-3.15	-8.88	0.00	ENSMUSG00000033860
C4b	17	1.45	-3.60	-12.15	0.00	ENSMUSG00000073418
Apln	X	1.06	-3.69	-12.91	0.00	ENSMUSG00000037010
H2-Q10	17	5.76	-3.74	-13.32	0.00	ENSMUSG00000067235
Cyp21a1	17	14.41	-3.90	-14.89	0.00	ENSMUSG00000024365

Supplemental Table 14. Differentiation of hematopoietic progenitor cells (*Dot1L*-KO)

Genes in dataset	Prediction	Expr Fold Change	Findings	ENSEMBL ID
CD79A	Affected	17.39	Affects (1)	ENSMUSG00000003379
CEBPA	Increased	13.87	Increases (11)	ENSMUSG00000034957
FOSL1	Increased	11.80	Increases (2)	ENSMUSG00000024912
NOG	Increased	9.55	Increases (1)	ENSMUSG00000048616
CDKN2B	Increased	9.47	Increases (1)	ENSMUSG00000073802
HIST1H3F	Affected	7.90	Affects (1)	ENSMUSG00000101972
HIST1H4L	Decreased	7.60	Decreases (2)	ENSMUSG00000069305
JUN	Decreased	7.48	Decreases (1)	ENSMUSG00000052684
HIST1H3A	Affected	6.20	Affects (1)	ENSMUSG00000099583
JUNB	Increased	6.06	Increases (1)	ENSMUSG00000052837
CDKN1A	Increased	5.94	Increases (1)	ENSMUSG00000023067
JAG1	Increased	5.92	Increases (1)	ENSMUSG00000027276
HSD3B1	Increased	5.85	Increases (1)	ENSMUSG00000027869
PNP	Increased	5.40	Increases (1)	ENSMUSG00000068417
LOX	Affected	5.23	Affects (1)	ENSMUSG00000024529
HIST4H4	Decreased	4.76	Decreases (2)	ENSMUSG00000060639
CSF2RB	Affected	4.76	Affects (2)	ENSMUSG00000071714
FN1	Increased	4.47	Increases (1)	ENSMUSG00000026193
NFKBIA	Increased	4.40	Increases (2)	ENSMUSG00000021025
PPP1R15A	Decreased	3.63	Decreases (1)	ENSMUSG00000040435
EPHA4	Increased	3.59	Increases (1)	ENSMUSG00000026235
BTG2	Increased	3.41	Increases (1)	ENSMUSG00000020423
MAFK	Increased	3.06	Increases (1)	ENSMUSG00000018143
CDKN1B	Increased	2.79	Increases (2)	ENSMUSG00000003031
VEGFA	Increased	2.73	Increases (4)	ENSMUSG00000023951
EGR1	Increased	2.69	Increases (2)	ENSMUSG00000038418
BCL2L1	Increased	2.64	Increases (10)	ENSMUSG00000007659
MAFG	Increased	2.19	Increases (1)	ENSMUSG00000051510
RAF1	Decreased	2.07	Decreases (2)	ENSMUSG00000000441
RUNX1T1	Decreased	2.05	Decreases (1)	ENSMUSG00000006586

HMGA1	Decreased	-2.32	Increases (3)	ENSMUSG00000046711
CDK6	Affected	-2.46	Affects (2)	ENSMUSG00000040274
GP1BA	Affected	-2.83	Affects (2)	ENSMUSG00000050675
PRKCA	Increased	-3.22	Decreases (1)	ENSMUSG00000050965
FLI1	Increased	-3.29	Decreases (6)	ENSMUSG00000016087
PLCG2	Decreased	-3.98	Increases (2)	ENSMUSG00000034330
Meis1	Increased	-4.18	Decreases (1)	ENSMUSG00000020160
MEF2C	Affected	-4.55	Affects (1)	ENSMUSG00000005583
S100A8	Increased	-5.08	Decreases (2)	ENSMUSG00000056054
MPL	Affected	-6.30	Affects (1)	ENSMUSG00000006389
CSF3R	Affected	-6.92	Affects (2)	ENSMUSG00000028859
ZNF616	Affected	-11.09	Affects (1)	ENSMUSG00000066000
HOXA9	Affected	-38.02	Affects (3)	ENSMUSG00000038227
FLT3	Decreased	-70.41	Increases (8)	ENSMUSG00000042817

Supplemental Table 15. Differentiation of hematopoietic progenitor cells (*Dot1L* -MM)

Genes in dataset	Prediction	Expr Fold Change	Findings	ENSEMBL ID
MYH11	Decreased	16.522	Decreases (2)	ENSMUSG00000018830
INHBA	Increased	13.912	Increases (5)	ENSMUSG00000041324
SIGLEC10	Affected	10.064	Affects (1)	ENSMUSG00000030468
CXCL12	Increased	9.177	Increases (3)	ENSMUSG00000061353
THBS2	Affected	8.086	Affects (1)	ENSMUSG00000023885
HIST1H3F	Affected	7.995	Affects (1)	ENSMUSG00000101972
LOX	Affected	7.451	Affects (1)	ENSMUSG00000024529
JAG1	Increased	6.849	Increases (1)	ENSMUSG00000027276
HIST1H3A	Affected	6.699	Affects (1)	ENSMUSG00000099583
CEBPA	Increased	6.468	Increases (11)	ENSMUSG00000034957
PITX2	Increased	6.406	Increases (1)	ENSMUSG00000028023
CSF1	Increased	6.397	Increases (6)	ENSMUSG00000014599
MAFB	Affected	5.702	Affects (3)	ENSMUSG00000074622
AXL	Increased	5.66	Increases (2)	ENSMUSG00000002602
FSTL3	Affected	5.611	Affects (1)	ENSMUSG00000020325
JUN	Decreased	5.477	Decreases (1)	ENSMUSG00000052684
CDKN2B	Increased	5.446	Increases (1)	ENSMUSG00000073802
FOSL1	Increased	5.189	Increases (2)	ENSMUSG00000024912
FN1	Increased	4.99	Increases (1)	ENSMUSG00000026193
HSD3B1	Increased	4.856	Increases (1)	ENSMUSG00000027869
MYL9	Increased	4.848	Increases (2)	ENSMUSG00000067818
PNP	Increased	4.753	Increases (1)	ENSMUSG00000068417
PALLD	Increased	4.258	Increases (1)	ENSMUSG00000058056
PTGS2	Affected	4.228	Affects (1)	ENSMUSG00000032487
Cdkn1c	Increased	3.92	Increases (3)	ENSMUSG00000037664
CSF2RB	Affected	3.612	Affects (2)	ENSMUSG00000071714
JUNB	Increased	3.534	Increases (1)	ENSMUSG00000052837
Marcks	Increased	3.479	Increases (1)	ENSMUSG00000069662
HIST4H4	Decreased	3.272	Decreases (2)	ENSMUSG00000060639
BTG2	Increased	2.838	Increases (1)	ENSMUSG00000020423

CDKN1A	Increased	2.804	Increases (1)	ENSMUSG00000023067
CDKN1B	Increased	2.793	Increases (2)	ENSMUSG00000003031
NFKBIA	Increased	2.777	Increases (2)	ENSMUSG00000021025
IL6R	Increased	2.694	Increases (4)	ENSMUSG00000027947
MAFK	Increased	2.471	Increases (1)	ENSMUSG00000018143
CDK6	Affected	-2.14	Affects (2)	ENSMUSG00000040274
KIT	Affected	-2.299	Affects (3)	ENSMUSG00000005672
ITGA2B	Affected	-2.693	Affects (1)	ENSMUSG00000034664
PLCG2	Decreased	-2.861	Increases (2)	ENSMUSG00000034330
MEF2C	Affected	-3.028	Affects (1)	ENSMUSG00000005583
PF4	Increased	-3.171	Decreases (2)	ENSMUSG00000029373
PECAM1	Increased	-3.333	Decreases (6)	ENSMUSG00000020717
PRKCA	Increased	-3.661	Decreases (1)	ENSMUSG00000050965
FLI1	Increased	-3.877	Decreases (6)	ENSMUSG00000016087
GP1BA	Affected	-4.065	Affects (2)	ENSMUSG00000050675
HMGA1	Decreased	-4.171	Increases (3)	ENSMUSG00000078249
LAT	Decreased	-4.306	Increases (3)	ENSMUSG00000030742
MPIG6B	Affected	-4.748	Affects (2)	ENSMUSG00000073414
ZNF616	Affected	-6.041	Affects (1)	ENSMUSG00000066000
MPL	Affected	-8.076	Affects (1)	ENSMUSG00000006389

Supplemental Table 16. Proliferation of hematopoietic progenitor cells (*Dot1L* -KO)				
**Genes in dataset**	**Prediction**	**Expr Fold Change**	**Findings**	**ENSEMBL ID**
CD79A	Increased	17.39	Increases (5)	ENSMUSG00000003379
GDF15	Decreased	17.14	Decreases (1)	ENSMUSG00000038508
CEBPA	Decreased	13.87	Decreases (3)	ENSMUSG00000034957
JUNB	Decreased	6.06	Decreases (4)	ENSMUSG00000052837
GSTP1	Decreased	6.04	Decreases (5)	ENSMUSG00000038155
CDKN1A	Decreased	5.94	Decreases (1)	ENSMUSG00000023067
BBC3	Increased	5.56	Increases (1)	ENSMUSG00000002083
LOX	Increased	5.23	Increases (1)	ENSMUSG00000024529
TP53INP1	Decreased	4.76	Decreases (1)	ENSMUSG00000028211
CSF2RB	Affected	4.76	Affects (1)	ENSMUSG00000071714
NFKBIA	Affected	4.40	Affects (2)	ENSMUSG00000021025
RHBDF2	Decreased	4.04	Decreases (1)	ENSMUSG00000020806
MDM2	Increased	3.69	Increases (4)	ENSMUSG00000020184
IL4R	Increased	3.22	Increases (7)	ENSMUSG00000030748
AGRN	Increased	3.20	Increases (1)	ENSMUSG00000041936
TNFAIP3	Decreased	3.12	Decreases (1)	ENSMUSG00000019850
PIK3R1	Increased	3.07	Increases (1)	ENSMUSG00000041417
CEBPB	Increased	2.89	Increases (1)	ENSMUSG00000056501
BTG1	Decreased	2.85	Decreases (1)	ENSMUSG00000036478
LPIN1	Decreased	2.80	Decreases (1)	ENSMUSG00000020593
CDKN1B	Decreased	2.79	Decreases (8)	ENSMUSG00000003031
VEGFA	Increased	2.73	Increases (5)	ENSMUSG00000023951
EGR1	Increased	2.69	Increases (1)	ENSMUSG00000038418
BCL2L1	Increased	2.64	Increases (6)	ENSMUSG00000007659
FGFR1	Affected	2.59	Affects (1)	ENSMUSG00000031565
IFNAR2	Increased	2.59	Increases (2)	ENSMUSG00000022971
PIM1	Increased	2.47	Increases (2)	ENSMUSG00000024014
GRAP2	Increased	2.20	Increases (1)	ENSMUSG00000042351
MAFG	Decreased	2.19	Decreases (1)	ENSMUSG00000051510
RAF1	Increased	2.07	Increases (1)	ENSMUSG00000000441

RUNX1T1	Increased	2.05	Increases (2)	ENSMUSG00000006586
SHB	Affected	2.03	Affects (1)	ENSMUSG00000044813
FLI1	Decreased	-3.29	Increases (2)	ENSMUSG00000016087
Meis1	Decreased	-4.18	Increases (1)	ENSMUSG00000020160
MEF2C	Decreased	-4.55	Increases (2)	ENSMUSG00000005583
MPL	Increased	-6.30	Decreases (9)	ENSMUSG00000006389
CSF3R	Affected	-6.92	Affects (1)	ENSMUSG00000028859
HOXA9	Decreased	-38.02	Increases (4)	ENSMUSG00000038227
FLT3	Decreased	-70.41	Increases (18)	ENSMUSG00000042817

Supplemental Table 17. Development of hematopoietic progenitor cells (Dot1L-MM)

Genes in dataset	Prediction	Expr Fold Change	Findings	ENSEMBL ID
INHBA	Increased	13.912	Increases (2)	ENSMUSG00000041324
THBS2	Affected	8.086	Affects (1)	ENSMUSG00000023885
HIST1H3F	Affected	7.995	Affects (1)	ENSMUSG00000101972
LOX	Affected	7.451	Affects (1)	ENSMUSG00000024529
JAG1	Increased	6.849	Increases (1)	ENSMUSG00000027276
HIST1H3A	Affected	6.699	Affects (1)	ENSMUSG00000099583
CEBPA	Increased	6.468	Increases (1)	ENSMUSG00000034957
CSF1	Increased	6.397	Increases (29)	ENSMUSG00000014599
FNDC5	Increased	6.351	Increases (1)	ENSMUSG00000001334
AXL	Increased	5.66	Increases (1)	ENSMUSG00000002602
CDKN2B	Increased	5.446	Increases (1)	ENSMUSG00000073802
FN1	Increased	4.99	Increases (1)	ENSMUSG00000026193
HSD3B1	Increased	4.856	Increases (1)	ENSMUSG00000027869
MYL9	Increased	4.848	Increases (2)	ENSMUSG00000067818
NFIL3	Increased	3.925	Increases (2)	ENSMUSG00000056749
CSF2RB	Affected	3.612	Affects (5)	ENSMUSG00000071714
LGMN	Decreased	3.525	Decreases (3)	ENSMUSG00000021190
Marcks	Increased	3.479	Increases (1)	ENSMUSG00000069662
HIST4H4	Decreased	3.272	Decreases (2)	ENSMUSG00000060639
CDKN1A	Increased	2.804	Increases (1)	ENSMUSG00000023067
NFKBIA	Increased	2.777	Increases (2)	ENSMUSG00000021025
IL6R	Increased	2.694	Increases (1)	ENSMUSG00000027947
MAFK	Increased	2.471	Increases (1)	ENSMUSG00000018143
KIT	Affected	-2.299	Affects (1)	ENSMUSG00000005672
ITGA2B	Affected	-2.693	Affects (1)	ENSMUSG00000034664
MEF2C	Affected	-3.028	Affects (1)	ENSMUSG00000005583
ITGAL	Decreased	-3.13	Increases (1)	ENSMUSG00000030830
PF4	Increased	-3.171	Decreases (2)	ENSMUSG00000029373
PECAM1	Increased	-3.333	Decreases (6)	ENSMUSG00000020717
FLI1	Decreased	-3.877	Increases (3)	ENSMUSG00000016087

GP1BA	Affected	-4.065	Affects (2)	ENSMUSG00000050675
HMGA1	Increased	-4.171	Decreases (1)	ENSMUSG00000078249
MPIG6B	Affected	-4.748	Affects (2)	ENSMUSG00000073414
MPL	Affected	-8.076	Affects (1)	ENSMUSG00000006389
FLT3	Decreased	-12.33	Increases (1)	ENSMUSG00000042817
